# Synthesis of fluoroalkenes and fluoroenynes via cross-coupling reactions using novel multihalogenated vinyl ethers

**DOI:** 10.3762/bjoc.20.226

**Published:** 2024-10-24

**Authors:** Yukiko Karuo, Keita Hirata, Atsushi Tarui, Kazuyuki Sato, Kentaro Kawai, Masaaki Omote

**Affiliations:** 1 Faculty of Pharmaceutical Sciences, Setsunan University, 45-1 Nagaotoge-cho, Hirakata, Osaka 573-0101, Japan

**Keywords:** fluoroalkenes, fluoroenynes, multihalogenated vinyl ethers, Suzuki–Miyaura cross-coupling reactions, Sonogashira cross-coupling reactions

## Abstract

In this study, we develop the synthesis methods of fluoroalkenes and fluoroenynes via Suzuki–Miyaura and Sonogashira cross-coupling reactions using novel multihalogenated fluorovinyl ethers, which are easily prepared from the reaction between phenols and 2-bromo-2-chloro-1,1,1-trifluoroethane (halothane). These reactions make use of the unique structure of multihalogenated fluorovinyl ethers, which contains a reactive bromine atom, to afford a series of fluoroalkenes and fluoroenynes in moderate to high yields.

## Introduction

Fluoroalkenes are one of the important frameworks for a wide range of industrial fields. For example, they are used as a bioisostere of amide bonds in medicines and agrochemicals, and contribute to the synthesis of peptide medicines that are stable to enzymatic metabolism and possess high lipophilicity [1]. In fact, several inhibitors of the β-site amyloyd β A4 precursor protein cleaving enzyme (BACE1), which is involved in the production of β-amyloid, and fluoroalkene analogs of dipeptidyl peptidase-4 inhibitors have previously been reported [2–3]. These inhibitors possess higher drug efficacies than their parent compounds. Furthermore, fluoroalkenes can be utilized as feedstock for fluoropolymers. Teflon, which is a well-known fluoropolymer with excellent water-repellent and oleophobic properties, is synthesized by polymerizing a monomer called tetrafluoroethylene. As a consequence, convenient and diverse synthetic methods for fluoroalkenes have attracted considerably and become increasingly necessary in pharmaceutical and industrial fields.

**Scheme 1:**
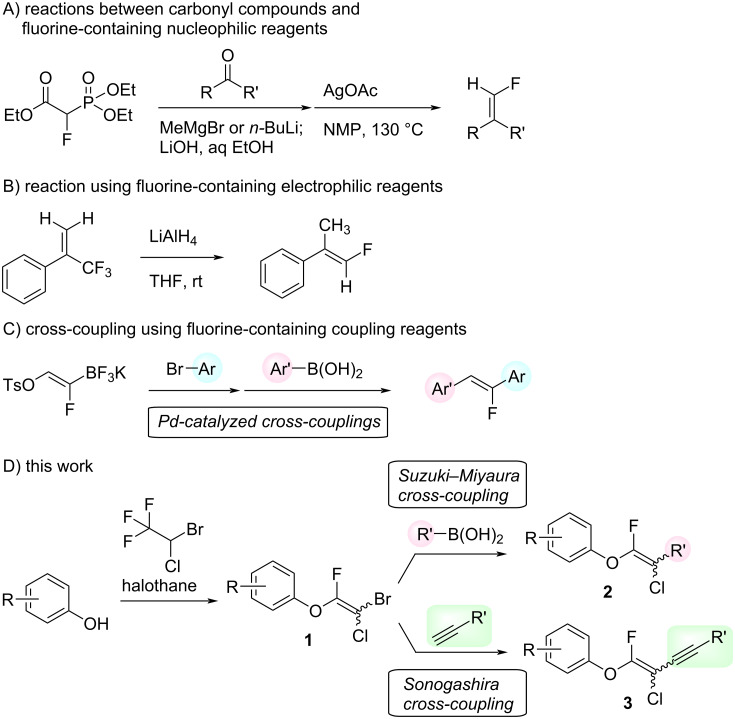
Synthesis of monofluoroalkenes using fluorine-containing building blocks.

Fluoroalkenes have been constructed in a variety of methods [4–14], and one of the methods is to make use of fluorine-containing building blocks. When using them as nucleophilic reagents [15–20], the reaction between anion species, such as fluorine-containing Horner–Wadsworth–Emmons reagents, and carbonyl compounds led to *E*-selective olefination (Scheme 1A) [15]. On the other hand, some reactions with electrophilic fluorine-containing building blocks have been developed [21–25]. Jubault and Poisson et al. reported S_N_2’ reactions of hydride or alcohols to electrophilic fluorine-containing alkenes gave the corresponding fluoroalkenes (Scheme 1B) [21]. In recent years, many fluorine-containing coupling reagents have been developed. These reagents are easily being converted into multisubstituted fluoroalkenes through cross-coupling using palladium, nickel, copper, ruthenium, and manganese catalysts [26–41]. Hosoya and Niwa et al. published the development of a dual-reactive fluorine-containing C2-unit, which was prepared from trifluoroethanol in two steps in 63% yield, allowed the convergent synthesis of fluoroalkenes (Scheme 1C) [26]. We recently found multihalogenated vinyl ethers **1** could be obtained by the reaction of phenols with 2-bromo-2-chloro-1,1,1-trifluoroethane (halothane) in good yields (Scheme 1D) [42]. Compound **1** has a unique structure possessing three types of halogen atoms, namely bromine, chlorine, and fluorine, and it would be expected to afford multisubstituted fluoroalkenes by installing various substituents to bromine or chlorine atoms as reported by Hosoya and Niwa et al. In this study, we investigated the synthesis of fluoroalkenes **2** or fluoroenynes **3** by Suzuki–Miyaura or Sonogashira cross-couplings with a key building block **1** (Scheme 1D).

## Results and Discussion

### Optimization of the conditions of cross-coupling reactions

First, we optimized the conditions of the Suzuki–Miyaura cross-coupling in reference to the report by Yang et al. (Table 1) [43]. Upon the treatment of multihalogenated vinyl ether **1a** with phenylboronic acid **4a** (1.3 equiv) and palladium diacetate (10 mol %) as a catalyst at 40 °C, Suzuki–Miyaura cross-coupling proceeded to produce fluoroalkene **2a** in 50% yield (Table 1, entry 1). Increasing the amount of **4a** to 2.0 equiv and decreasing the amount of palladium diacetate to 5 mol % improved the reaction yield (Table 1, entry 2). When the reaction mixture was heated to 60 °C or reflux conditions, **2a** could be synthesized in 84% yield under reflux conditions (Table 1, entries 3 and 4). Next, we examined an effective catalyst for the cross-coupling. Reactions using palladium dichloride or bis(2,4-pentanedionato)palladium significantly reduced the yields of **2a** (Table 1, entries 5 and 6, respectively). When an allylpalladium chloride dimer or bis(triphenylphosphine)palladium dichloride were used as catalyst, the reaction proceeded with the same yield as that in Table 1, entry 4 (entries 7 and 8). Utilizing palladium catalyst such as bis(triphenylphosphine)palladium dichloride, all these reactions could convert **1a** into **2a** in good yields (Table 1, entries 9–11). Cross-coupling with palladium bis(trifluoroacetate), which is more reactive than palladium diacetate, gave the corresponding product in high yield of 96% (Table 1, entry 12). Without the addition of triphenylphosphine, the reaction proceeded in only 12% yield (Table 1, entry 13). Thus, it was concluded that triphenylphosphine is necessary for Suzuki–Miyaura cross-coupling of **1** with **4** and that it is involved in the production of palladium(0).

**Table 1:** Optimization of reaction conditions for Suzuki–Miyaura cross-coupling using multihalogenated vinyl ether **1a**.

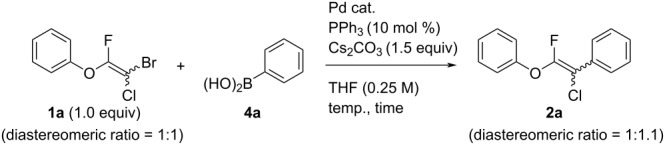					

Entry	**4a** (equiv)	Pd cat. (mol %)	Temp. (°C)	Time (h)	**2a** (%)^a^

1	1.3	Pd(OAc)_2_ (10)	40	3.0	50
2	2.0	Pd(OAc)_2_ (5)	40	2.5	73
3	2.0	Pd(OAc)_2_ (5)	60	2.5	68
4	2.0	Pd(OAc)_2_ (5)	reflux	3.5	84
5	2.0	PdCl_2_ (5)	reflux	3.5	61
6	2.0	Pd(acac)_2_ (5)	reflux	3.5	12
7	2.0	[Pd(allyl)Cl]_2_ (5)	reflux	3.5	81
8	2.0	Pd(PPh_3_)_2_Cl_2_ (5)	reflux	3.5	84
9	2.0	Pd[P(*o*-Tol)_3_]_2_Cl_2_ (5)	reflux	3.5	89
10	2.0	Pd(MeCN)_2_Cl_2_ (5)	reflux	3.5	93
11	2.0	Pd(PhCN)_2_Cl_2_ (5)	reflux	3.5	92
12	2.0	Pd(OCOCF_3_)_2_ (5)	reflux	3.5	96
13^b^	2.0	Pd(OCOCF_3_)_2_ (5)	reflux	3.5	12

^a^Isolated yields; ^b^no PPh_3_.

Next, the reaction conditions for the Sonogashira cross-coupling were optimized (Table 2). On the basis of a previous study by Thorand [44], we performed the reaction between fluorine-containing vinyl ether **1a** and 1.05 equiv of trimethylsilylacetylene (**5a**) to afford the corresponding enyne **3a** in 55% yield (Table 2, entry 1). Cross-coupling utilizing a palladium(II) catalysts containing phosphine ligands produced low yields of **3a** (Table 2, entries 2 and 3). In the case of palladium(II), which produced good yields of the Suzuki–Miyaura cross-coupling products, only a small amount of **3a** was obtained (Table 2, entries 4–8). In particular, when the allylpalladium dichloride dimer was used, Sonogashira coupling hardly proceeded at all, and the starting ether **1a** was recovered in an 83% yield (Table 2, entry 6). Zero-valent tetrakis(triphenylphosphine)palladium and tris(dibenzylideneacetone)dipalladium allowed the reaction to undergo in 37% or 23% yields, respectively (Table 2, entries 9 and 10). In entry 11, Table 2, we selected bis(triphenylphosphine)palladium as an effective catalyst, but increase of **5a** to 1.5 equiv did not improve the reaction yield. Diluting the reaction concentration from 0.83 M to 0.2 M achieved to give **3a** in a 63% yield (Table 2, entry 12). Increasing the amount of palladium catalyst to 4 mol % led to the conversion of **1a** into **3a** in 77% yield (Table 2, entry 13). In addition, using 2.0 equiv of **5a** gave **3a** in high 80% yield (Table 2, entry 14).

**Table 2:** Optimization of reaction conditions for Sonogashira cross-coupling using multihalogenated vinyl ether **1a**.

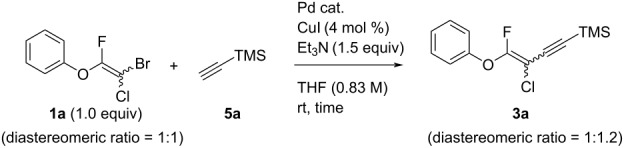				

Entry	**5a** (equiv)	Pd cat. (mol %)	Time (h)	**3a** (%)^a^

1	1.05	Pd(PPh_3_)_2_Cl_2_ (2)	6.5	55
2	1.05	Pd[P(*o*-Tol)_3_]_2_Cl_2_ (2)	24	14
3	1.05	Pd(PCy_3_)_2_Cl_2_ (2)	24	0
4	1.05	Pd(OAc)_2_ (2)	20	6
5	1.05	PdCl_2_ (2)	24	13
6^b^	1.05	[Pd(allyl)Cl]_2_ (2)	18.5	0
7	1.05	Pd(MeCN)_2_Cl_2_ (2)	25.5	13
8	1.05	Pd(PhCN)_2_Cl_2_ (2)	24	10
9	1.05	Pd(PPh_3_)_4_ (2)	21.5	37
10	1.05	Pd_2_(dba)_3_ (2)	24	23
11	1.5	Pd(PPh_3_)_2_Cl_2_ (2)	19	53
12^c^	1.5	Pd(PPh_3_)_2_Cl_2_ (2)	17	63
13^c^	1.5	Pd(PPh_3_)_2_Cl_2_ (4)	18	77
14^c^	2.0	Pd(PPh_3_)_2_Cl_2_ (4)	19	80

^a^Isolated yields; ^b^Recovery of **1a** was 83% yield; ^c^THF (0.2 M) was used.

Based on these results, we determined entry 13 in Table 1 and entry 14 in Table 2 as the optimum reaction conditions. We used **1** as a mixture of diastereomers (diastereomer ratio = 1:1) for cross-coupling, and the corresponding compounds **2** and **3** were obtained as mixtures of diastereomers in a certain ratio as estimated by proton and fluorine NMR spectroscopy.

### Substrate scope for cross-coupling reactions

The substrate scope was investigated using various boronic acids **4** and alkynes **5** in cross-coupling reactions using **1** (Table 3 and Table 4). *p*-Tolylboronic acid **4b** provided **2b** quantitatively, whereas *m*- and *o*-tolylboronic acids **4c** and **4d** produced **2c** and **2d** in low yields because the methyl group was positioned near the reaction site (Table 3, entries 1–3). Introduction of 3,4-methylenedioxyphenyl (**4e**) or *p*-fluorophenyl groups (**4f**) to **1a** proceeded in high yields (Table 3, entries 4 and 5). Boronic acids with carbonyl groups such as acetyl, ester or formyl moieties in *para* position (**4g**–**i**) underwent the cross-coupling in 76, 96 or 77% yields (Table 3, entries 6–8). The reaction between **1a** and **4j**, which contains an electron-withdrawing nitro group, afforded **2j** in 88% yield (Table 3, entry 9). Although *p*-hydroxyphenylboronic acid (**4k**) gave **2k** in only 9% yield, *m*-aminophenylboronic acid (**4l**) provided **2l** in high yield (Table 3, entries 10 and 11). We predicted that the product yield would decrease because **2k** is labile in column chromatography. Utilizing a boronic acid bearing an *n*-butyl group as a primary alkyl group (**4m**), the cross-coupling did not proceed due to β-elimination (Table 3, entry 12). In contrast, the reaction with cyclopropylboronic acid (**4n**) achieved to give **2n** in a 71% yield (Table 3, entry 13). When thiopheneboronic acid **4o** was used as a coupling partner, the thiophene ring could be installed on **1a** in a comparatively low yield of 31% (Table 3, entry 14). In addition, we investigated the substrate scope of **1** in the Suzuki–Miyaura cross-coupling. The reaction of **1b** or **1c**, which had a *m*-methoxy or *p*-nitro group on the benzene ring, with **4a** proceeded smoothly to furnish **2p** or **2q** in good yieds (Table 3, entries 15 and 16). A phenyl group could be introduced into **1d** possessing an ester moiety in moderate yield, whereas the cross-coupling between **1e**, derived from *m*-aminophenol, and **4a** proceeded in only 15% yield (Table 3, entries 17 and 18).

**Table 3:** Cross-coupling reactions between multihalogenated vinyl ethers **1** and various boronic acids **4**.

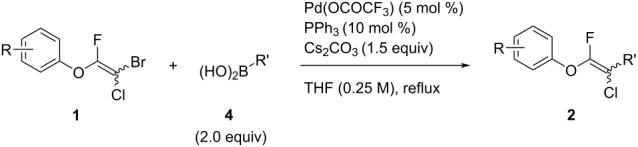					

Entry	**1** (diastereomeric ratio)	**4**	**2** (diastereomeric ratio)	Time (h)	Yield (%)^a^

1	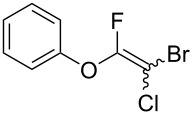 **1a** (1:1)	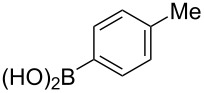 **4b**	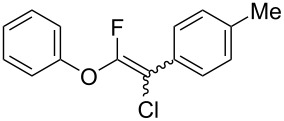 **2b** (1:1)	3.5	98
2^b^	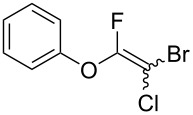 **1a** (1:1)	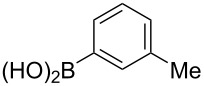 **4c**	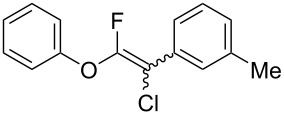 **2c** (1:1)	2.5	26
3	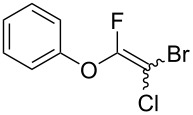 **1a** (1:1)	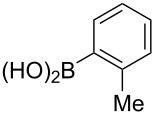 **4d**	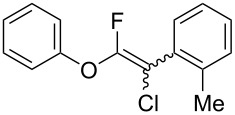 **2d** (1:1)	3.5	16
4	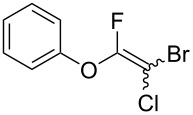 **1a** (1:1)	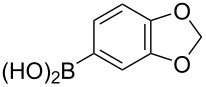 **4e**	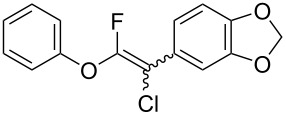 **2e** (1:1)	3.5	85
5	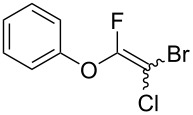 **1a** (1:1)	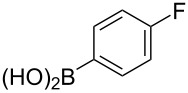 **4f**	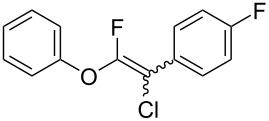 **2f** (1:1)	3.5	94
6	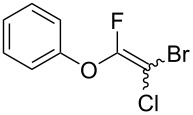 **1a** (1:1)	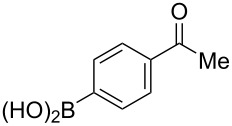 **4g**	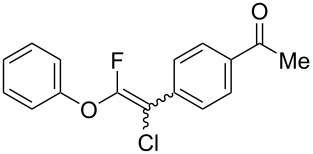 **2g** (1:1)	2.5	76
7	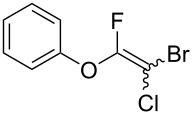 **1a** (1:1)	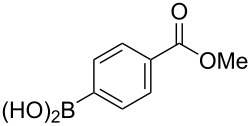 **4h**	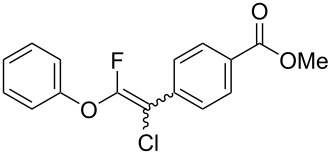 **2h** (1:1)	1.5	96
8	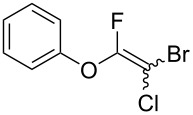 **1a** (1:1)	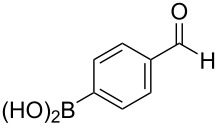 **4i**	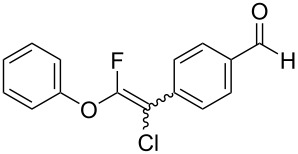 **2i** (1:1)	3.5	77
9	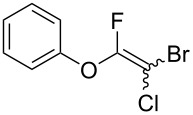 **1a** (1:1)	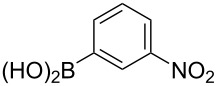 **4j**	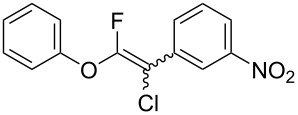 **2j** (1:1:1)	3.0	88
10	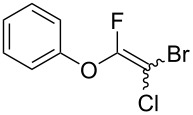 **1a** (1:1)	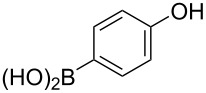 **4k**	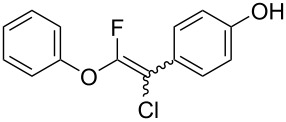 **2k** (1:1.4)	6.5	9
11	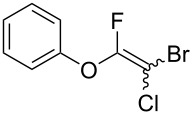 **1a** (1:1)	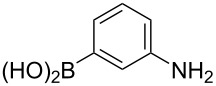 **4l**	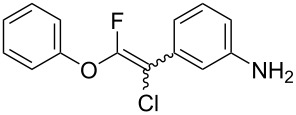 **2l** (1:1)	4.0	92
12	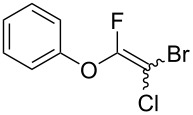 **1a** (1:1)	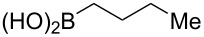 **4m**	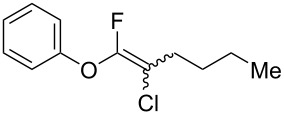 **2m** (1:1)	3.5	trace
13	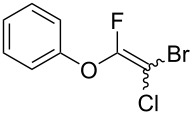 **1a** (1:1)	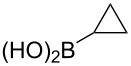 **4n**	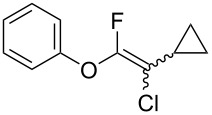 **2n** (1:1)	5.0	71
14	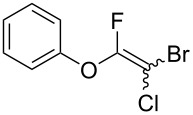 **1a** (1:1)	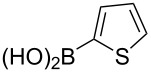 **4o**	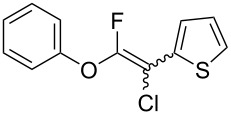 **2o** (1:1.1)	4.0	31
15	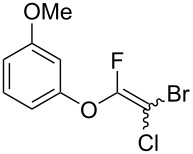 **1b** (1:1)	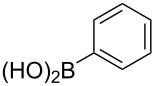 **4a**	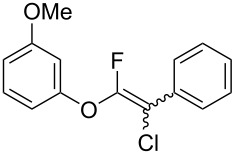 **2p** (1:1)	3.5	62
16	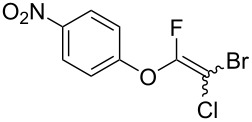 **1c** (1:1)	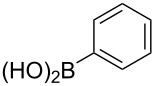 **4a**	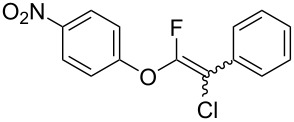 **2q** (1:1)	5.5	85
17	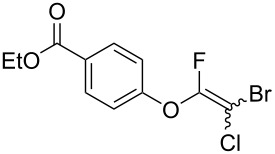 **1d** (1:1)	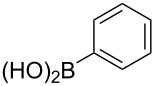 **4a**	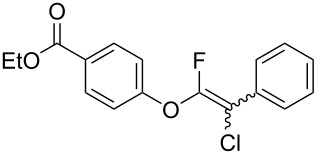 **2r** (1:1.6)	3.5	45
18^c^	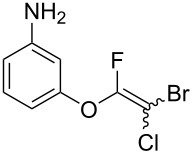 **1e** (1:1)	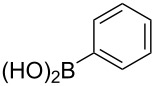 **4a**	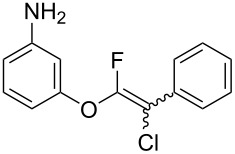 **2s** (1:1.2)	21.5	15

^a^Isolated yields; ^b^**1** (1.5 equiv) and **2** (1.0 equiv) were used; ^c^DME was used as a solvent.

We performed Sonogashira cross-couplings between **1** and a variety of alkynes **5** (Table 4). Arylacetylenes, which have electron-donating substituents on the aromatic ring (**5b**–**f**), and 2-naphthylacetylene (**5g**) provided the corresponding enynes (**3b**–**g**) in 43–92% yields (Table 4, entries 1–6). On the contrary, electron-withdrawing substituents such as chloro, trifluoromethyl and nitro groups resulted in low cross-coupling yields (**3h**–**j**) (Table 4, entries 7–9). *p*-Acetyl or *p*-formylphenylacetylene (**5k** or **5l**) could be introduced into **1a** in 76% or 52% yields, respectively (Table 4, entries 10 and 11). Reactions using acetylenes possessing a hydroxy group, amino group and thiophene proceeded well (**3m**–**o**) (Table 4, entries 12–14). Hexa-1-yne **5p** and cyclopropylacetylene (**5q**) afforded **3p** and **3q**, respectively, in high yields without byproduct formation (Table 4, entries 15 and 16). Enyne compound **5r** and 3-butyn-1-ol **5s** also participated in cross-coupling reactions and products **3r** and **3s** could be obtained in moderate yields of 52 and 53% (Table 4, entries 17 and 18). Then, we attempted the cross-coupling between **1** derived from various phenols and **5a**. Vinyl ethers **1b**–**d** were converted into enynes **3t**–**v** in 29–35% yields (Table 4, entries 19–21). The reaction using **1e**, which bears an amino group on the benzene ring, did not complete despite requiring a long reaction time (Table 4, entry 22).

**Table 4:** Cross-coupling reactions between multihalogenated vinyl ethers **1** and various alkynes **5**.

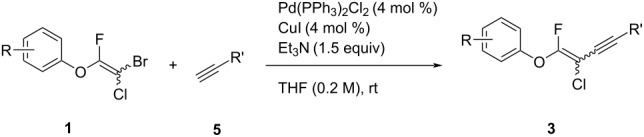					

Entry	**1** (diastereomeric ratio)	**5**	**3** (diastereomeric ratio)	Time (h)	Yield (%)^a^

1	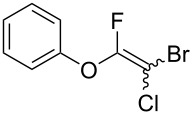 **1a** (1:1)	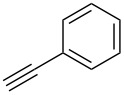 **5b**	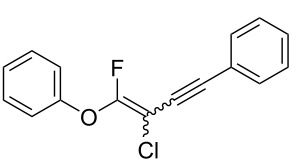 **3b** (1:1.1)	18.5	92
2^b^	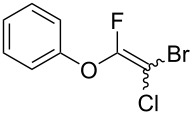 **1a** (1:1)	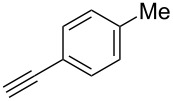 **5c**	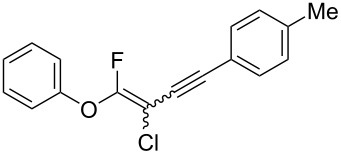 **3c** (1:1.4)	3.5	74
3^b^	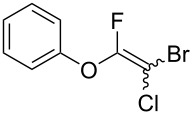 **1a** (1:1)	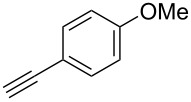 **5d**	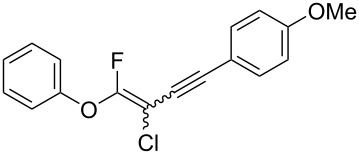 **3d** (1:1.4)	2.5	49
4^b^	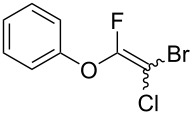 **1a** (1:1)	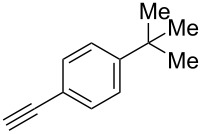 **5e**	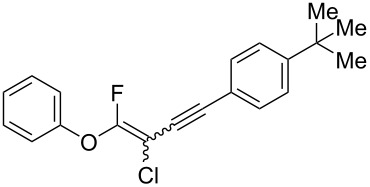 **3e** (1:1.4)	4.5	70
5	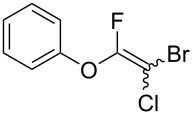 **1a** (1:1)	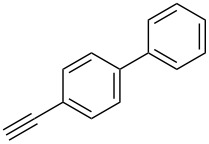 **5f**	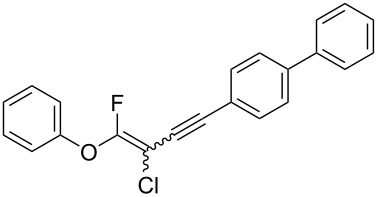 **3f** (1:1.1)	4.5	43
6	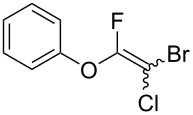 **1a** (1:1)	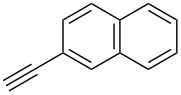 **5g**	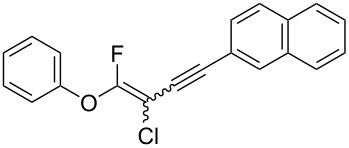 **3g** (1:1.2)	17.5	59
7^c^	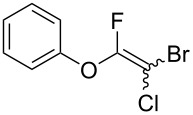 **1a** (1:1)	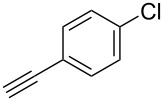 **5h**	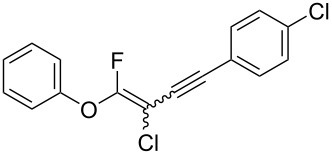 **3h** (1:1)	16	39
8	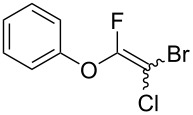 **1a** (1:1)	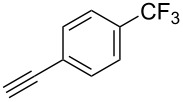 **5i**	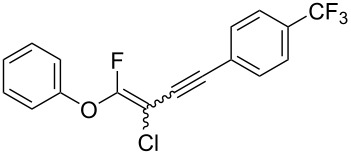 **3i** (1:1.2)	14	49
9	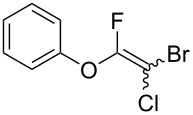 **1a** (1:1)	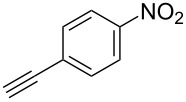 **5j**	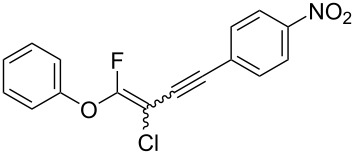 **3j** (1:1.3)	17	42
10	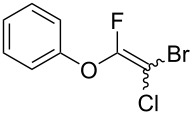 **1a** (1:1)	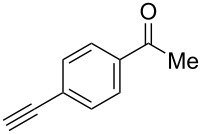 **5k**	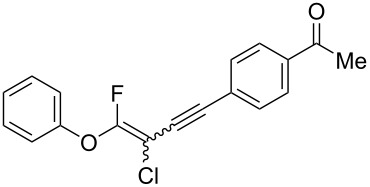 **3k** (1:1.1)	16.5	76
11	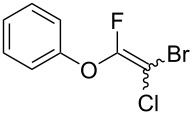 **1a** (1:1)	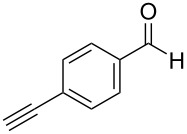 **5l**	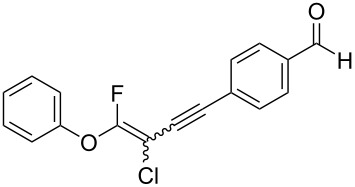 **3l** (1:1.1)	28.5	52
12	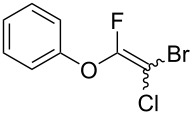 **1a** (1:1)	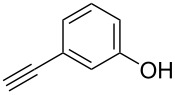 **5m**	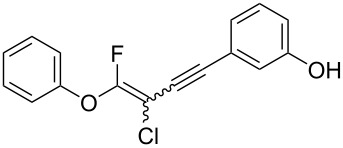 **3m** (1:1)	15.5	38
13	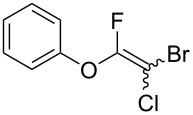 **1a** (1:1)	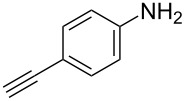 **5n**	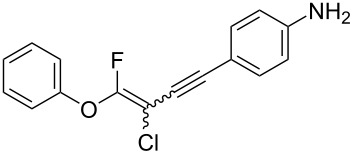 **3n** (1:1.1)	15.5	87
14^b^	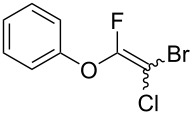 **1a** (1:1)	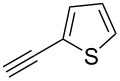 **5o**	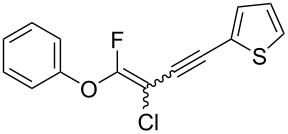 **3o** (1:1)	15	37
15^d^	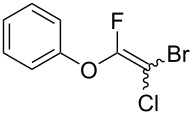 **1a** (1:1)	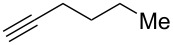 **5p**	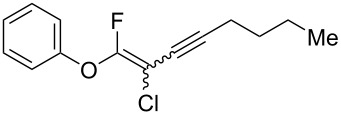 **3p** (1:1.1)	15	89
16	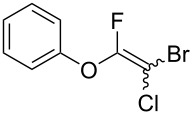 **1a** (1:1)	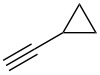 **5q**	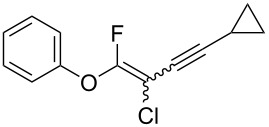 **3q** (1:1.4)	14.5	86
17	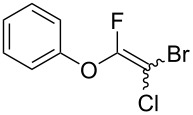 **1a** (1:1)	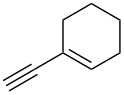 **5r**	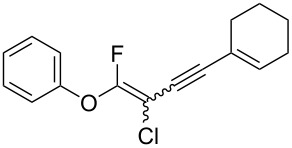 **3r** (1:1.4)	15	52
18	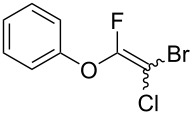 **1a** (1:1)	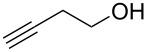 **5s**	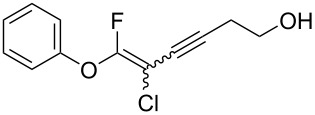 **3s** (1:1)	13.5	53
19	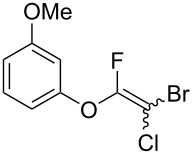 **1b** (1:1)	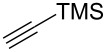 **5a**	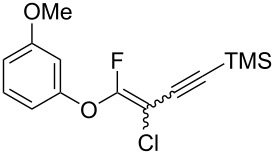 **3t** (1:1.1)	12.5	35
20^e^	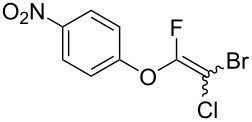 **1c** (1:1)	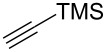 **5a**	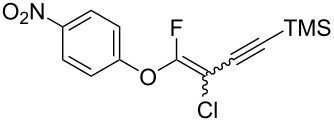 **3u** (1:1.2)	22	29
21	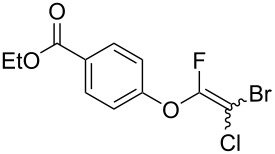 **1d** (1:1.1)	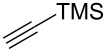 **5a**	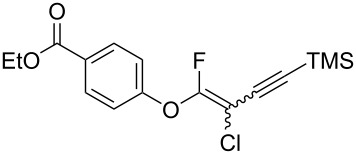 **3v** (1:1.6)	20.5	35
22^f^	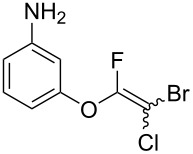 **1e** (1:1.1)	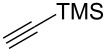 **5a**	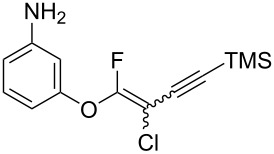 **3w** (1:1.3)	110	7

^a^Isolated yields; ^b^**1** (2.0 equiv) and **5** (1.0 equiv) were used; ^c^**1** (2.0 equiv) and **5** (1.0 equiv) were used; ^d^reaction temperature was 50 °C; ^e^**5** (1.3 equiv) was used; ^f^reaction temperature was rt to 50 °C.

Therefore, Suzuki–Miyaura and Sonogashira cross-coupling with **1** has a broad substrate scope and can be used to synthesize various fluoroalkenes **2** and fluoroenyne **3**. We speculate that these reaction mechanisms were similar to general cross-coupling mechanisms [45–46].

## Conclusion

We used Suzuki–Miyaura and Sonogashira cross-coupling to exploit the unique structure of multihalogenated fluorovinyl ethers **1** for the synthesis of many kinds of fluoroalkenes **2** and fluoroenynes **3** in moderate to high yields. The synthesized alkenes **2** still possess reactive chlorine atoms and phenoxy groups. Thus, new multisubstituted fluoroalkenes could be synthesized by applying other cross-couplings to **2**. In addition, enynes **3** could be converted into derivatives, such as fluorine-containing alkynylalcohols [47], allene compounds [48–50], and heterocycles [51–52]. However, further experiments are required to expand the abilities of **2** and **3** as new fluorine-containing building blocks.

## Experimental

### General information

^1^H NMR, ^19^F NMR, and ^13^C NMR spectra were recorded on JEOL ECZ 400S spectrometers. Chemical shifts of ^1^H NMR are reported in ppm from tetramethylsilane (TMS) as an internal standard. Chemical shifts of ^13^C NMR are reported in ppm from the center line of the triplet at 77.16 ppm for deuteriochloroform. Chemical shifts of ^19^F NMR are reported in ppm from CFCl_3_ as an internal standard. All data are reported as follows: chemical shifts, multiplicity (s = singlet, d = doublet, t = triplet, q = quartet, sep = septet, br = broad, brd = broad-doublet, m = multiplet), coupling constants (Hz), relative integration value. Mass spectra were obtained on a JEOL JMS-700T spectrometer (EI). Melting points were measured on a Yanaco MP-500V.

### Materials

All commercially available materials were used as received without further purification. All experiments were carried out under argon atmosphere in flame-dried glassware using standard inert techniques for introducing reagents and solvents unless otherwise noted.

### Suzuki–Miyaura cross-coupling with multihalogenated vinyl ethers **1**

To a solution of **1** (1.0 equiv), triphenylphosphine (10 mol %), cesium carbonate (1.5 equiv), palladium bis(trifluoroacetate) (5 mol %) in THF (2.0 mL) was added the respective boronic acid derivative **4** (2.0 equiv). The reaction solution was refluxed for 3.5 h. The reaction mixture was quenched by the addition of water (40 mL) at 0 °C and extracted with EtOAc. The organic phase was washed with brine (40 mL), dried over Na_2_SO_4_ and filtered. Then, the filtrate was concentrated under reduced pressure. The residue was purified by column chromatography and preparative TLC to afford **2**.

**2-Chloro-1-fluoro-2-phenylethenyl phenyl ether (2a):** Compound **2a** was purified by column chromatography and preparative TLC (hexane only), and obtained in 96% yield (122.0 mg) as a pale yellow oil; ^1^H NMR (400 MHz, CDCl_3_) δ 7.04–7.22 (m, 2H), 7.26–7.50 (m, 6H), 7.55–7.69 (m, 2H); ^13^C NMR (100 MHz, CDCl_3_) δ 101.4 (d, *J* = 30.9 Hz), 102.5 (d, *J* = 48.0 Hz), 116.5 (d, *J* = 3.8 Hz), 124.8, 127.3, 127.4, 127.9 (d, *J* = 3.2 Hz), 128.2 (d, *J* = 5.5 Hz), 128.5 (d, *J* = 7.2 Hz), 128.6 (d, *J* = 11.8 Hz), 128.9, 130.1 (d, *J* = 4.2 Hz), 132.46 (d, *J* = 5.7 Hz), 132.52, 141.3, 151.2 (d, *J* = 286.1 Hz), 151.5 (d, *J* = 287.3 Hz), 154.3 (d, *J* = 3.4 Hz), 154.4 (d, *J* = 3.3 Hz); ^19^F NMR (376 MHz, CDCl_3_) δ −80.8 (s) and −87.6 (s) (1F, 1:1.1); EIMS (*m*/*z*): 248, 250 [M]^+^; HREIMS [M]^+^ (*m*/*z*): calcd. for C_14_H_10_ClFO, 248.0402; found, 248.0404.

### Sonogashira cross-coupling with multihalogenated vinyl ethers **1**

To a solution of **1** (1.0 equiv), copper iodide (4 mol %), bis(triphenylphosphine)palladium dichloride (4 mol %) and triethylamine (1.5 equiv) in THF (2.5 mL) was added the respective alkyne **5** (2.0 equiv). The reaction solution was stirred at room temperature until **1** was disappeared. The reaction mixture was evaporated and concentrated under reduced pressure. The residue was purified by column chromatography and preparative TLC to afford **3**.

**(3-Chloro-4-fluoro-4-phenoxybut-3-en-1-yn-1-yl)trimethylsilane (3a):** Reaction time was 19 h. **3a** was purified by column chromatography (pentane only), and obtained in 80% yield (107.2 mg) as a yellow oil; ^1^H NMR (400 MHz, CDCl_3_) δ 0.13 (s) and 0.24 (s) (9H), 7.08 (d, *J* = 8.0 Hz, 2H), 7.07–7.15 (m, 2H), 7.16–7.23 (m, 1H) , 7.33–7.42 (m, 1H); ^13^C NMR (100 MHz, CDCl_3_) δ −0.36, −0.26, 85.2 (d, *J* = 44.8 Hz), 85.6 (d, *J* = 53.3 Hz), 94.5 (d, *J* = 47.8 Hz), 94.6 (d, *J* = 43.5 Hz), 103.7 (d, *J* = 63.4 Hz), 103.8 (d, *J* = 66.3 Hz), 117.2, 117.3, 125.2, 125.3, 130.0, 130.1, 154.00 (d, *J* = 75.1 Hz), 154.02 (d, *J* = 75.8 Hz), 158.2 (d, *J* = 292.4 Hz), 158.8 (d, *J* = 290.1 Hz); ^19^F NMR (376 MHz, CDCl_3_) δ −73.3 (s) and −78.4 (s) (1F, 1:1.2); EIMS *m*/*z*: 268 (M^+^); HREIMS [M]^+^ (*m*/*z*): calcd. for C_13_H_14_ClFOSi, 268.0486; found, 268.0490.

## Supporting Information

File 1 Characterization data for **2b–s** and **3b–w,** and copies of ^1^H, ^13^C, and ^19^F NMR spectra.

## Data Availability

All data that supports the findings of this study is available in the published article and/or the supporting information of this article.
