# Supplementary material for: Synthesis of fluoroalkenes and fluoroenynes via cross-coupling reactions using novel multihalogenated vinyl ethers
Source: Beilstein J Org Chem. 2024 Oct 24;20:2691–703. doi: 10.3762/bjoc.20.226 (PMC11514441; doi:10.3762/bjoc.20.226)
Supplement: File 1 — Characterization data for 2b–s and 3b–w, and copies of 1H, 13C, and 19F NMR spectra. [file Beilstein_J_Org_Chem-20-2691-s001.pdf]

BEILSTEIN JOURNAL OF ORGANIC CHEMISTRY

Supporting Information

for

Synthesis of fluoroalkenes and fluoroenynes via cross-coupling reactions using novel multihalogenated vinyl ethers

Yukiko Karuo, Keita Hirata, Atsushi Tarui, Kazuyuki Sato, Kentaro Kawai and Masaaki Omote

Beilstein J. Org. Chem. **2024,** *20,* 2691–2703. doi:10.3762/bjoc.20.226

Characterization data for 2b–s and 3b–w, and copies of ^{1}H, ^{13}C, and ^{19}F NMR spectra

License and Terms: This is a supporting information file under the terms of the Creative Commons Attribution License (https://creativecommons.org/licenses/by/4.0). Please note that the reuse, redistribution and reproduction in particular requires that the author(s) and source are credited and that individual graphics may be subject to special legal provisions.
The license is subject to the *Beilstein Journal of Organic Chemistry* terms and conditions: (https://www.beilstein-journals.org/bjoc/terms)

Table of content

Characterization data ……….…………………….…………….……………………..S2

^{1}H, ^{13}C and ^{19}F NMR charts……………………………………..……....S20–S81

Characterization data:

2-Chloro-1-fluoro-2-*p*-tolylethenyl phenyl ether (2b)

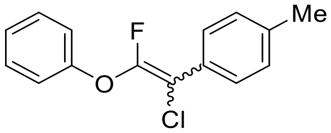

The title product (**2b**) was purified by column chromatography and preparative TLC (hexane only). **2b** was obtained in 98% yield (131.1 mg).

A pale yellow oil.; ^{1}H NMR (400 MHz, $CDCl_3$) δ: 2.31 (s) and 2.37 (s) (3H), 7.03-7.09 (2H, m), 7.10-7.17 (3H, m), 7.18-7.25 (1H, m), 7.30-7.40 (2H, m), 7.43-7.49 (2H, m), 7.52 (1H, d, J = 8.0 Hz); ^{13}C NMR (100 MHz, $CDCl_3$) δ: 21.3 21.4, 101.5 (d, J = 31.7 Hz), 102.6 (d, J = 48.2 Hz), 116.4, 116.5, 124.6 , 126.9, 127.8 (d, J = 3.1 Hz), 128.1 (d, J = 5.3 Hz), 129.1 (d, J = 5.9 Hz), 129.57, 129.64, 130.0 (d, J = 3.7 Hz), 138.6, 138.7, 151.1 (d, J = 286.0 Hz), 151.3 (d, J = 286.4 Hz), 154.4 (d, J = 3.6 Hz), 154.5 (d, J = 3.6 Hz); ^{19}F NMR (376 MHz, $CDCl_3$) δ: -81.7 (s) and -88.2 (s) (1F, 1:1); MS (EI) *m/z*: 262 (M^+); HRMS (EI) Calcd. for $C_{15}H_{12}ClFO$: 262.0561 (M^+), Found: 262.0562.

2-Chloro-1-fluoro-2-*m*-tolylethenyl phenyl ether (2c)

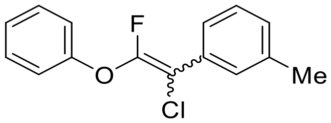

Multihalogenated vinyl ether (1.5 equiv) and *m*-tolylboronic acid (1.0 equiv) were used. Reaction time was 2.5 h.

The title product (**2c**) was purified by column chromatography and preparative TLC (hexane only). **2c** was obtained in 26% yield (33.9 mg).

A colorless oil.; ^{1}H NMR (400 MHz, $CDCl_3$) δ: 2.32 (s) and 2.40 (s) (3H), 7.04-7.11 (2H, m), 7.12-7.23 (3H, m), 7.27-7.48 (4H, m); ^{13}C NMR (100 MHz, $CDCl_3$) δ: 21.57, 21.60, 101.5 (d, J = 31.2 Hz), 102.6 (d, J = 48.1 Hz), 116.5, 124.7, 125.0 (d, J = 2.7 Hz), 125.3 (d, J = 5.3 Hz), 128.3, 128.4, 128.7 (d, J = 3.2 Hz), 128.8 (d, J = 4.9 Hz), 129.4, 129.5, 130.0, 130.1, 132.3, 132.4, 138.1, 138.2, 151.2 (d, J = 285.3 Hz), 151.4 (d, J = 286.6 Hz), 154.3 (d, J = 3.2 Hz), 154.5 (d, J = 3.0 Hz); ^{19}F NMR (376 MHz, $CDCl_3$) δ: -81.0 (s) and -87.7 (s) (1F, 1:1); MS (EI) *m/z*: 262 (M^+); HRMS (EI) Calcd. for $C_{15}H_{12}ClFO$: 262.0561 (M^+), Found: 262.0561.

2-Chloro-1-fluoro-2-*o*-tolylethenyl phenyl ether (2d)

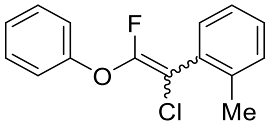

The title product (**2d**) was purified by column chromatography (hexane only). **2d** was obtained in 16% yield (52.6 mg).

A colorless oil.; ^{1}H NMR (400 MHz, $CDCl_3$) δ: 2.37 (s) and 2.44 (s) (3H), 6.33-6.99 (1H, m), 7.06-

7.16 (1H, m), 7.16-7.22 (2H, m), 7.22-7.33 (3.5H, m), 7.36-7.44 (1.5H, m); ^{13}C NMR (100 MHz, $CDCl_3$) δ: 19.7, 19.8, 99.3 (d, *J* = 31.8 Hz), 100.5 (d, *J* = 55.8 Hz), 116.5, 116.6, 124.6 (d, *J* = 18.4 Hz), 126.1 (d, *J* = 16.3 Hz), 129.6, 129.7, 129.9, 130.1, 130.3 (d, *J* = 3.4 Hz), 130.4, 130.5, 130.6, 132.1 (d, *J* = 2.8 Hz), 137.6 , 138.0 (d, *J* = 2.8 Hz), 151.5 (d, *J* = 280.8 Hz) , 151.6 (d, *J* = 287.0 Hz), 154.4 (d, *J* = 3.1 Hz) , 154.7 (d, *J* = 3.1 Hz); ^{19}F NMR (376 MHz, $CDCl_3$) δ: -85.4 (s) and -87.5 (s) (1F, 1:1); MS (EI) *m/z*: 262 (M^+); HRMS (EI) Calcd. for $C_{15}H_{12}ClFO$: 262.0561 (M^+), Found: 262.0560.

2-Chloro-1-fluoro-2-(3,4-methylenedioxyphenyl)ethenyl phenyl ether (2e)

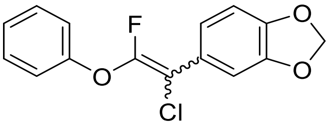

The title product (**2e**) was purified by column chromatography and preparative TLC (hexane only). **2e** was obtained in 85% yield (278.9 mg).

A pale yellow oil; ^{1}H NMR (400 MHz, $CDCl_3$) δ: 5.95 (s) and 6.00 (s) (2H), 6.75 (d, *J* = 8.0 Hz) and 6.84 (d, *J* = 8.0 Hz) (1H), 7.03-7.09 (2H, m), 7.10-7.21 (3H, m), 7.30-7.44 (2H, m); ^{13}C NMR (100 MHz, $CDCl_3$) δ: 101.3 (d, *J* = 32.8 Hz), 101.5, 101.6, 102.3 (d, *J* = 48.2 Hz), 108.2, 108.3, 108.6 (d, *J* = 2.9 Hz), 108.8 (d, *J* = 5.8 Hz), 116.4, 116.5, 122.4 (d, *J* = 3.4 Hz), 122.6 (d, *J* = 5.1 Hz), 124.7, 126.2, 126.25, 126.30, 130.0, 130.1, 147.8 (d, *J* = 11.1 Hz), 147.9 (d, *J* = 7.8 Hz) , 151.0 (d, *J* = 284.9 Hz), 151.1 (d, *J* = 285.4 Hz), 154.4; ^{19}F NMR (376 MHz, $CDCl_3$) δ: -81.9 (s) and -88.2 (s) (1F, 1:1); MS (EI) *m/z*: 292, 294 (M^+); HRMS (EI) Calcd. for $C_{15}H_{10}ClFO_3$: 292.0303, 294.0273 (M^+), Found: 292.0301, 294.0278.

2-Chloro-1-fluoro-2-*p*-fluorophenylethenyl phenyl ether (2f)

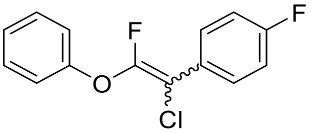

The title product (**2f**) was purified by column chromatography (hexane only). **2f** was obtained in 94% yield (128.6 mg).

A colorless oil; ^{1}H NMR (400 MHz, $CDCl_3$) δ: 6.97-7.23 (5H, m), 7.32-7.43 (2H, m), 7.51-7.66 (2H, m); ^{13}C NMR (100 MHz, $CDCl_3$) δ: 100.5 (d, *J* = 31.7 Hz), 101.5 (d, *J* = 47.9 Hz), 115.4 (d, *J* = 6.5 Hz), 115.6 (d, *J* = 6.6 Hz), 116.4, 116.5, 124.8, 124.9, 128.6 (m), 128.8, 129.9 (d, *J* = 3.0 Hz), 130.0 (d, *J* = 3.1 Hz), 130.1, 130.2, 151.3 (d, *J* = 287.7 Hz), 151.5 (d, *J* = 285.9 Hz), 152.2 (d, *J* = 286.9 Hz), 154.3, 161.3 (d, *J* = 3.0 Hz), 163.8 (d, *J* = 3.7 Hz); ^{19}F NMR (376 MHz, $CDCl_3$) δ: -81.1 (s) and -87.8 (s) (1F, 1:1), -112. 2 (m), -112.4 (m) and -115.6 (m) (1F); MS (EI) -*m/z*: 266 (M^+); HRMS (EI) Calcd. for $C_{14}H_9ClF_2O$: 266.0310 (M^+), Found: 266.0314.

2-*p*-Acetylphenyl-2-chloro-1-fluoroethenyl phenyl ether (2g)

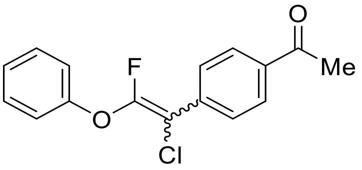

Reaction time was 2.5 h.

The title product (**2g**) was purified by column chromatography (pentane only to pentane:Et_2O = 4:1). **2g** was obtained in 76% yield (112.3 mg).

A red oil; ^{1}H NMR (400 MHz, $CDCl_3$) δ: 2.58 (s) and 2.63 (s) (3H), 7.04-7.12 (1H, m), 7.13-7.25 (2H, m), 7.32-7.46 (2H, m), 7.71 (1H, d, *J* = 8.5 Hz), 7.75 (1H, d, *J* = 8.5 Hz), 7.91 (1H, d, *J* = 8.5 Hz), 7.99 (1H, d, *J* = 8.5 Hz); ^{13}C NMR (100 MHz, $CDCl_3$) δ: 26.76, 26.81, 100.6 (d, *J* = 31.8 Hz), 101.3 (d, *J* = 46.7 Hz), 116.6, 116.8, 125.07, 125.11, 128.0 (d, *J* = 3.2 Hz), 128.2 (d, *J* = 6.2 Hz), 128.39, 128.44, 130.1, 130.2, 136.58, 136.62, 137.1 (d, *J* = 6.2 Hz), 137.2, 152.2 (d, *J* = 288.3 Hz), 152.4 (d, *J* = 290.0 Hz), 153.9, 154.0, 197.4, 197.5; ^{19}F NMR (376 MHz, $CDCl_3$) δ: -77.2 (s) and -84.3 (s) (1F, 1:1); MS (EI) *m/z*: 290 (M^+); HRMS (EI) Calcd. for $C_{16}H_{12}ClFO_2$: 290.0510 (M^+), Found: 290.0508.

2-Chloro-1-fluoro-2-(*p*-methoxycarbonylphenyl)ethenyl phenyl ether (2h)

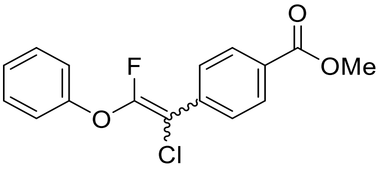

Reaction time was 1.5 h.

The title product (**2h**) was purified by column chromatography (hexane:AcOEt = 9:1). **2h** was obtained in 96% yield (149.9 mg).

A pale yellow oil; ^{1}H NMR (400 MHz, $CDCl_3$) δ: 3.90 (s) and 3.94 (s) (3H), 7.04-7.11 (1H, m), 7.13-7.24 (2H, m), 7.32-7.44 (2H, m), 7.68 (1H, d, *J* = 8.5 Hz), 7.73 (1H, d, *J* = 8.5 Hz), 7.99 (1H, d, *J* = 8.5 Hz), 8.06 (1H, d, *J* = 8.5 Hz); ^{13}C NMR (100 MHz, $CDCl_3$) δ: 52.3, 52.4, 100.6 (d, *J* = 32.1 Hz), 101.4 (d, *J* = 46.6 Hz), 116.6, 116.8, 125.0, 125.1, 127.8 (d, *J* = 3.3 Hz), 128.0 (d, *J* = 6.2 Hz), 129.7 (d, *J* = 5.5 Hz), 129.85, 129.89, 130.2 (d, *J* = 9.1 Hz), 136.91, 136.97, 137.0, 152.1 (d, *J* = 287.9 Hz), 152.2 (d, *J* = 289.7 Hz), 154.03, 154.06, 166.6; ^{19}F NMR (376 MHz, $CDCl_3$) δ: -77.5 (s) and -84.5 (s) (1F, 1:1); MS (EI) *m/z*: 306 (M^+); HRMS (EI) Calcd. for $C_{16}H_{12}ClFO_3$: 306.0459 (M^+), Found: 306.0459.

2-Chloro-1-fluoro-2-(*p*-formylphenyl)ethenyl phenyl ether (2i)

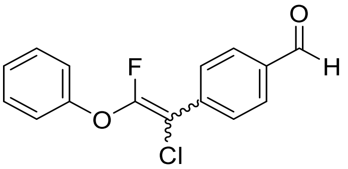

The title product (**2i**) was purified by column chromatography and preparative TLC (hexane:AcOEt = 9:1). **2i** was obtained in 77% yield (108.5 mg).

A pale yellow oil; ^{1}H NMR (400 MHz, $CDCl_3$) δ: 7.04-7.11 (1H, m), 7.12-7.23 (2H, m), 7.32-7.43 (2H, m), 7.74-7.87 (3H, m), 7.91 (1H, d, *J* = 8.4 Hz), 9.97 (s) and 10.0 (s) (1H); ^{13}C NMR (100 MHz, $CDCl_3$) δ: 100.3 (d, *J* = 32.3 Hz), 101.1 (d, *J* = 46.1 Hz), 116.6, 116.9, 125.16, 125.22, 128.4 (d, *J* = 3.3 Hz), 128.6 (d, *J* = 6.5 Hz), 129.7 (d, *J* = 4.5 Hz), 130.2 (d, *J* = 11.4 Hz), 135.78, 135.82, 138.41, 138.47, 138.5, 152.4 (d, *J* = 289.0 Hz), 152.6 (d, *J* = 290.7 Hz), 153.9, 191.5, 191.6; ^{19}F NMR (376 MHz, $CDCl_3$) δ: -76.3 (s) and -83.5 (s) (1F, 1:1); MS (EI) *m/z*: 276 (M^+); HRMS (EI) Calcd. for $C_{15}H_{10}ClFO_2$: 276.0353 (M^+), Found: 276.0352.

2-Chloro-1-fluoro-2-*m*-nitrophenylethenyl phenyl ether (2j)

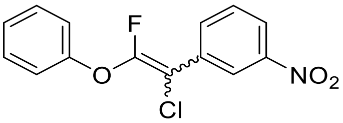

Reaction time was 3.0 h.

The title product (**2j**) was purified by column chromatography (hexane:AcOEt = 9:1). **2j** was obtained in 88% yield (131.8 mg).

A brown oil; ^{1}H NMR (400 MHz, $CDCl_3$) δ: 7.07-7.13 (1H, m), 7.14-7.26 (2H, m), 7.34-7.45 (2H, m), 7.52 (t, *J* = 8.1 Hz) and 7.60 (t, *J* = 8.1 Hz) (1H), 7.94 (ddd, *J* = 7.8, 1.8, 1.8 Hz) and 7.96-8.01 (m) (1H), 8.14 (ddd, *J* = 8.2, 2.2, 0.9 Hz) and 8.20 (ddd, *J* = 8.2, 2.2, 2.2 Hz) (1H), 3.53 (1H, br s); ^{13}C NMR (100 MHz, $CDCl_3$) δ: 99.2 (d, *J* = 33.4 Hz), 99.9 (d, *J* = 46.5 Hz), 116.6, 116.9, 122.97, 123.00, 123.06, 123.1, 123.2, 125.28, 125.34, 129.5, 129.6, 130.2, 130.3, 133.5 (d, *J* = 3.1 Hz), 133.7 (d, *J* = 6.4 Hz), 134.38, 134.45, 148.34, 148.39, 152.5 (d, *J* = 287.5 Hz), 152.6 (d, *J* = 289.9 Hz), 153.8 (d, *J* = 2.5 Hz), 153.9 (d, *J* = 2.4 Hz); ^{19}F NMR (376 MHz, $CDCl_3$) δ: -76.5 (s) and -84.0 (s) (1F, 1:1.1); MS (EI) *m/z*: 293 (M^+); HRMS (EI) Calcd. for $C_{14}H_9ClFNO_3$: 293.0255 (M^+), Found: 293.0255.

2-Chloro-1-fluoro-2-*p*-hydroxyphenylethenyl phenyl ether (2k)

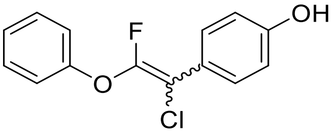

Reaction time was 6.5 h.

The title product (**2k**) was purified by column chromatography (hexane:AcOEt = 3:1) and preparative TLC (hexane:AcOEt = 7:3). **2k** was obtained in 9% yield (12.3 mg).

A yellow oil; ^{1}H NMR (400 MHz, $CDCl_3$) δ: 4.90 (s) and 4.97 (s) (1H), 6.76 (d, J = 8.6 Hz) and 6.86 (d, J = 8.7 Hz) (1H), 7.06 (1H, d, J = 8.0 Hz), 7.10-7.21 (2H, m), 7.34 (t, J = 7.7 Hz) and 7.38 (t, J = 7.8 Hz) (2H), 7.45 (d, J = 8.6 Hz) and 7.52 (d, J = 8.4 Hz) (2H); ^{13}C NMR (100 MHz, $CDCl_3$) δ: 101.3 (d, J = 32.1 Hz), 102.4 (d, J = 48.4 Hz), 115.3, 115.4, 116.35, 116.39, 124.6, 125.0 (d, J = 5.6 Hz), 125.1, 129.6 (d, J = 3.2 Hz), 129.8 (d, J = 5.3 Hz), 130.0, 130.1, 150.8 (d, J = 284.7 Hz), 151.0 (d, J = 285.1 Hz), 154.4 (d, J = 3.3 Hz), 154.5 (d, J = 3.3 Hz), 155.6, 155.7; ^{19}F NMR (376 MHz, $CDCl_3$) δ: -82.6 (s) and -89.03 (s) (1F, 1.4:1); MS (EI) *m/z*: 264 (M^+); HRMS (EI) Calcd. for $C_{14}H_{10}ClFO_2$: 264.0353 (M^+), Found: 264.0354.

2-*m*-Aminophenyl-2-chloro-1-fluoroethenyl phenyl ether (2l)

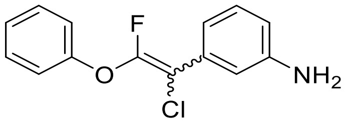

Reaction time was 4.0 h.

The title product (**2l**) was purified by column chromatography (hexane:AcOEt = 4:1). **2l** was obtained in 92% yield (123.5 mg).

A yellow oil; ^{1}H NMR (400 MHz, $CDCl_3$) δ: 3.72 (2H, br s), 6.57-6.70 (1H, m), 6.88-7.25 (6H, m), 7.31-7.58 (2H, m); ^{13}C NMR (100 MHz, $CDCl_3$) δ: 101.6 (d, J = 31.4 Hz), 102.7 (d, J = 47.7 Hz), 114.6 (d, J = 3.5 Hz), 114.7 (d, J = 5.1 Hz), 115.4, 115.5, 116.46, 116.5, 118.3 (d, J = 2.8 Hz), 118.6 (d, J = 5.9 Hz), 124.66, 124.69, 129.3, 129.4, 130.0, 130.1, 133.38, 133.44, 133.5, 146.4, 146.5, 151.2 (d, J = 285.3 Hz), 151.4 (d, J = 286.6 Hz), 154.4 (d, J = 3.4 Hz), 154.5 (d, J = 3.3 Hz); ^{19}F NMR (376 MHz, $CDCl_3$) δ: -80.7 (s) and -87.0 (s) (1F, 1:1); MS (EI) *m/z*: 263 (M^+); HRMS (EI) Calcd. for $C_{14}H_{11}ClFNO$: 263.0513 (M^+), Found: 263.0512.

2-Chloro-2-cyclopropyl-1-fluoroethenyl phenyl ether (2n)

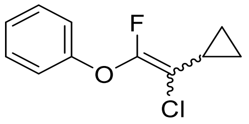

Reaction time was 5.0 h.

The title product (**2n**) was purified by column chromatography (hexane:AcOEt = 9:1). **2n** was obtained in 71% yield (77.4 mg).

A yellow oil; ^{1}H NMR (400 MHz, $CDCl_3$) δ: 0.65-0.84 (4H, m), 1.67-1.77 (m) and 1.84-1.95 (m) (1H), 7.08 (2H, d, J = 8.0 Hz), 7.10-7.18 (1H, m), 7.30-7.40 (2H, m); ^{13}C NMR (100 MHz, $CDCl_3$) δ: 4.77, 4.98, 10.7, 10.9, 104.0 (d, J = 28.8 Hz), 105.3 (d, J = 52.1 Hz), 116.0, 116.2, 124.3, 129.9, 130.0, 150.8 (d, J = 280.1 Hz), 151.7 (d, J = 278.5 Hz), 155.1 (d, J = 3.9 Hz), 154.87 (d, J = 3.9 Hz); ^{19}F NMR (376 MHz, $CDCl_3$) δ: -85.6 (s) and -96.0 (s) (1F, 1:1); MS (EI) *m/z*: 212 (M^+); HRMS (EI) Calcd. for $C_{11}H_{10}ClFO_3$: 212.0404 (M^+), Found: 212.0404.

2-Chloro-1-fluoro-2-(thiophene-2-yl)ethenyl phenyl ether (2o)

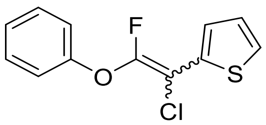

Reaction time was 4 h.

The title product (**2o**) was purified by column chromatography and preparative TLC (hexane only). **2o** was obtained in 31% yield (38.5 mg).

A colorless oil; ^{1}H NMR (400 MHz, $CDCl_3$) δ: 6.95-7.03 (m) and 7.06 (td, J = 4.4, 1.3 Hz) (1H), 7.13 (2H, d, J = 8.1 Hz), 7.15-7.21 (1H, m), 7.23-7.27 (m) and 7.31 (dd, J = 3.7, 1.1 Hz) (1H), 7.33-7.40 (3H, m); ^{13}C NMR (100 MHz, $CDCl_3$) δ: 98.1 (d, J = 37.2 Hz), 98.8 (d, J = 51.0 Hz), 116.5, 116.6, 124.2 (d, J = 60.1 Hz), 124.9, 125.1, 126.8 (d, J = 5.5 Hz), 126.8, 127.0, 127.1, 127.23 (d, J = 15.4 Hz), 130.09, 130.15, 134.5, 134.9 (d, J = 9.4 Hz), 137.5, 150.4 (d, J = 287.3 Hz), 150.8 (d, J = 288.6 Hz), 153.7 (d, J = 3.6 Hz), 154.4 (d, J = 3.9 Hz); ^{19}F NMR (376 MHz, $CDCl_3$) δ: -82.1 (s) and -84.5 (s) (1F, 1.1:1); MS (EI) *m/z*: 254 (M^+); HRMS (EI) Calcd. for $C_{12}H_8ClFOS$: 253.9968 (M^+), Found: 253.9972.

2-Chloro-1-fluoro-2-phenylethenyl 3-methoxyphenyl ether (2p)

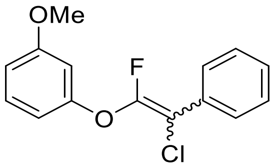

Reaction time was 3.5 h.

The title product (**2p**) was purified by column chromatography (hexane to hexane:AcOEt = 9:1). **2p** was obtained in 62% yield (92.3 mg).

A yellow oil; ^{1}H NMR (400 MHz, $CDCl_3$) δ: 3.78 (s) and 3.81(s) (3H), 6.57-6.79 (3H, m), 7.19-7.37 (3H, m), 7.41 (1H, t, J = 7.6 Hz), 7.57 (1H, t, J = 7.6 Hz), 7.64 (1H, t, J = 7.6 Hz); ^{13}C NMR (100 MHz, $CDCl_3$) δ: 55.6, 101.6 (d, J = 31.7 Hz), 102.7 (d, J = 50.5 Hz), 102.92, 102.93, 108.4, 110.3, 127.9 (d, J = 3.2 Hz), 128.17, 128.22, 128.4, 128.5 128.6 (d, J = 12.1 Hz), 130.48, 130.54, 132.45 (d, J = 5.6 Hz), 132.54, 151.2 (d, J = 285.6 Hz), 151.3 (d, J = 286.9 Hz), 155.4 (d, J = 3.5 Hz), 155.5 (d, J = 3.4 Hz), 161.1; ^{19}F NMR (376 MHz, $CDCl_3$) δ: -80.7 (s) and -87.5 (s) (1F, 1:1); MS (EI) *m/z*: 278 (M^+); HRMS (EI) Calcd. for $C_{15}H_{12}ClFO_2$: 278.0510 (M^+), Found: 278.0510.

2-Chloro-1-fluoro-2-phenylethenyl 4-nitrophenyl ether (2q)

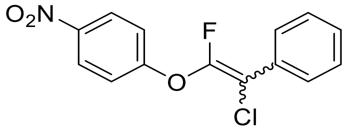

Reaction time was 5.5 h.

The title product (**2q**) was purified by column chromatography (hexane to hexane:AcOEt = 9:1). **2p**

was obtained in 85% yield (128.0 mg).

A yellow oil; ^{1}H NMR (400 MHz, $CDCl_3$) δ: 7.18 (1H, dd, *J* = 9.1, 1.1 Hz), 7.23-7.35 (3H, m), 7.38-7.53 (2H, m), 7.65 (1H, dt, *J* = 8.0, 1.3 Hz), 8.24 (1H, d, *J* = 9.2 Hz), 8.30 (1H, d, *J* = 9.2 Hz); ^{13}C NMR (100 MHz, $CDCl_3$) δ: 103.3 (d, *J* = 29.6 Hz), 104.5 (d, *J* = 45.0 Hz), 116.6, 116.7, 126.3, 128.0 (d, *J* = 3.1 Hz), 128.2 (d, *J* = 5.4 Hz), 128.6, 128.7, 129.2, 129.3, 131.6 (d, *J* = 5.8 Hz), 131.8, 144.5, 150.2 (d, *J* = 285.8 Hz), 150.3 (d, *J* = 287.5 Hz), 158.76 (d, *J* = 4.3 Hz), 158.81(d, *J* = 3.8 Hz); ^{19}F NMR (376 MHz, $CDCl_3$) δ: -82.9 (s) and -88.9 (s) (1F, 1:1); MS (EI) *m/z*: 293 (M^+); HRMS (EI) Calcd. for $C_{14}H_9ClFNO_3$: 293.0255 (M^+), Found: 293.0258.

2-Chloro-1-fluoro-2-phenylethenyl 4-ethoxycarbonylphenyl ether (2r)

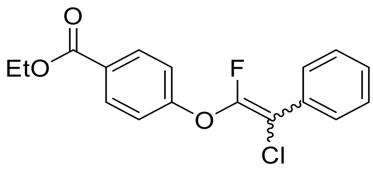

Reaction time was 3.5 h.

The title product (**2r**) was purified by column chromatography (hexane:AcOEt = 14:1) and preparative TLC (hexane:AcOEt = 39:1). **2r** was obtained in 45% yield (58.9 mg).

A pale yellow oil; ^{1}H NMR (400 MHz, $CDCl_3$) δ: 1.38 (t, *J* = 6.9 Hz) and 1.40 (t, *J* = 7.1 Hz) (3H), 4.36 (d, *J* = 6.9 Hz) and 4.38 (d, *J* = 7.1 Hz) (2H), 7.18 (1H, dd, *J* = 8.8, 1.4 Hz), 7.18 (1H, dd, *J* = 9.1, 0.9 Hz), 7.27-7.48 (3H, m), 7.52 (1H, dd, *J* = 8.2, 1.3 Hz), 7.65 (1H, dt, *J* = 8.3, 1.2 Hz), 8.04 (1H, d, *J* = 8.9 Hz), 8.09 (1H, d, *J* = 8.9 Hz); ^{13}C NMR (100 MHz, $CDCl_3$) δ: 14.5, 61.2, 102.4 (d, *J* = 30.4 Hz), 103.5 (d, *J* = 46.6 Hz), 115.98, 116.02, 127.02, 127.07, 128.0 (d, *J* = 3.0 Hz), 128.2 (d, *J* = 5.5 Hz), 128.5, 128.6, 128.8, 129.0, 131.97, 132.1 (d, *J* = 5.8 Hz), 132.2, 150.7 (d, *J* = 286.0 Hz), 150.8 (d, *J* = 286.9 Hz), 158.7 (d, *J* = 3.8 Hz), 157.8 (d, *J* = 3.7 Hz), 165.7, 165.8; ^{19}F NMR (376 MHz, $CDCl_3$) δ: -81.7 (s) and -88.1 (s) (1F, 1:1.6); MS (EI) *m/z*: 320 (M^+); HRMS (EI) Calcd. for $C_{17}H_{14}ClFO_3$: 320.0616 (M^+), Found: 320.0617.

3-Aminophenyl 2-chloro-1-fluoro-2-phenylethenyl ether (2s)

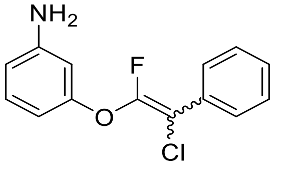

DME was used as a solvent. Reaction time was 21.5 h.

The title product (**2s**) was purified by column chromatography and preparative TLC (hexane:AcOEt = 4:1) and preparative TLC (hexane:AcOEt = 9:1). **2s** was obtained in 15% yield (20.0 mg).

A yellow oil; ^{1}H NMR (400 MHz, $CDCl_3$) δ: 3.77 (2H, br s), 6.35-6.59 (3H, m), 7.10 (t, *J* = 8.2 Hz) and 7.13 (t, *J* = 8.0 Hz) (1H), 7.26-7.36 (2H, m), 7.38-7.45 (1H, m), 7.55-7.67 (2H, m); ^{13}C NMR (100 MHz, $CDCl_3$) δ: 101.3 (d, *J* = 31.6 Hz), 102.4 (d, *J* = 47.9 Hz), 103.0, 103.1, 106.1, 106.2, 111.42,

111.45, 127.9 (d, J = 2.8 Hz), 128.2 (d, J = 5.5 Hz), 128.4 (d, J = 6.9 Hz), 128.5 (d, J = 13.1 Hz), 130.6, 130.7, 132.5, 132.6, 148.3, 151.2 (d, J = 285.1 Hz), 151.5 (d, J = 287.2 Hz), 155.4 (d, J = 3.2 Hz), 155.5 (d, J = 3.2 Hz); ^{19}F NMR (376 MHz, $CDCl_3$) δ: -80.1 (s) and -87.0 (s) (1F, 1:1.2); MS (EI) *m/z*: 263 (M^+); HRMS (EI) Calcd. for $C_{14}H_{11}ClFNO$: 263.0513 (M^+), Found: 263.0519.

2-Chloro-1-fluoro-1-phenoxy-4-phenylbut-1-en-3-yne (3b)

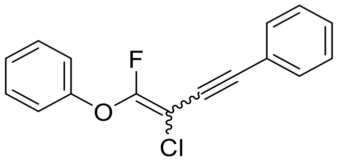

Reaction time was 18.5 h.

The title product (**3b**) was purified by column chromatography (pentane only). **3b** was obtained in 92% yield (125.4 mg).

A yellow oil; ^{1}H NMR (400 MHz, $CDCl_3$) δ: 7.10-7.24 (2H, m), 7.26-7.42 (6H, m) , 7.33-7.42 (1H, m); ^{13}C NMR (100 MHz, $CDCl_3$) δ: 79.7 (d, J = 7.1 Hz), 80.1 (d, J = 3.1 Hz), 85.4 (d, J = 39.0 Hz), 85.9 (d, J = 46.3 Hz), 96.4 (d, J = 6.3 Hz), 97.2 (d, J = 8.4 Hz), 117.1, 117.2, 121.9, 122.0, 125.26, 125.29, 128.5 (d, J = 10.6 Hz), 128.6, 129.2 (d, J = 11.3 Hz), 129.4, 130.1 (d, J = 3.4 Hz), 131.7 (d, J = 5.0 Hz), 132.6, 153.8 (d, J = 2.6 Hz), 154.4 (d, J = 2.8 Hz), 157.3 (d, J = 292.2 Hz), 157.6 (d, J = 291.2 Hz); ^{19}F NMR (376 MHz, $CDCl_3$) δ: -74.7 (s) and -78.9 (s) (1F, 1.1:1); MS (EI) *m/z*: 272 (M^+); HRMS (EI) Calcd. for $C_{16}H_{10}ClFO$: 272.0404 (M^+), Found: 272.0407.

2-Chloro-1-fluoro-4-(4-methylphenyl)-1-phenoxybut-1-en-3-yne (3c)

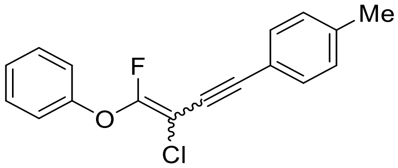

Multihalogenated vinyl ether (2.0 equiv) and 4-methylphenylacetylene (1.0 equiv) were used. Reaction time was 3.5 h.

The title product (**3c**) was purified by column chromatography (hexane only). **3c** was obtained in 74% yield (105.5 mg).

A yellow solid; ^{1}H NMR (400 MHz, $CDCl_3$) δ: 2.33 (s) and 2.37 (s) (3H), 7.06-7.26 (6H, m), 7.33-7.46 (3H, m); ^{13}C NMR (100 MHz, $CDCl_3$) δ: 21.69, 21.72, 79.1 (d, J = 7.3 Hz), 79.5 (d, J = 2.9 Hz), 85.87, 85.93 (d, J = 98.7 Hz), 96.7 (d, J = 6.3 Hz), 97.5 (d, J = 8.2 Hz), 117.0, 117.2, 118.8, 118.9, 125.19, 125.28, 129.2, 129.3, 130.05, 130.10, 131.56, 131.62, 132.5, 139.5, 139.6, 153.8 (d, J = 2.9 Hz), 154.4 (d, J = 3.0 Hz), 157.1 (d, J = 289.9 Hz), 157.4 (d, J = 290.4 Hz); ^{19}F NMR (376 MHz, $CDCl_3$) δ: -75.2 (s) and -79.3 (s) (1F, 1:1.4); MS (EI) *m/z*: 286 (M^+); HRMS (EI) Calcd. for $C_{17}H_{12}ClFO$: 286.0561 (M^+), Found: 286.0561; Mp:153.5-154.5.

2-Chloro-1-fluoro-4-(4-methoxyphenyl)-1-phenoxybut-1-en-3-yne (3d)

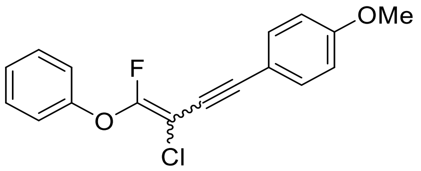

Multihalogenated vinyl ether (2.0 equiv) and 4-methoxyphenylacetylene (1.0 equiv) were used. Reaction time was 2.5 h.

The title product (**3d**) was purified by column chromatography (hexane:AcOEt =19:1) and preparative TLC (hexane:AcOEt = 9:1). **3d** was obtained in 49% yield (76.7 mg).

A yellow oil; ^{1}H NMR (400 MHz, $CDCl_3$) δ: 3.80 (s) and 3.83 (s) (3H), 6.81 (d, J = 8.7 Hz) and 6.88 (d, J = 8.7 Hz) (2H), 7.10-7.24 (3H, m), 7.29 (1H, d, J = 8.7 Hz), 7.35-7.42 (2H, m), 7.44 (1H, d, J = 8.7 Hz); ^{13}C NMR (100 MHz, $CDCl_3$) δ: 55.43, 55.46, 78.5 (d, J = 7.3 Hz), 78.9 (d, J = 2.5 Hz), 85.8 (d, J = 46.0 Hz), 86.3 (d, J = 53.6 Hz), 96.6 (d, J = 6.3 Hz), 97.4 (d, J = 8.4 Hz), 114.01, 114.04, 114.1, 114.2, 117.0, 117.2, 125.16, 125.20, 130.0, 130.1, 133.29, 133.34, 153.9 (d, J = 2.8 Hz), 154.5 (d, J = 3.2 Hz), 156.9 (d, J = 290.9 Hz), 157.3 (d, J = 290.3 Hz), 160.3, 160.4; ^{19}F NMR (376 MHz, $CDCl_3$) δ: -75.6 (s) and -79.6 (s) (1F, 1.4:1); MS (EI) *m*/*z*: 302 (M^+); HRMS (EI) Calcd. for $C_{17}H_{12}ClFO_2$: 302.0510 (M^+), Found: 302.0500.

4-(4-*tert*-Butylphenyl)-2-chloro-1-fluoro-1-phenoxybut-1-en-3-yne (3e)

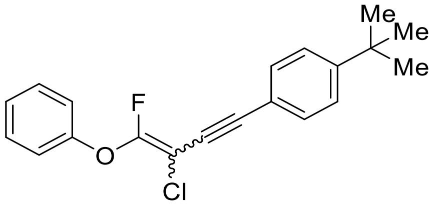

Multihalogenated vinyl ether (2.0 equiv) and 4-*tert*-butylphenylacetylene (1.0 equiv) were used. Reaction time was 4.5 h.

The title product (**3e**) was purified by column chromatography and preparative TLC (hexane only). **3e** was obtained in 70% yield (115.6 mg).

A pale yellow solid; ^{1}H NMR (400 MHz, $CDCl_3$) δ: 1.28 (s) and 1.31 (s) (3H), 7.10-7.22 (3H, m), 7.25-7.50 (6H, m); ^{13}C NMR (100 MHz, $CDCl_3$) δ: 31.23, 31.25, 34.97, 35.01, 79.1 (d, J = 7.2 Hz), 79.5 (d, J = 2.8 Hz), 85.7 (d, J = 37.2 Hz), 86.2 (d, J = 44.8 Hz), 96.7 (d, J = 6.4 Hz), 97.5 (d, J = 8.4 Hz), 117.05, 117.15, 118.9, 119.0, 125.2 (d, J = 4.6 Hz), 125.5, 125.57, 125.59 (d, J = 4.1 Hz), 130.06, 130.10, 131.44, 131.49, 132.4, 152.6, 152.7, 153.8 (d, J = 2.2 Hz), 154.4 (d, J = 3.2 Hz), 157.1 (d, J = 291.6 Hz), 157.5 (d, J = 290.6 Hz); ^{19}F NMR (376 MHz, $CDCl_3$) δ: -75.0 (s) and -79.3 (s) (1F, 1:1.4); MS (EI) *m*/*z*: 328 (M^+); HRMS (EI) Calcd. for $C_{20}H_{18}ClFO$: 328.1030 (M^+), Found: 328.1031; Mp: 192.2-193.3.

2-Chloro-1-fluoro-1-phenoxy-4-(4-phenylphenyl)but-1-en-3-yne (3f)

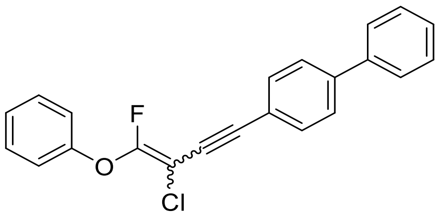

Reaction time was 4.5 h.

The title product (**3f**) was purified by column chromatography (hexane:AcOEt = 9:1) and preparative TLC (hexane:AcOEt = 14:1). **3f** was obtained in 43% yield (74.6 mg).

An orange solid; ^{1}H NMR (400 MHz, $CDCl_3$) δ: 7.10-7.23 (3H, m), 7.32-7.49 (6H, m), 7.50-7.65 (5H, m); ^{13}C NMR (100 MHz, $CDCl_3$) δ: 80.4 (d, *J* = 7.3 Hz), 80.8 (d, *J* = 3.1 Hz), 85.5 (d, *J* = 39.5 Hz), 86.0 (d, *J* = 46.6 Hz), 96.4 (d, *J* = 6.4 Hz), 97.1 (d, *J* = 8.1 Hz), 117.1, 117.2, 120.79, 120.85, 125.28, 125.30, 127.1, 127.16, 127.19, 128.0, 129.0, 130.11, 130.12, 132.08, 132.14, 140.2, 141.9, 142.0, 153.8 (d, *J* = 2.4 Hz), 154.4 (d, *J* = 2.7 Hz), 157.2 (d, *J* = 291.7 Hz), 157.6 (d, *J* = 291.1 Hz); ^{19}F NMR (376 MHz, $CDCl_3$) δ: -74.6 (s) and -78.7 (s) (1F, 1:1.1); MS (EI) *m*/*z*: 348 (M^+); HRMS (EI) Calcd. for $C_{22}H_{14}ClFO$: 348.0717 (M^+), Found: 348.0717; Mp: 61.2-61.8.

2-Chloro-1-fluoro-4-(2-naphthyl)-1-phenoxybut-1-en-3-yne (3g)

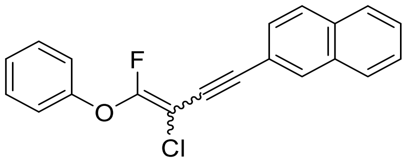

Reaction time was 17.5 h.

The title product (**3g**) was purified by column chromatography (hexane:AcOEt = 4:1) and preparative TLC (hexane only). **3g** was obtained in 59% yield (95.0 mg).

A yellow oil; ^{1}H NMR (400 MHz, $CDCl_3$) δ: 7.10-7.23 (3H, m), 7.32-7.41 (m) and 7.42-7.54 (m) (5H), 7.66-7.82 (3H, m), 7.85 (s) and 8.01 (s) (1H); ^{13}C NMR (100 MHz, $CDCl_3$) δ: 80.0 (d, *J* = 7.3 Hz), 80.4 (d, *J* = 3.5 Hz), 85.3, 85.7, 86.2, 96.9 (d, *J* = 6.2 Hz), 97.6 (d, *J* = 8.5 Hz), 117.0, 117.2, 119.16, 119.23, 125.3, 126.8 (d, *J* = 5.2 Hz), 127.2 (d, *J* = 5.5 Hz), 127.89, 127.93, 127.97, 128.1, 128.2, 128.3, 130.1, 131.87, 131.93, 132.9 (d, *J* = 8.6 Hz), 133.2 (d, *J* = 8.5 Hz), 153.8 (d, *J* = 2.0 Hz), 154.4 (d, *J* = 2.7 Hz), 157.3 (d, *J* = 292.4 Hz), 157.7 (d, *J* = 290.8 Hz); ^{19}F NMR (376 MHz, $CDCl_3$) δ: -74.4 (s) and -78.6 (s) (1F, 1.2:1); MS (EI) *m*/*z*: 322 (M^+); HRMS (EI) Calcd. for $C_{20}H_{12}ClFO$: 322.0561 (M^+), Found: 322.0561.

2-Chloro-4-(4-chlorophenyl)-1-fluoro-1-phenoxybut-1-en-3-yne (3h)

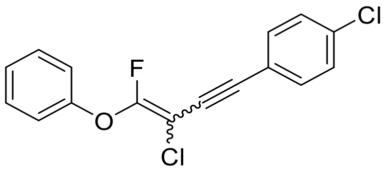

Multihalogenated vinyl ether (1.5 equiv) and 4-methylphenylacetylene (1.0 equiv) was used. Reaction time was 16 h.

The title product (**3h**) was purified by column chromatography and preparative TLC (hexane only). **3h** was obtained in 39% yield (59.9 mg).

A yellow oil; ^{1}H NMR (400 MHz, $CDCl_3$) δ: 7.11-7.17 (2H, m), 7.18-7.25 (1H, m), 7.27 (2H, s), 7.33 (1H, d, *J* = 8.5 Hz), 7.36-7.41 (2H, m), 7.43 (1H, d, *J* =8.5 Hz); ^{13}C NMR (100 MHz, $CDCl_3$) δ: 80.7 (d, *J* = 7.6 Hz), 81.1 (d, *J* = 3.6 Hz), 84.9 (d, *J* = 7.1 Hz), 85.3 (d, *J* = 7.9 Hz), 95.3 (d, *J* = 6.3 Hz), 95.9 (d, *J* = 8.0 Hz), 117.17, 117.22, 120.4, 120.5, 125.35, 125.40, 128.8, 129.0, 130.12, 130.14, 132.86, 132.90, 135.3, 135.4, 153.7 (d, *J* = 2.3 Hz), 154.3 (d, *J* = 2.9 Hz), 157.4 (d, *J* = 293.1 Hz), 157.8 (d, *J* = 291.4 Hz); ^{19}F NMR (376 MHz, $CDCl_3$) δ: -74.1 (s) and -78.3 (s) (1F, 1:1); MS (EI) *m*/*z*: 306 (M^+); HRMS (EI) Calcd. for $C_{16}H_9Cl_2FO$: 306.0014 (M^+), Found: 306.0015.

2-Chloro-1-fluoro-1-phenoxy-4-(4-trifluoromethylphenyl)but-1-en-3-yne (3i)

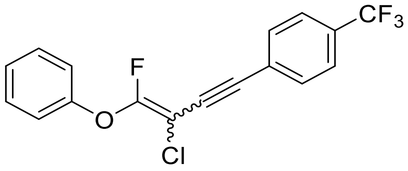

Reaction time was 14 h.

The title product (**3i**) was purified by column chromatography and preparative TLC (hexane only). **3i** was obtained in 49% yield (82.8 mg).

A colorless oil; ^{1}H NMR (400 MHz, $CDCl_3$) δ: 7.11-7.18 (2H, m), 7.19-7.25 (1H, m), 7.40 (1H, t, *J* = 7.9 Hz), 7.44 (1H, d, *J* = 8.3 Hz), 7.55 (1H, d, *J* = 8.1 Hz), 7.61 (2H, s); ^{13}C NMR (100 MHz, $CDCl_3$) δ: 82.1 (d, *J* = 7.3 Hz), 82.5 (d, *J* = 3.3 Hz), 84.6 (d, *J* = 31.6 Hz), 85.1 (d, *J* = 38.5 Hz), 94.8 (d, *J* = 6.0 Hz), 95.5 (d, *J* = 8.3 Hz), 117.3, 123.9 (q, *J* = 272.2 Hz), 124.0 (q, *J* = 272.2 Hz), 125.38, 125.42, 125.46, 125.51, 125.7, 125.8, 130.2, 130.6 (d, *J* = 9.4 Hz), 130.9 (d, *J* = 9.7 Hz), 131.8, 131.9, 153.6 (d, *J* = 1.9 Hz), 154.2 (d, *J* = 2.4 Hz), 157.8 (d, *J* = 294.2 Hz), 158.1 (d, *J* = 292.2 Hz); ^{19}F NMR (376 MHz, $CDCl_3$) δ: -62.79 (s) and -62.82 (s) (3F), -73.0 (s) and -77.4 (s) (1F, 1:1.2); MS (EI) *m*/*z*: 340 (M^+); HRMS (EI) Calcd. for $C_{17}H_9ClF_4O$: 340.0278 (M^+), Found: 340.0279.

2-Chloro-1-fluoro-4-(4-nitrophenyl)-1-phenoxybut-1-en-3-yne (3j)

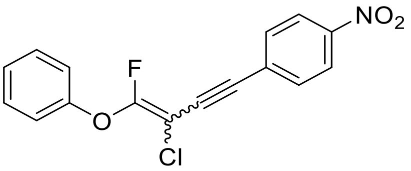

Reaction time was 17 h.

The title product (**3j**) was purified by column chromatography (hexane:AcOEt = 49:1 to19:1). **3j** was obtained in 42% yield (66.9 mg).

A yellow solid; ^{1}H NMR (400 MHz, $CDCl_3$) δ: 7.15 (2H, t, *J* = 6.7 Hz), 7.24 (2H, t, *J* = 7.3 Hz), 7.41 (2H, t, *J* = 7.7 Hz), 7.47 (1H, d, *J* = 8.7 Hz), 7.64 (1H, d, *J* = 8.7 Hz), 8.16 (1H, d, *J* = 8.7 Hz), 8.22 (1H, d, *J* = 8.7 Hz); ^{13}C NMR (100 MHz, $CDCl_3$) δ: 84.1 (d, *J* = 23.4 Hz), 84.6 (d, *J* = 30.1 Hz), 84.9 (d, *J* = 7.4 Hz), 85.3 (d, *J* = 3.6 Hz), 117.3, 117.4, 123.7, 123.8, 125.6, 125.7, 128.7, 128.8, 130. 2, 132.2, 132.3, 147.5, 147.6, 153.4 (d, *J* = 1.7 Hz), 154.0 (d, *J* = 2.3 Hz), 158.1 (d, *J* = 294.5 Hz), 158.4 (d, *J* = 292.6 Hz); ^{19}F NMR (376 MHz, $CDCl_3$) δ: -71.6 (s), -76.1 (s) (1F, 1:1.3); MS (EI) *m/z*: 340 (M^+); HRMS (EI) Calcd. for $C_{17}H_9ClF_4O$: 340.0278 (M^+), Found: 340.0279; Mp: 55.5-57.7.

4-(4-Acetylphenyl)-2-chloro-1-fluoro-1-phenoxybut-1-en-3-yne (3k)

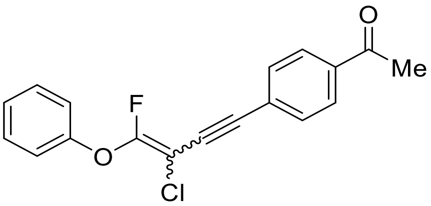

Reaction time was 16.5 h.

The title product (**3k**) was purified by column chromatography (hexane:AcOEt = 4:1). **3k** was obtained in 76% yield (119.7 mg).

A yellow oil; ^{1}H NMR (400 MHz, $CDCl_3$) δ: 2.59 (s) and 2.62 (s) (3H), 7.15 (2H, t, *J* = 7.3 Hz), 7.23 (1H, td, *J* = 7.3, 1.0 Hz), 7.39 (2H, d, *J* = 7.5 Hz), 7.40-7.43 (1H, m), 7.58 (1H, d, *J* = 8.1 Hz), 7.88 (1H, d, *J* = 8.3 Hz), 7.94 (1H, d, *J* = 8.3 Hz); ^{13}C NMR (100 MHz, $CDCl_3$) δ: 26.79, 26.82, 82.9 (d, *J* = 7.3 Hz), 83.3 (d, *J* = 3.8 Hz), 84.7 (d, *J* = 33.6 Hz), 85.2 (d, *J* = 40.6 Hz), 95.5 (d, *J* = 5.9 Hz), 96.1 (d, *J* = 8.3 Hz), 117.3, 125.46, 125.50, 126.7, 126.8, 128.3, 128.4, 130.2, 131.68, 131.72, 136.8, 136.9, 153.6 (d, *J* = 2.3 Hz), 154.2 (d, *J* = 2.4 Hz), 157.8 (d, *J* = 293.1 Hz), 158.0 (d, *J* = 292.5 Hz), 197.3, 197.4; ^{19}F NMR (376 MHz, $CDCl_3$) δ: -73.0 (s) and -77.3 (s) (1F, 1:1.1); MS (EI) *m/z*: 314 (M^+); HRMS (EI) Calcd. for $C_{18}H_{12}ClFO_2$: 314.0510 (M^+), Found: 314.0511.

2-Chloro-4-(4-formylphenyl)-1-fluoro-1-phenoxybut-1-en-3-yne (3l)

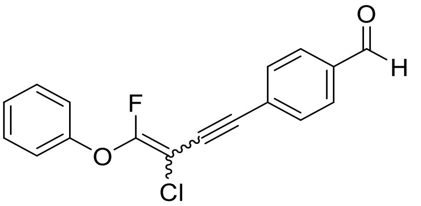

Reaction time was 28.5 h.

The title product (**3l**) was purified by column chromatography (hexane:AcOEt = 4:1). **3l** was obtained in 52% yield (77.5 mg).

A yellow oil; ^{1}H NMR (400 MHz, $CDCl_3$) δ: 7.15 (2H, td, *J* = 7.5, 1.0 Hz), 7.23 (1H, td, *J* = 7.4, 1.0 Hz), 7.41 (2H, t, *J* = 8.0 Hz), 7.49 (1H, d, *J* = 8.2 Hz), 7.64 (1H, d, *J* = 8.1 Hz), 7.81 (1H, d, *J* = 8.2 Hz), 7.87 (1H, d, *J* = 8.1 Hz); ^{13}C NMR (100 MHz, $CDCl_3$) δ: 83.6 (d, *J* = 7.5 Hz), 84.0 (d, *J* = 3.4 Hz), 84.6 (d, *J* = 28.4 Hz), 85.0 (d, *J* = 35.0 Hz), 117.3, 125.50, 125.54, 128.0, 128.1, 129.6, 129.7, 130.2, 132.03, 132.07, 135.9, 136.0, 153.5 (d, *J* = 2.1 Hz), 154.1 (d, *J* = 2.4 Hz), 157.9 (d, *J* = 294.1 Hz), 158.2 (d, *J* = 292.7 Hz), 191.4, 191.5; ^{19}F NMR (376 MHz, $CDCl_3$) δ: -72.5 (s) and -76.9 (s) (1F, 1:1.1); MS (EI) *m*/*z*: 300 (M^+); HRMS (EI) Calcd. for $C_{17}H_{10}ClFO_2$: 300.0353 (M^+), Found: 300.0351.

2-Chloro-1-fluoro-4-(3-hydroxyphenyl)-1-phenoxybut-1-en-3-yne (3m)

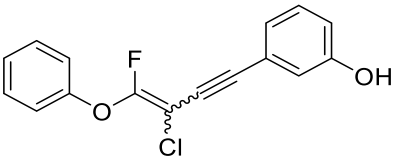

Reaction time was 15.5 h.

The title product (**3m**) was purified by column chromatography and preparative TLC (hexane:AcOEt = 3:1). **3m** was obtained in 38% yield (55.0 mg).

A yellow oil; ^{1}H NMR (400 MHz, $CDCl_3$) δ: 4.78 (br s) and 4.84 (br s) (1H), 6.77-6.89 (2H, m), 6.91-6.99 (1H, m), 7.06-7.25 (4H, m), 7.35-7.44 (2H, m); ^{13}C NMR (100 MHz, $CDCl_3$) δ: 79.8 (d, *J* = 7.5 Hz), 80.2 (d, *J* = 3.2 Hz), 85.3 (d, *J* = 35.9 Hz), 85.8 (d, *J* = 42.1 Hz), 96.30 (d, *J* = 67.6 Hz), 96.31 (d, *J* = 82.5 Hz), 116.6, 116.7, 117.1, 117.2, 118.17, 118.21, 123.1, 123.2, 124.4, 124.5, 125.29, 125.32, 129.8, 129.9, 130.11, 130.12, 153.7 (d, *J* = 2.3 Hz), 154.3 (d, *J* = 2.7 Hz), 155.3, 155.4, 157.4 (d, *J* = 292.1 Hz), 157.7 (d, *J* = 291.1 Hz); ^{19}F NMR (376 MHz, $CDCl_3$) δ: -74.4 (s) and -78.6 (s) (1F, 1:1); MS (EI) *m*/*z*: 288 (M^+); HRMS (EI) Calcd. for $C_{16}H_{10}ClFO_2$: 288.0353 (M^+), Found: 288.0353.

4-(4-Aminophenyl)-2-chloro-1-fluoro-1-phenoxybut-1-en-3-yne (3n)

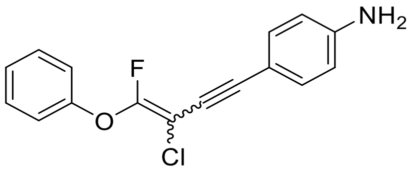

Reaction time was 15.5 h.

The title product (**3n**) was purified by column chromatography and preparative TLC (hexane:AcOEt = 4:1). **3n** was obtained in 87% yield (125.7 mg).

A brown oil; ^{1}H NMR (400 MHz, $CDCl_3$) δ: 3.84 (br s) and 3.88 (br s) (2H), 6.56 (1H, d, J = 8.4 Hz), 6.63 (1H, d, J = 7.4 Hz), 7.11-7.22 (4H, m), 7.31 (1H, d, J = 8.4 Hz), 7.35-7.41 (2H, m); ^{13}C NMR (100 MHz, $CDCl_3$) δ: 77.7 (d, J = 7.6 Hz), 78.0 (d, J = 2.5 Hz), 86.2 (d, J = 46.3 Hz), 86.7 (d, J = 53.9 Hz), 97.5 (d, J = 6.4 Hz), 98.3 (d, J = 8.2 Hz), 111.1, 114.6, 114.7, 116.9, 117.1, 125.06, 125.09, 130.0, 130.1, 133.2, 133.3, 147.5, 147.6, 153.9 (d, J = 2.8 Hz), 154.5 (d, J = 2.9 Hz), 156.8 (d, J = 290.1 Hz), 157.0 (d, J = 289.8 Hz); ^{19}F NMR (376 MHz, $CDCl_3$) δ: -76.5 (s) and -80.3 (s) (1F, 1:1.1); MS (EI) *m/z*: 287 (M^+); HRMS (EI) Calcd. for $C_{16}H_{11}ClFNO$: 287.0513 (M^+), Found: 287.0513.

2-Chloro-1-fluoro-1-phenoxy-4-(thiophene-2-yl)-but-1-en-3-yne (3o)

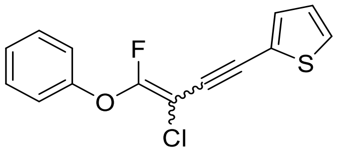

Multihalogenated vinyl ether (2.0 equiv) and 2-ethynylthiophene (1.0 equiv) was used. Reaction time was 15 h.

The title product (**3o**) was purified by column chromatography (hexane:AcOEt = 19:1) and preparative TLC (hexane only). **3o** was obtained in 37% yield (51.9 mg).

An orange oil; ^{1}H NMR (400 MHz, $CDCl_3$) δ: 6.94-7.05 (1H, m), 7.10-7.25 (3H, m), 7.28-7.45 (4H, m); ^{13}C NMR (100 MHz, $CDCl_3$) δ: 83.1 (d, J = 7.2 Hz), 83.6 (d, J = 3.4 Hz), 85.1 (d, J = 45.4 Hz), 85.8, 89.6 (d, J = 6.4 Hz), 90.3 (d, J = 8.1 Hz), 117.2, 117.3, 121.8, 122.0, 125.4, 127.3, 127.4, 128.8 (d, J = 13.3 Hz), 129.1, 130.09, 130.12, 133.3 (d, J = 6.7 Hz), 153.6 (d, J = 1.7 Hz), 154.2 (d, J = 2.2 Hz), 157.3 (d, J = 292.5 Hz), 157.6 (d, J = 292.5 Hz); ^{19}F NMR (376 MHz, $CDCl_3$) δ: -73.8 (s) and -77.8 (s) (1F, 1:1); MS (EI) *m/z*: 278 (M^+); HRMS (EI) Calcd. for $C_{14}H_8ClFOS$: 277.9968 (M^+), Found: 277.9968.

2-Chloro-1-fluoro-1-phenoxy-oct-1-en-3-yne (3p)

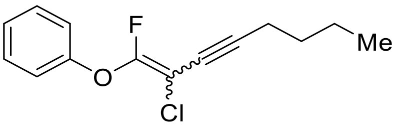

Reaction time was 15 h at 50 °C.

The title product (**3p**) was purified by column chromatography (hexane only). **3p** was obtained in 89% yield (112.0 mg).

An colorless oil; ^{1}H NMR (400 MHz, $CDCl_3$) δ: 0.85 (t, J = 7.3 Hz) and 0.94 (t, J = 7.3 Hz) (3H), 1.32 (tq, J = 7.3, 7.3 Hz) and 1.45 (tq, J = 7.3, 7.3 Hz) (2H), 1.44 (tt, J = 7.3, 7.3 Hz) and 1.58 (tt, J = 7.3, 7.3 Hz) (2H), 2.31 (td, J = 6.9, 1.5 Hz) and 2.43 (td, J = 6.9, 1.5 Hz) (2H), 7.10 (2H, d, J = 7.9 Hz), 7.18 (1H, t, J = 7.4 Hz), 7.37 (1H, t, J = 7.9 Hz); ^{13}C NMR (100 MHz, $CDCl_3$) δ: 13.66, 13.73, 19.4, 19.5, 21.9, 22.1, 30.3, 30.4, 71.3 (d, J = 7.0 Hz), 71.6 (d, J = 2.3 Hz), 86.2 (d, J = 47.6 Hz), 86.6 (d, J = 54.3 Hz), 98.7 (d, J = 6.2 Hz), 99.6 (d, J = 9.0 Hz), 116.9, 124.97, 125.05, 129.98, 130.05, 153.9 (d, J = 2.4 Hz), 154.5 (d, J = 2.9 Hz), 157.0 (d, J = 289.3 Hz), 157.2 (d, J = 288.8 Hz); ^{19}F NMR (376 MHz, $CDCl_3$) δ: -77.4 (s) and -82.0 (s) (1F, 1:1.1); MS (EI) *m*/*z*: 252 (M^+); HRMS (EI) Calcd. for $C_{14}H_{14}ClFO$: 252.0717 (M^+), Found: 252.0715.

2-Chloro-4-cyclopropyl-1-fluoro-1-phenoxy-but-1-en-3-yne (3q)

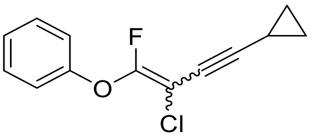

Reaction time was 14.5 h.

The title product (**3q**) was purified by column chromatography (hexane only). **3q** was obtained in 86% yield (102.3 mg).

A pale yellow oil; ^{1}H NMR (400 MHz, $CDCl_3$) δ: 0.61-1.00 (4H, m), 1.22-1.52 (1H, m), 7.05-7.12 (2H, m), 7.15-7.21 (1H, m), 7.32-7.41 (2H, m); ^{13}C NMR (100 MHz, $CDCl_3$) δ: 0.39, 0.48, 9.04, 9.11, 66.3 (d, J = 6.9 Hz), 66.6 (d, J = 2.9 Hz), 86.1 (d, J = 45.9 Hz), 86.7 (d, J = 53.8 Hz), 101.8 (d, J = 6.3 Hz), 102.6 (d, J = 8.3 Hz), 116.9, 117.0, 125.0, 125.1, 129.98, 130.05, 153.9 (d, J = 2.9 Hz), 154.5 (d, J = 3.1 Hz), 157.3 (d, J = 290.2 Hz), 157.6 (d, J = 289.2 Hz); ^{19}F NMR (376 MHz, $CDCl_3$) δ: -76.7 (s) and -81.7 (s) (1F, 1:1.4); MS (EI) *m*/*z*: 236 (M^+); HRMS (EI) Calcd. for $C_{13}H_{10}ClFO$: 236.0404 (M^+), Found: 236.0404.

2-Chloro-4-(cyclohex-1-ene-1-yl)-1-fluoro-1-phenoxy-but-1-en-3-yne (3r)

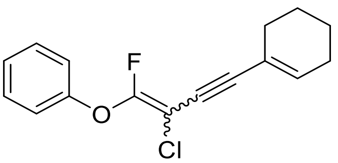

Reaction time was 15 h.

The title product (**3r**) was purified by column chromatography and preparative TLC (hexane only). **3r** was obtained in 52% yield (72.6 mg).

A yellow oil; ^{1}H NMR (400 MHz, $CDCl_3$) δ: 1.50-1.72 (4H, m), 2.00-2.23 (4H, m), 6.07-6.13 (m) and 6.22-6.28 (m) (1s), 7.07-7.14 (2H, m), 7.18 (1H, t, *J* = 7.4 Hz), 7.37 (1H, t, *J* = 7.8 Hz); ^{13}C NMR (100 MHz, $CDCl_3$) δ: 21.4, 21.5, 22.2, 22.3, 25.8, 25.9, 28.6, 28.8, 86.0 (d, *J* = 47.0 Hz), 86.4 (d, *J* = 54.1 Hz), 98.5 (d, *J* = 6.3 Hz), 99.2 (d, *J* = 8.4 Hz), 116.9, 117.1, 119.9, 120.0, 125.06, 125.11, 130.0, 130.1, 137.2, 137.4, 138.3, 153.9 (d, *J* = 2.8 Hz), 154.5 (d, *J* = 2.9 Hz), 156.7 (d, *J* = 291.3 Hz), 157.1 (d, *J* = 290.3Hz); ^{19}F NMR (376 MHz, $CDCl_3$) δ: -76.3 (s) and -80.3 (s) (1F, 1:1); MS (EI) *m*/*z*: 276 (M^+); HRMS (EI) Calcd. for $C_{16}H_{14}ClFO$: 276.0717 (M^+), Found: 276.0717.

5-Chloro-6-fluoro-6-phenoxy-hex-5-en-3-yn-1-ol (3s)

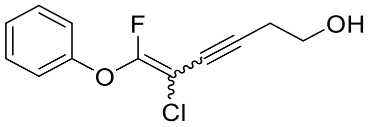

Reaction time was 13.5 h.

The title product (**3s**) was purified by column chromatography (hexane:AcOEt = 7:3). **3s** was obtained in 53% yield (65.3 mg).

An orange oil; ^{1}H NMR (400 MHz, $CDCl_3$) δ: 1.64 (br s) and 1.82 (br s) (1H), 2.58 (1H, td, *J* = 6.3, 1.5 Hz), 2.71 (1H, td, *J* = 6.3, 1.5 Hz), 3.66 (1H, t, *J* = 6.3 Hz), 3.81 (1H, t, *J* = 6.3 Hz), 7.07-7.13 (2H, m), 7.16-7.23 (1H, m), 7.33-7.42 (2H, m); ^{13}C NMR (100 MHz, $CDCl_3$) δ: 24.1, 24.2, 60.7, 60.8, 73.1 (d, *J* = 7.1 Hz), 73.5 (d, *J* = 3.1 Hz), 85.4 (d, *J* = 37.2 Hz), 85.9 (d, *J* = 45.4 Hz), 94.8 (d, *J* = 6.2 Hz), 95.6 (d, *J* = 8.4 Hz), 116.9, 117.0 , 125.18, 125.22, 130.1, 153.7 (d, *J* = 2.5 Hz), 154.3 (d, *J* = 3.0 Hz), 157.4 (d, *J* = 290.7 Hz), 157.7 (d, *J* = 289.1 Hz); ^{19}F NMR (376 MHz, $CDCl_3$) δ: -75.9 (s) and -80.6 (s) (1F, 1:1); MS (EI) *m*/*z*: 240 (M^+); HRMS (EI) Calcd. for $C_{12}H_{10}ClFO_2$: 240.0353 (M^+), Found: 240.0350.

(3-Chloro-4-fluoro-4-(3-methoxyphenoxy)but-3-en-1-yn-1-yl)trimethylsilane (3t)

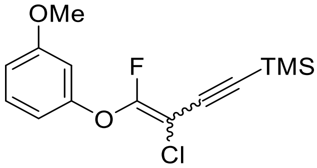

Reaction time was 12.5 h.

The title product (**3t**) was purified by column chromatography (pentane only). **3t** was obtained in 35% yield (51.6 mg).

A pale yellow oil; ^{1}H NMR (400 MHz, $CDCl_3$) δ: 0.14 (s) and 0.24 (s) (9H), 3.81 (3H, s), 6.62-6.72 (2H, m), 6.74 (1H, dd, *J* = 8.5, 2.3 Hz), 7.25 (d, *J* = 8.2 Hz) and 7.27 (d, *J* = 8.3 Hz) (1H); ^{13}C NMR (100 MHz, $CDCl_3$) δ: -0.35, -0.25, 55.6, 55.7, 85.4 (d, *J* = 44.4 Hz), 85.8 (d, *J* = 52.9 Hz), 94.3 (d, *J* = 7.0 Hz), 94.7 (d, *J* = 2.4 Hz), 103.44 (d, *J* = 6.7 Hz), 103.48, 103.7, 104.1 (d, *J* = 8.0 Hz), 109.1 (d,

J = 26.1 Hz), 110.9 (d, J = 5.8 Hz), 130.4, 130.5, 154.6 (d, J = 2.5 Hz), 155.3 (d, J = 2.5 Hz), 157.9 (d, J = 292.8 Hz), 158.6 (d, J = 291.1 Hz), 161.0, 161.1; ^{19}F NMR (376 MHz, $CDCl_3$) δ: -73.4 (s) and -78.3 (s) (1F, 1:1.1); MS (EI) *m/z*: 298 (M^+); HRMS (EI) Calcd. for $C_{14}H_{16}ClFO_2Si$: 298.0592 (M^+), Found: 298.0593.

(3-Chloro-4-fluoro-4-(4-nitrophenoxy)but-3-en-1-yn-1-yl)trimethylsilane (3u)

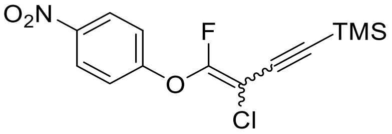

5a (1.3 equiv) was used. Reaction time was 22 h.
The title product (**3u**) was purified by column chromatography (pentane only). **3u** was obtained in 29% yield (38.0 mg).
A pale yellow oil; ^{1}H NMR (400 MHz, $CDCl_3$) δ: 0.10 (s) and 0.26 (s) (9H), 7.22 (dd, J = 9.4, 1.0 Hz) and 7.23 dd, J = 9.4, 1.0 Hz) (2H), 8.29 (d, J = 9.4 Hz) and 8.30 (d, J = 9.4 Hz) (2H); ^{13}C NMR (100 MHz, $CDCl_3$) δ: -0.50, -0.36, 87.3 (d, J = 43.2 Hz), 87.9 (d, J = 51.3 Hz), 93.2 (d, J = 7.0 Hz), 93.7 (d, J = 1.9 Hz), 105.1 (d, J = 5.5 Hz), 105.7 (d, J = 8.2 Hz), 117.1, 117.2, 126.1, 126.2, 144.77, 144.84, 156.9 (d, J = 293.0 Hz), 157.3 (d, J = 291.5 Hz), 158.0 (d, J = 3.8 Hz), 158.6 (d, J = 3.8 Hz); ^{19}F NMR (376 MHz, $CDCl_3$) δ: -75.4 (s) and -79.9 (s) (1F, 1:1.2); MS (EI) *m/z*: 313 (M^+); HRMS (EI) Calcd. for $C_{13}H_{13}ClFNO_3Si$: 313.1337 (M^+), Found: 313.0334.

(3-Chloro-4-(4-ethoxycarbonylphenoxy)-4-fluorobut-3-en-1-yn-1-yl)trimethylsilane (3v)

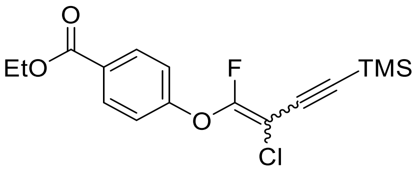

Reaction time was 20.5 h.
The title product (**3v**) was purified by column chromatography and preparative TLC (hexane:AcOEt = 14:1). **3u** was obtained in 35% yield (46.8 mg).
A pale yellow oil; ^{1}H NMR (400 MHz, $CDCl_3$) δ: 0.11 (s) and 0.25 (s) (9H), 1.40 (3H, t, J = 7.1 Hz), 4.379 (q, J = 7.1 Hz) and 4.381 (q, J = 7.1 Hz) (2H), 7.13 (dd, J = 9.0, 1.0 Hz) and 7.14 (dd, J = 8.7, 0.9 Hz) (2H), 8.076 (dd, J = 9.0, 1.0 Hz) and 8.078 (dd, J = 8.7, 0.9 Hz) (2H); ^{13}C NMR (100 MHz, $CDCl_3$) δ: -0.43, -0.30, 14.5, 61.3, 86.4 (d, J = 44.9 Hz), 86.9 (d, J = 52.8 Hz), 93.8 (d, J = 7.1 Hz), 94.2 (d, J = 2.3 Hz), 104.2 (d, J = 5.3 Hz), 104.8 (d, J = 8.4 Hz), 116.5, 116.6, 127.45, 127.55, 131.9, 132.0, 156.9 (d, J = 2.9 Hz), 157.4 (d, J = 292.9 Hz), 157.6 (d, J = 3.8 Hz),158.0 (d, J = 291.6 Hz), 165.71, 165.74; ^{19}F NMR (376 MHz, $CDCl_3$) δ: -74.2 (s) and -79.0 (s) (1F, 1:1.6); MS (EI) *m/z*: 340 (M^+); HRMS (EI) Calcd. for $C_{16}H_{18}ClFO_3Si$: 340.0698 (M^+), Found: 340.0695.

(4-(3-Aminophenoxy)-3-chloro-4-fluorobut-3-en-1-yn-1-yl)trimethylsilane (3w)

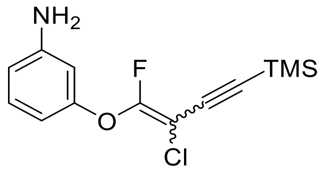

Reaction temperature was rt to 50 °C. Reaction time was 110 h.

The title product (**3w**) was purified by preparative TLC (hexane:AcOEt = 7:3). **3w** was obtained in 7% yield (9.7 mg).

An orange oil; ^{1}H NMR (400 MHz, $CDCl_3$) δ: 0.15 (s) and 0.24 (s) (9H), 3.78 (2H, br s), 6.37-6.43 (1H, m), 6.44-6.52 (2H, m), 7.11 (1H, t, J = 8.1 Hz); ^{13}C NMR (100 MHz, $CDCl_3$) δ: -0.31, -0.23, 85.1 (d, J = 46.6 Hz), 85.6 (d, J = 55.1 Hz), 94.4 (d, J = 7.9 Hz), 94.9 (d, J = 2.6 Hz), 103.2 (d, J = 6.1 Hz), 103.6, 103.8, 103.9 (d, J = 8.8 Hz), 106.7, 107.0, 111.87, 111.92, 130.6, 130.7, 148.2, 148.3, 154.7 (d, J = 2.9 Hz), 155.4 (d, J = 3.0 Hz), 158.1 (d, J = 293.2 Hz), 158.6 (d, J = 291.1 Hz); ^{19}F NMR (376 MHz, $CDCl_3$) δ: -73.0 (s) and -77.8 (s) (1F, 1:1.3); MS (EI) *m/z*: 283 (M^+); HRMS (EI) Calcd. for $C_{13}H_{15}ClFNOSi$: 283.0595 (M^+), Found: 283.0591.

^{1}H and ^{13}C NMR charts

2-Chloro-1-fluoro-2-phenylethenyl phenyl ether (2a)

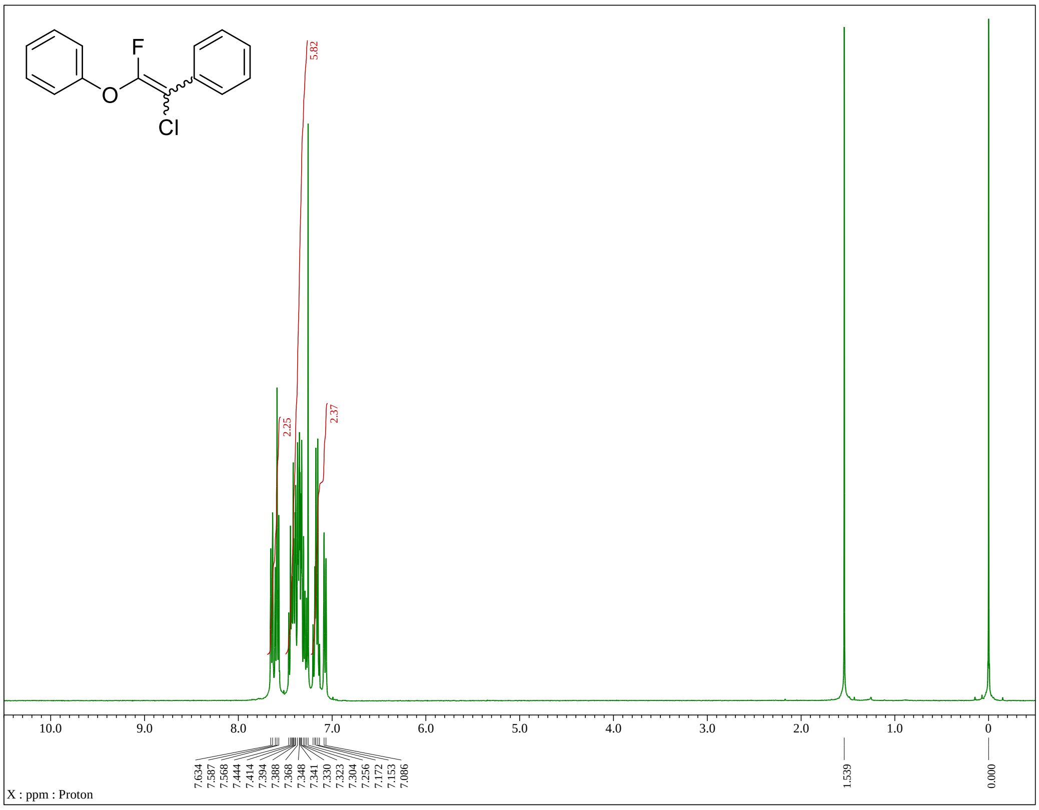

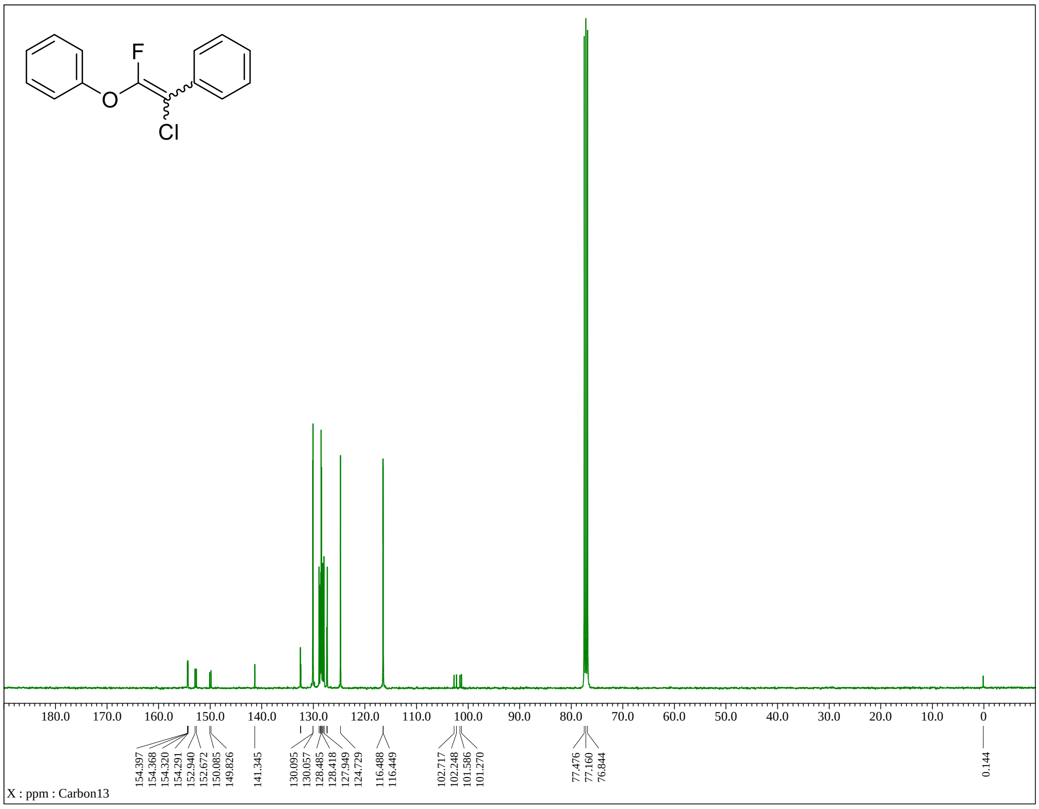

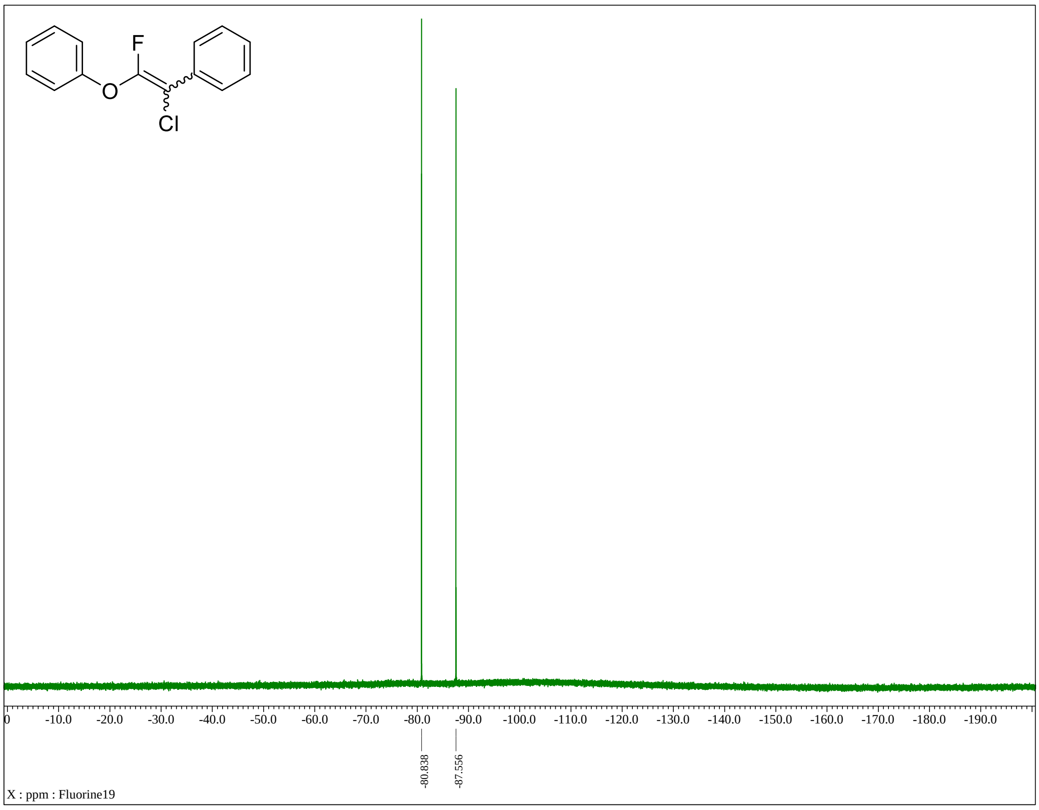

2-Chloro-1-fluoro-2-(4-tolyl)ethenyl phenyl ether (2b)

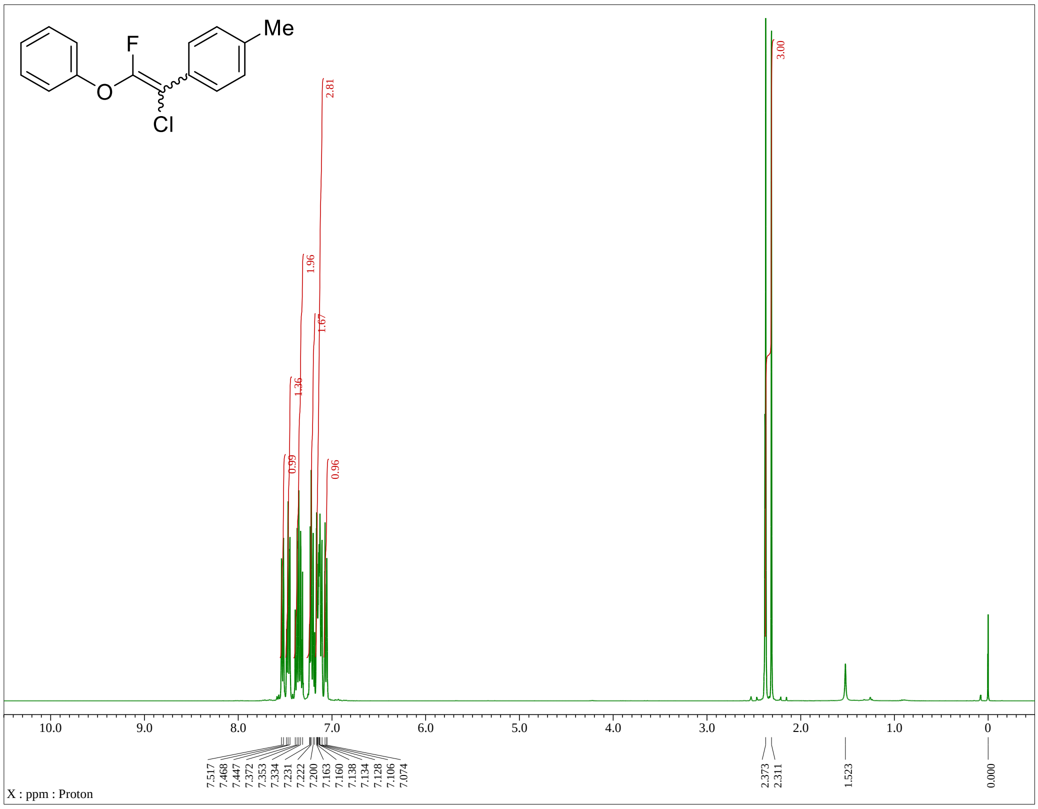

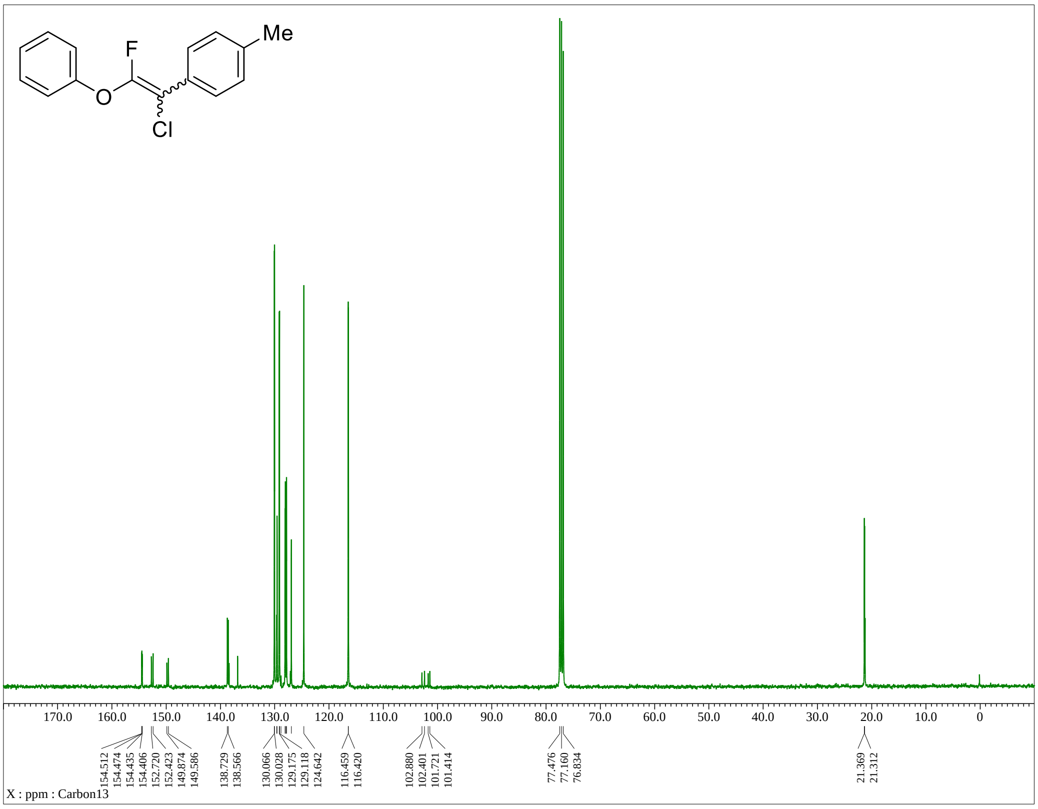
Me
F
O
Cl
170.0
160.0
150.0
140.0
130.0
120.0
110.0
100.0
90.0
80.0
70.0
60.0
50.0
40.0
30.0
20.0
10.0
0
154.512
154.474
154.435
154.406
152.720
152.423
149.874
149.586
138.729
138.566
130.066
130.028
129.175
129.118
124.642
116.459
116.420
102.880
102.401
101.721
101.414
77.476
77.160
76.834
21.369
21.312
X : ppm : Carbon13

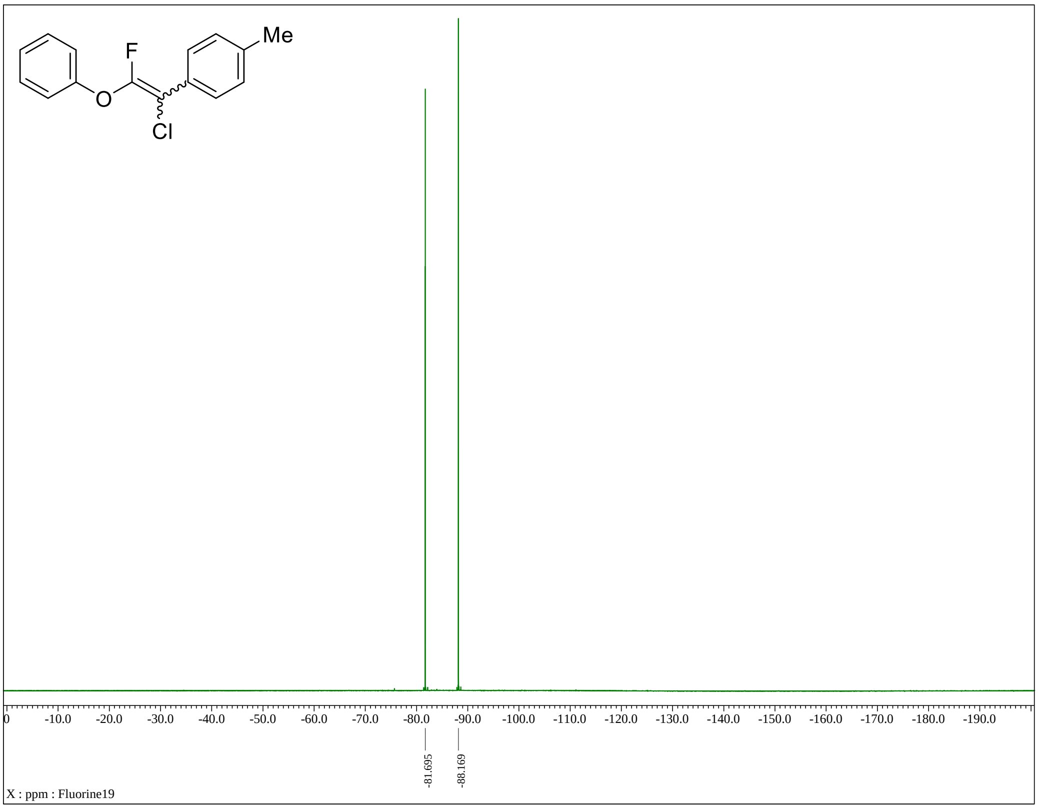
Me
F
O
Cl
0
-10.0
-20.0
-30.0
-40.0
-50.0
-60.0
-70.0
-80.0
-90.0
-100.0
-110.0
-120.0
-130.0
-140.0
-150.0
-160.0
-170.0
-180.0
-190.0
-81.695
-88.169
X : ppm : Fluorine19

2-Chloro-1-fluoro-2-(3-tolyl)ethenyl phenyl ether (2c)

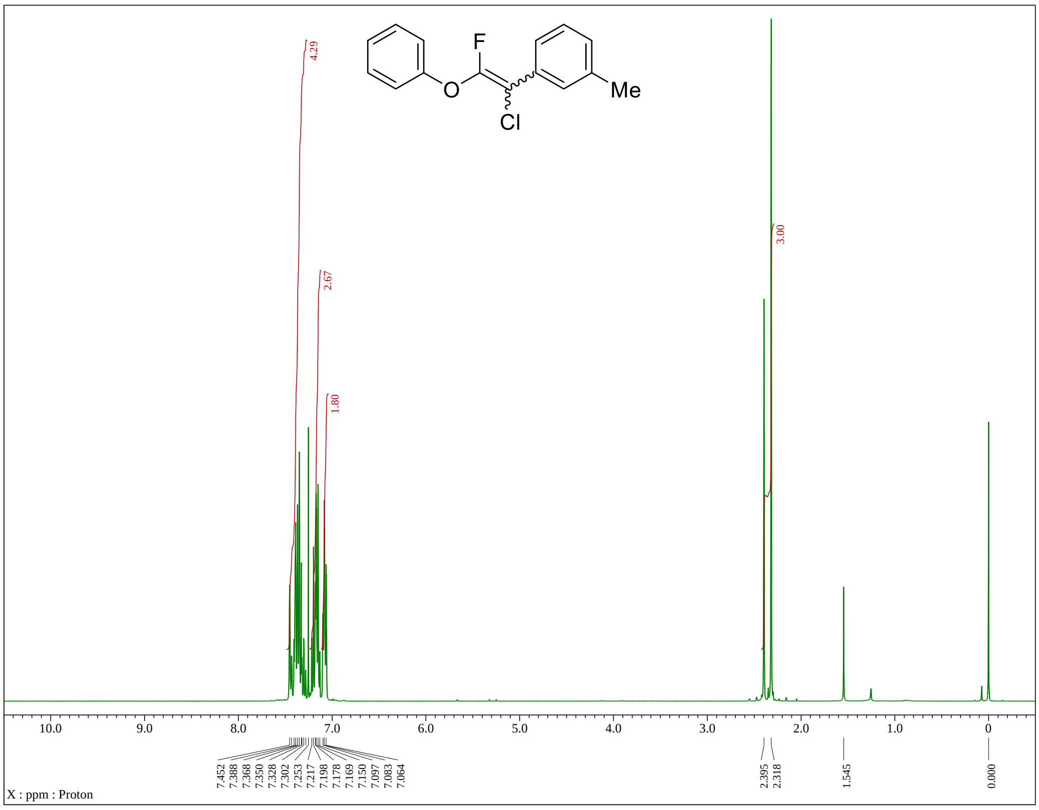

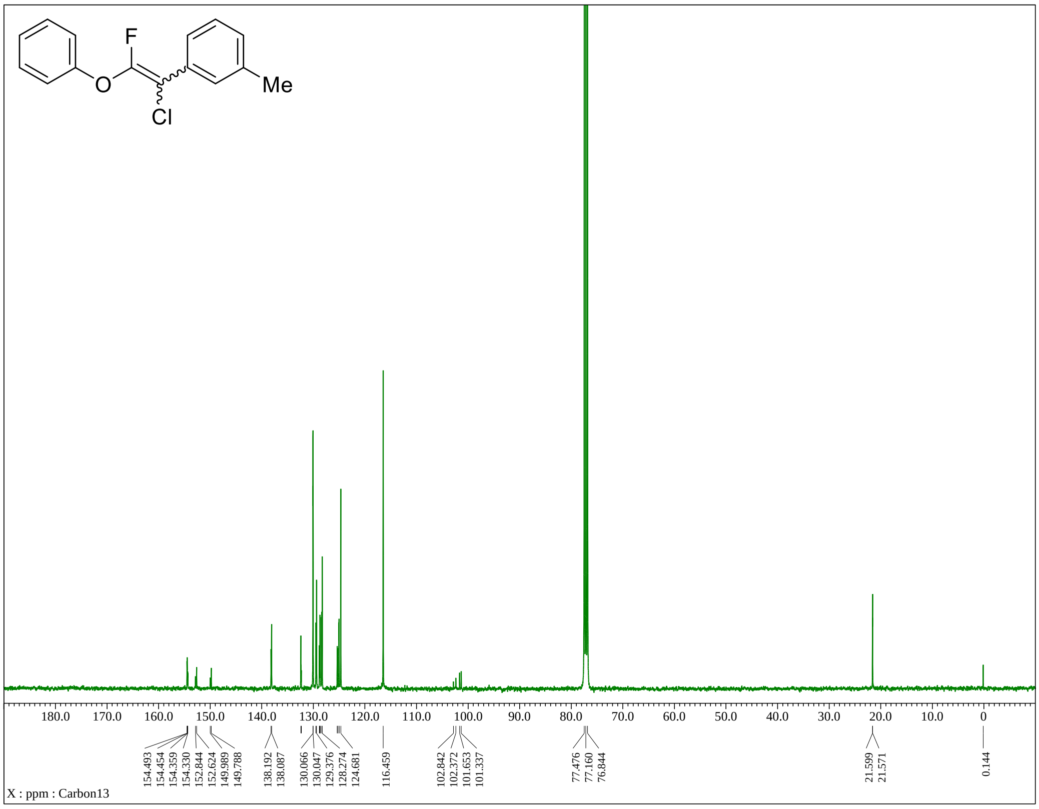

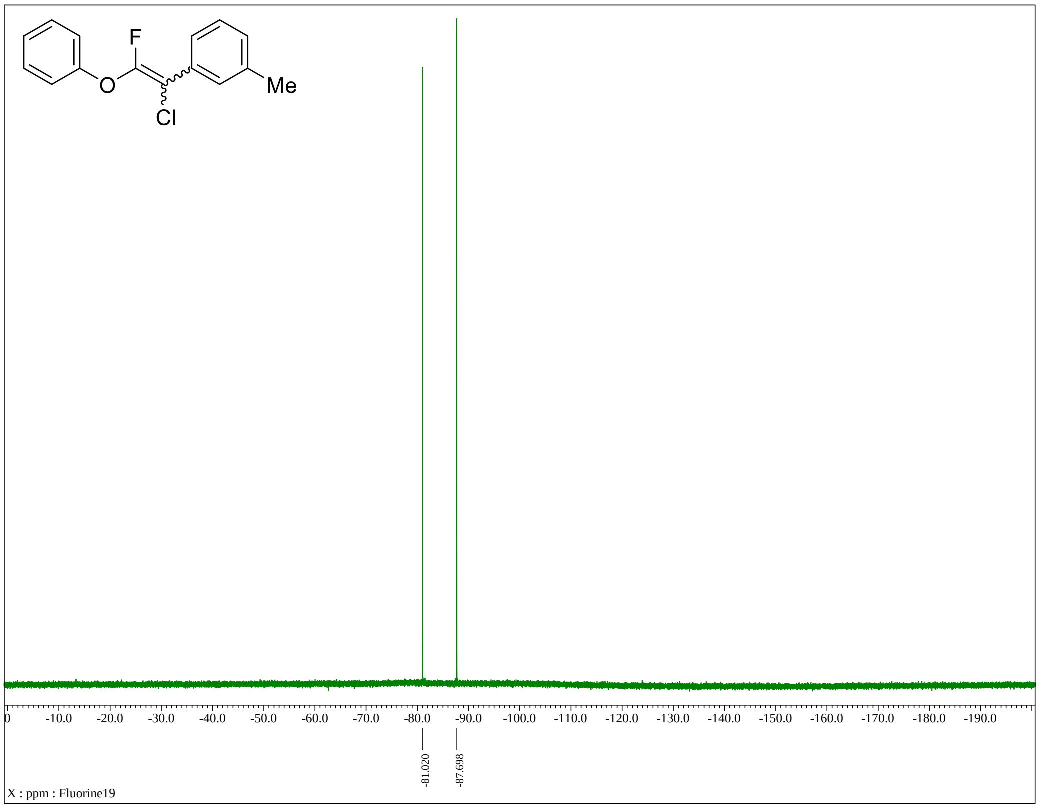

2-Chloro-1-fluoro-2-(2-tolyl)ethenyl phenyl ether (2d)

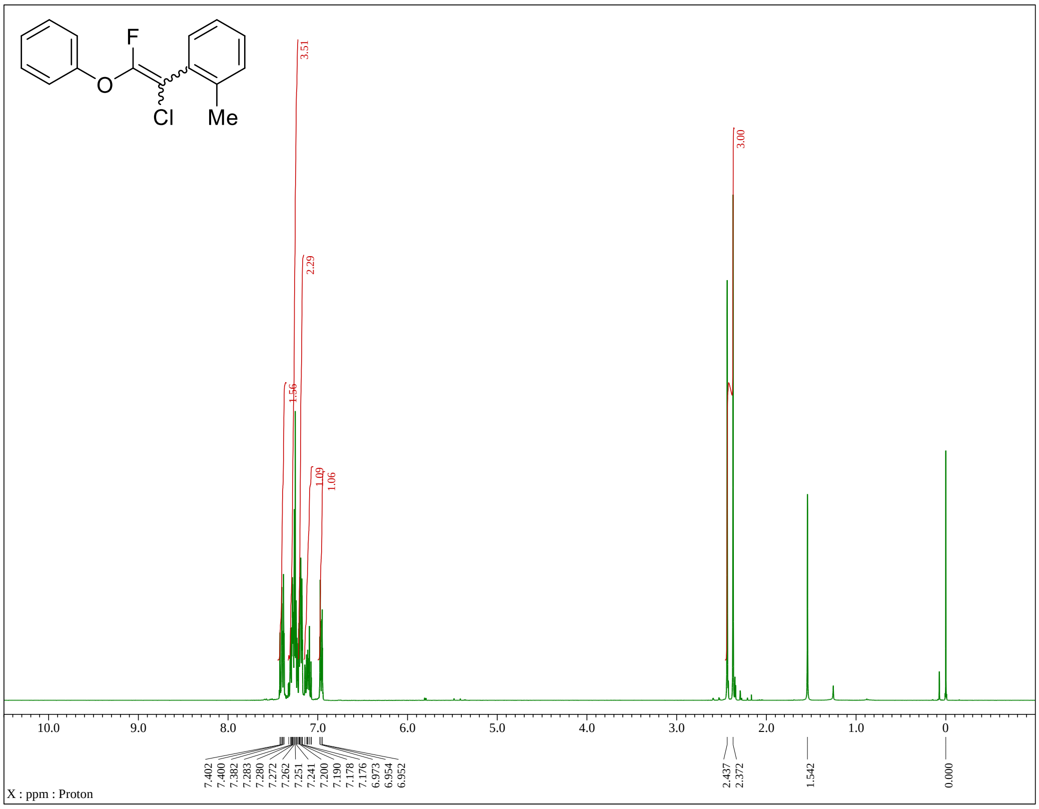

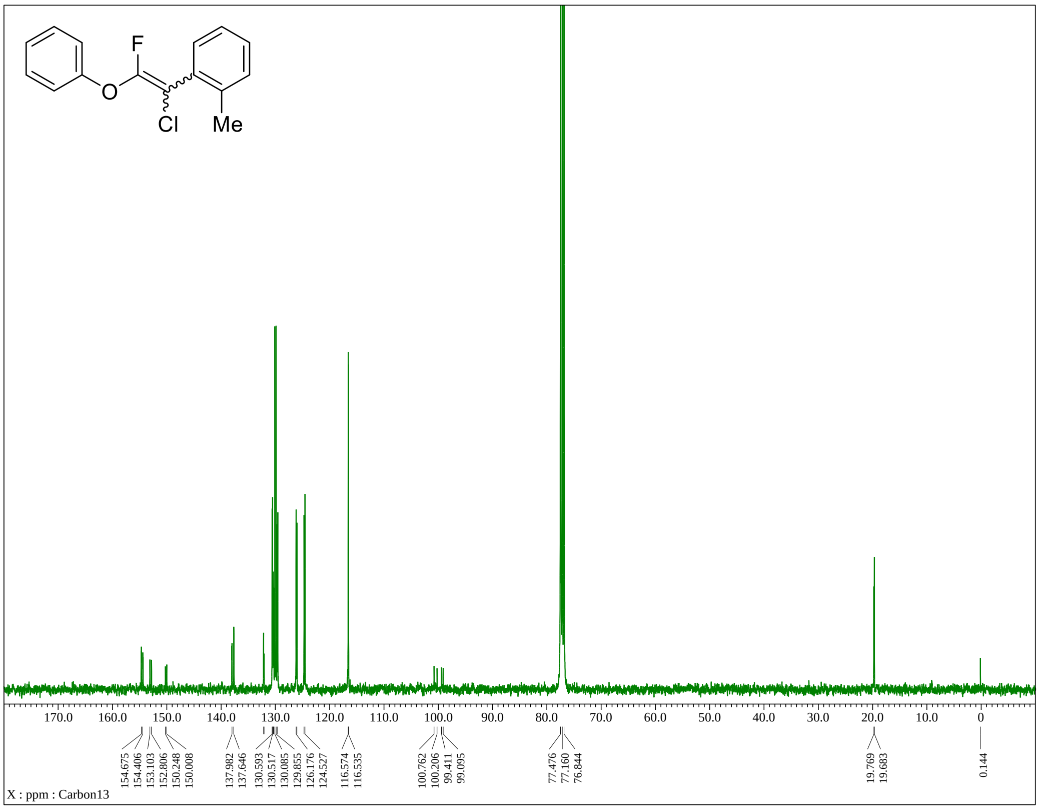
F
O
Cl
Me
170.0 160.0 150.0 140.0 130.0 120.0 110.0 100.0 90.0 80.0 70.0 60.0 50.0 40.0 30.0 20.0 10.0 0
154.675 154.406 153.103 152.806 150.248 150.008 137.982 137.646 130.593 130.517 130.085 129.855 126.176 124.527 116.574 116.535 100.762 100.206 99.411 99.095 77.476 77.160 76.844 19.769 19.683 0.144
X : ppm : Carbon13

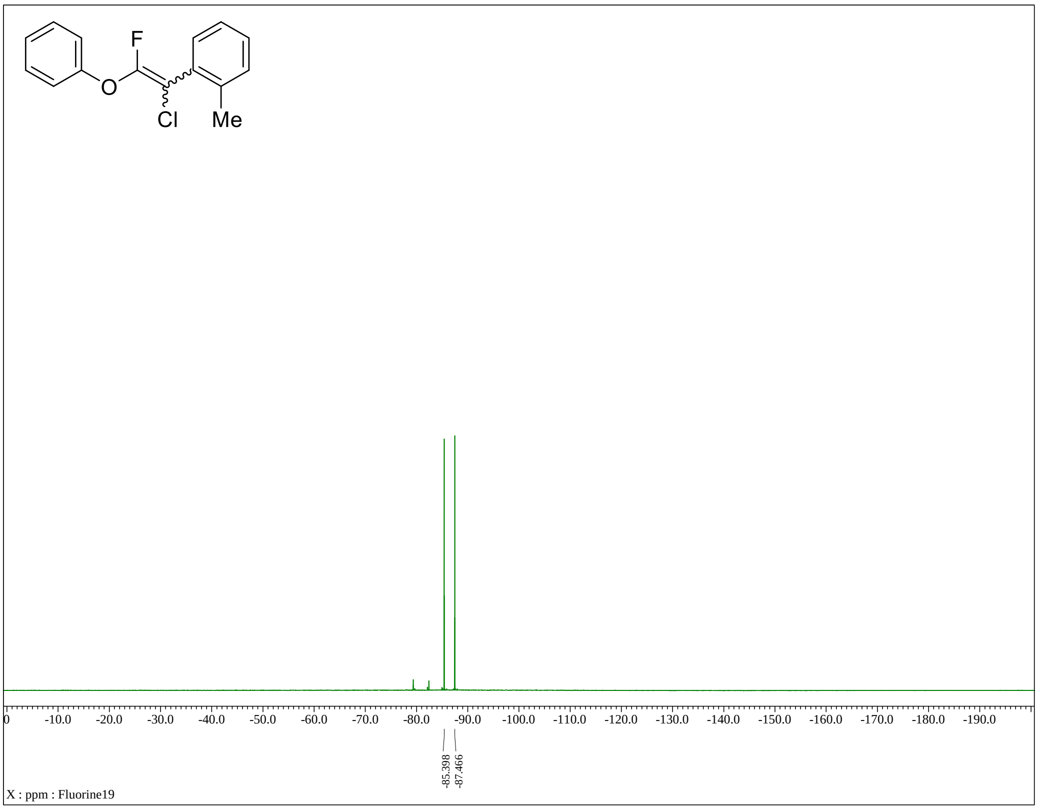
F
O
Cl
Me
0 -10.0 -20.0 -30.0 -40.0 -50.0 -60.0 -70.0 -80.0 -90.0 -100.0 -110.0 -120.0 -130.0 -140.0 -150.0 -160.0 -170.0 -180.0 -190.0
-85.398 -87.466
X : ppm : Fluorine19

2-Chloro-1-fluoro-2-(3,4-methylenedioxyphenyl)ethenyl phenyl ether (2e)

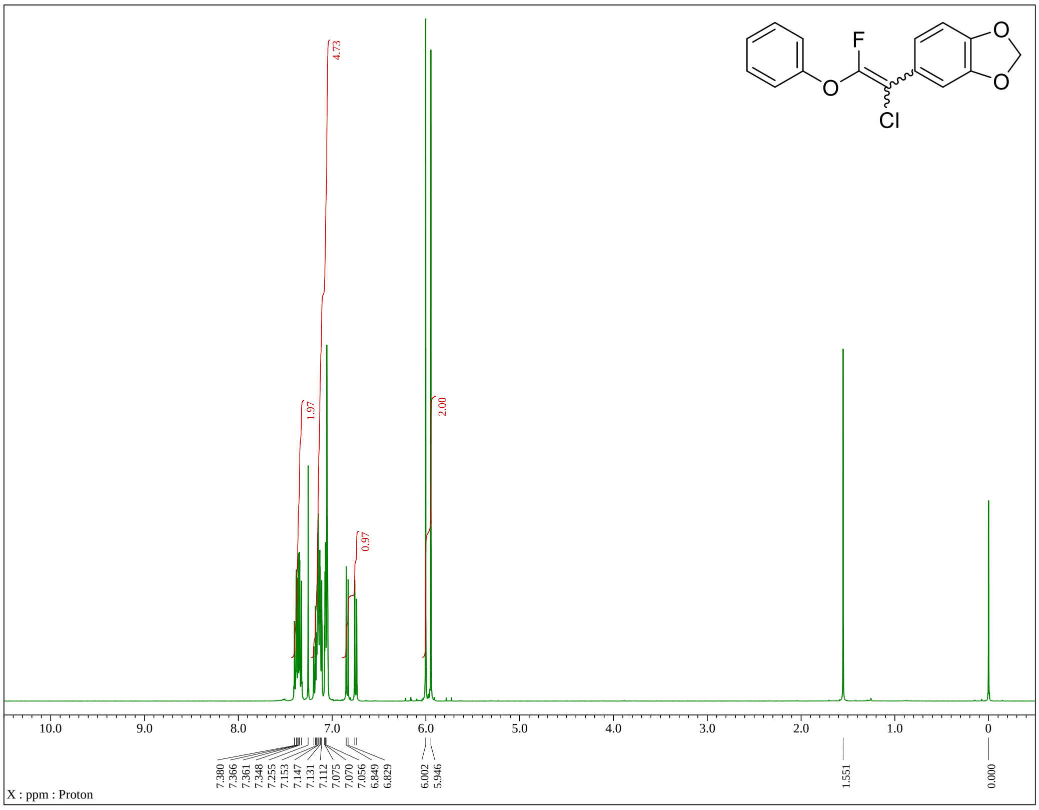

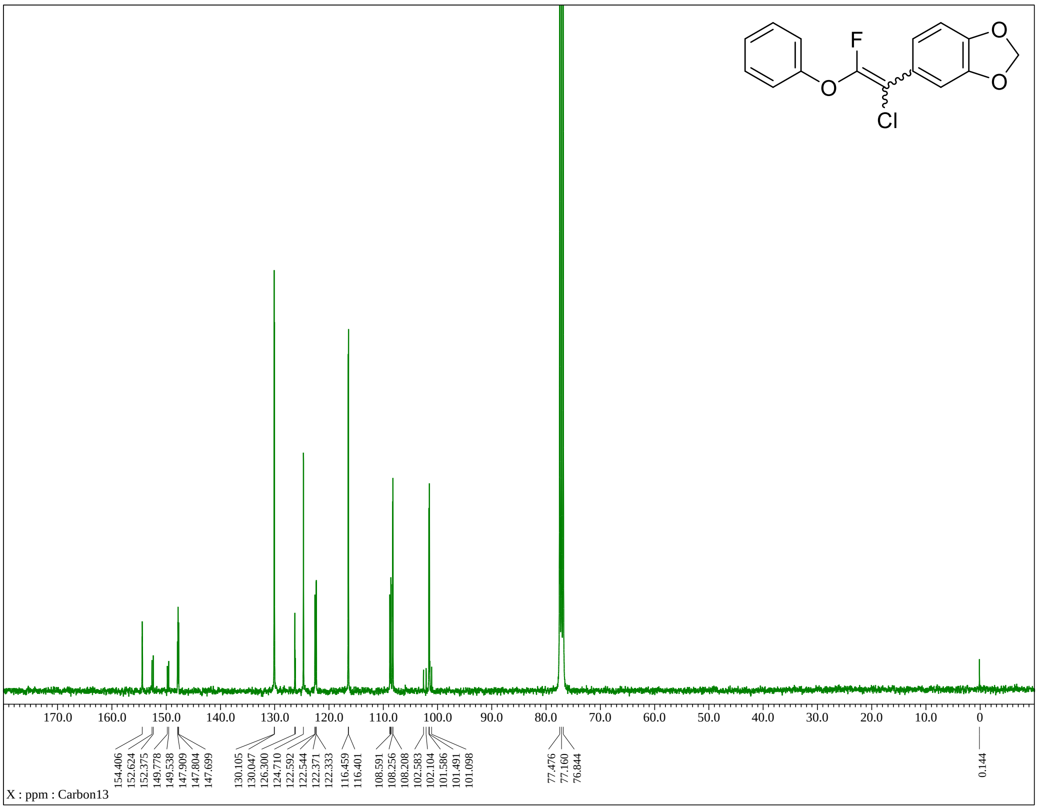

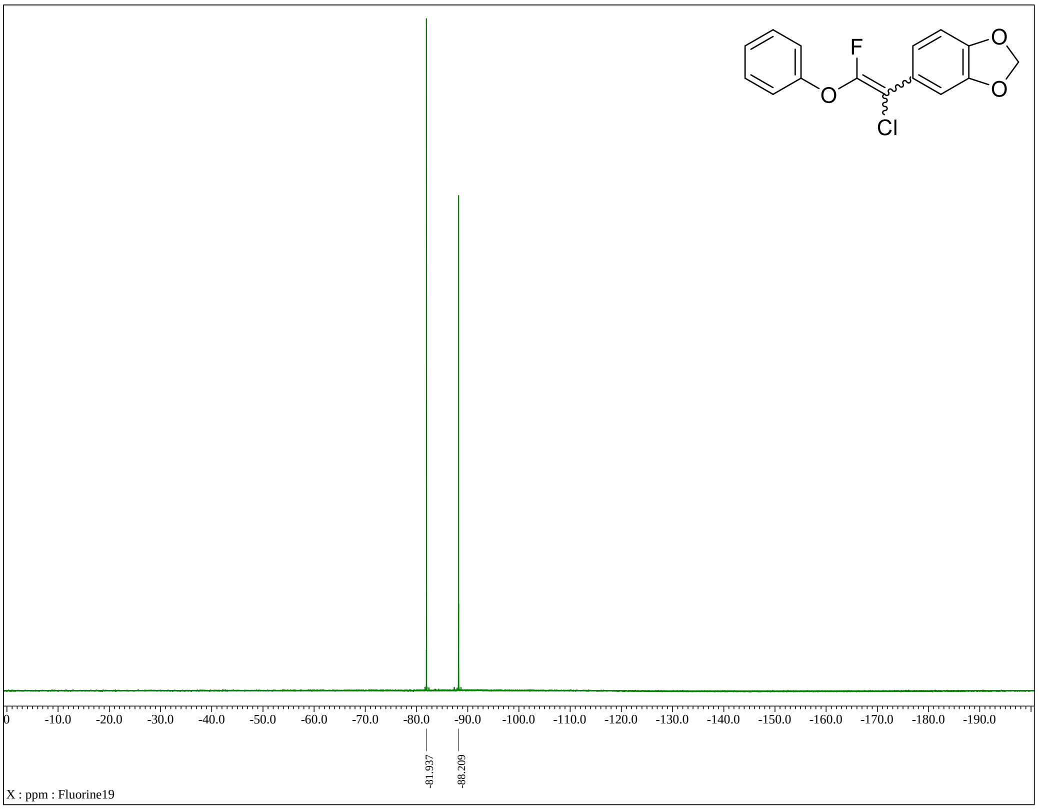

2-Chloro-1-fluoro-2-(4-fluorophenyl)ethenyl phenyl ether (2f)

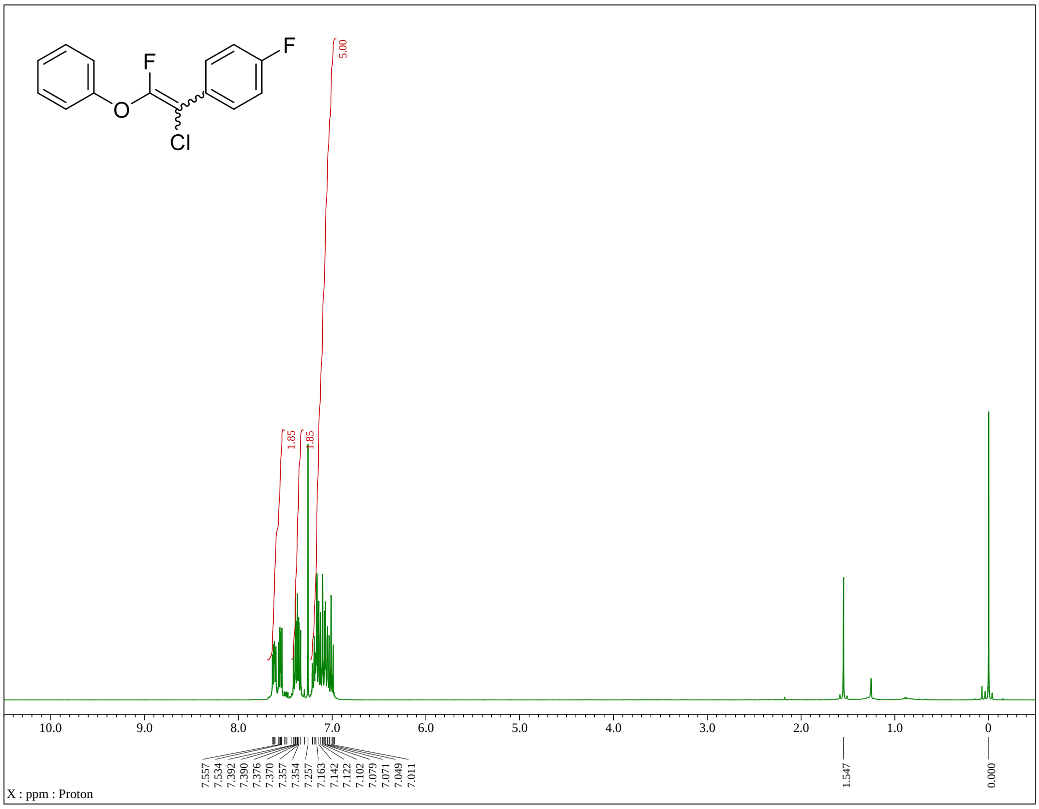

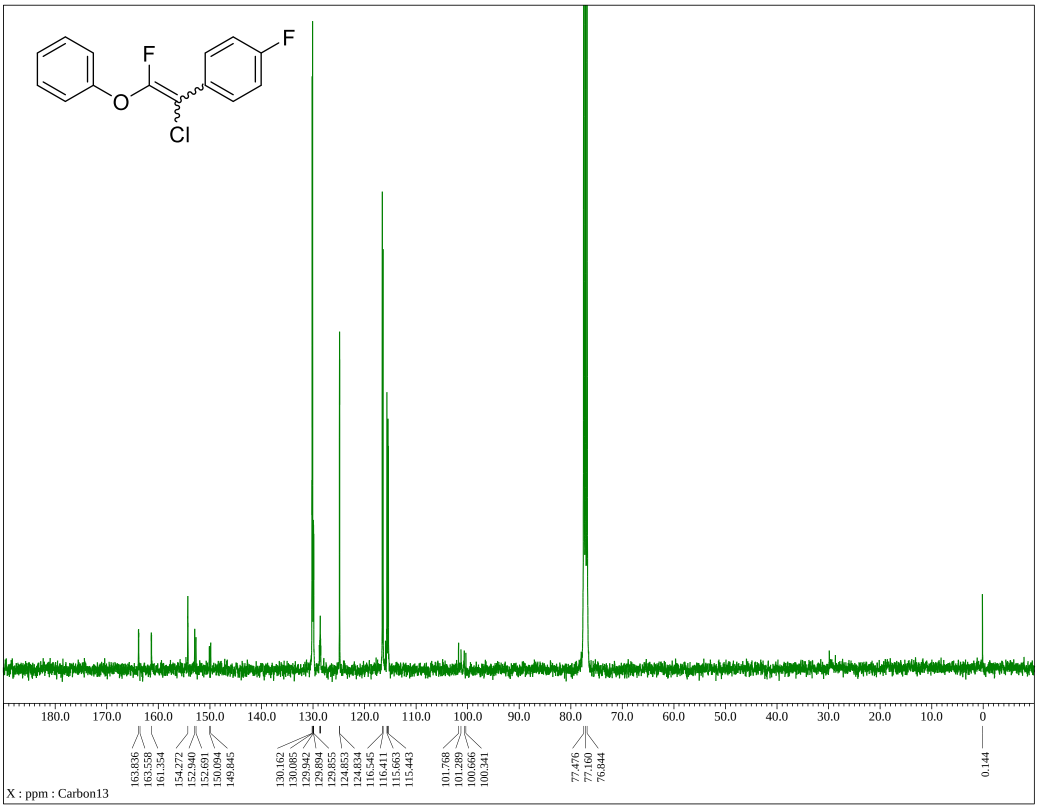
F
F
O
Cl
180.0 170.0 160.0 150.0 140.0 130.0 120.0 110.0 100.0 90.0 80.0 70.0 60.0 50.0 40.0 30.0 20.0 10.0 0
163.836
163.558
161.354
154.272
152.940
152.691
150.094
149.845
130.162
130.085
129.942
129.894
129.855
124.853
124.834
116.545
116.411
115.663
115.443
101.768
101.289
100.666
100.341
77.476
77.160
76.844
0.144
X : ppm : Carbon13

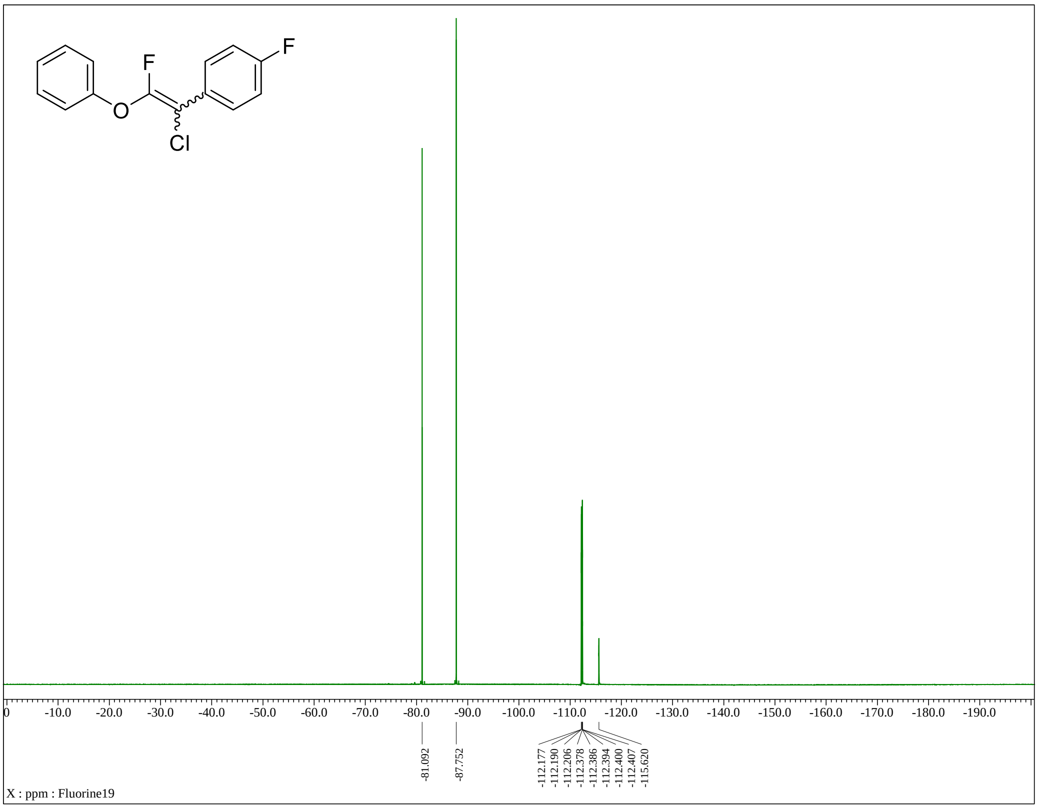
F
F
O
Cl
0 -10.0 -20.0 -30.0 -40.0 -50.0 -60.0 -70.0 -80.0 -90.0 -100.0 -110.0 -120.0 -130.0 -140.0 -150.0 -160.0 -170.0 -180.0 -190.0
-81.092
-87.752
-112.177
-112.190
-112.206
-112.378
-112.386
-112.394
-112.400
-112.407
-115.620
X : ppm : Fluorine19

2-(4-Acetylphenyl)-2-chloro-1-fluoroethenyl phenyl ether (2g)

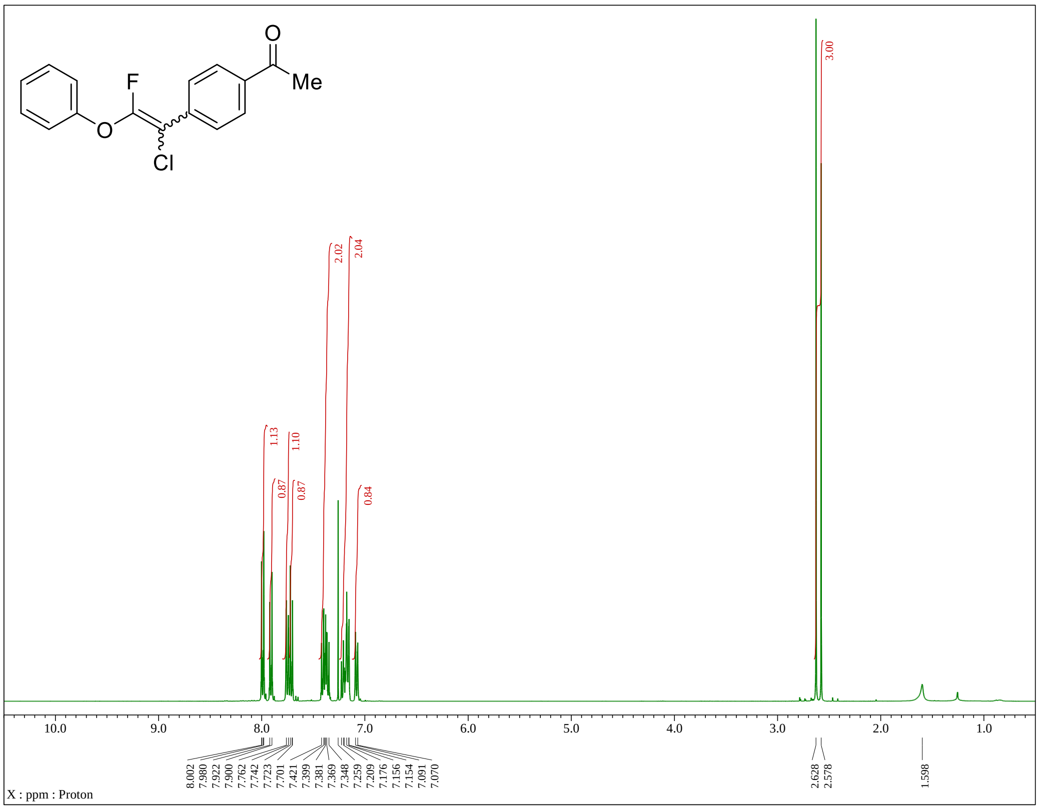

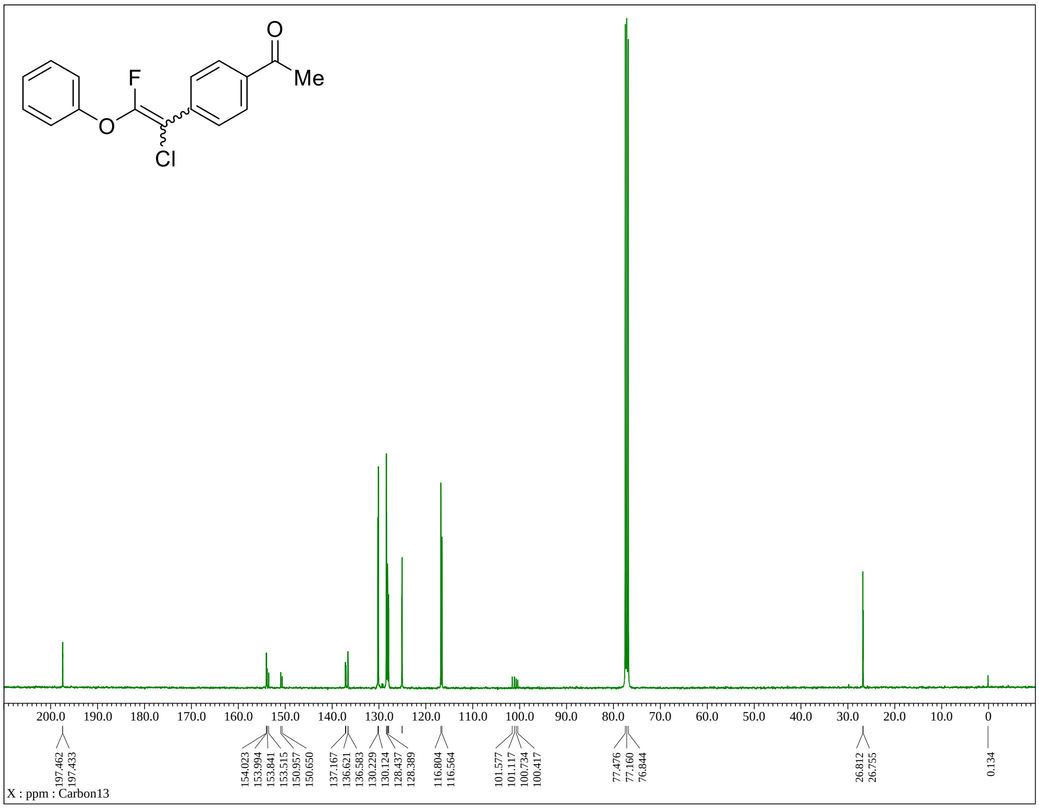

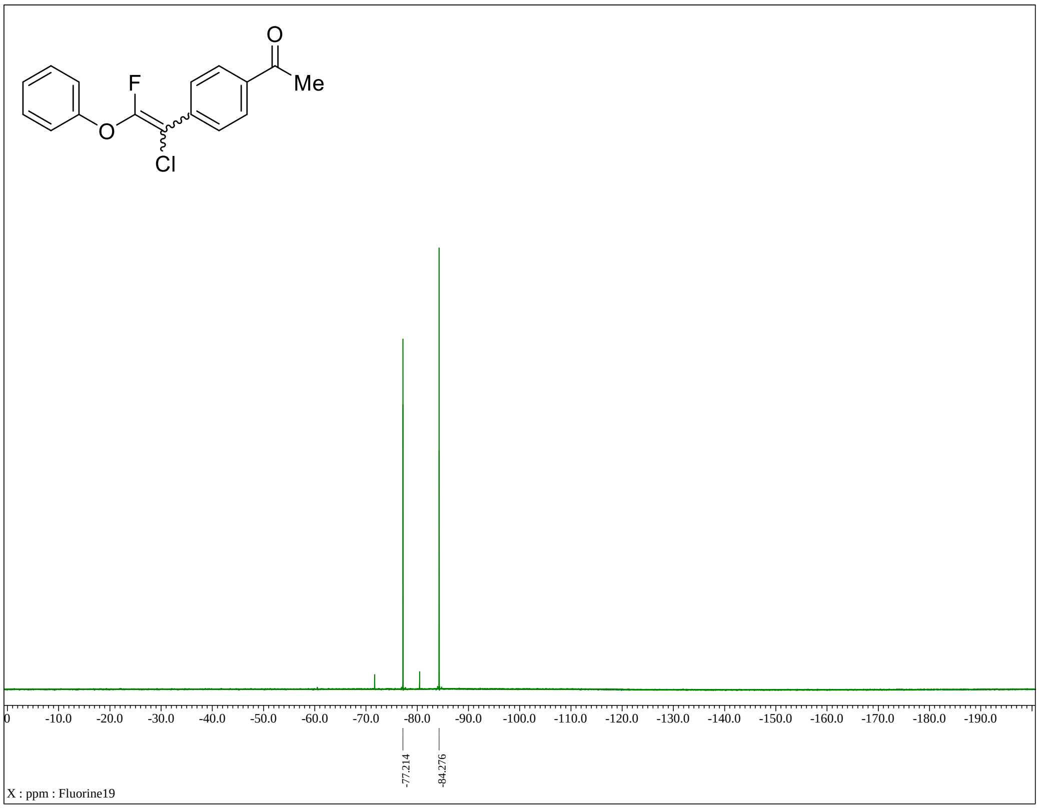

2-Chloro-1-fluoro-2-(4-methoxycarbonylphenyl)ethenyl phenyl ether (2h)

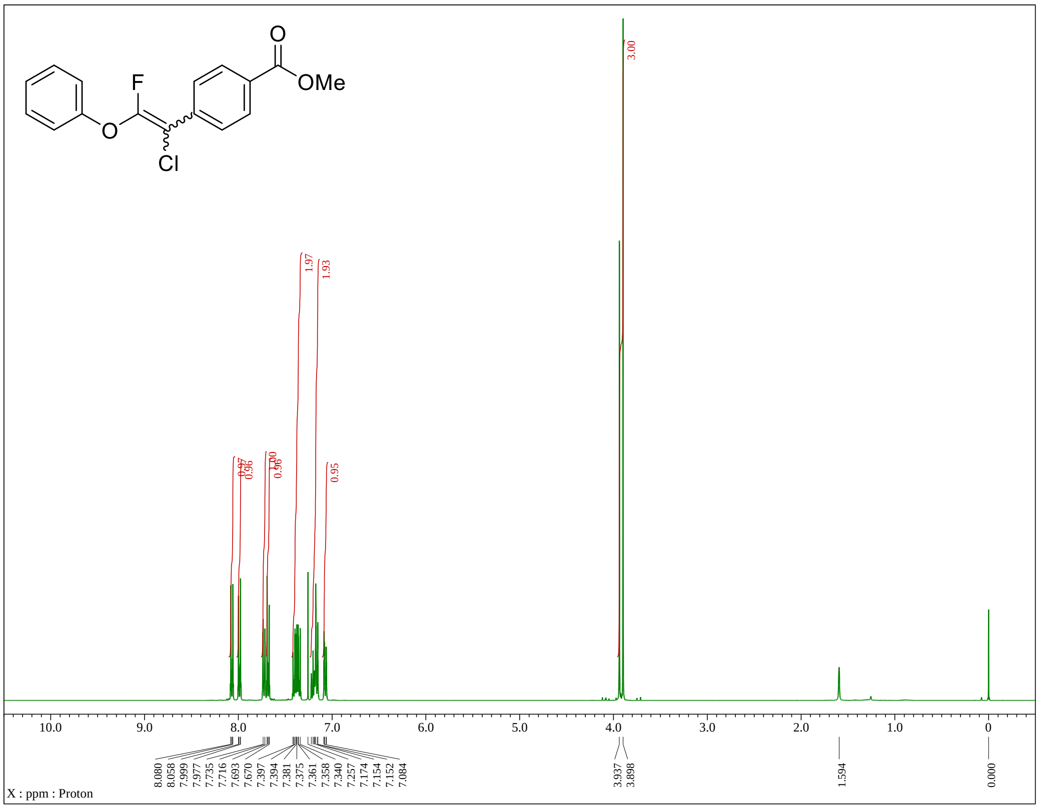

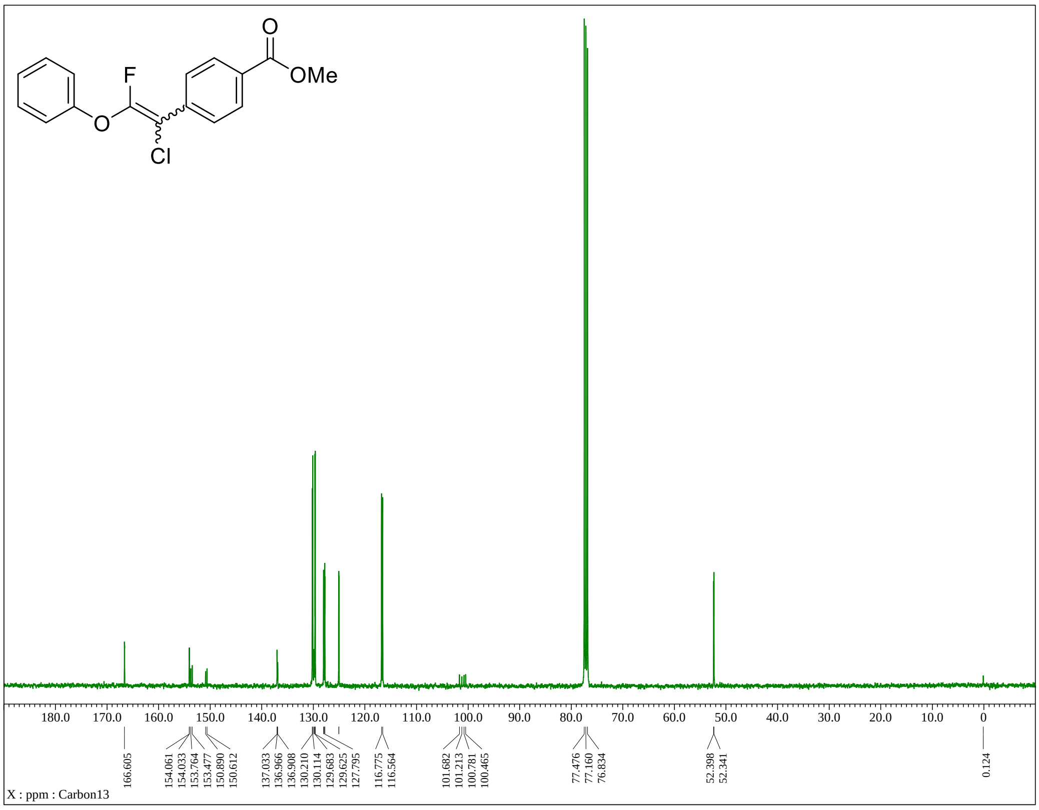

F
O
OMe
O
Cl
180.0
170.0
160.0
150.0
140.0
130.0
120.0
110.0
100.0
90.0
80.0
70.0
60.0
50.0
40.0
30.0
20.0
10.0
0
166.605
154.061
154.033
153.764
153.477
150.890
150.612
137.033
136.966
136.908
130.210
130.114
129.683
129.625
127.795
116.775
116.564
101.682
101.213
100.781
100.465
77.476
77.160
76.834
52.398
52.341
0.124
X : ppm : Carbon13

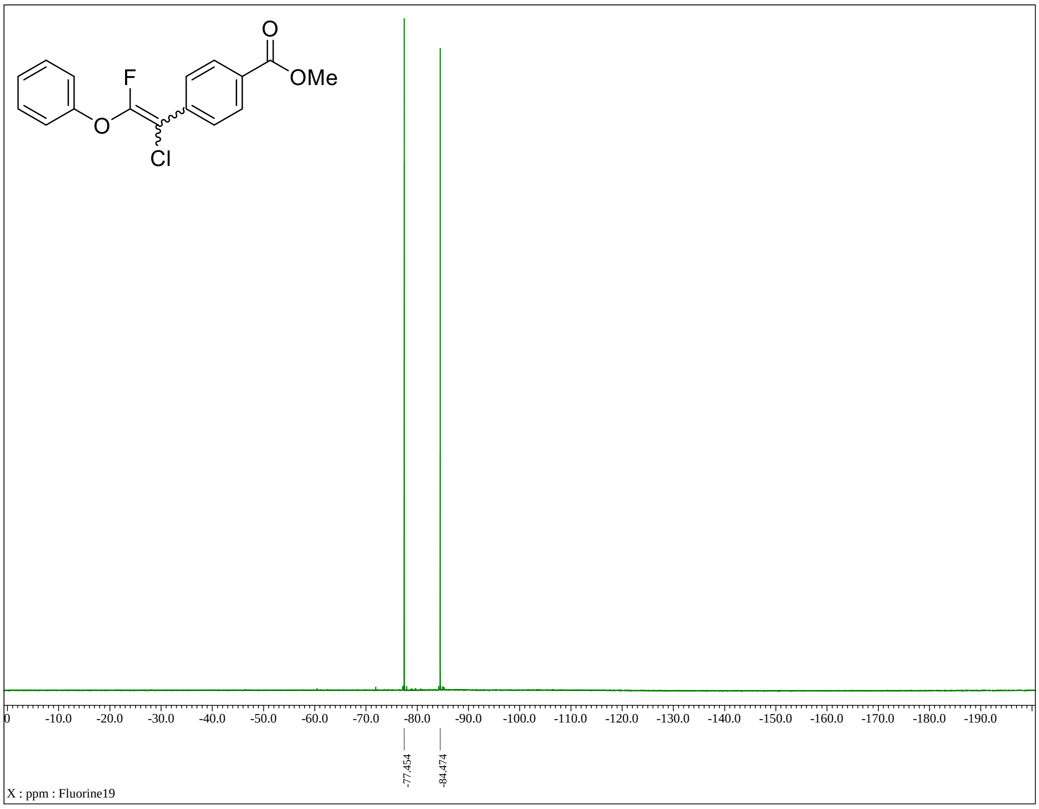

F
O
OMe
O
Cl
0
-10.0
-20.0
-30.0
-40.0
-50.0
-60.0
-70.0
-80.0
-90.0
-100.0
-110.0
-120.0
-130.0
-140.0
-150.0
-160.0
-170.0
-180.0
-190.0
-77.454
-84.474
X : ppm : Fluorine19

2-Chloro-1-fluoro-2-(4-formylphenyl)ethenyl phenyl ether (2i)

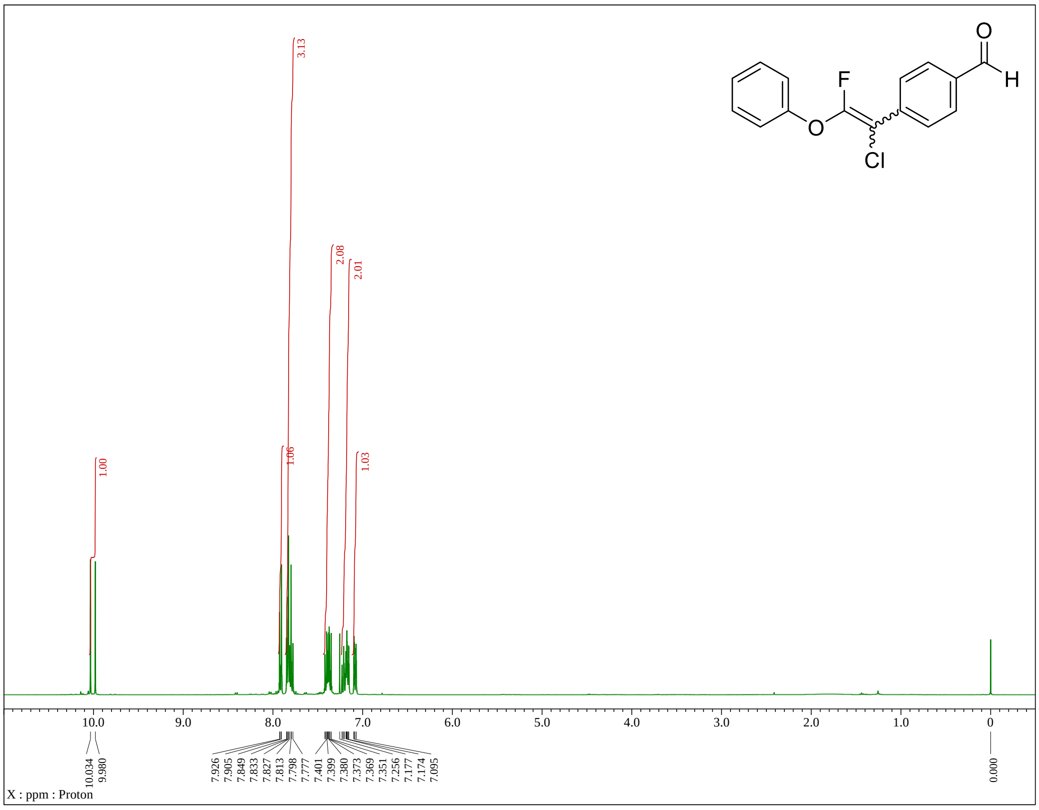

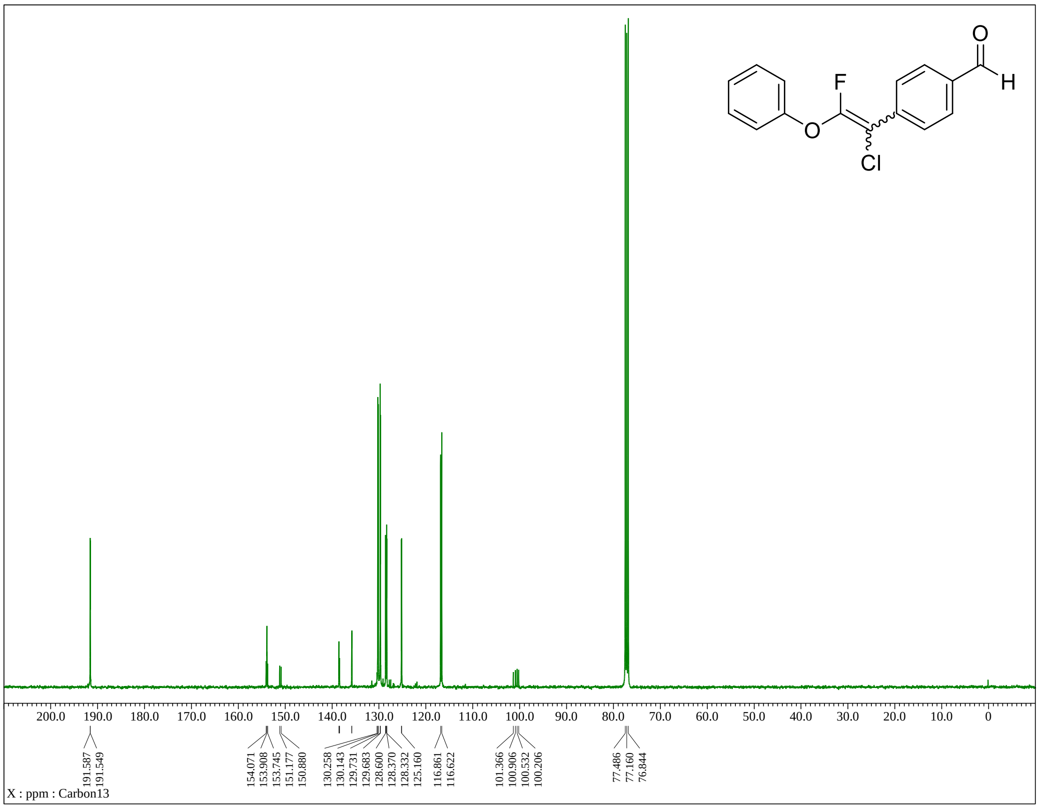

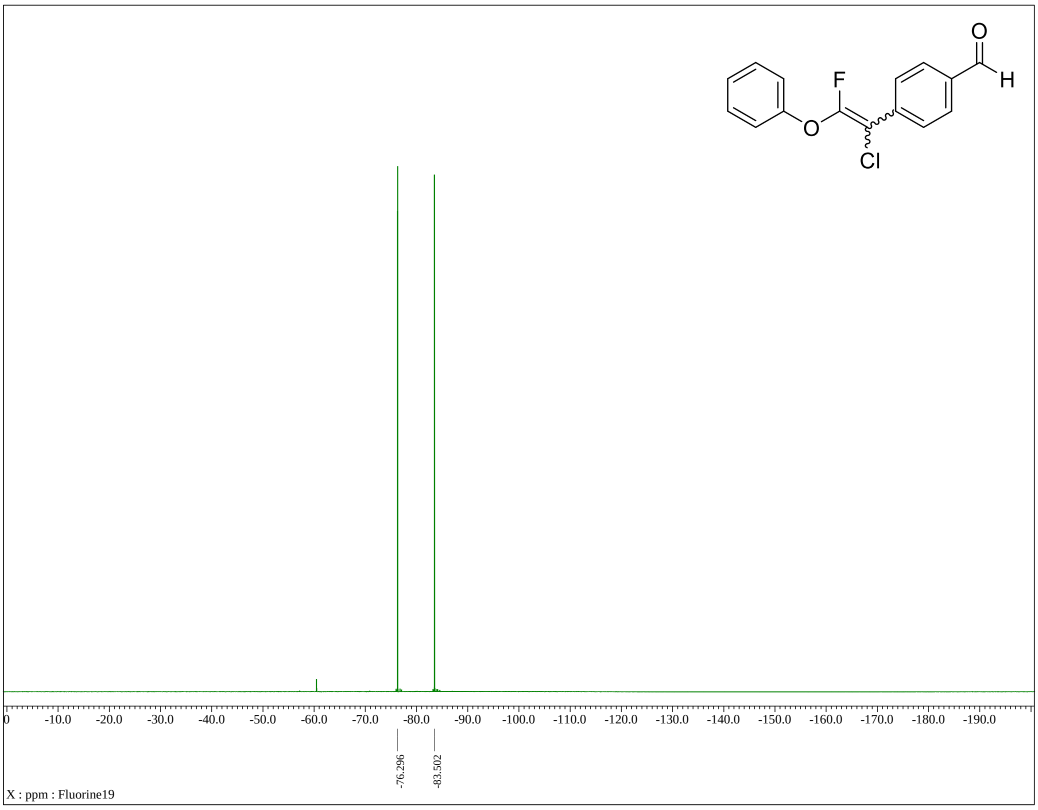

2-Chloro-1-fluoro-2-(3-nitrophenyl)ethenyl phenyl ether (2j)

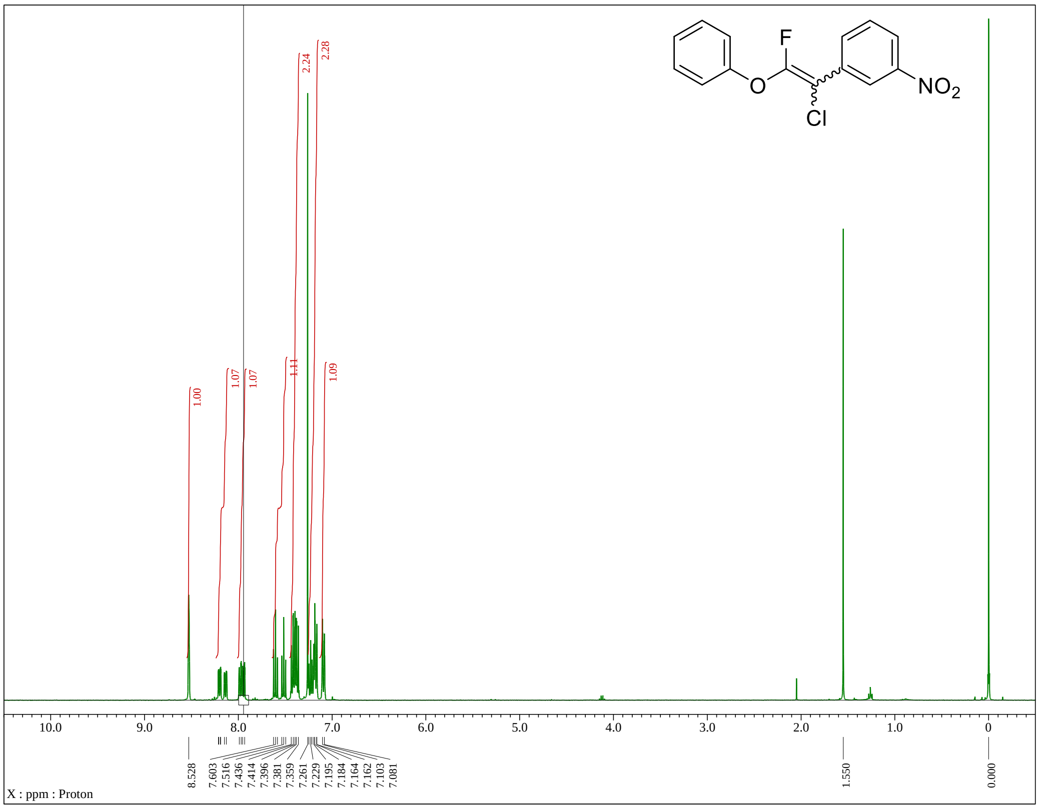

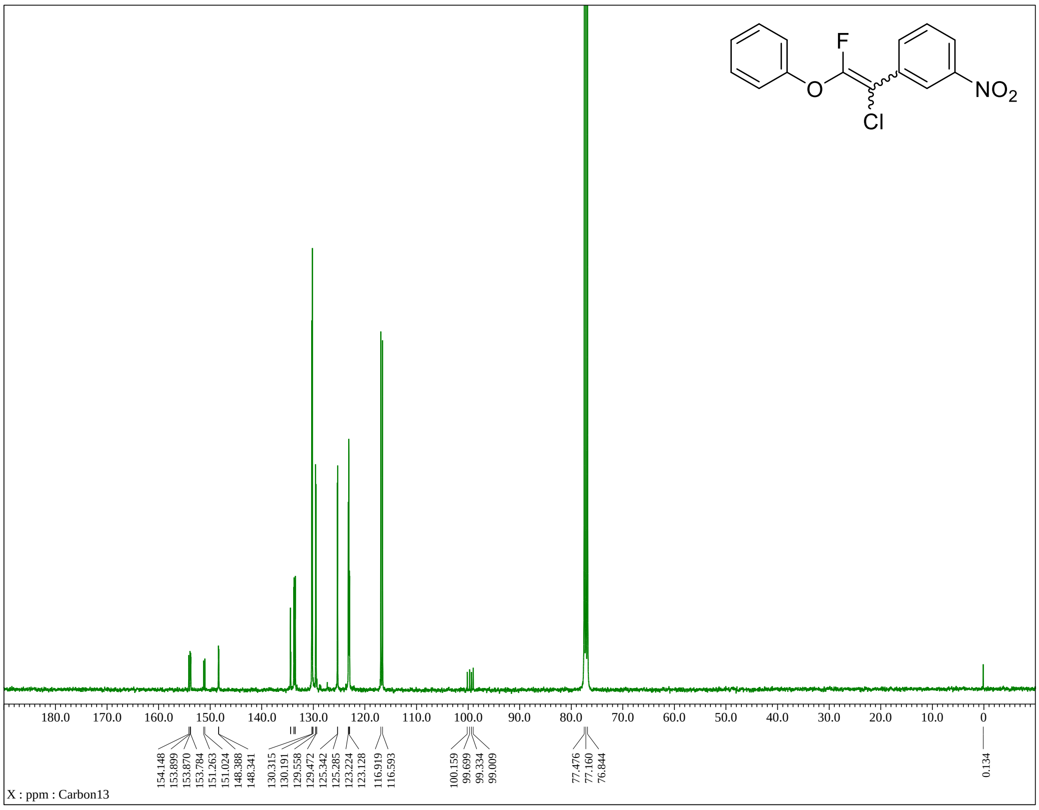
F
O
Cl
NO2
180.0 170.0 160.0 150.0 140.0 130.0 120.0 110.0 100.0 90.0 80.0 70.0 60.0 50.0 40.0 30.0 20.0 10.0 0
154.148
153.899
153.870
153.784
151.263
151.024
148.388
148.341
130.315
130.191
129.558
129.472
125.342
125.285
123.224
123.128
116.919
116.593
100.159
99.699
99.334
99.009
77.476
77.160
76.844
0.134
X : ppm : Carbon13

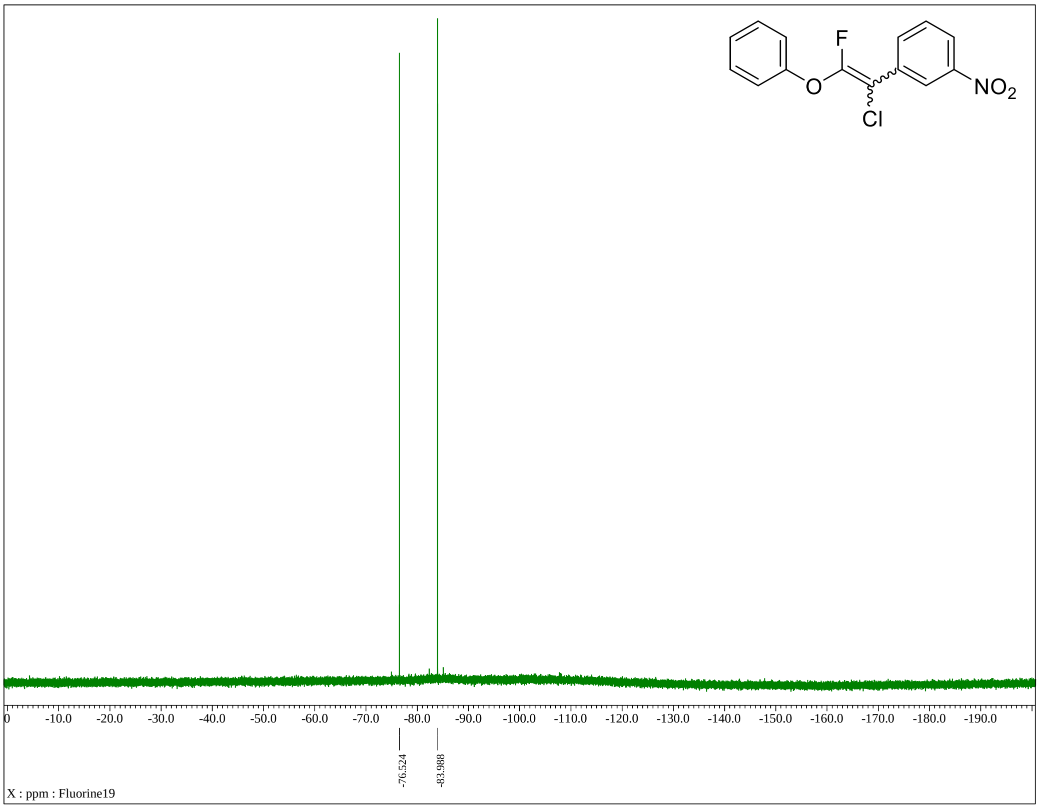
F
O
Cl
NO2
0 -10.0 -20.0 -30.0 -40.0 -50.0 -60.0 -70.0 -80.0 -90.0 -100.0 -110.0 -120.0 -130.0 -140.0 -150.0 -160.0 -170.0 -180.0 -190.0
-76.524
-83.988
X : ppm : Fluorine19

2-Chloro-1-fluoro-2-(4-hydroxyphenyl)ethenyl phenyl ether (2k)

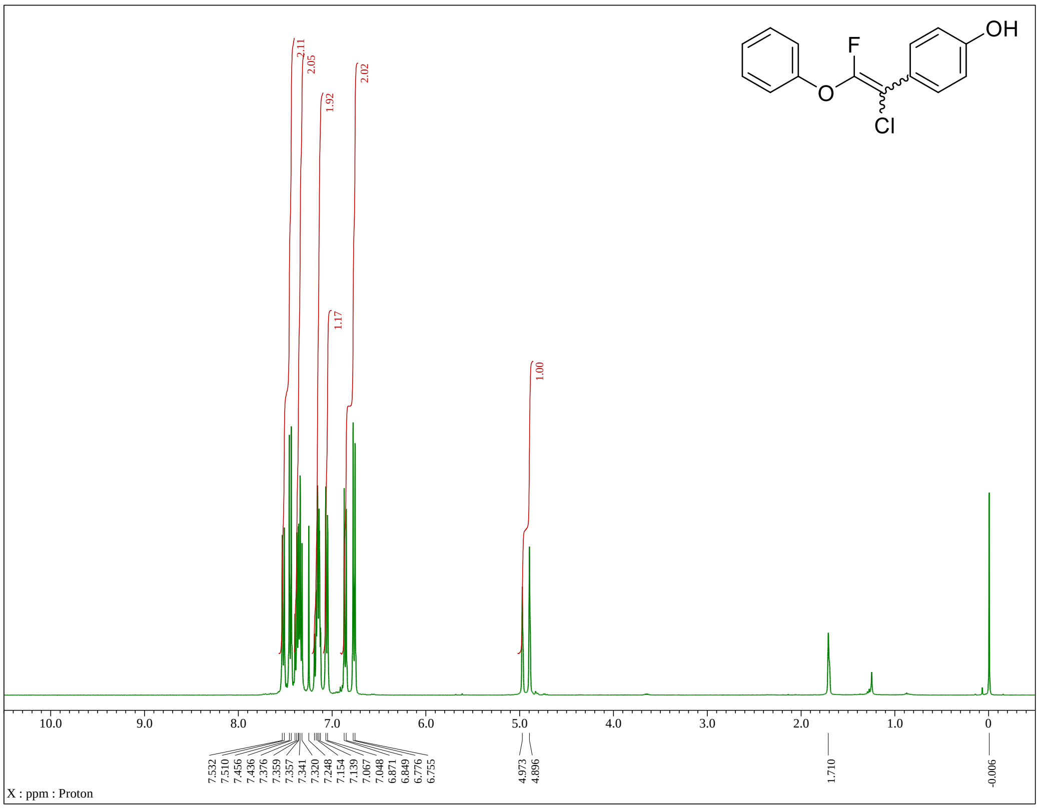

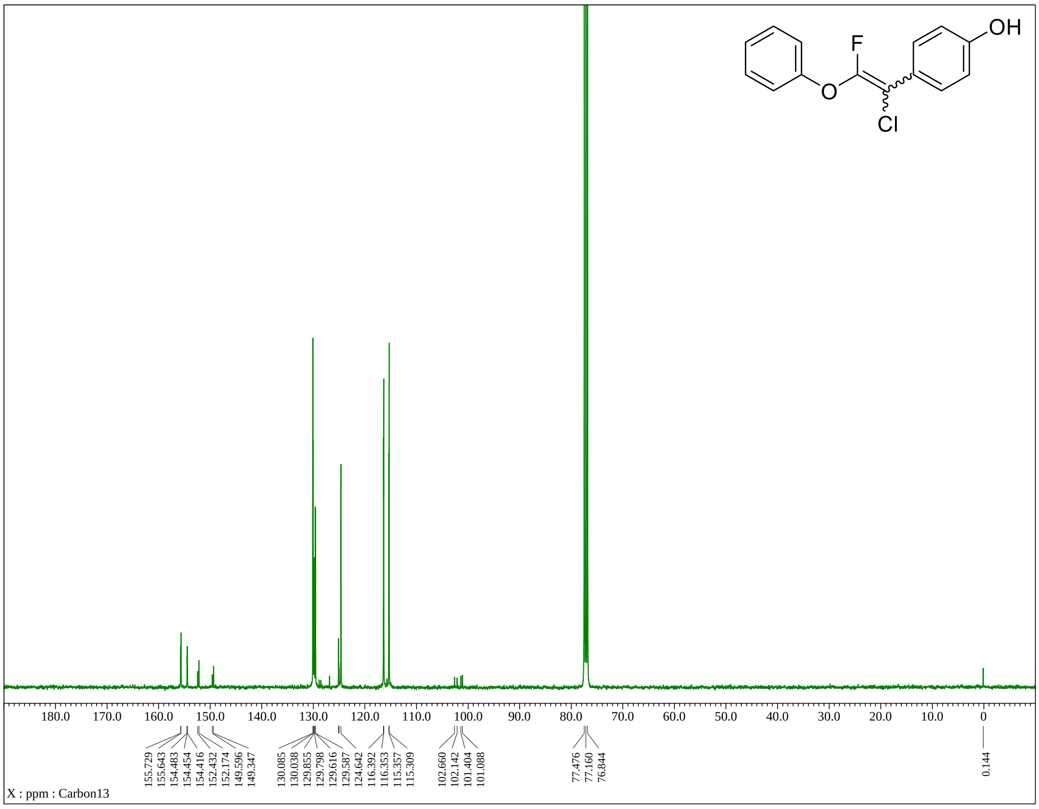

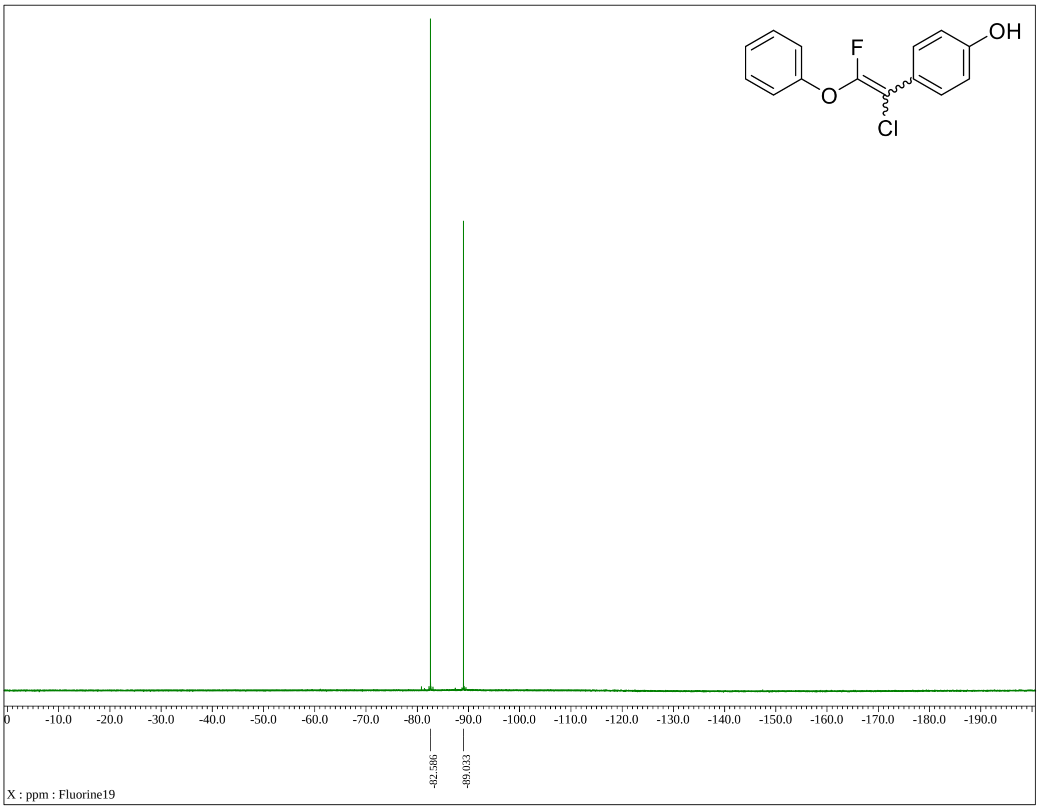

2-(3-Aminophenyl)-2-chloro-1-fluoroethenyl phenyl ether (2l)

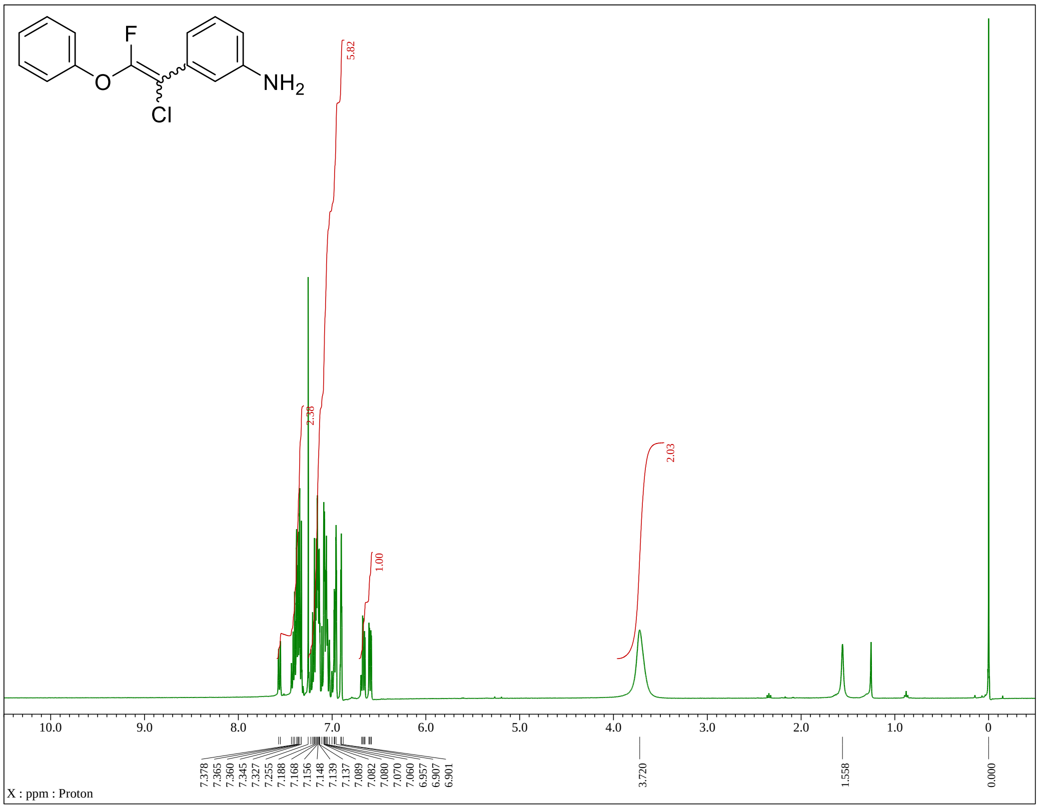

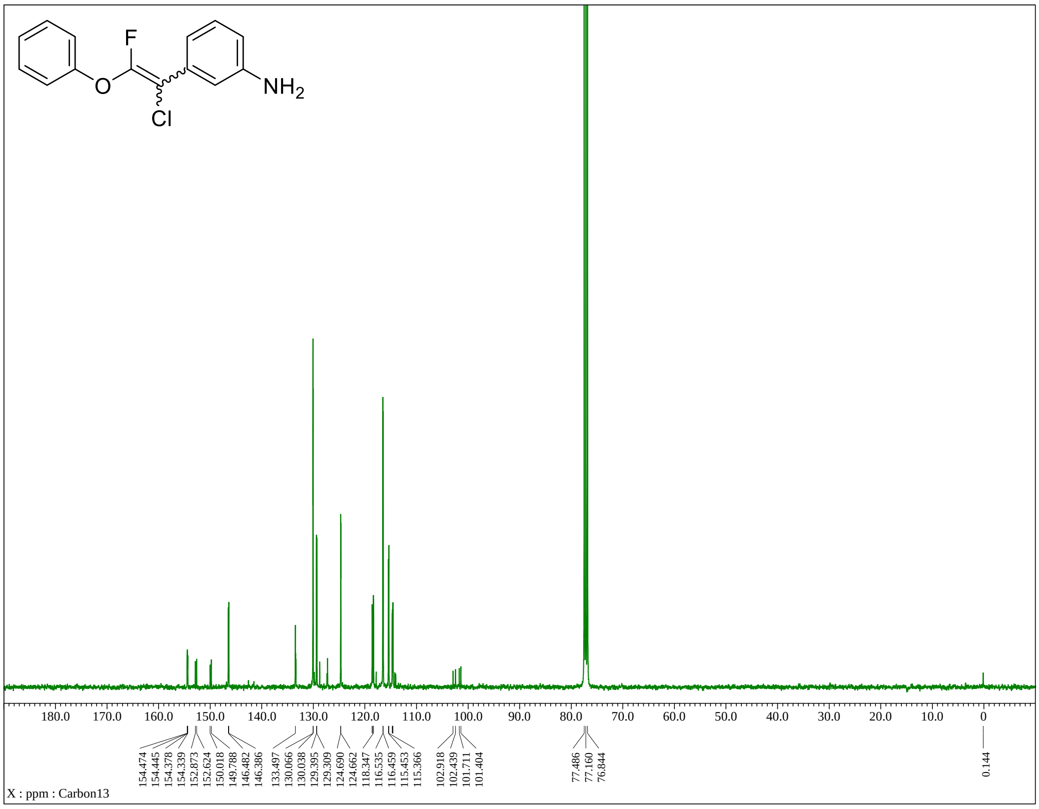

F
O
Cl
NH2
180.0 170.0 160.0 150.0 140.0 130.0 120.0 110.0 100.0 90.0 80.0 70.0 60.0 50.0 40.0 30.0 20.0 10.0 0
154.474 154.445 154.378 154.339 152.873 152.624 150.018 149.788 146.482 146.386 133.497 130.066 130.038 129.395 129.309 124.690 124.662 118.347 116.535 116.459 115.453 115.366 102.918 102.439 101.711 101.404
77.486 77.160 76.844
0.144
X : ppm : Carbon13

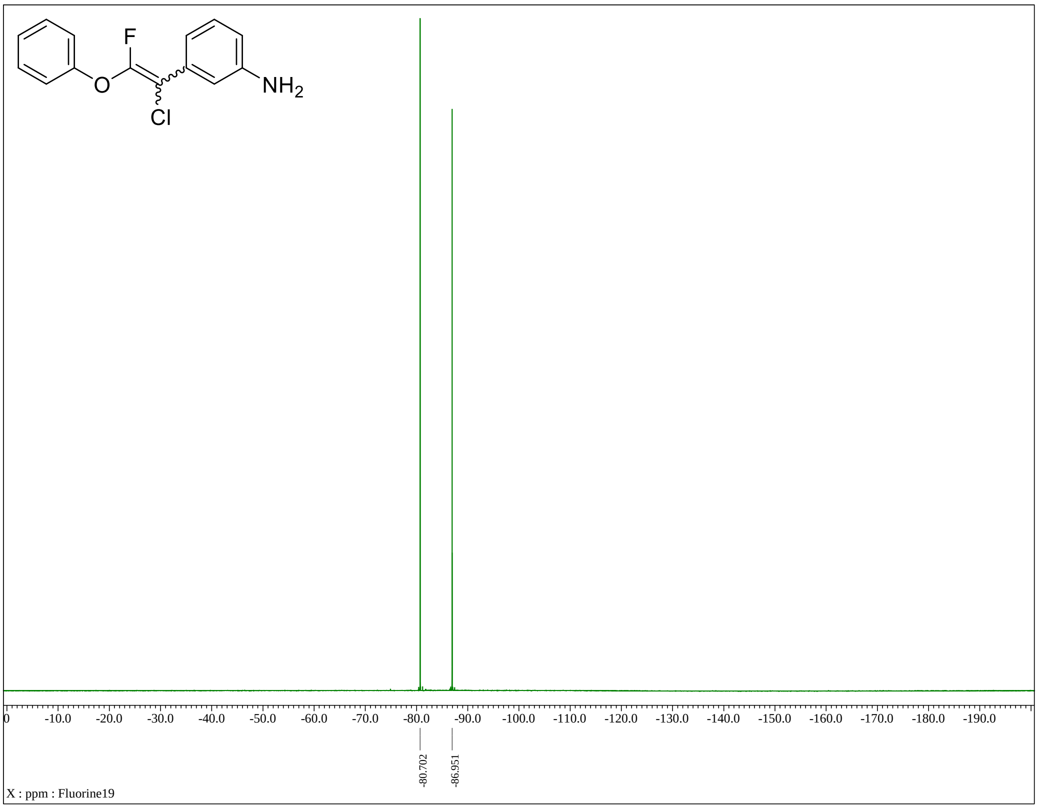

F
O
Cl
NH2
0 -10.0 -20.0 -30.0 -40.0 -50.0 -60.0 -70.0 -80.0 -90.0 -100.0 -110.0 -120.0 -130.0 -140.0 -150.0 -160.0 -170.0 -180.0 -190.0
-80.702
-86.951
X : ppm : Fluorine19

2-Chloro-2-cyclopropyl-1-fluoroethenyl phenyl ether (2n)

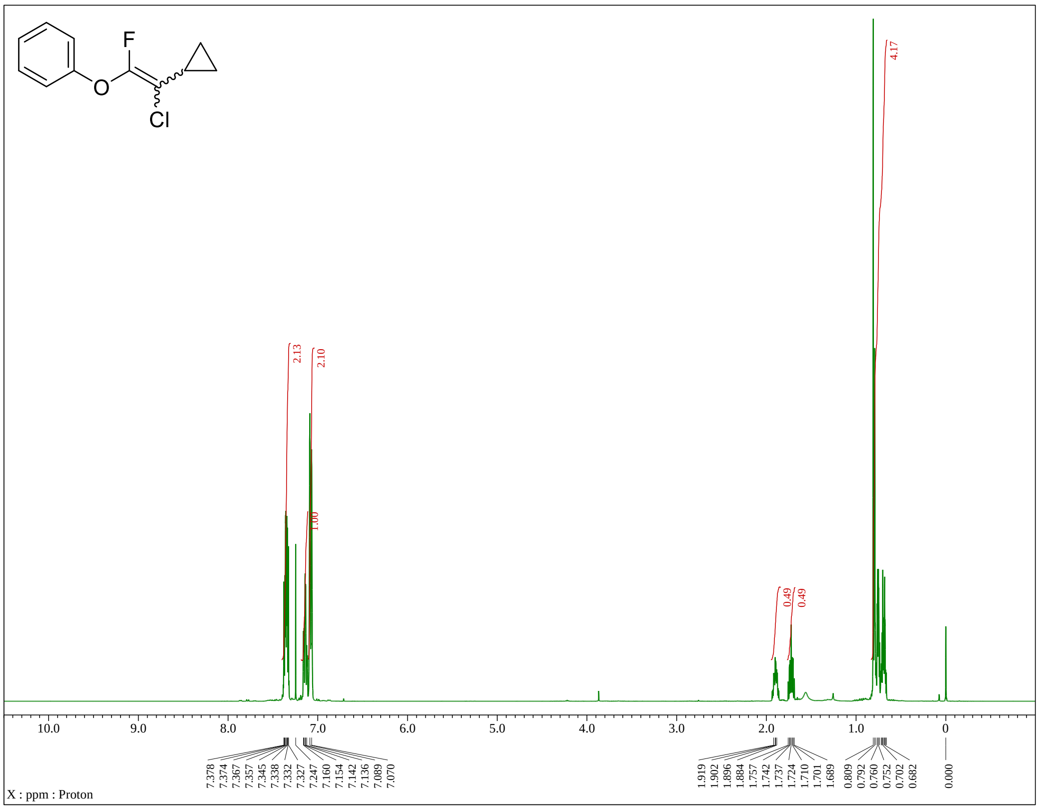

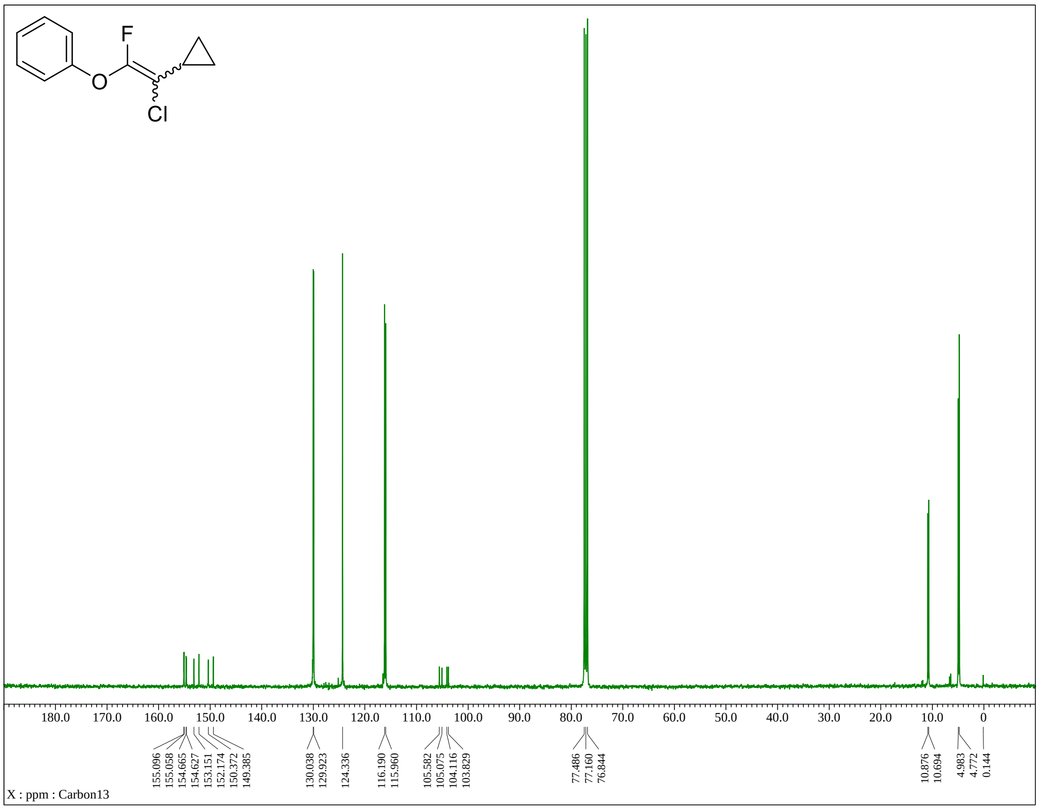

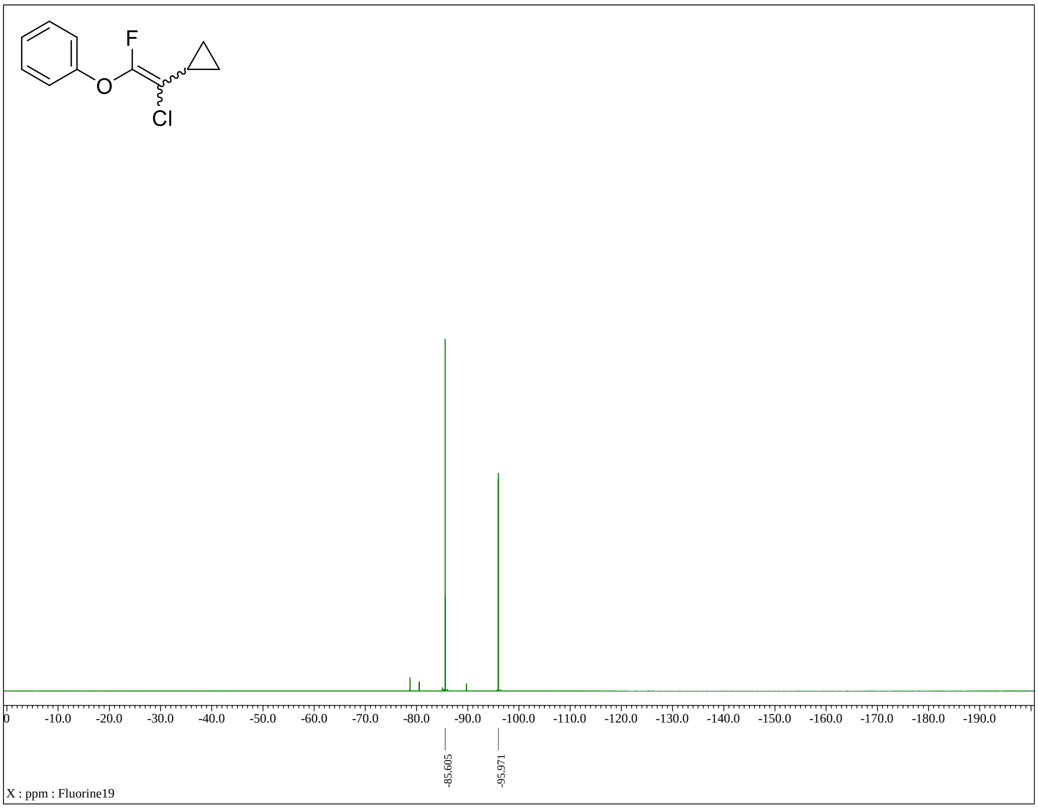

2-Chloro-1-fluoro-2-(thiophene-2-yl)ethenyl phenyl ether (2o)

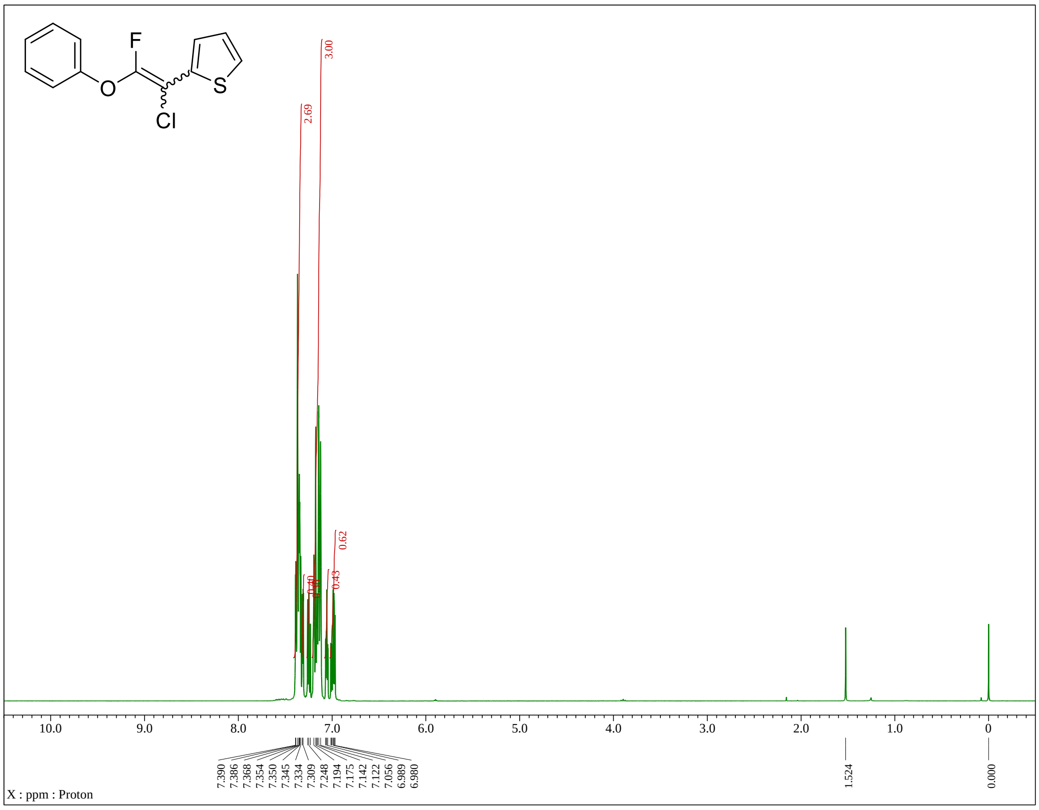

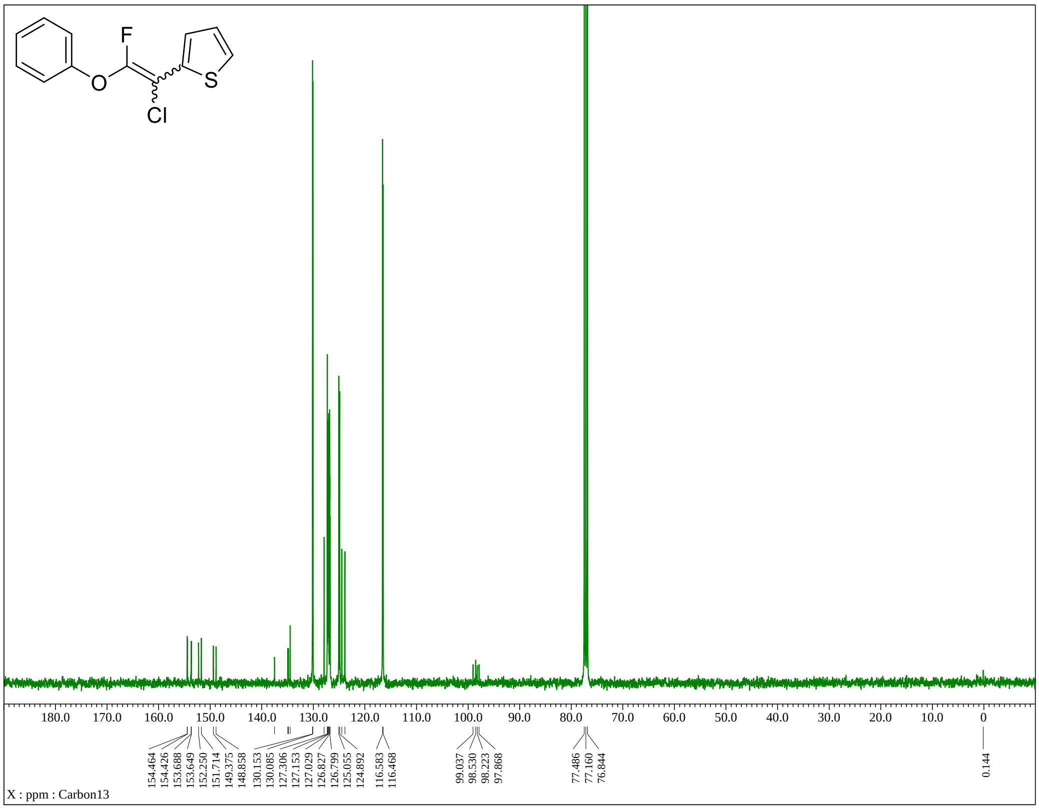
F
O
S
Cl
180.0
170.0
160.0
150.0
140.0
130.0
120.0
110.0
100.0
90.0
80.0
70.0
60.0
50.0
40.0
30.0
20.0
10.0
0
154.464
154.426
153.688
153.649
152.250
151.714
149.375
148.858
130.153
130.085
127.306
127.153
127.029
126.827
126.799
125.055
124.892
116.583
116.468
99.037
98.530
98.223
97.868
77.486
77.160
76.844
0.144
X : ppm : Carbon13

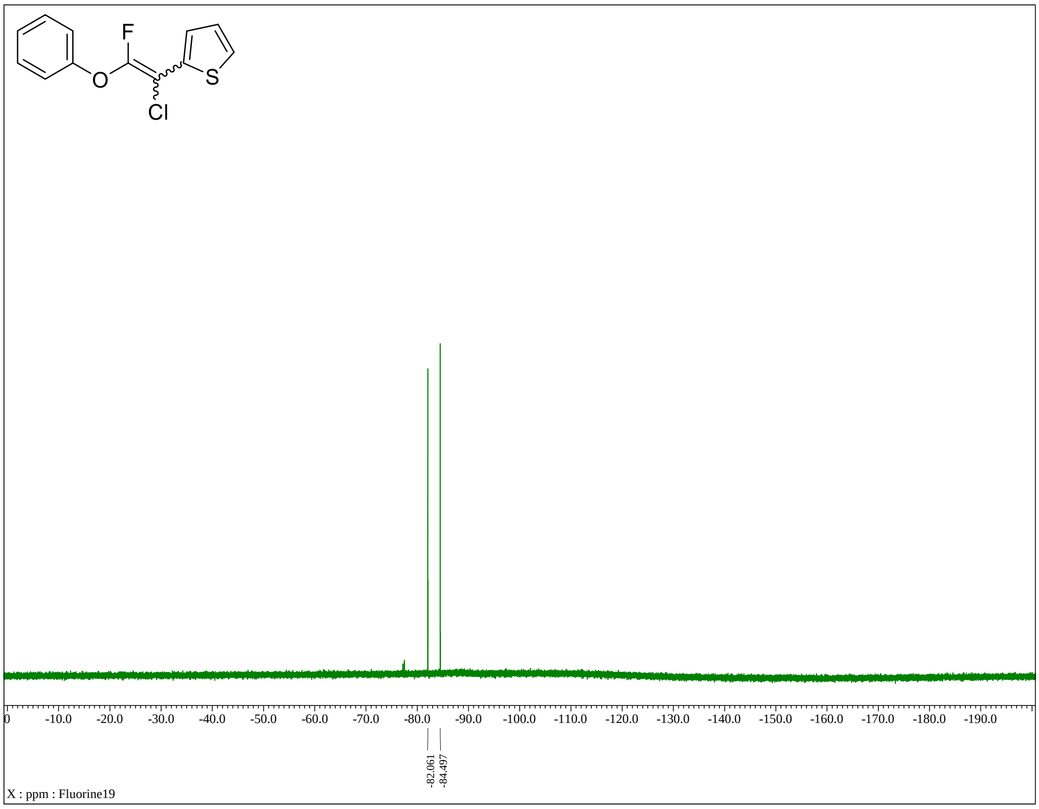
F
O
S
Cl
0
-10.0
-20.0
-30.0
-40.0
-50.0
-60.0
-70.0
-80.0
-90.0
-100.0
-110.0
-120.0
-130.0
-140.0
-150.0
-160.0
-170.0
-180.0
-190.0
-82.061
-84.497
X : ppm : Fluorine19

2-Chloro-1-fluoro-2-phenylethenyl 3-methoxyphenyl ether (2p)

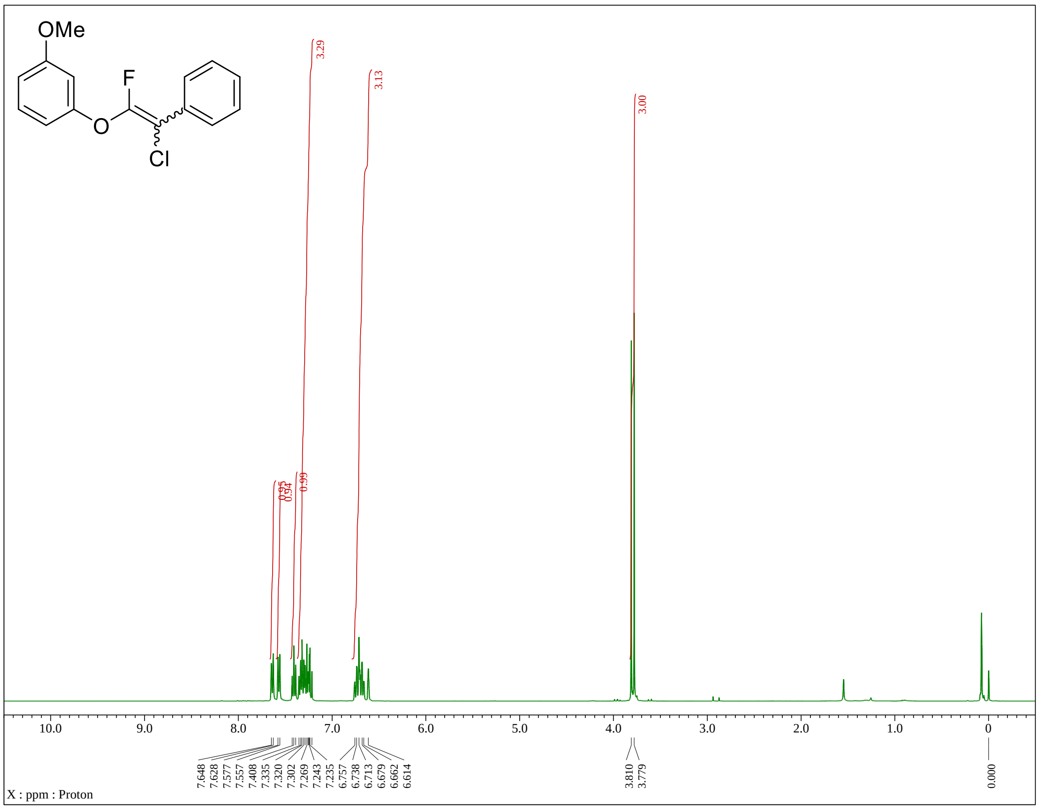

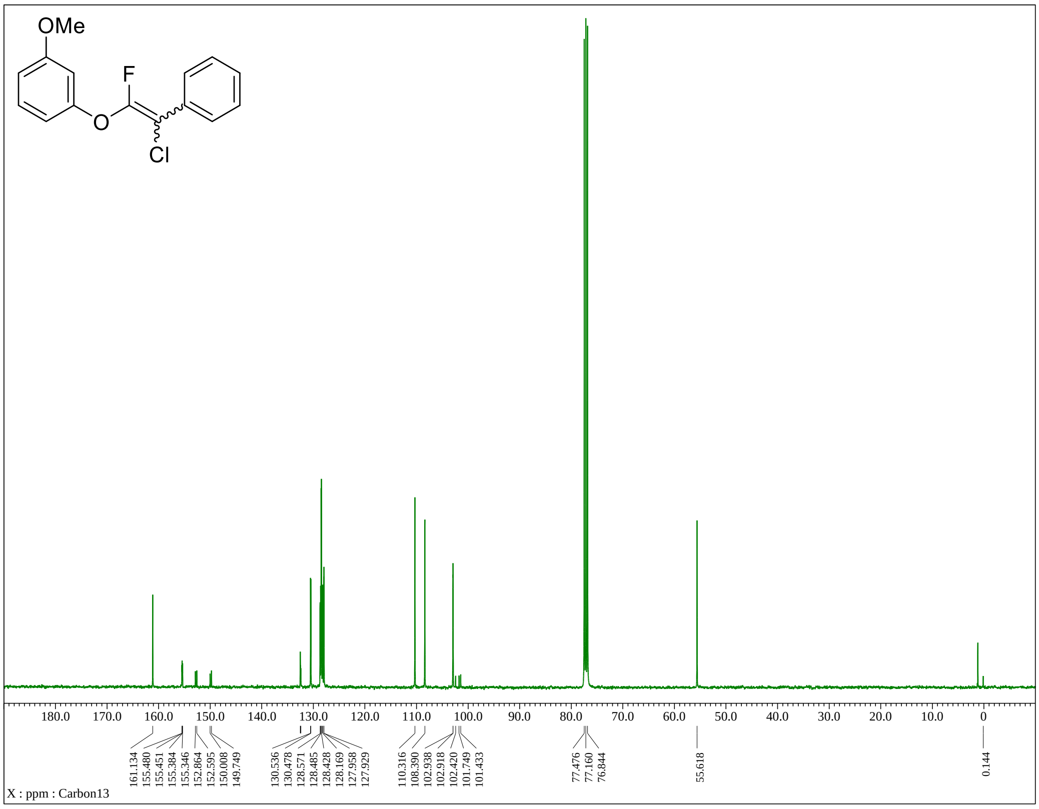

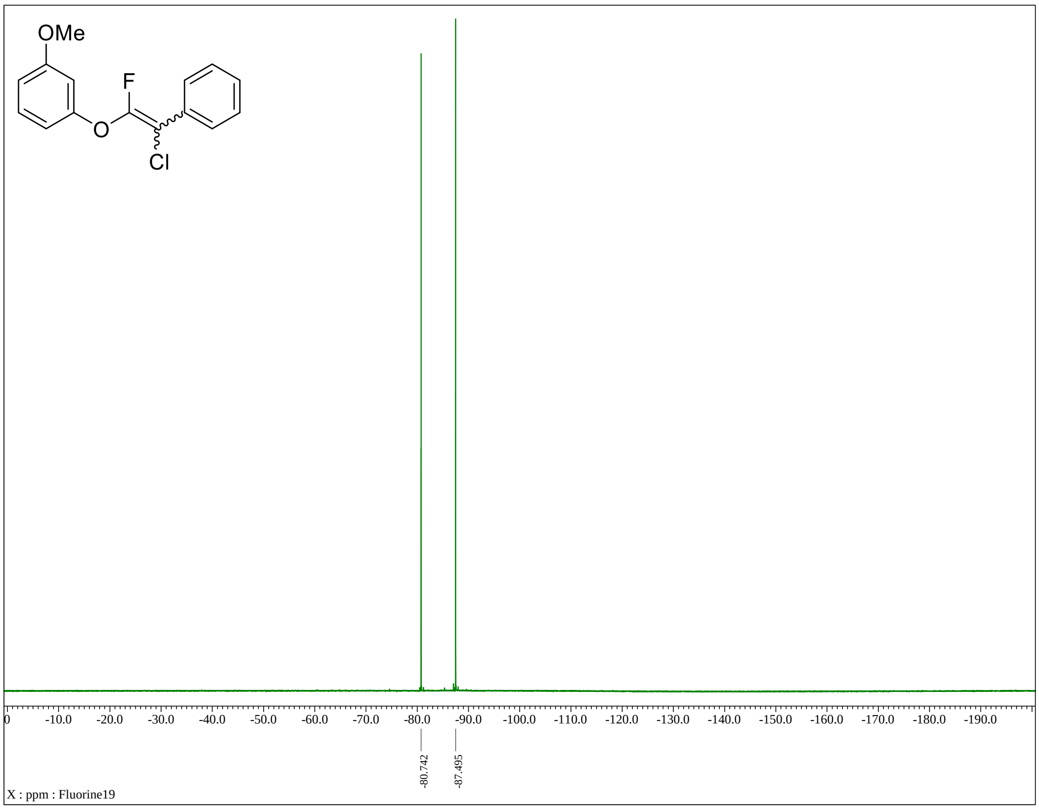

2-Chloro-1-fluoro-2-phenylethenyl 4-nitrophenyl ether (2q)

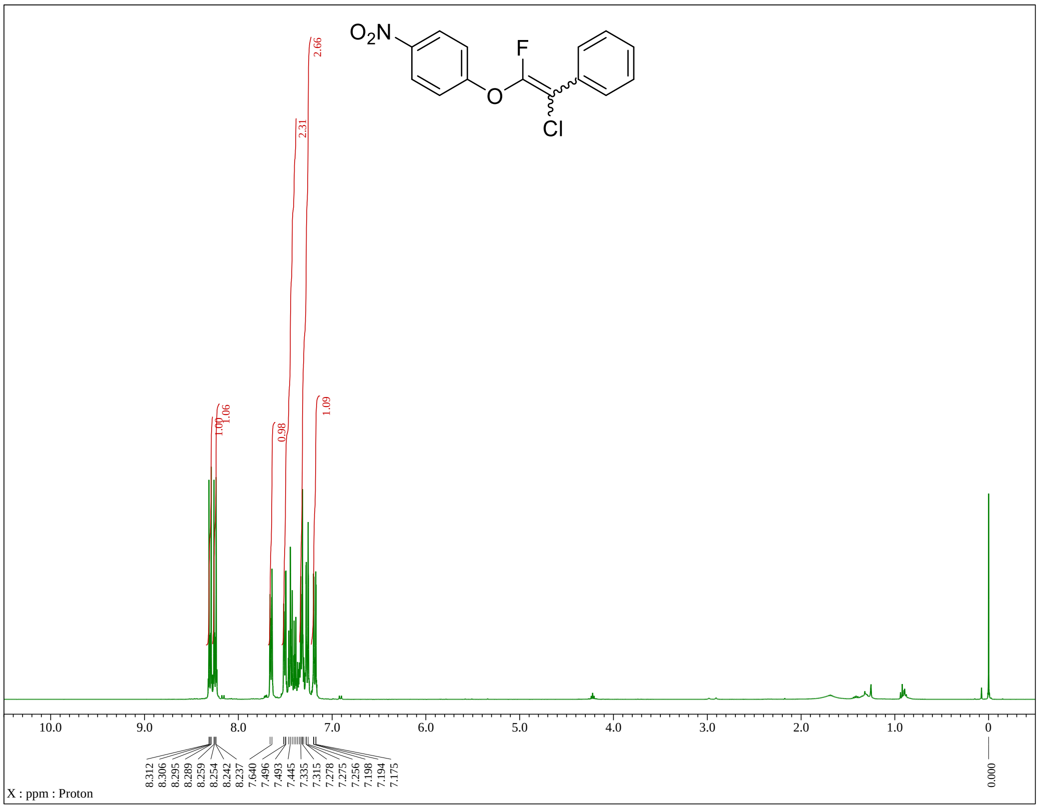

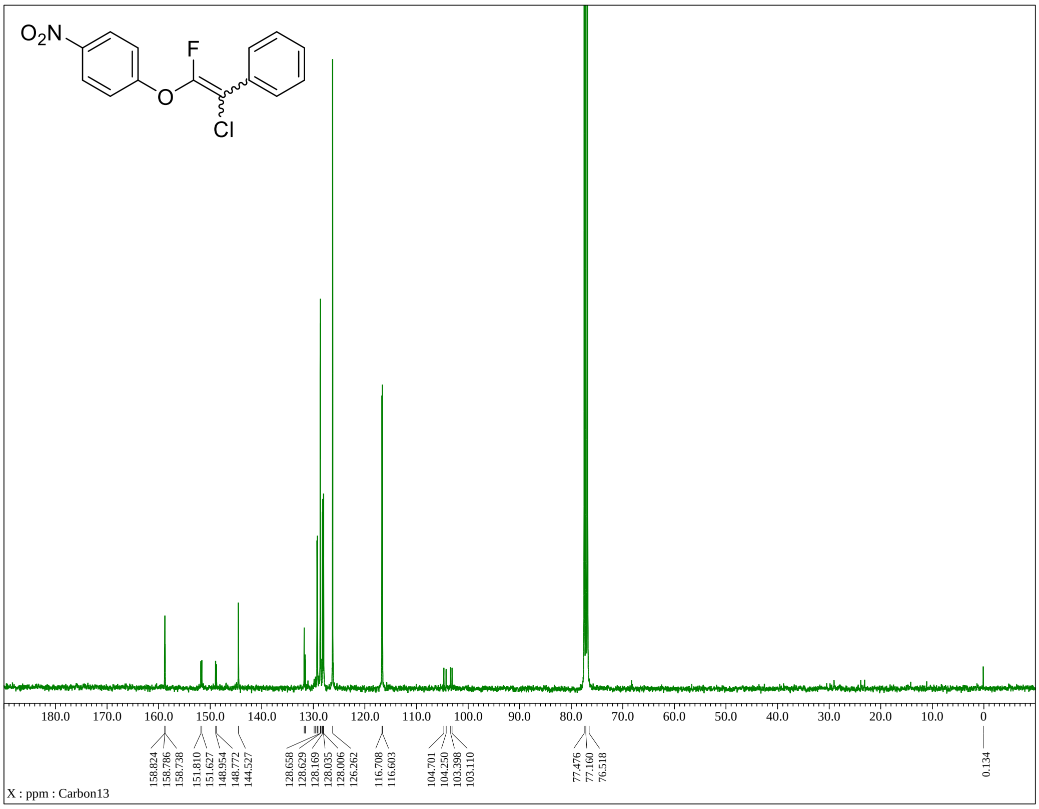
O2N
F
O
Cl
180.0
170.0
160.0
150.0
140.0
130.0
120.0
110.0
100.0
90.0
80.0
70.0
60.0
50.0
40.0
30.0
20.0
10.0
0
158.824
158.786
158.738
151.810
151.627
148.954
148.772
144.527
128.658
128.629
128.169
128.035
128.006
126.262
116.708
116.603
104.701
104.250
103.398
103.110
77.476
77.160
76.518
0.134
X : ppm : Carbon13

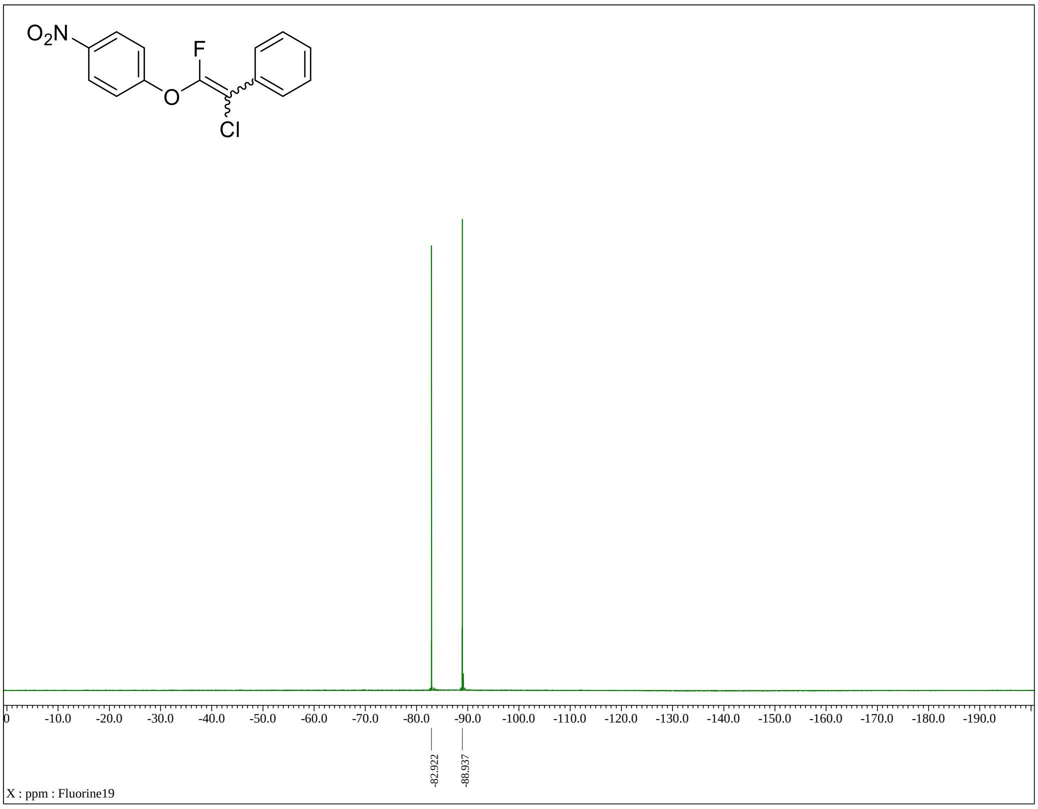
O2N
F
O
Cl
0
-10.0
-20.0
-30.0
-40.0
-50.0
-60.0
-70.0
-80.0
-90.0
-100.0
-110.0
-120.0
-130.0
-140.0
-150.0
-160.0
-170.0
-180.0
-190.0
-82.922
-88.937
X : ppm : Fluorine19

<u>**2-Chloro-1-fluoro-2-phenylethenyl 4-ethoxycarbonylphenyl ether (2r)**</u>

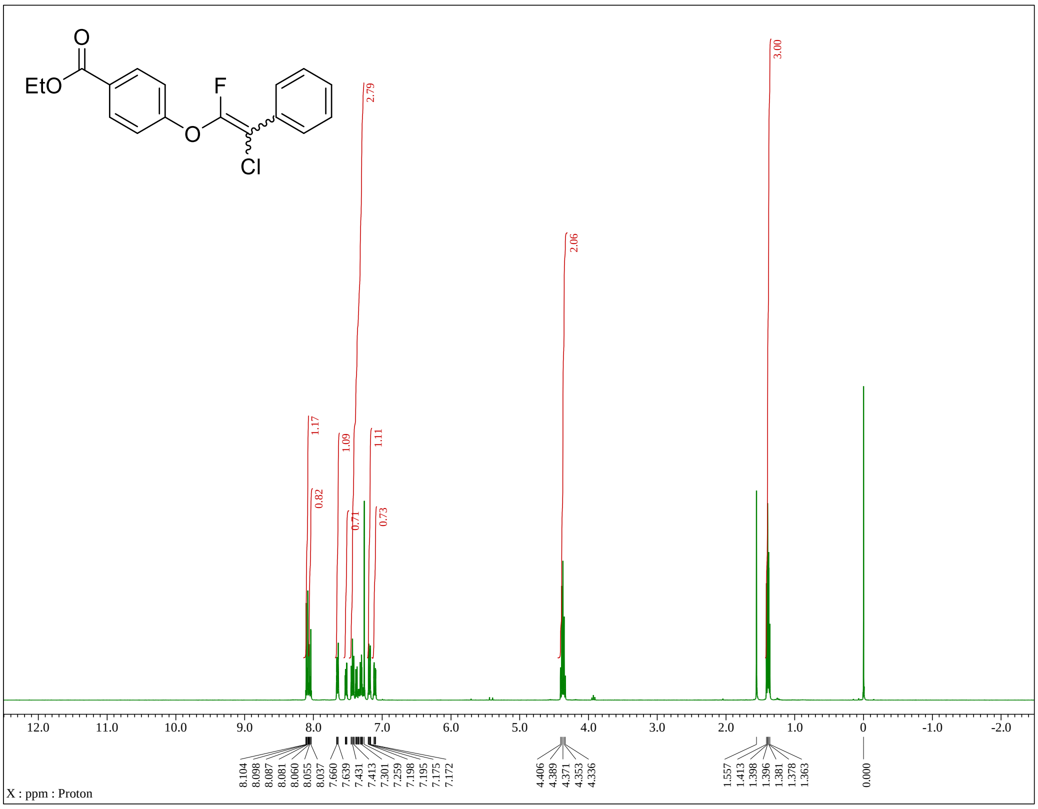

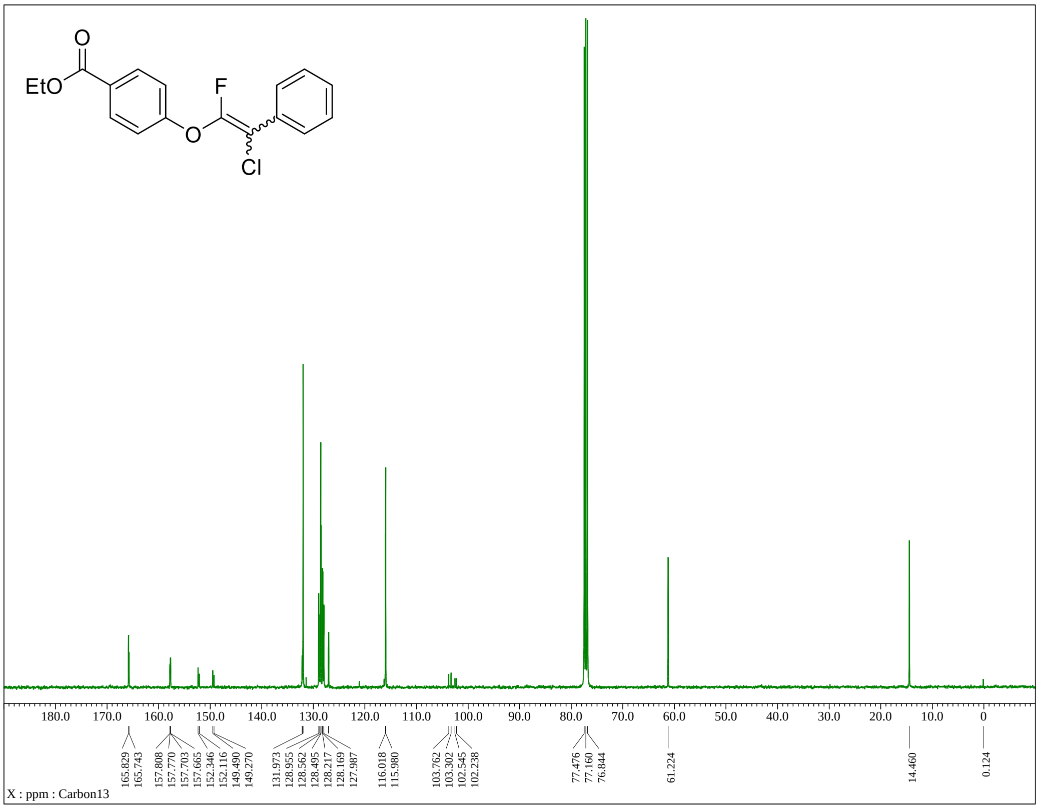

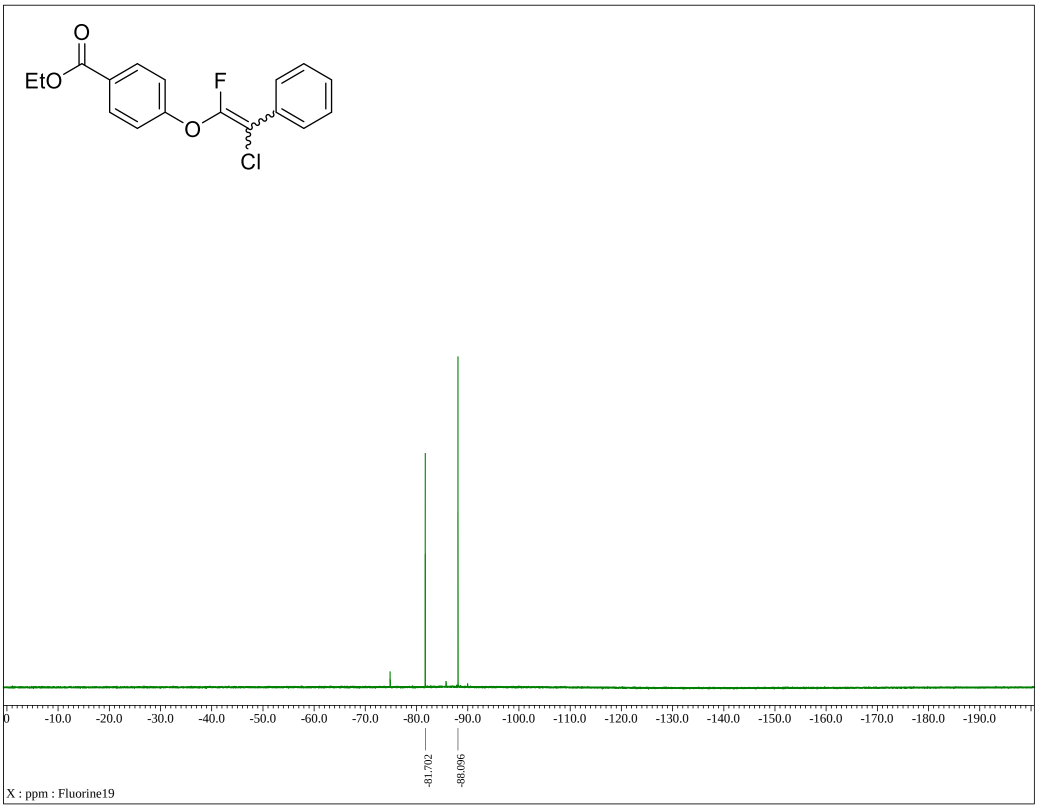

3-Aminophenyl 2-chloro-1-fluoro-2-phenylethenyl ether (2s)

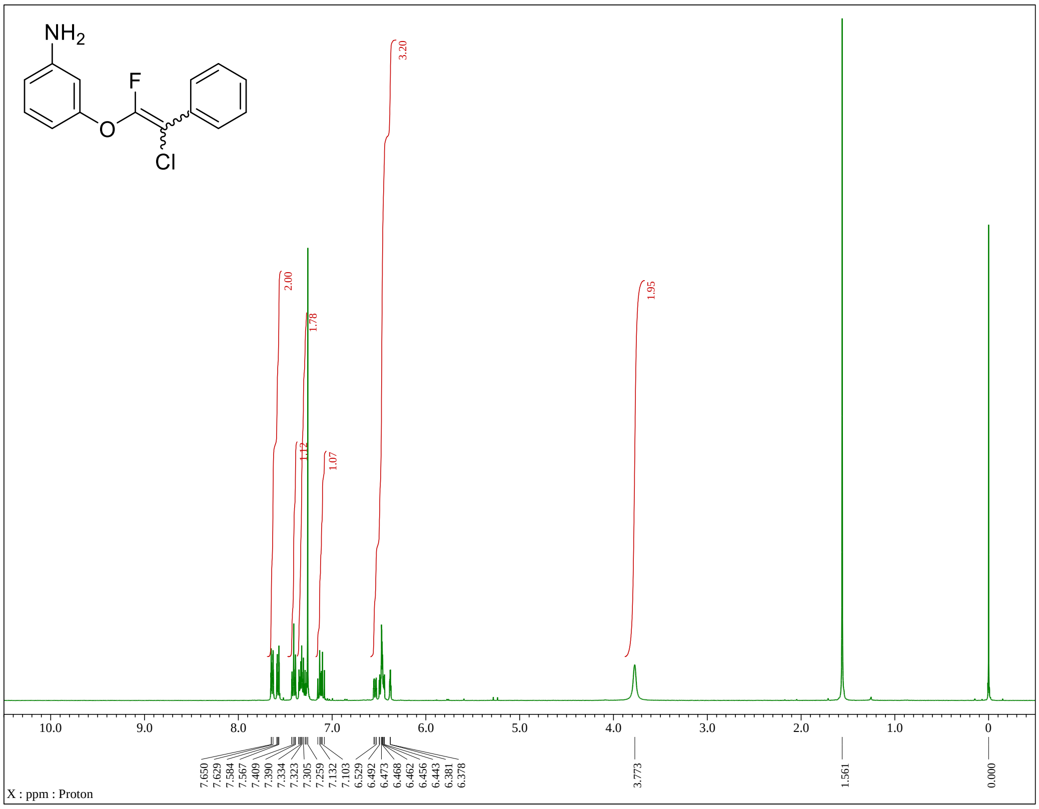

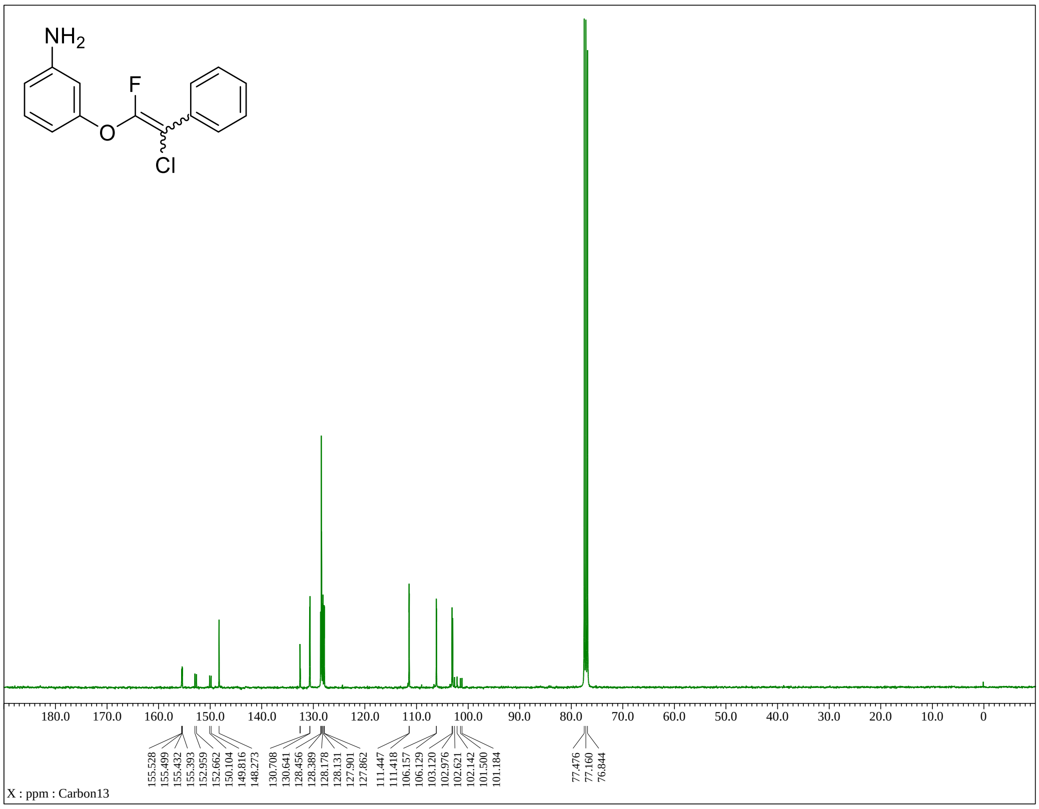
NH2
F
O
Cl
180.0 170.0 160.0 150.0 140.0 130.0 120.0 110.0 100.0 90.0 80.0 70.0 60.0 50.0 40.0 30.0 20.0 10.0 0
155.528 155.499 155.432 155.393 152.959 152.662 150.104 149.816 148.273 130.708 130.641 128.456 128.389 128.178 128.131 127.901 127.862 111.447 111.418 106.157 106.129 103.120 102.976 102.621 102.142 101.500 101.184
77.476 77.160 76.844
X : ppm : Carbon13

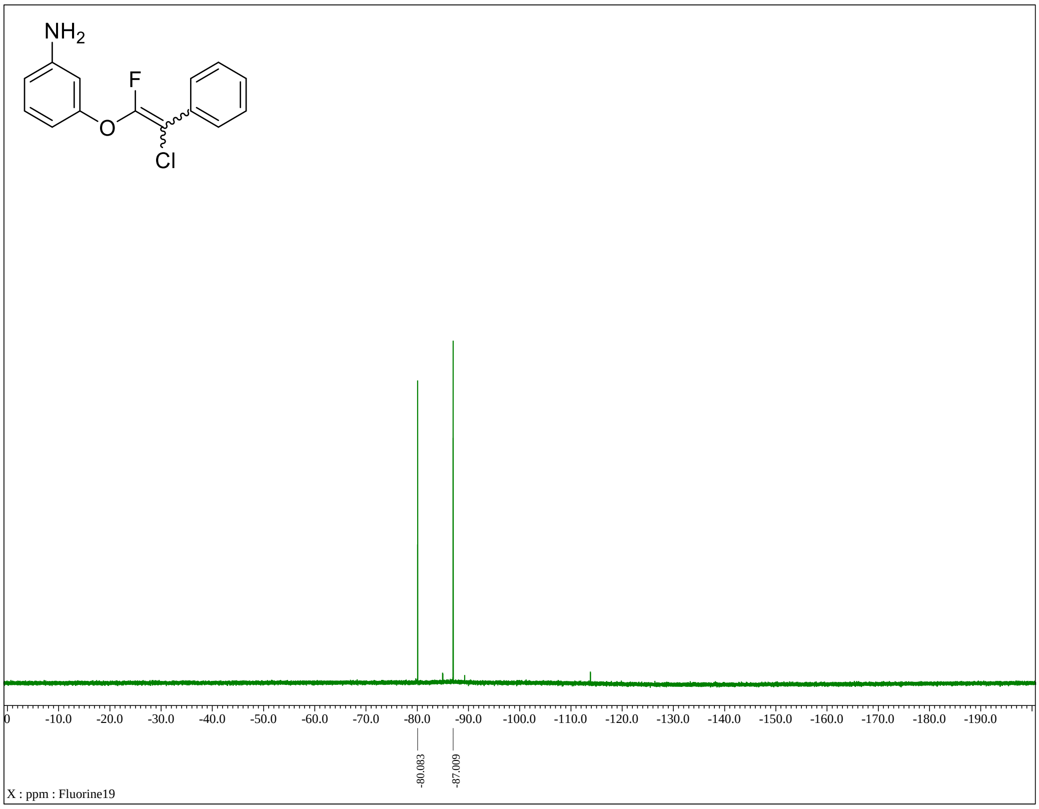
NH2
F
O
Cl
0 -10.0 -20.0 -30.0 -40.0 -50.0 -60.0 -70.0 -80.0 -90.0 -100.0 -110.0 -120.0 -130.0 -140.0 -150.0 -160.0 -170.0 -180.0 -190.0
-80.083
-87.009
X : ppm : Fluorine19

(3-Chloro-4-fluoro-4-phenoxybut-3-en-1-yn-1-yl)trimethylsilane (3a)

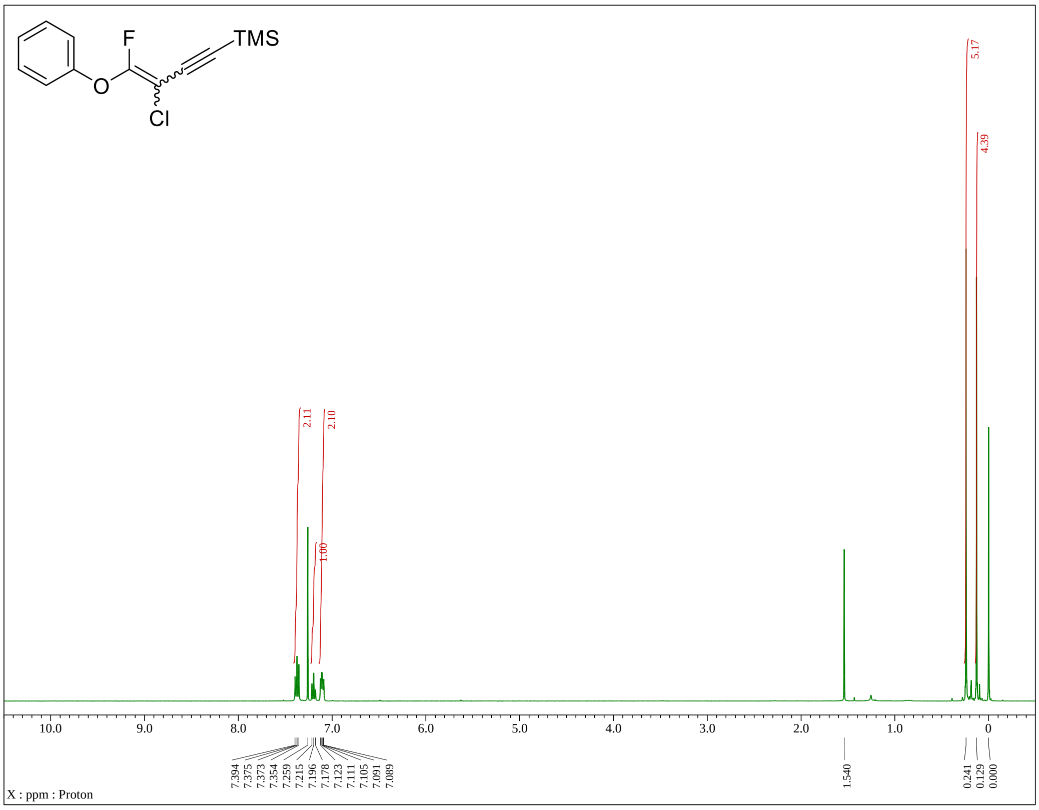

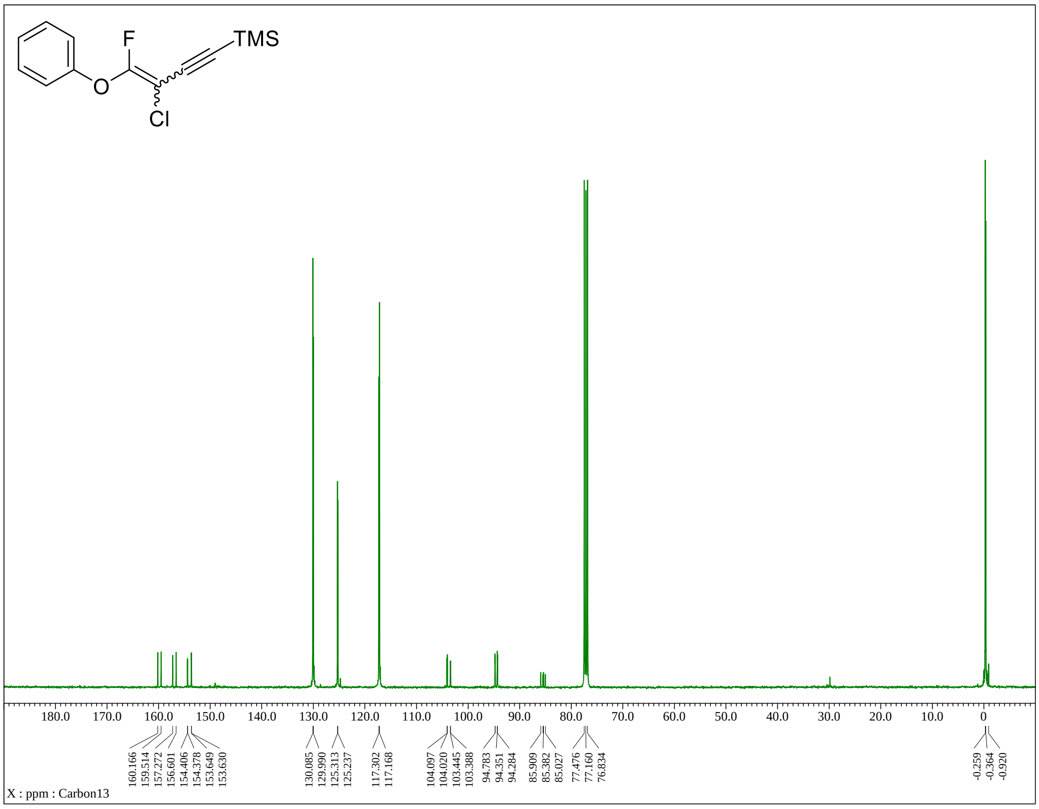

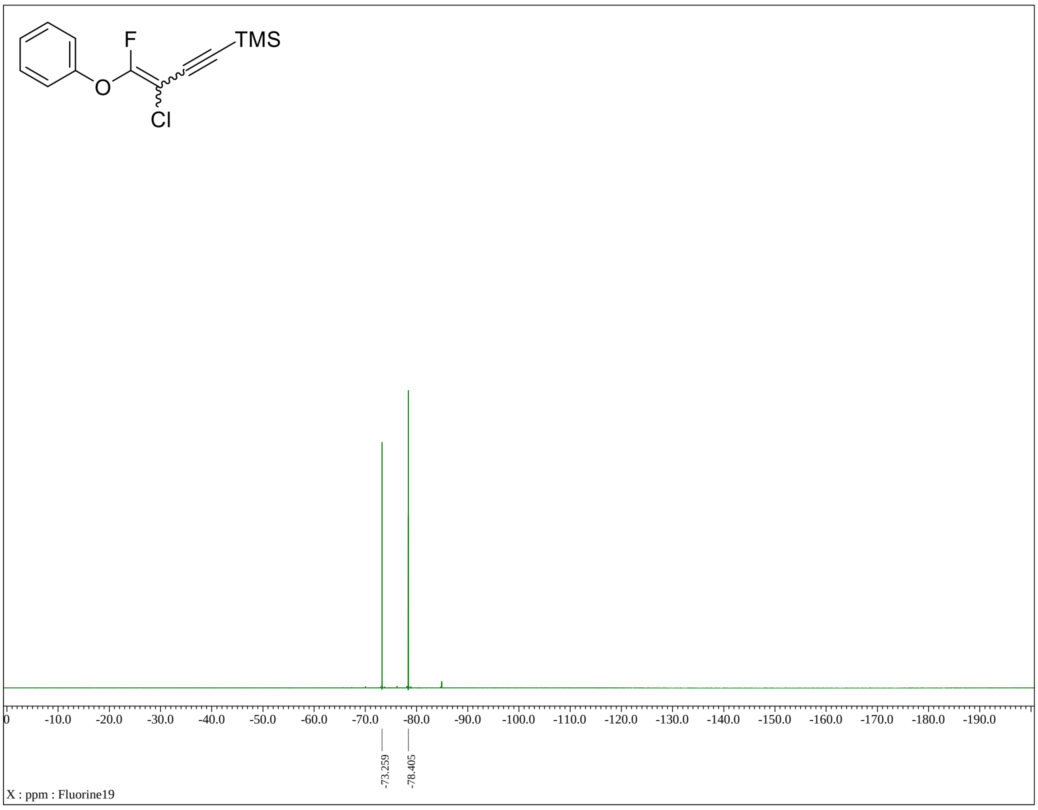

2-Chloro-1-fluoro-1-phenoxy-4-phenylbut-1-en-3-yne (3b)

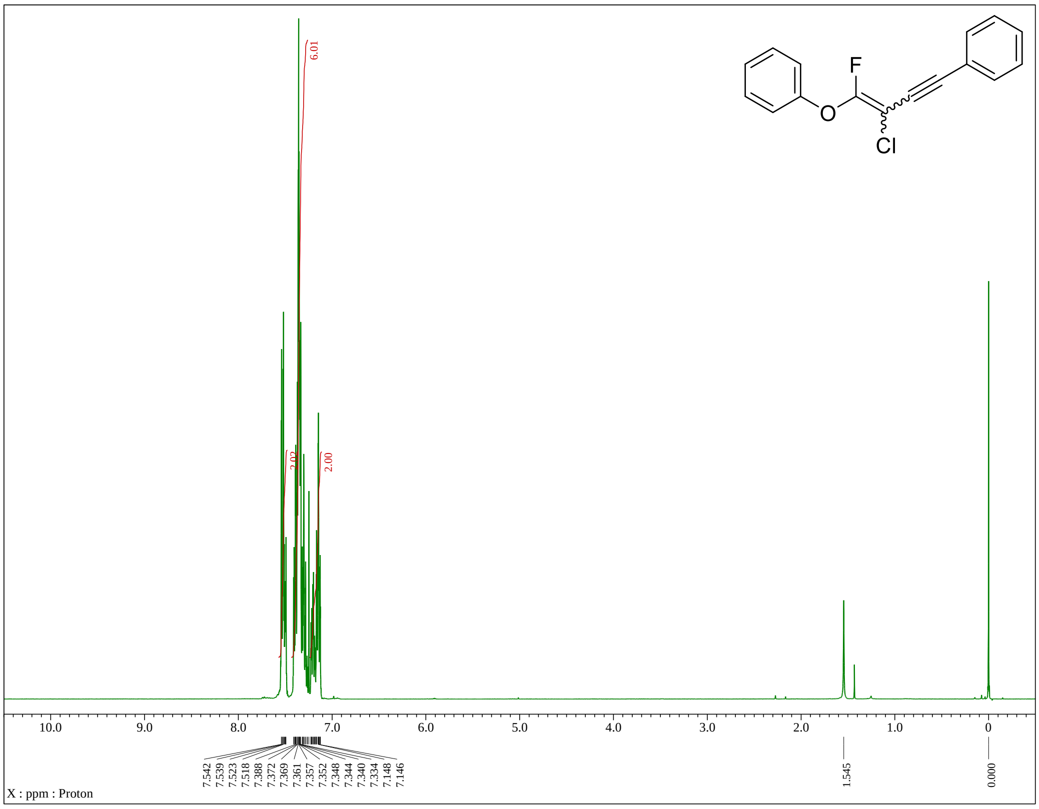

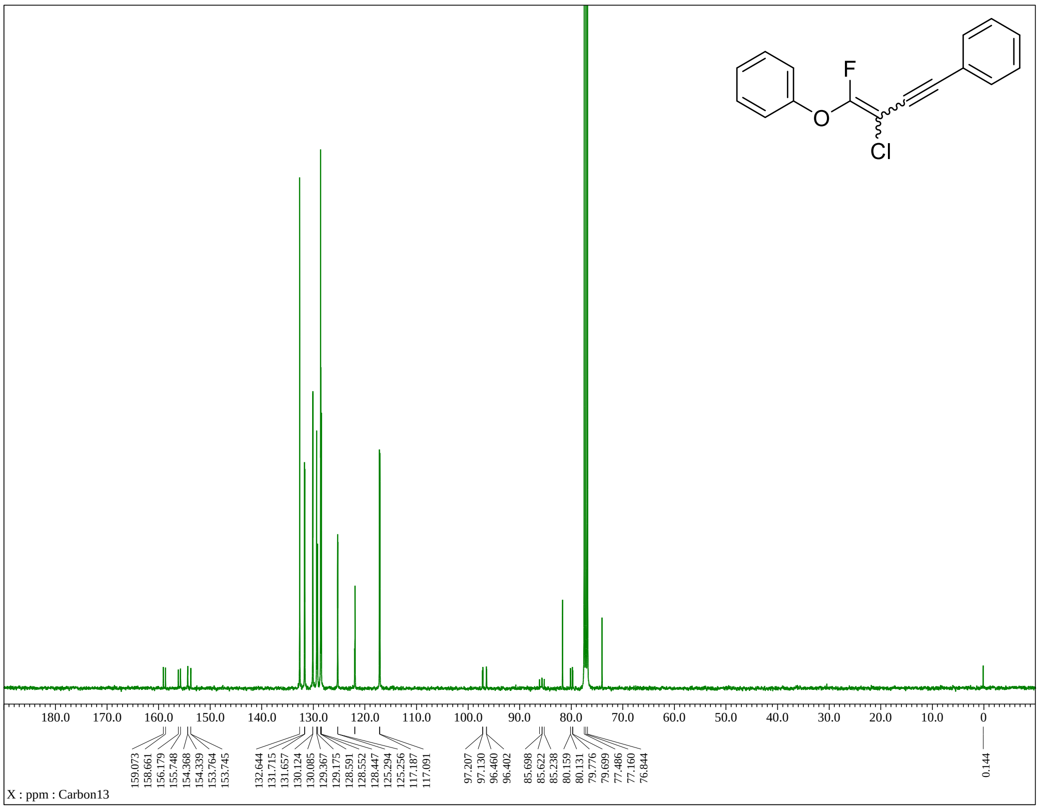
F
O
Cl
180.0 170.0 160.0 150.0 140.0 130.0 120.0 110.0 100.0 90.0 80.0 70.0 60.0 50.0 40.0 30.0 20.0 10.0 0
159.073 158.661 156.179 155.748 154.368 154.339 153.764 153.745
132.644 131.715 131.657 130.124 130.085 129.367 129.175 128.591 128.552 128.447 125.294 125.256 117.187 117.091
97.207 97.130 96.460 96.402
85.698 85.622 85.238 80.159 80.131 79.776 79.699 77.486 77.160 76.844
0.144
X : ppm : Carbon13

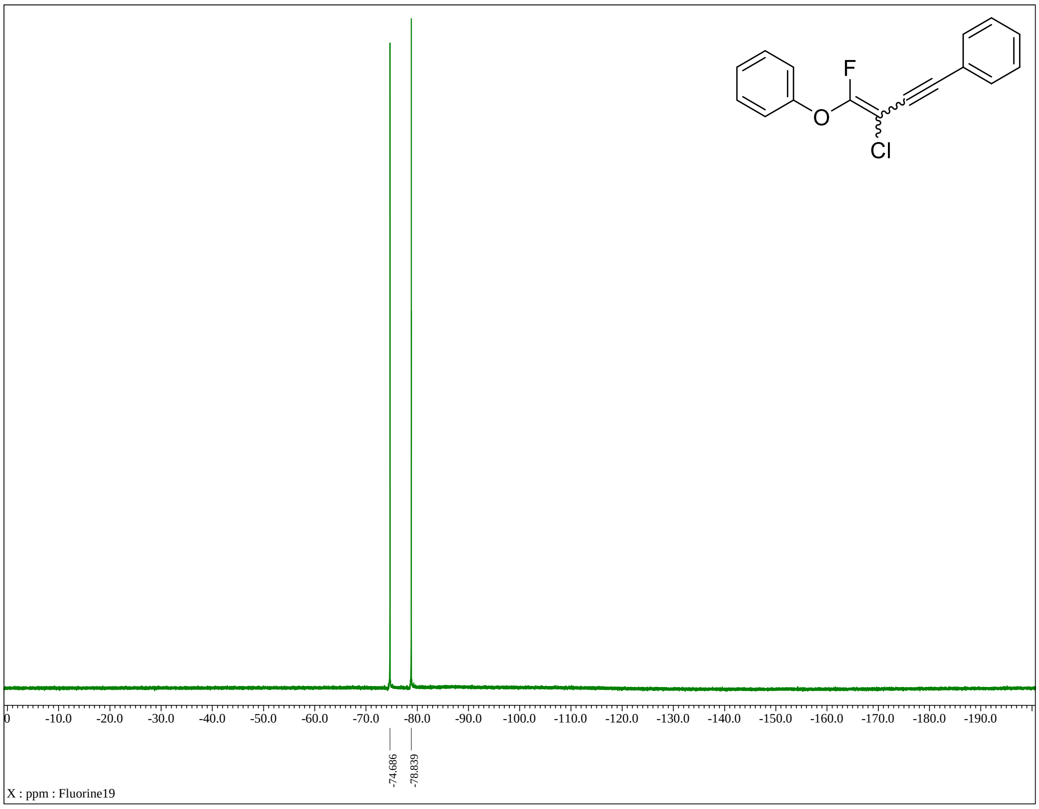
F
O
Cl
0 -10.0 -20.0 -30.0 -40.0 -50.0 -60.0 -70.0 -80.0 -90.0 -100.0 -110.0 -120.0 -130.0 -140.0 -150.0 -160.0 -170.0 -180.0 -190.0
-74.686
-78.839
X : ppm : Fluorine19

2-Chloro-1-fluoro-4-(4-methylphenyl)-1-phenoxybut-1-en-3-yne (3c)

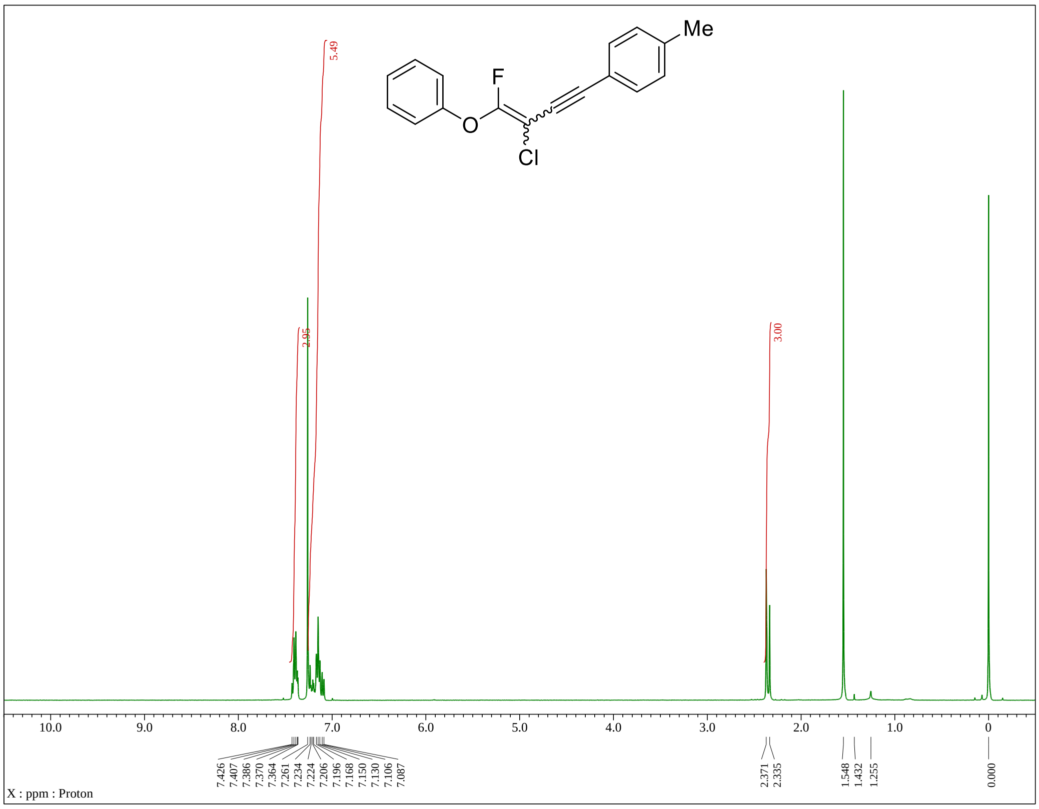

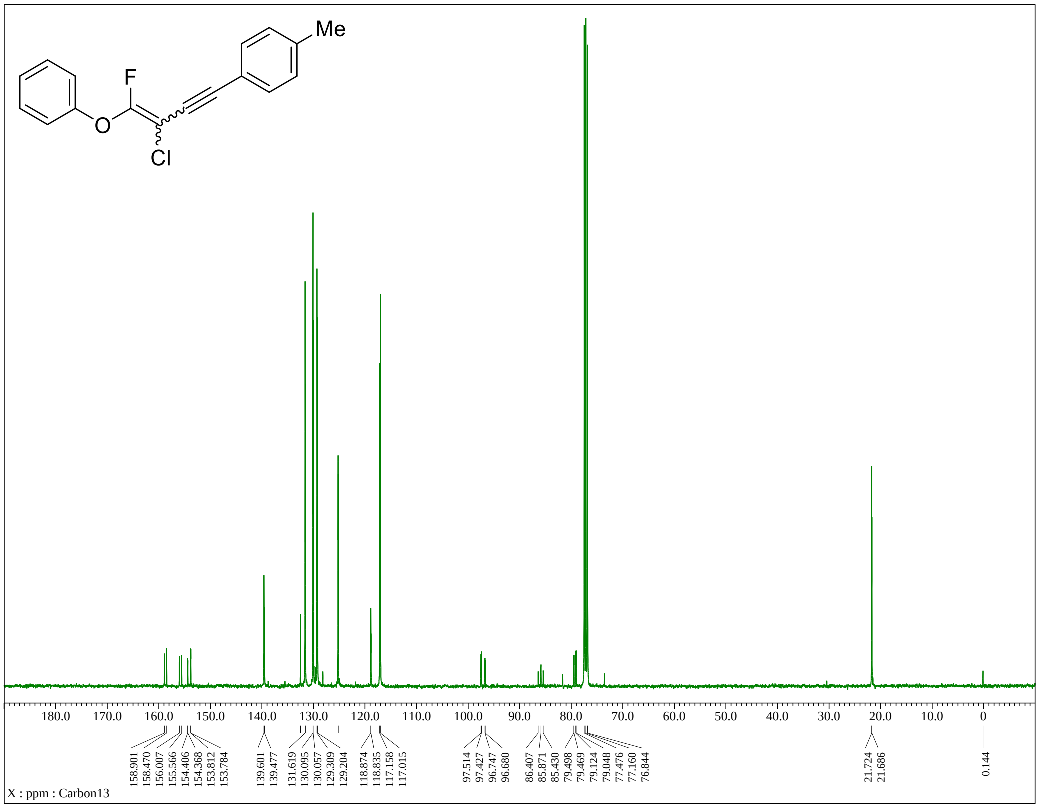

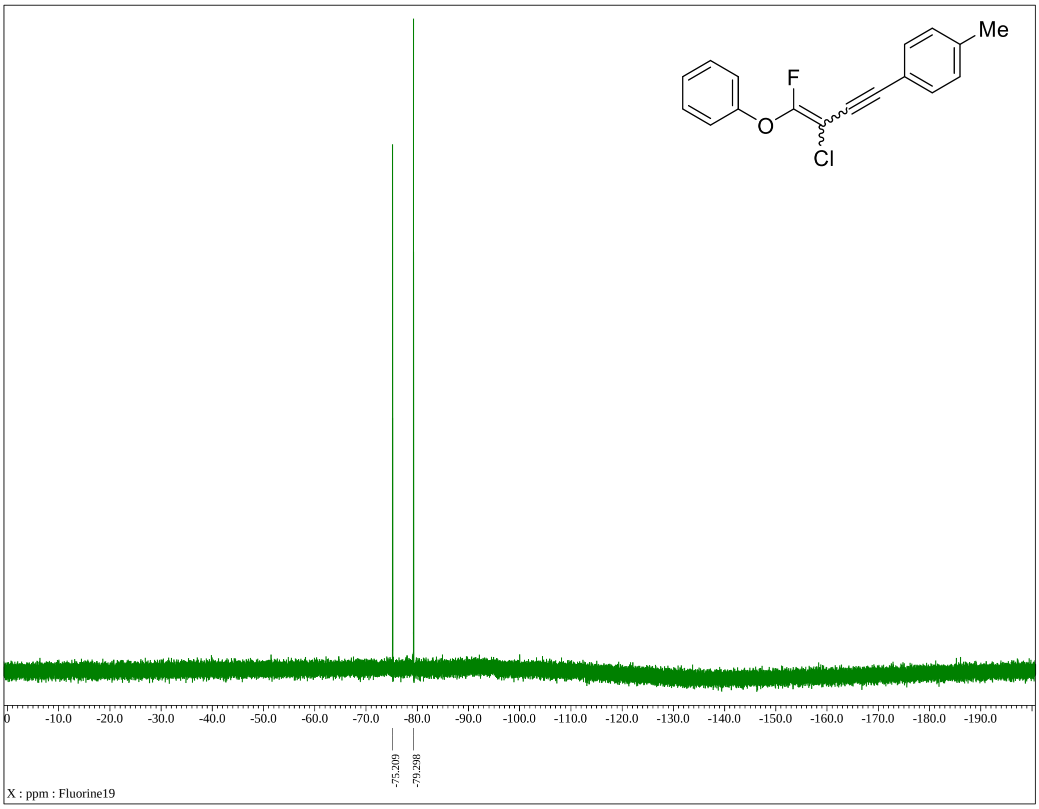

2-Chloro-1-fluoro-4-(4-methoxyphenyl)-1-phenoxybut-1-en-3-yne (3d)

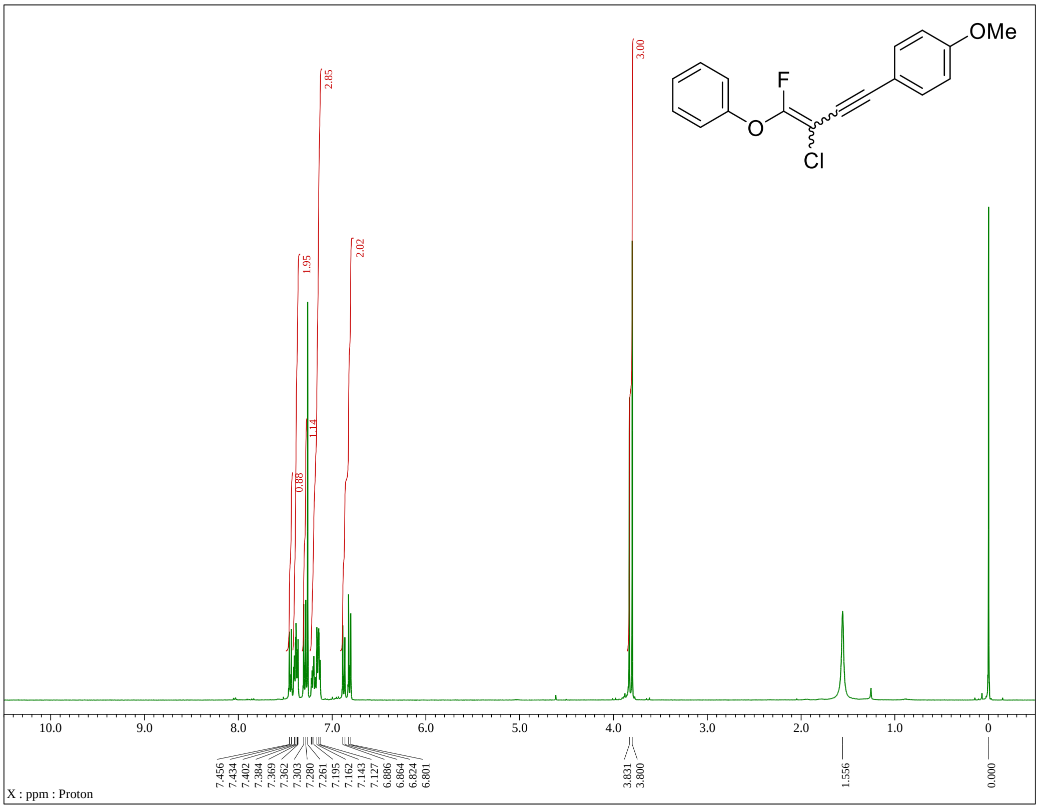

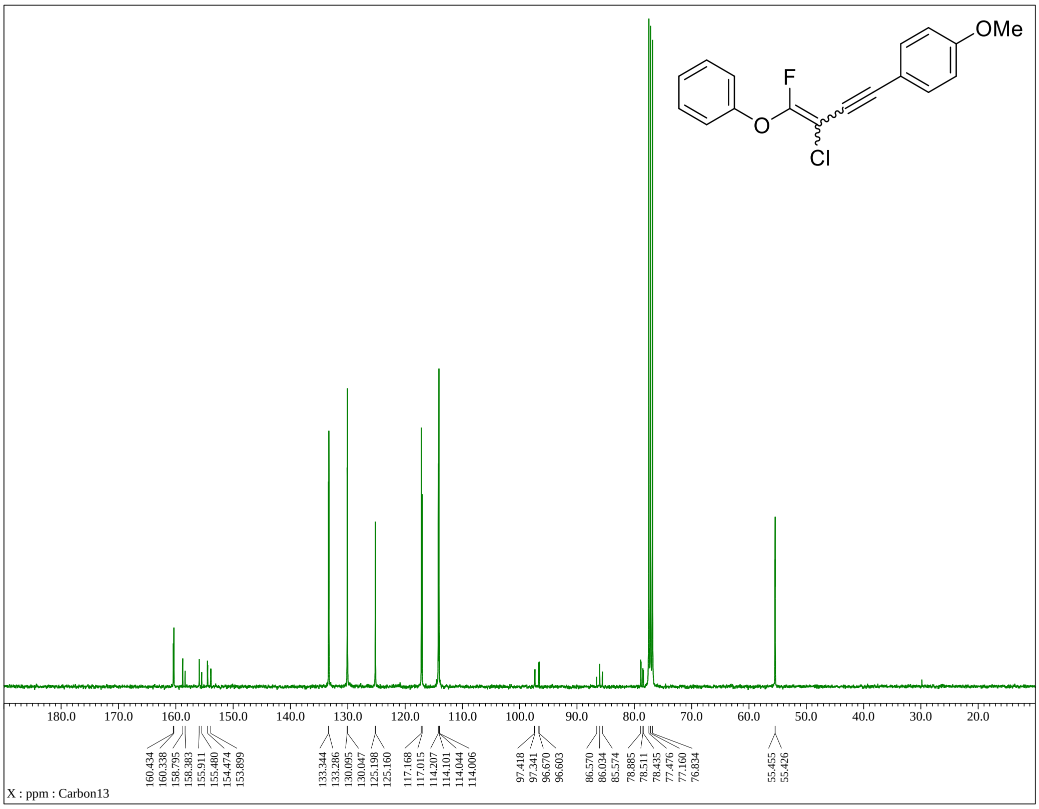
OMe
F
O
Cl
180.0
170.0
160.0
150.0
140.0
130.0
120.0
110.0
100.0
90.0
80.0
70.0
60.0
50.0
40.0
30.0
20.0
160.434
160.338
158.795
158.383
155.911
155.480
154.474
153.899
133.344
133.286
130.095
130.047
125.198
125.160
117.168
117.015
114.207
114.101
114.044
114.006
97.418
97.341
96.670
96.603
86.570
86.034
85.574
78.885
78.511
78.435
77.476
77.160
76.834
55.455
55.426
X : ppm : Carbon13

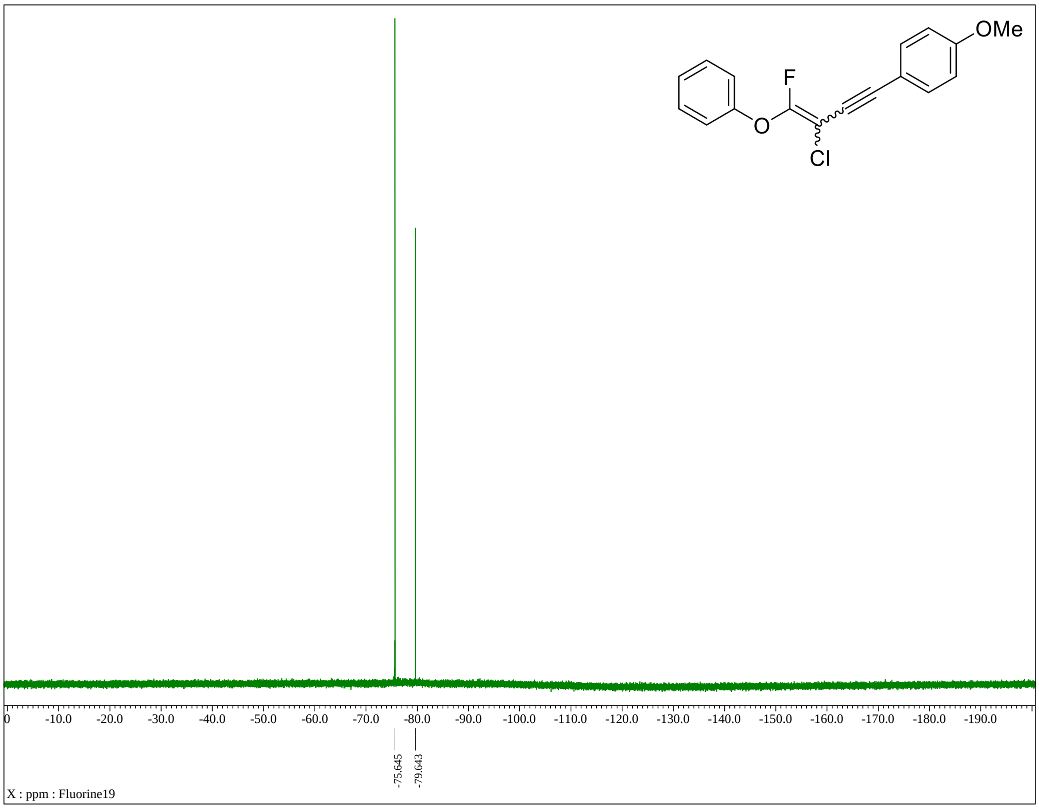
OMe
F
O
Cl
-10.0
-20.0
-30.0
-40.0
-50.0
-60.0
-70.0
-80.0
-90.0
-100.0
-110.0
-120.0
-130.0
-140.0
-150.0
-160.0
-170.0
-180.0
-190.0
-75.645
-79.643
X : ppm : Fluorine19

4-(4-*tert*-Butylphenyl)-2-chloro-1-fluoro-1-phenoxybut-1-en-3-yne (3e)

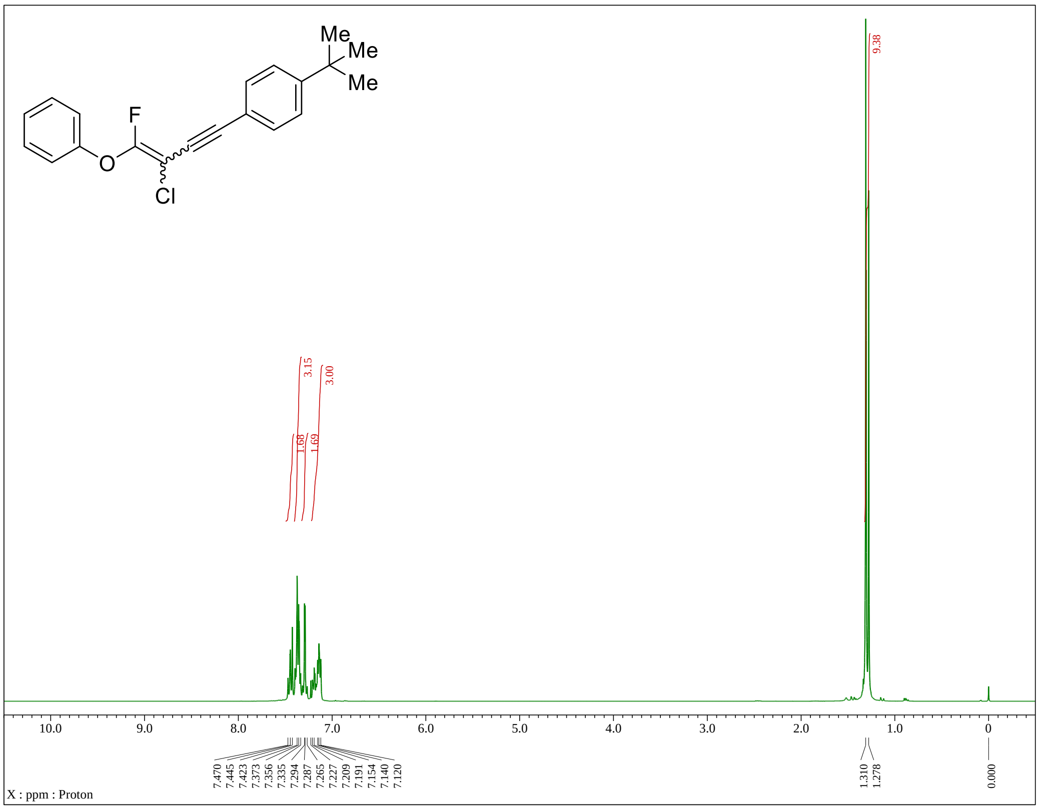

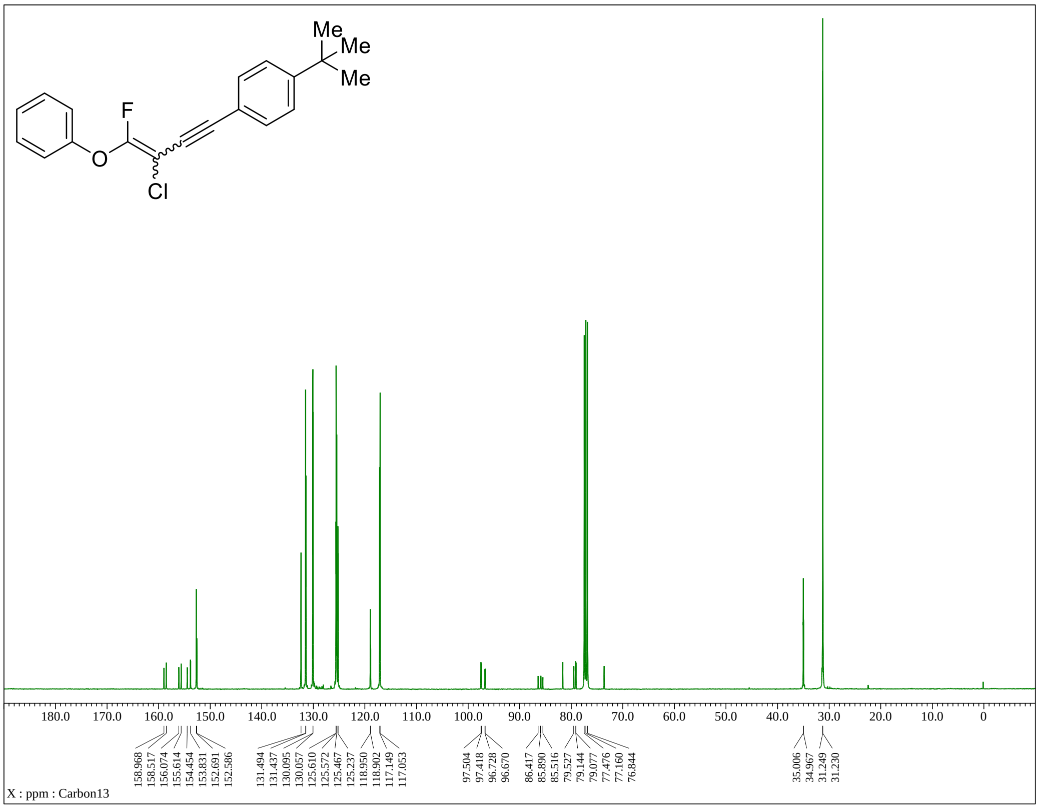

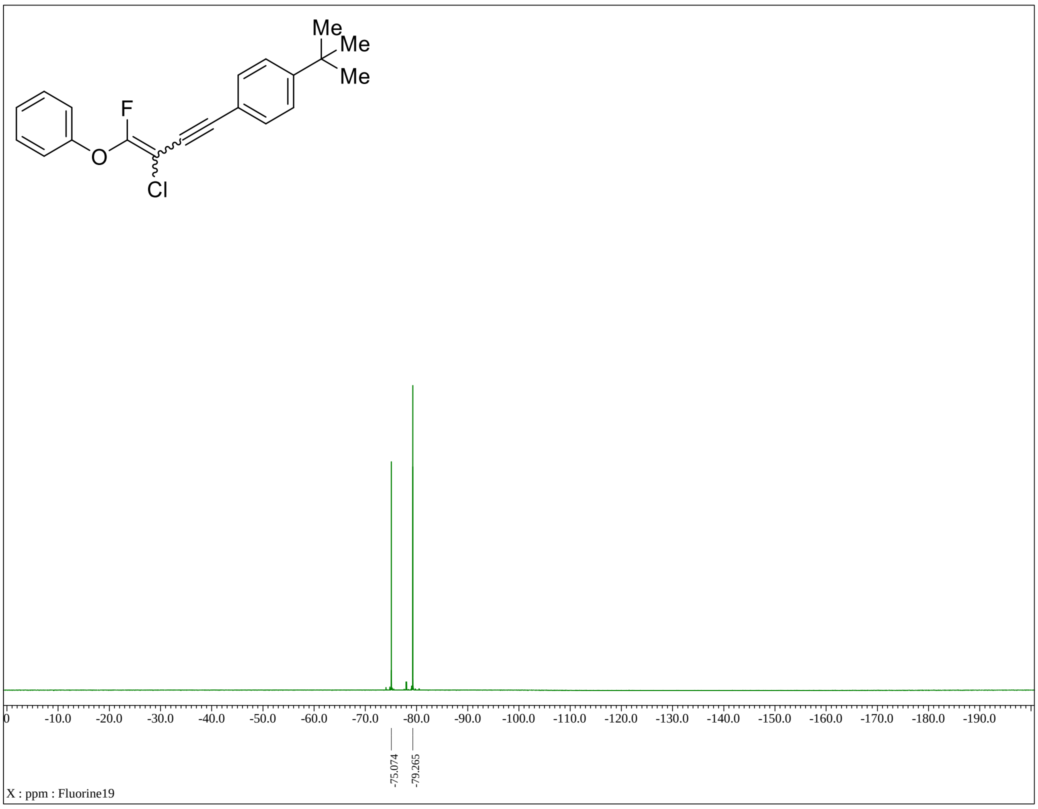

2-Chloro-1-fluoro-1-phenoxy-4-(4-phenylphenyl)but-1-en-3-yne (3f)

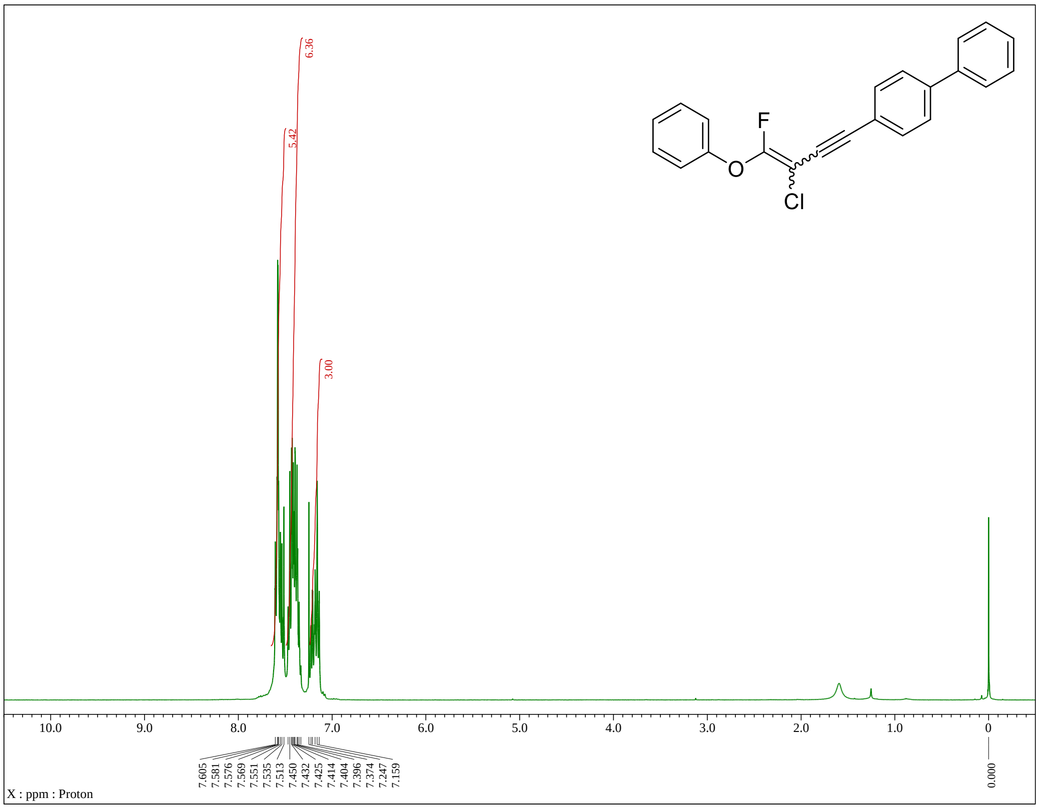

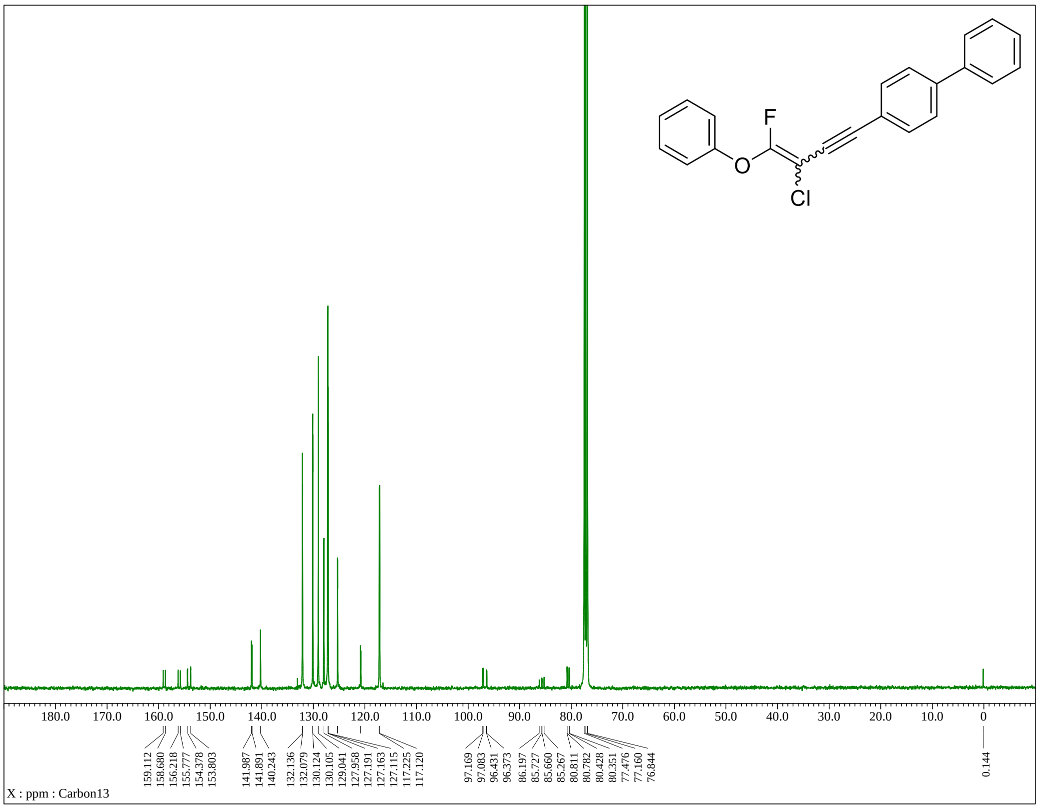
F
O
Cl
180.0 170.0 160.0 150.0 140.0 130.0 120.0 110.0 100.0 90.0 80.0 70.0 60.0 50.0 40.0 30.0 20.0 10.0 0
159.112
158.680
156.218
155.777
154.378
153.803
141.987
141.891
140.243
132.136
132.079
130.124
130.105
129.041
127.958
127.191
127.163
127.115
117.225
117.120
97.169
97.083
96.431
96.373
86.197
85.727
85.660
85.267
80.811
80.782
80.428
80.351
77.476
77.160
76.844
0.144
X : ppm : Carbon13

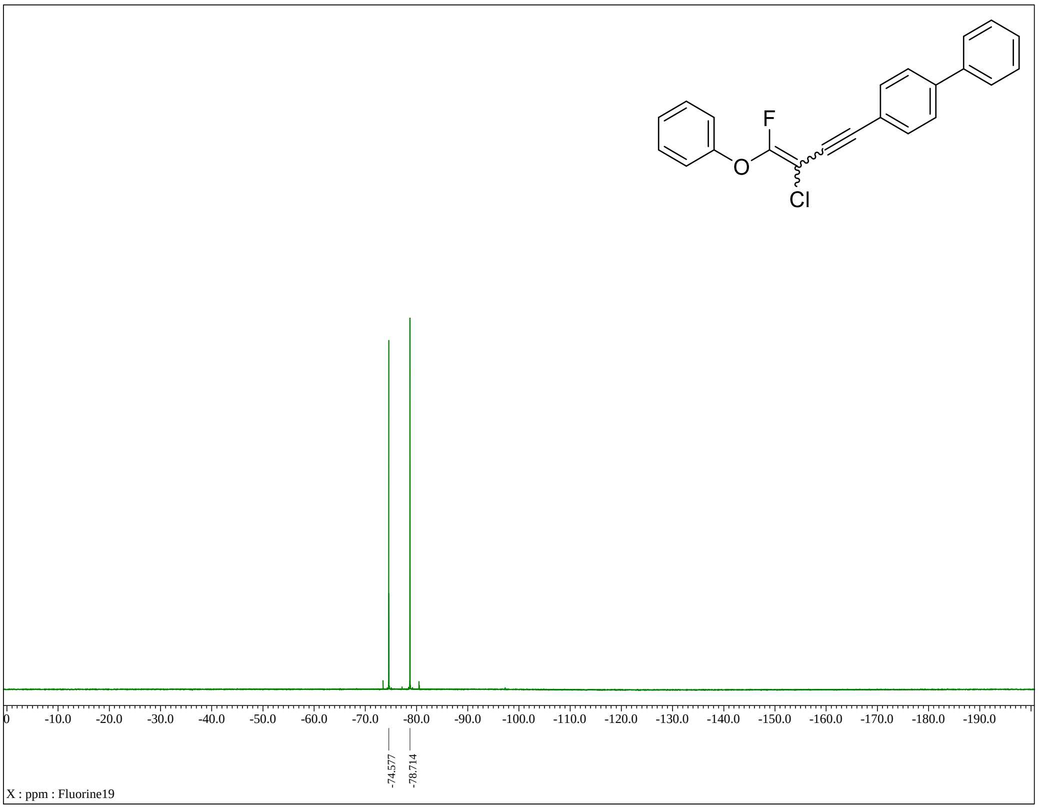
F
O
Cl
0 -10.0 -20.0 -30.0 -40.0 -50.0 -60.0 -70.0 -80.0 -90.0 -100.0 -110.0 -120.0 -130.0 -140.0 -150.0 -160.0 -170.0 -180.0 -190.0
-74.577
-78.714
X : ppm : Fluorine19

2-Chloro-1-fluoro-4-(2-naphthyl)-1-phenoxybut-1-en-3-yne (3g)

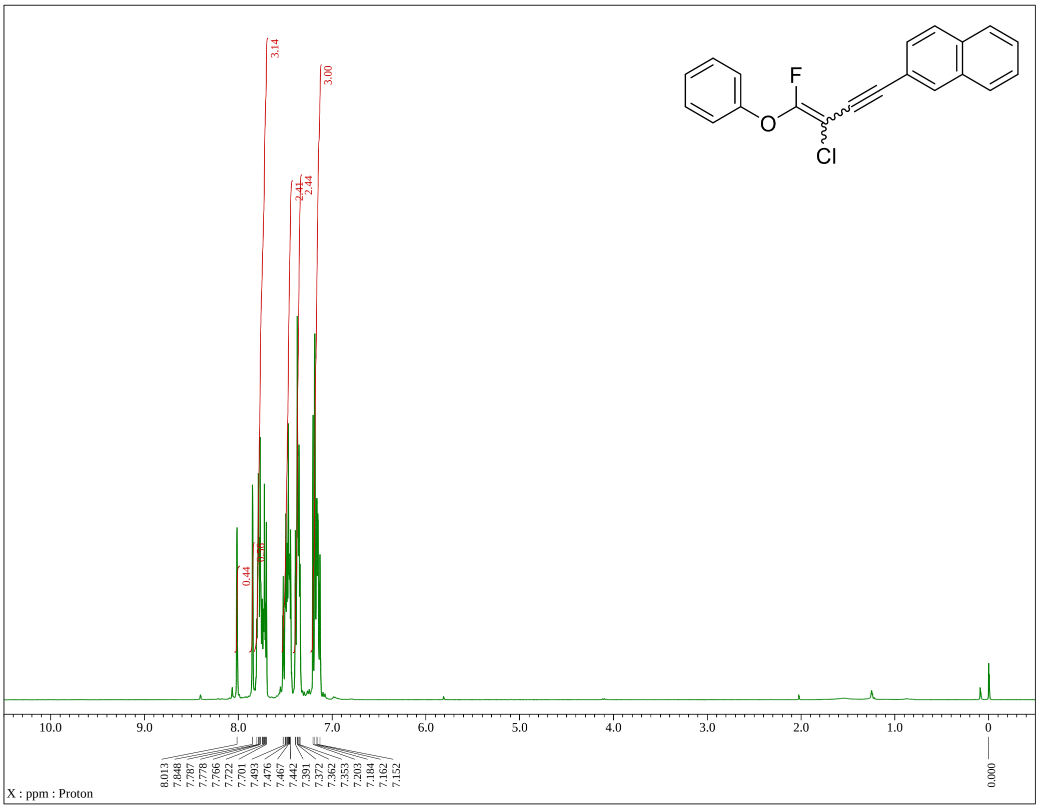

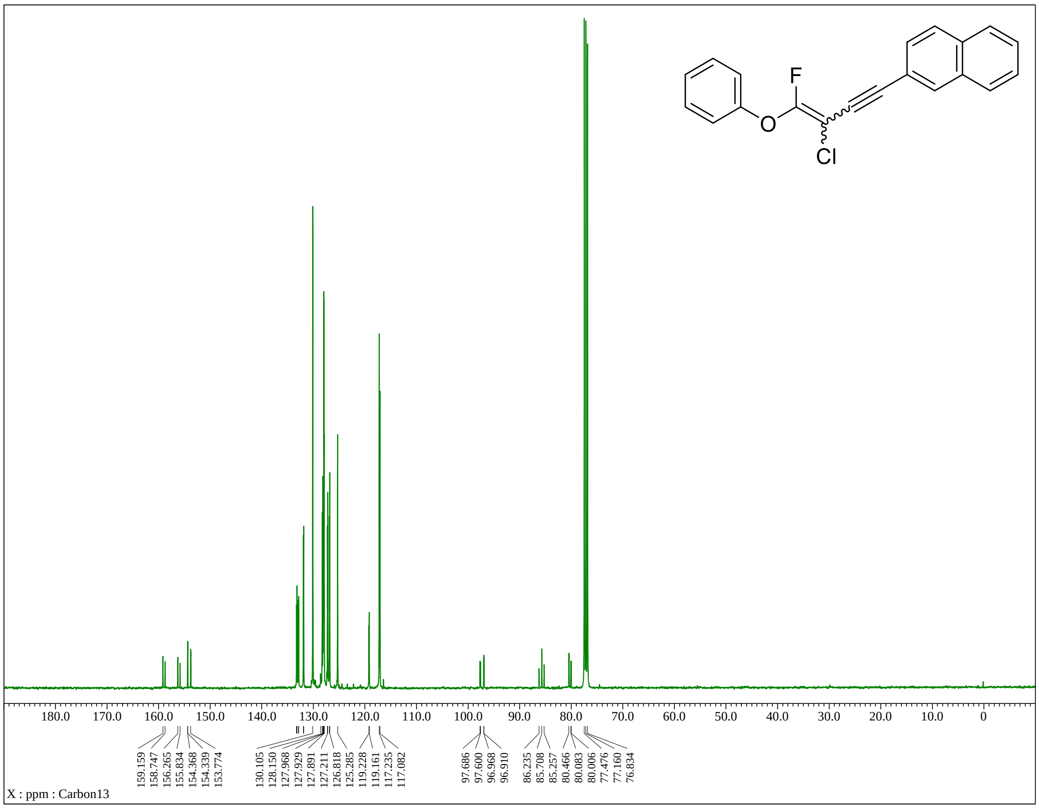

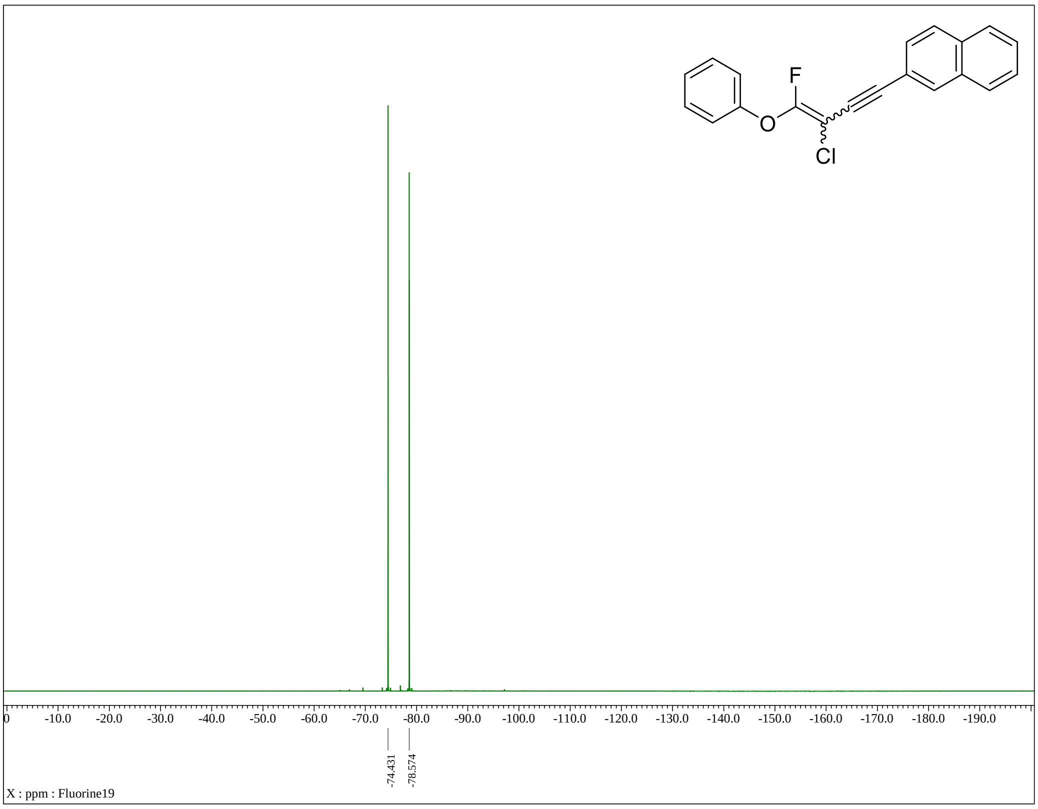

2-Chloro-4-(4-chlorophenyl)-1-fluoro-1-phenoxybut-1-en-3-yne (3h)

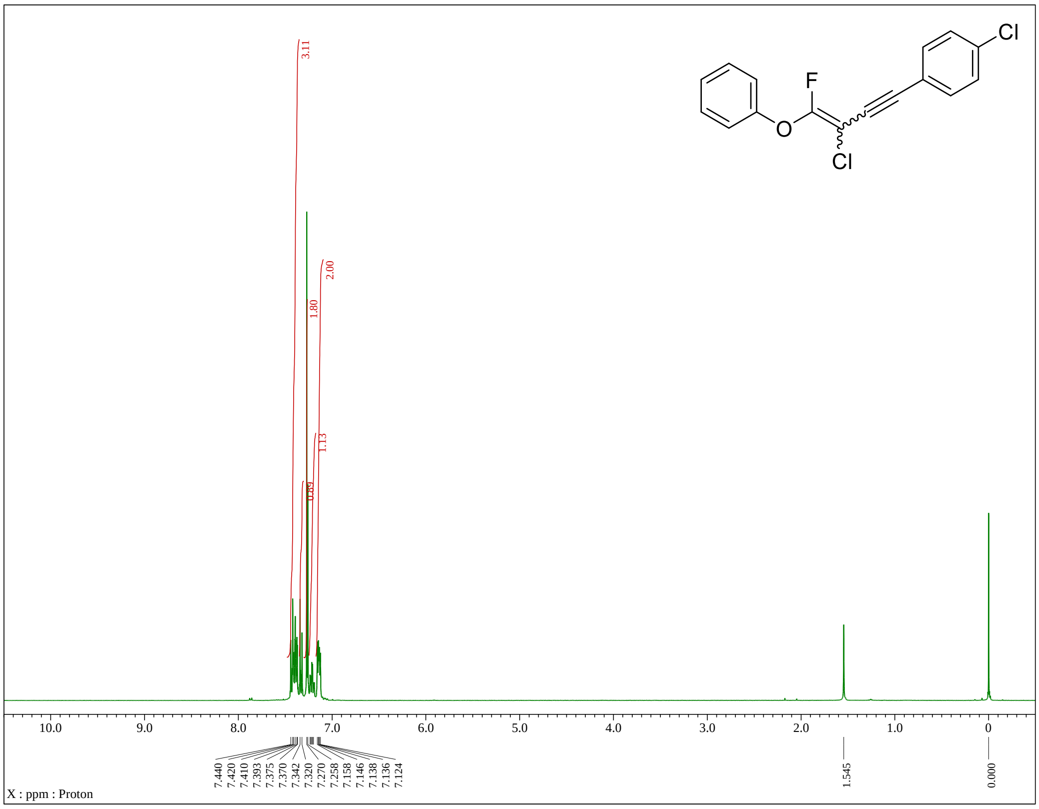

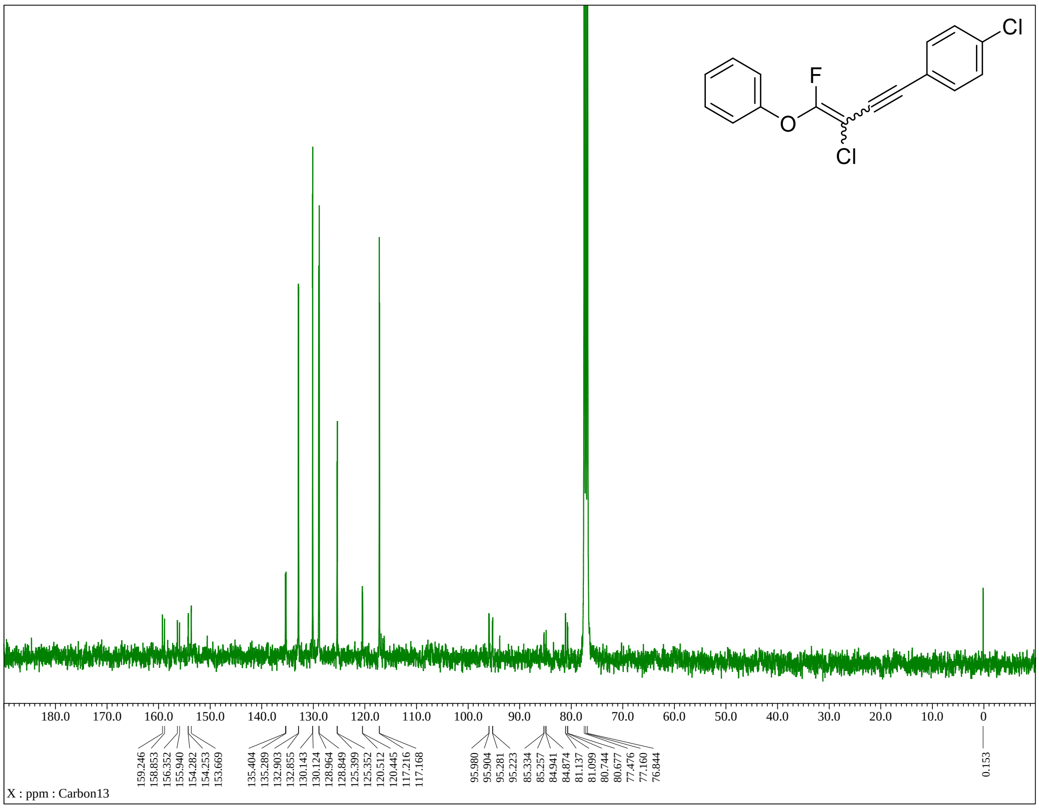
F
O
Cl
Cl
180.0 170.0 160.0 150.0 140.0 130.0 120.0 110.0 100.0 90.0 80.0 70.0 60.0 50.0 40.0 30.0 20.0 10.0 0
159.246 158.853 156.352 155.940 154.282 154.253 153.669
135.404 135.289 132.903 132.855 130.143 130.124 128.964 128.849 125.399 125.352 120.512 120.445 117.216 117.168
95.980 95.904 95.281 95.223 85.334 85.257 84.941 84.874 81.137 81.099 80.744 80.677 77.476 77.160 76.844
0.153
X : ppm : Carbon13

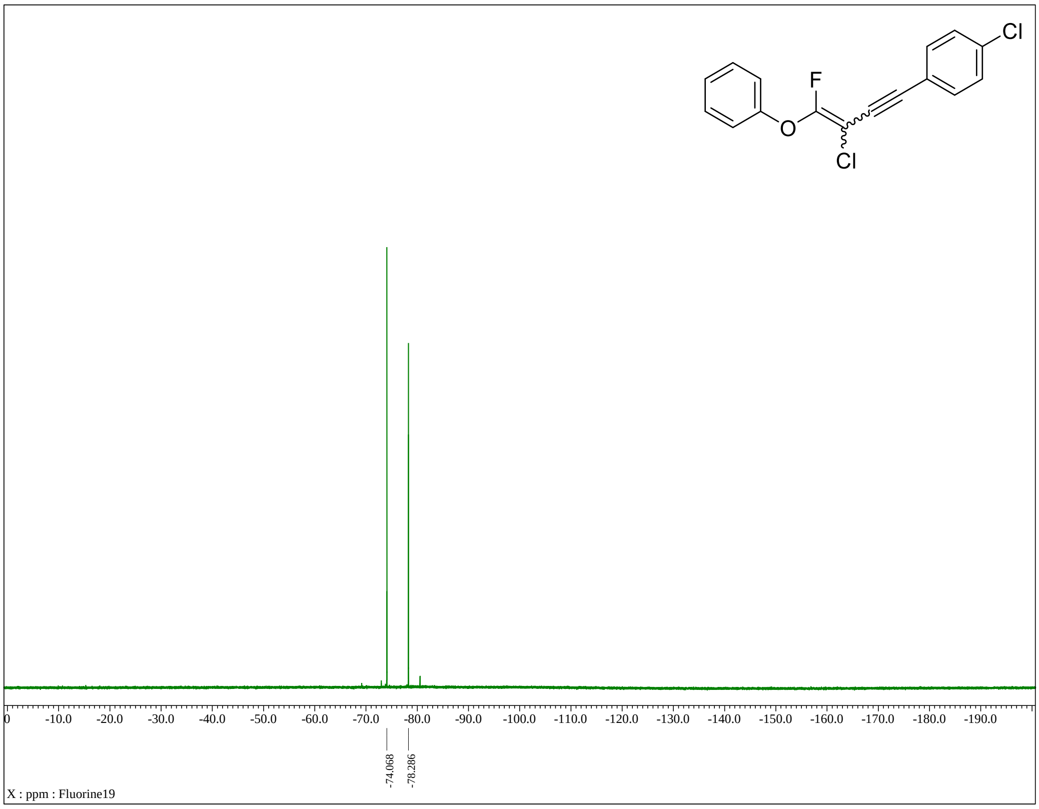
F
O
Cl
Cl
0 -10.0 -20.0 -30.0 -40.0 -50.0 -60.0 -70.0 -80.0 -90.0 -100.0 -110.0 -120.0 -130.0 -140.0 -150.0 -160.0 -170.0 -180.0 -190.0
-74.068
-78.286
X : ppm : Fluorine19

2-Chloro-1-fluoro-1-phenoxy-4-(4-trifluoromethylphenyl)but-1-en-3-yne (3i)

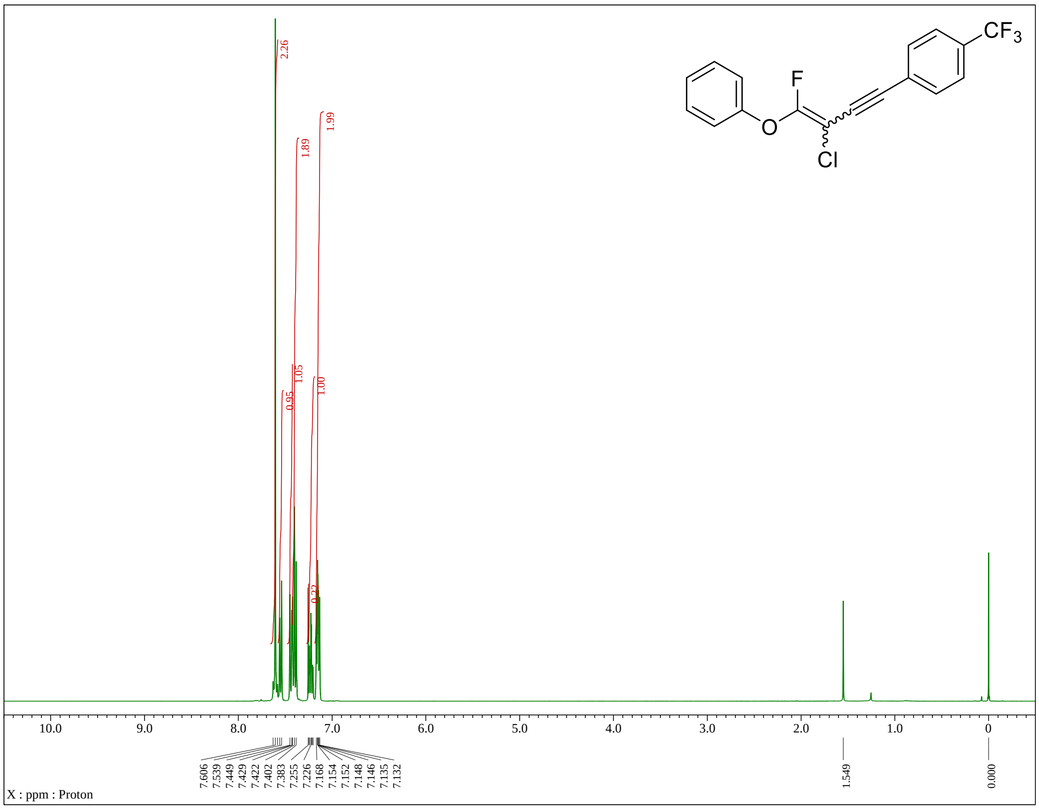

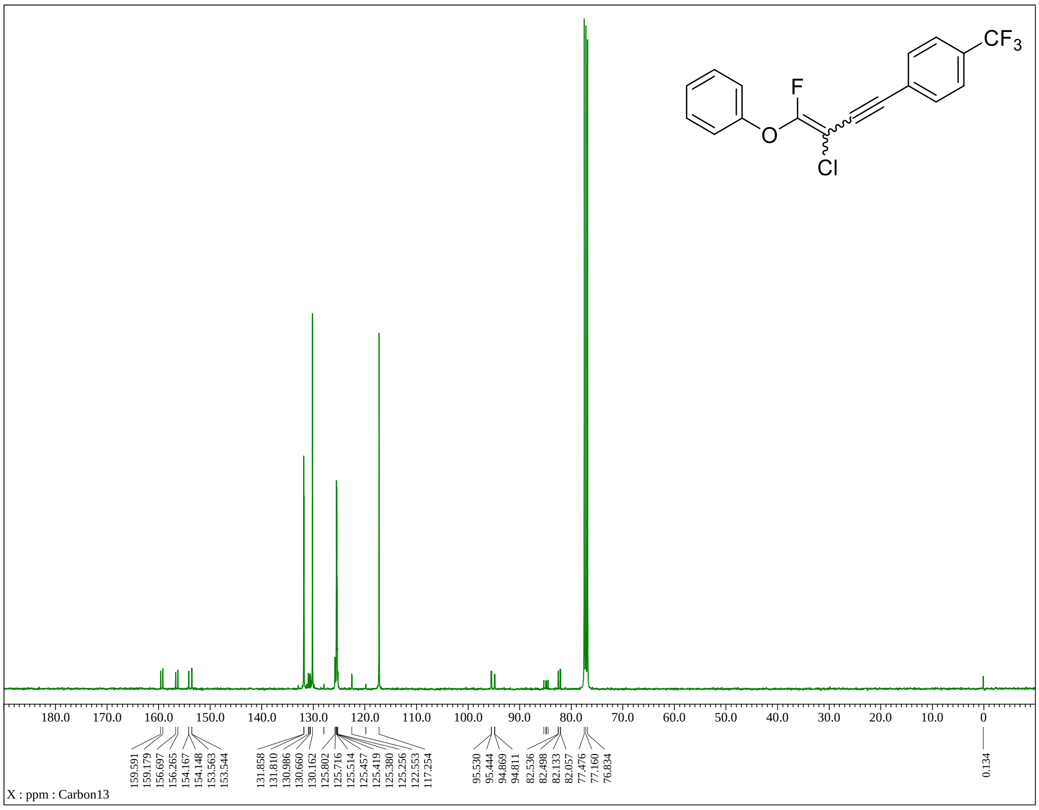

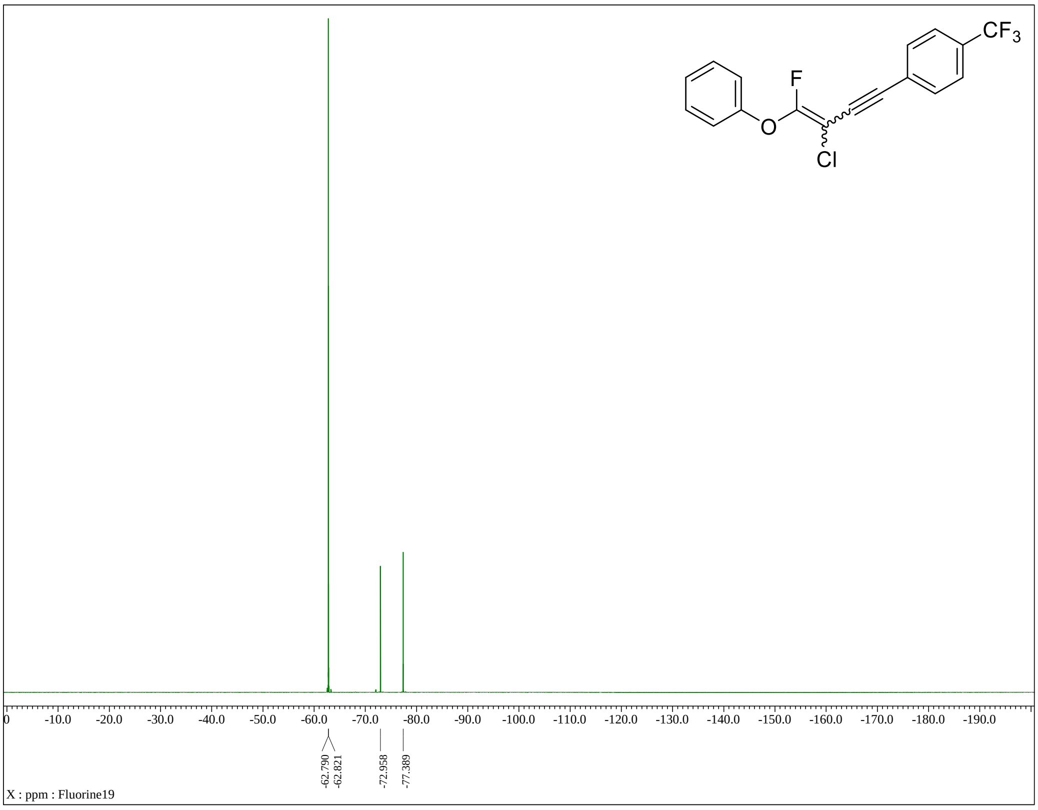

2-Chloro-1-fluoro-4-(4-nitrophenyl)-1-phenoxybut-1-en-3-yne (3j)

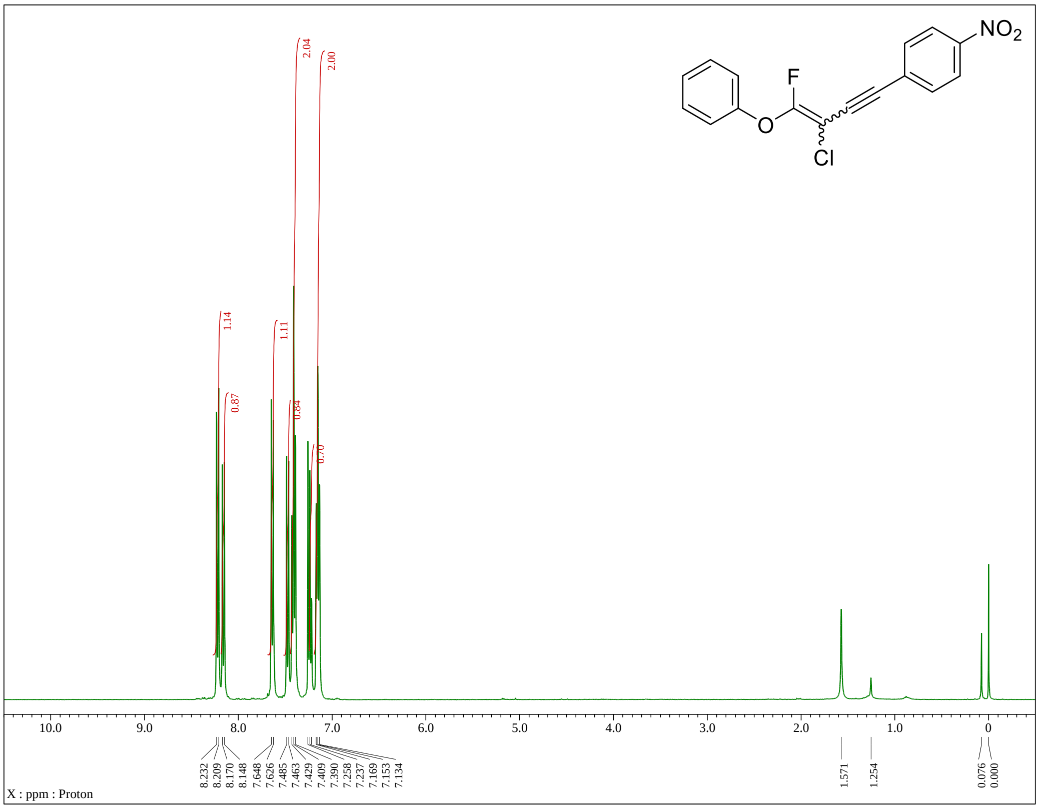

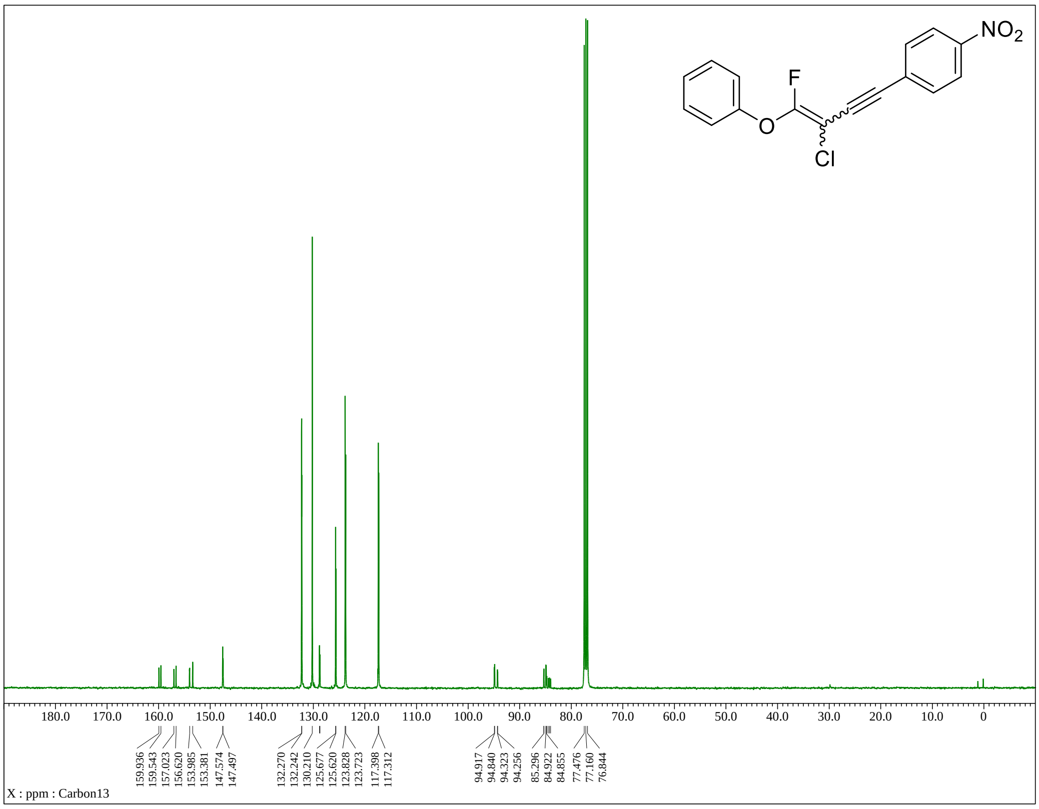
NO2
F
O
Cl
180.0
170.0
160.0
150.0
140.0
130.0
120.0
110.0
100.0
90.0
80.0
70.0
60.0
50.0
40.0
30.0
20.0
10.0
0
159.936
159.543
157.023
156.620
153.985
153.381
147.574
147.497
132.270
132.242
130.210
125.677
125.620
123.828
123.723
117.398
117.312
94.917
94.840
94.323
94.256
85.296
84.922
84.855
77.476
77.160
76.844
X : ppm : Carbon13

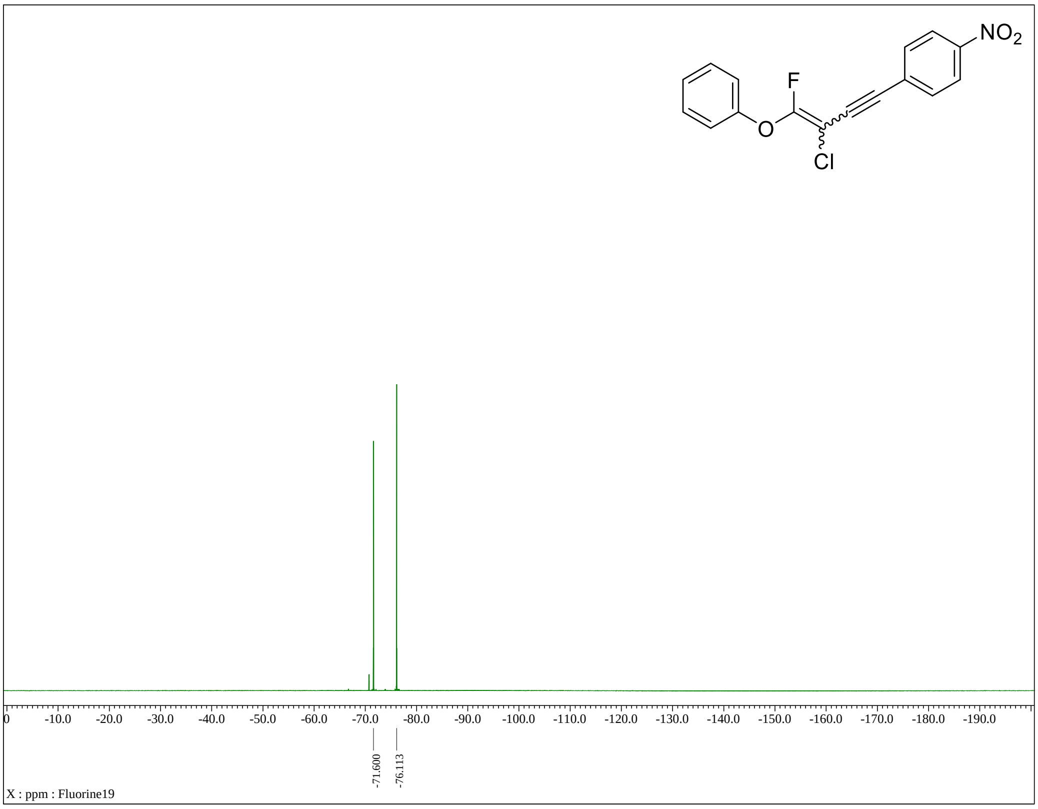
NO2
F
O
Cl
0
-10.0
-20.0
-30.0
-40.0
-50.0
-60.0
-70.0
-80.0
-90.0
-100.0
-110.0
-120.0
-130.0
-140.0
-150.0
-160.0
-170.0
-180.0
-190.0
-71.600
-76.113
X : ppm : Fluorine19

4-(4-Acetylphenyl)-2-chloro-1-fluoro-1-phenoxybut-1-en-3-yne (3k)

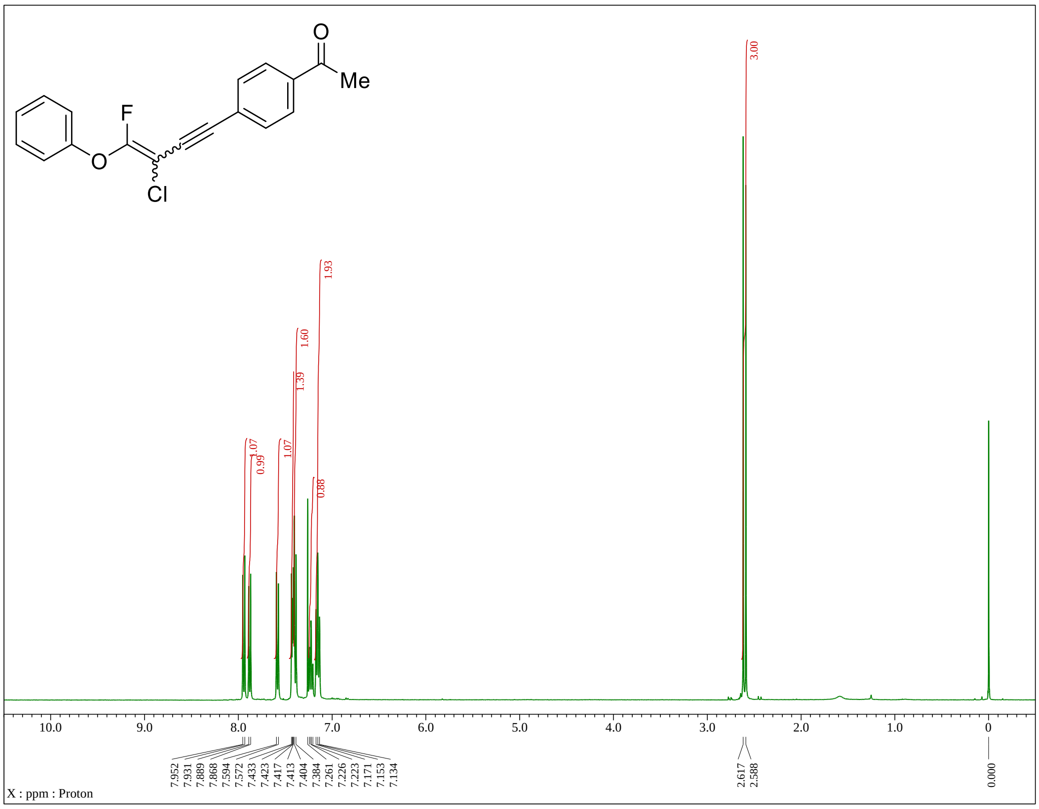

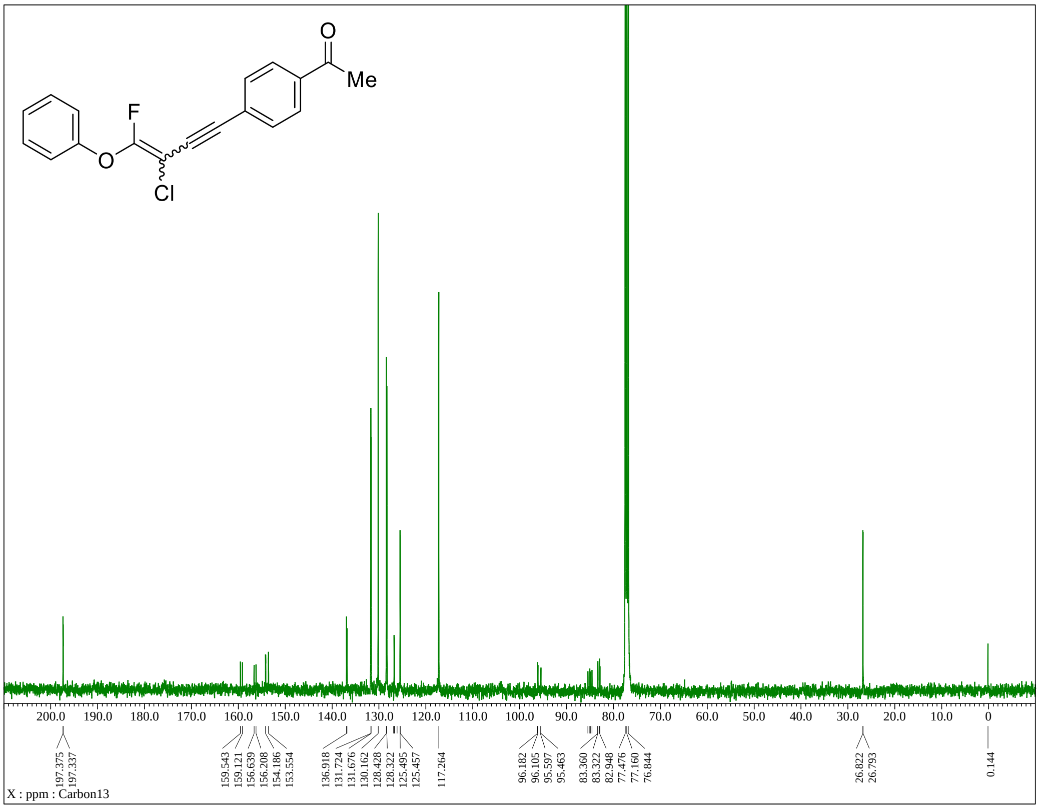

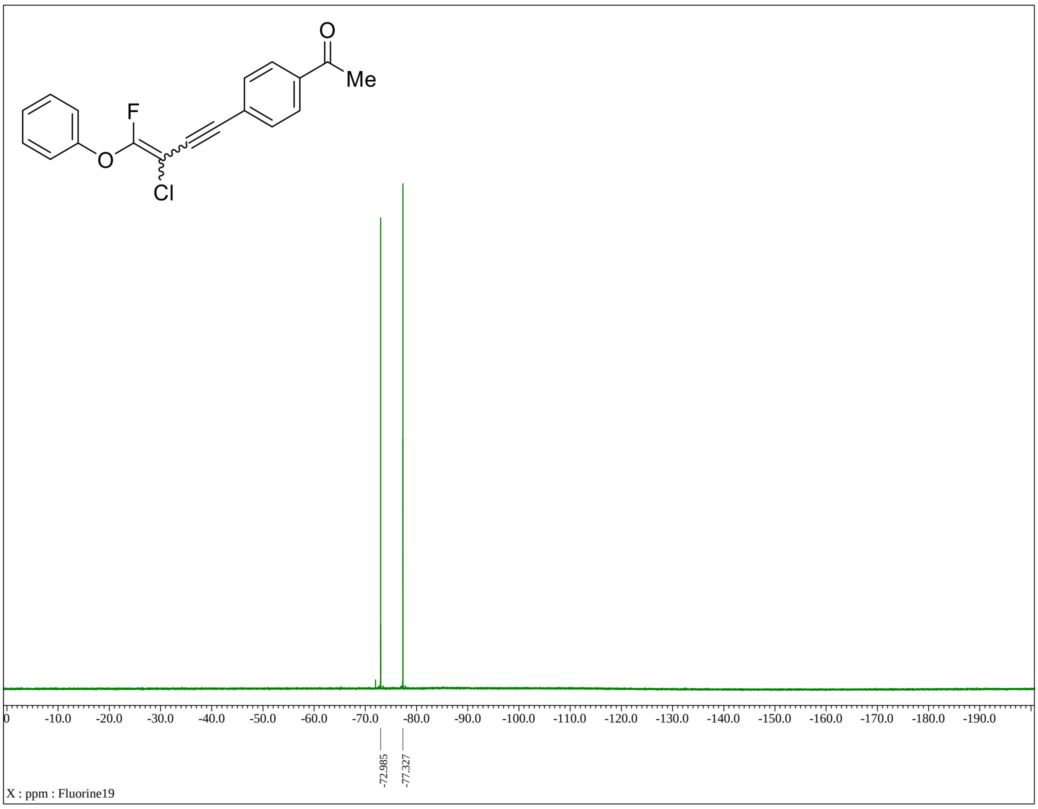

2-Chloro-1-fluoro-4-(4-formylphenyl)-1-phenoxybut-1-en-3-yne (3l)

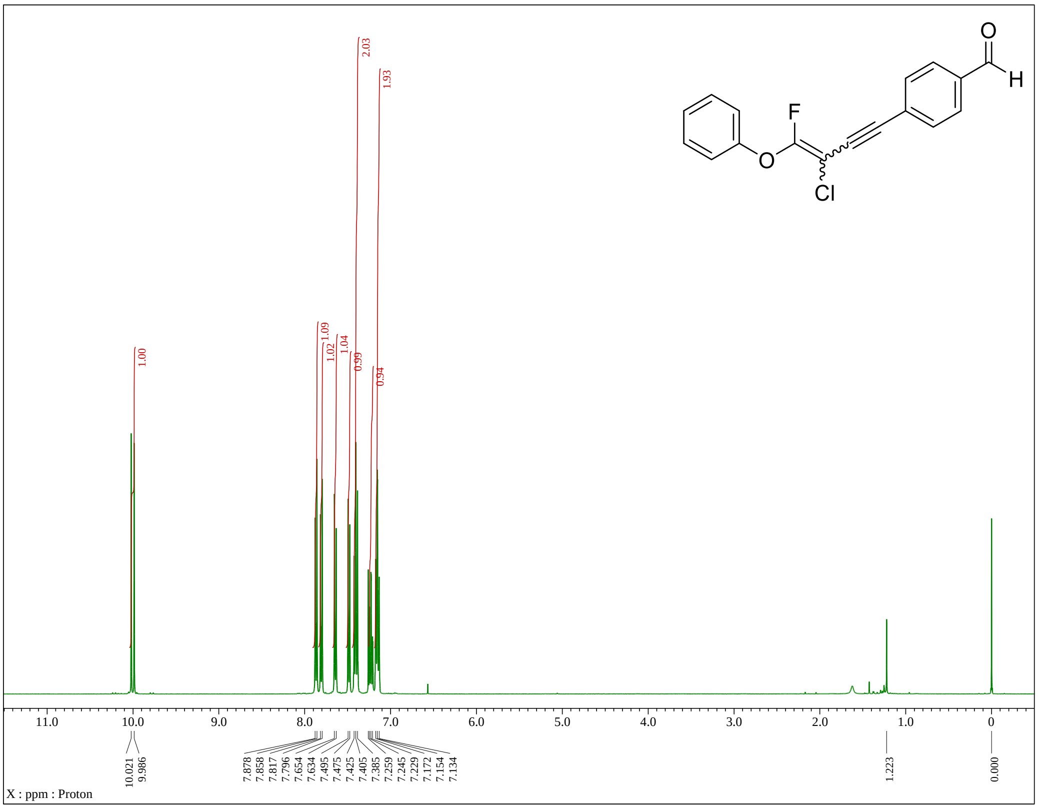

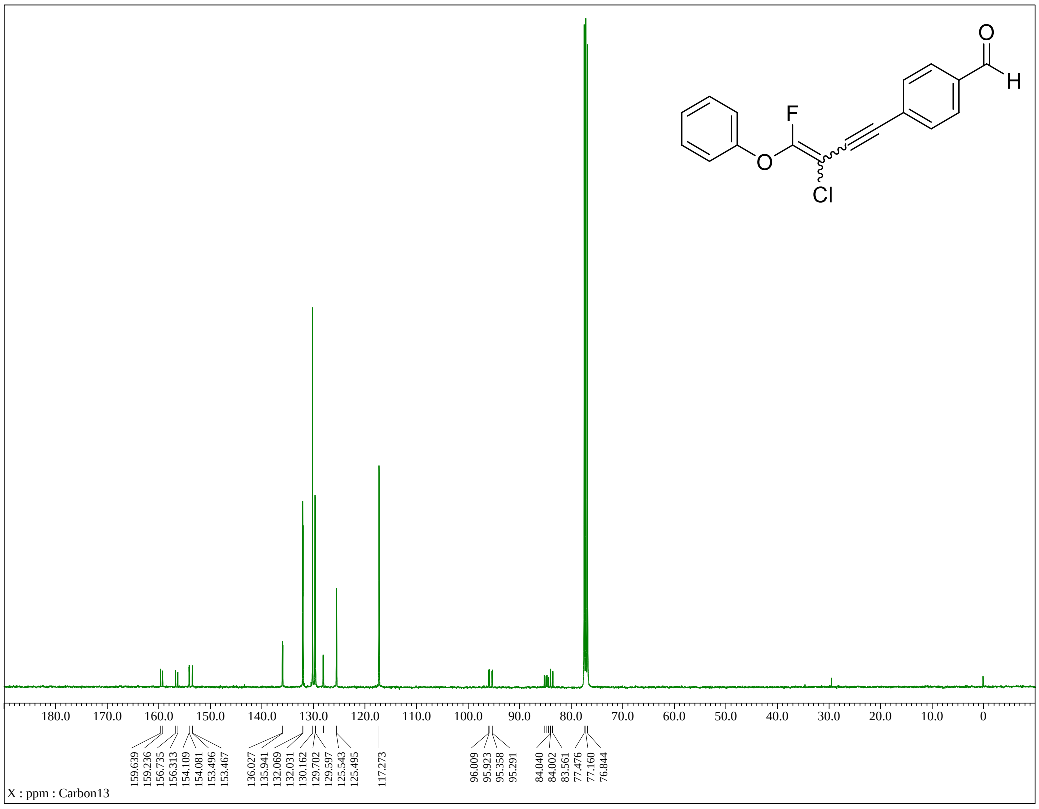
O
H
F
O
Cl
180.0 170.0 160.0 150.0 140.0 130.0 120.0 110.0 100.0 90.0 80.0 70.0 60.0 50.0 40.0 30.0 20.0 10.0 0
159.639 159.236 156.735 156.313 154.109 154.081 153.496 153.467
136.027 135.941 132.069 132.031 130.162 129.702 129.597 125.543 125.495
117.273
96.009 95.923 95.358 95.291
84.040 84.002 83.561 77.476 77.160 76.844
X : ppm : Carbon13

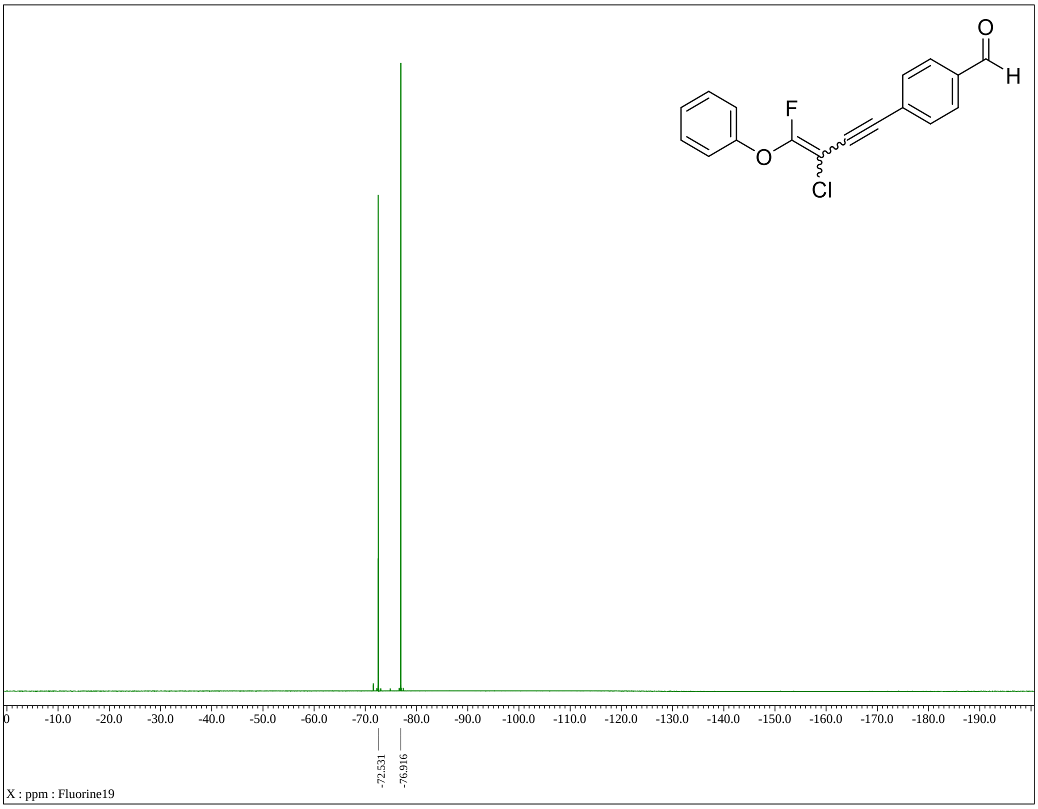
O
H
F
O
Cl
0 -10.0 -20.0 -30.0 -40.0 -50.0 -60.0 -70.0 -80.0 -90.0 -100.0 -110.0 -120.0 -130.0 -140.0 -150.0 -160.0 -170.0 -180.0 -190.0
-72.531
-76.916
X : ppm : Fluorine19

2-Chloro-1-fluoro-4-(3-hydroxyphenyl)-1-phenoxybut-1-en-3-yne (3m)

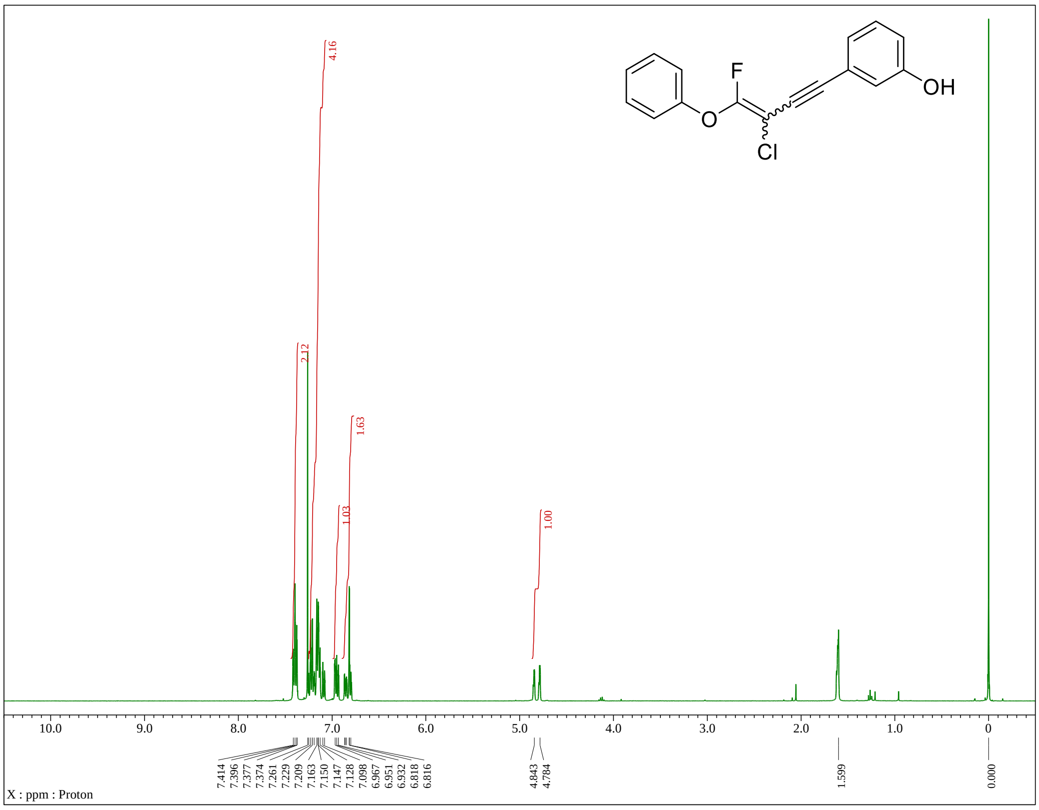

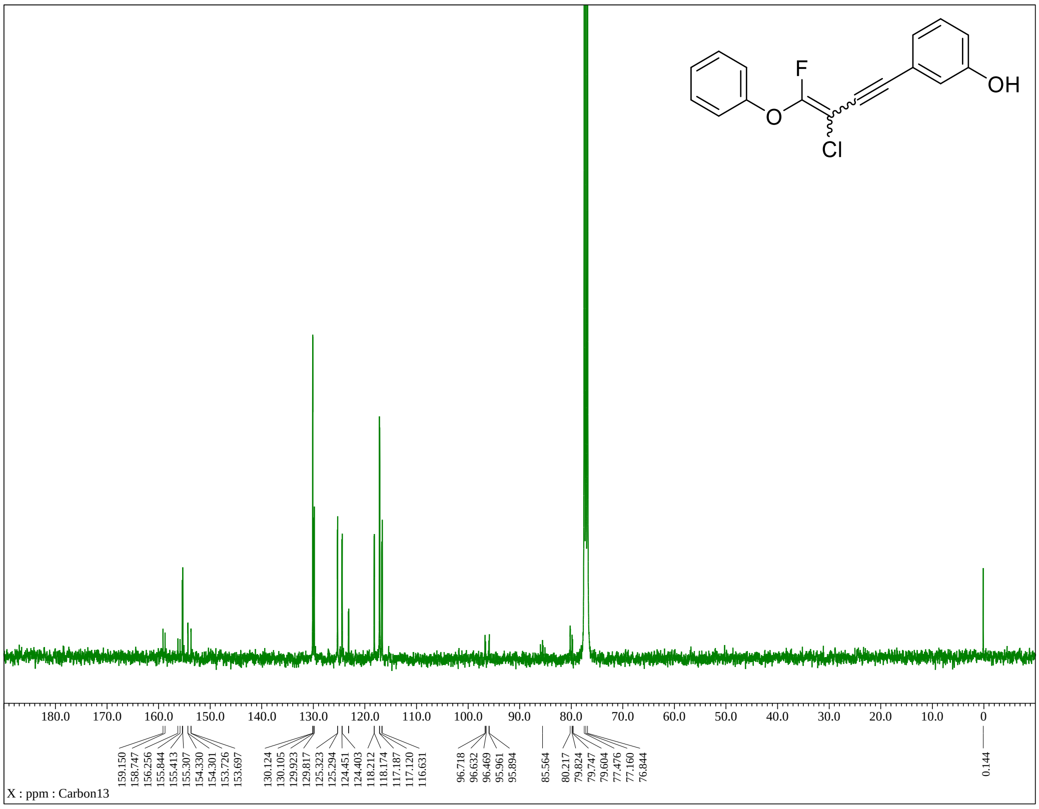

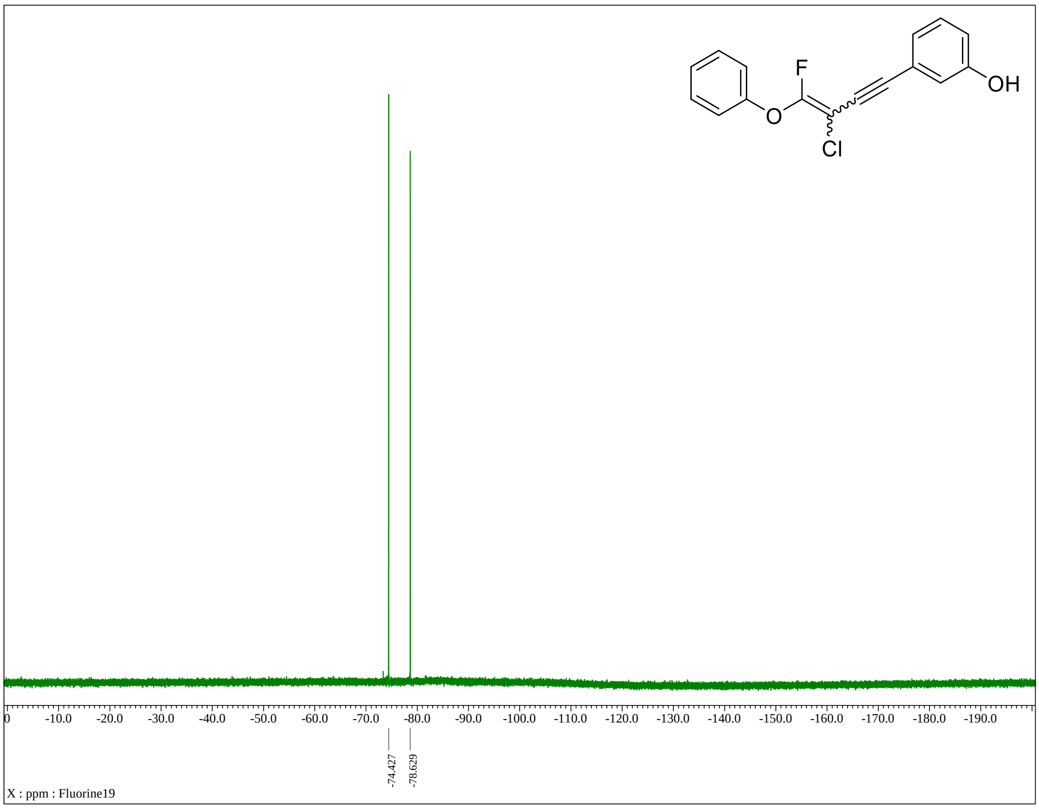

4-(4-Aminophenyl)-2-chloro-1-fluoro-1-phenoxybut-1-en-3-yne (3n)

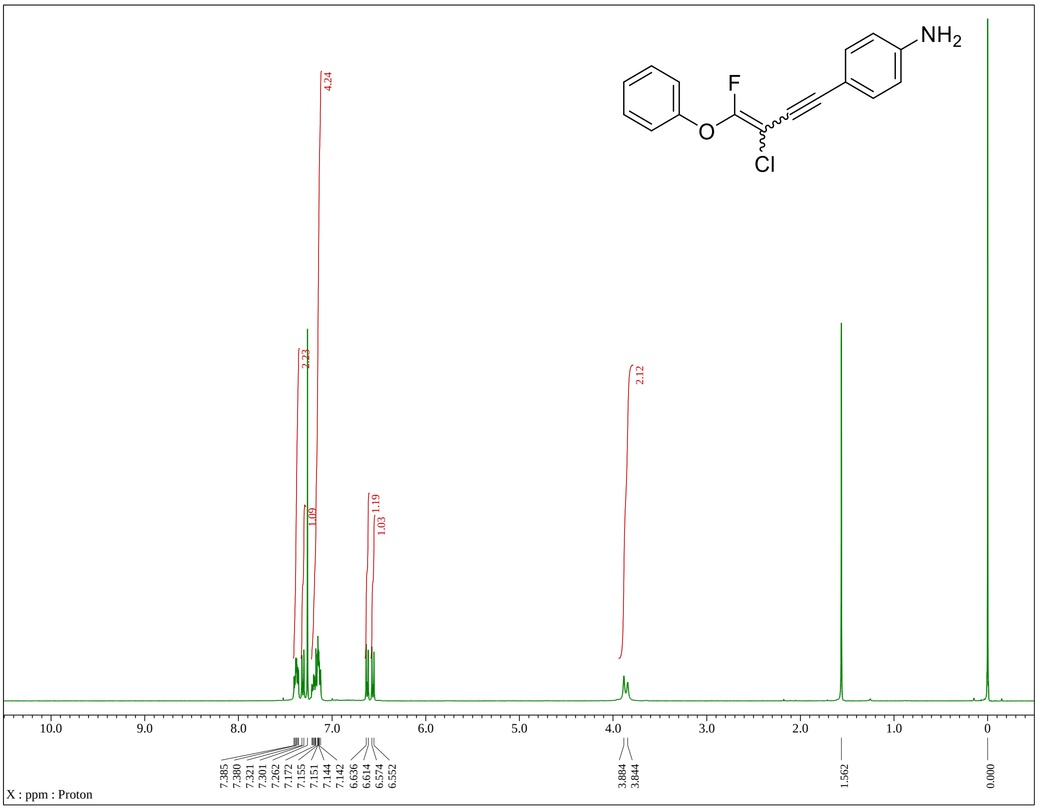

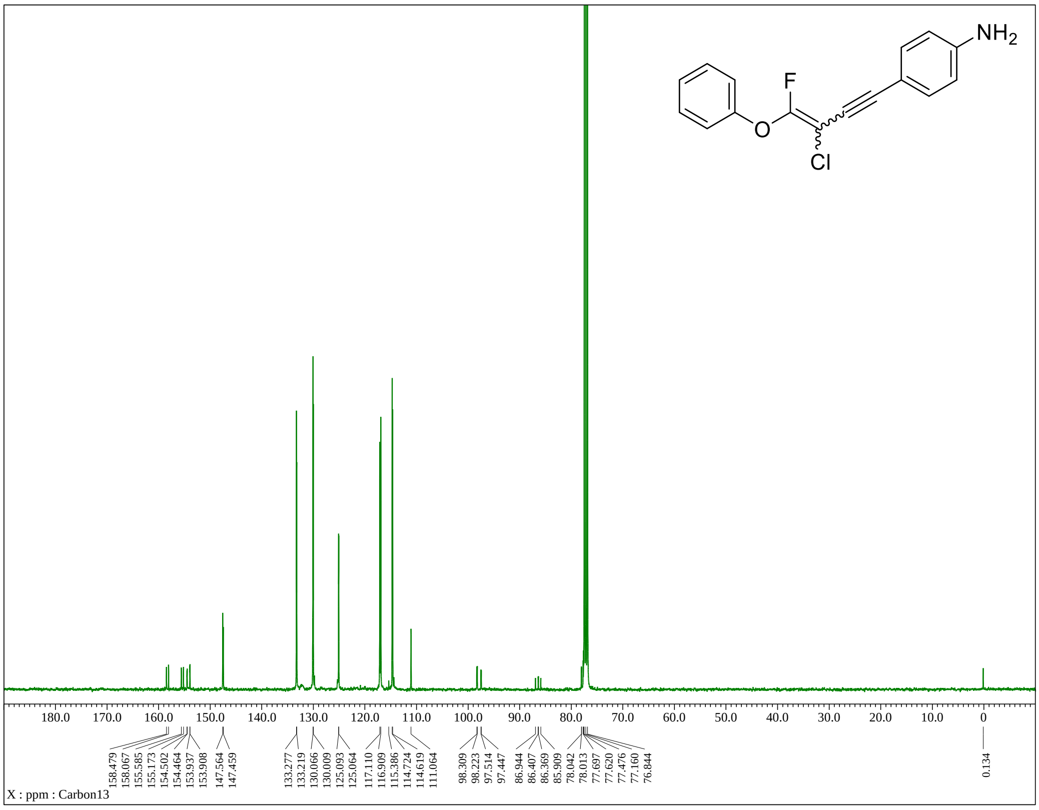
NH2
F
O
Cl
180.0 170.0 160.0 150.0 140.0 130.0 120.0 110.0 100.0 90.0 80.0 70.0 60.0 50.0 40.0 30.0 20.0 10.0 0
158.479 158.067 155.585 155.173 154.502 154.464 153.937 153.908 147.564 147.459
133.277 133.219 130.066 130.009 125.093 125.064 117.110 116.909 115.386 114.724 114.619 111.064
98.309 98.223 97.514 97.447 86.944 86.407 86.369 85.909 78.042 78.013 77.697 77.620 77.476 77.160 76.844
0.134
X : ppm : Carbon13

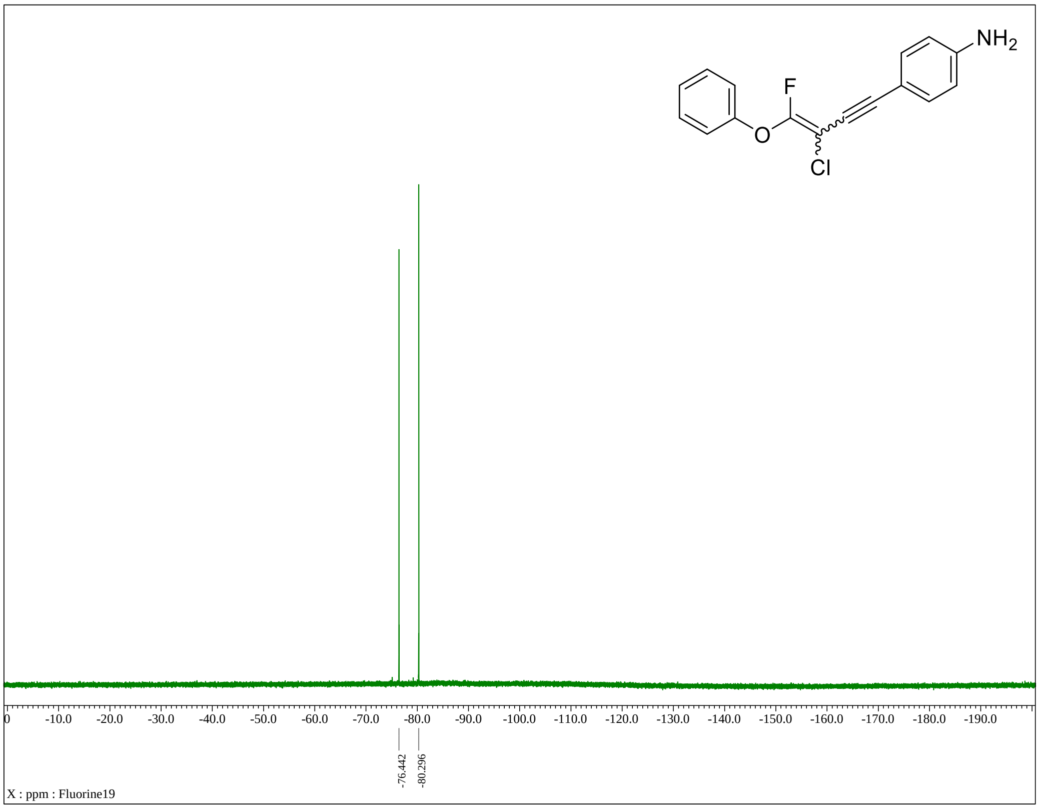
NH2
F
O
Cl
0 -10.0 -20.0 -30.0 -40.0 -50.0 -60.0 -70.0 -80.0 -90.0 -100.0 -110.0 -120.0 -130.0 -140.0 -150.0 -160.0 -170.0 -180.0 -190.0
-76.442
-80.296
X : ppm : Fluorine19

2-Chloro-1-fluoro-1-phenoxy-4-(thiophene-2-yl)-but-1-en-3-yne (3o)

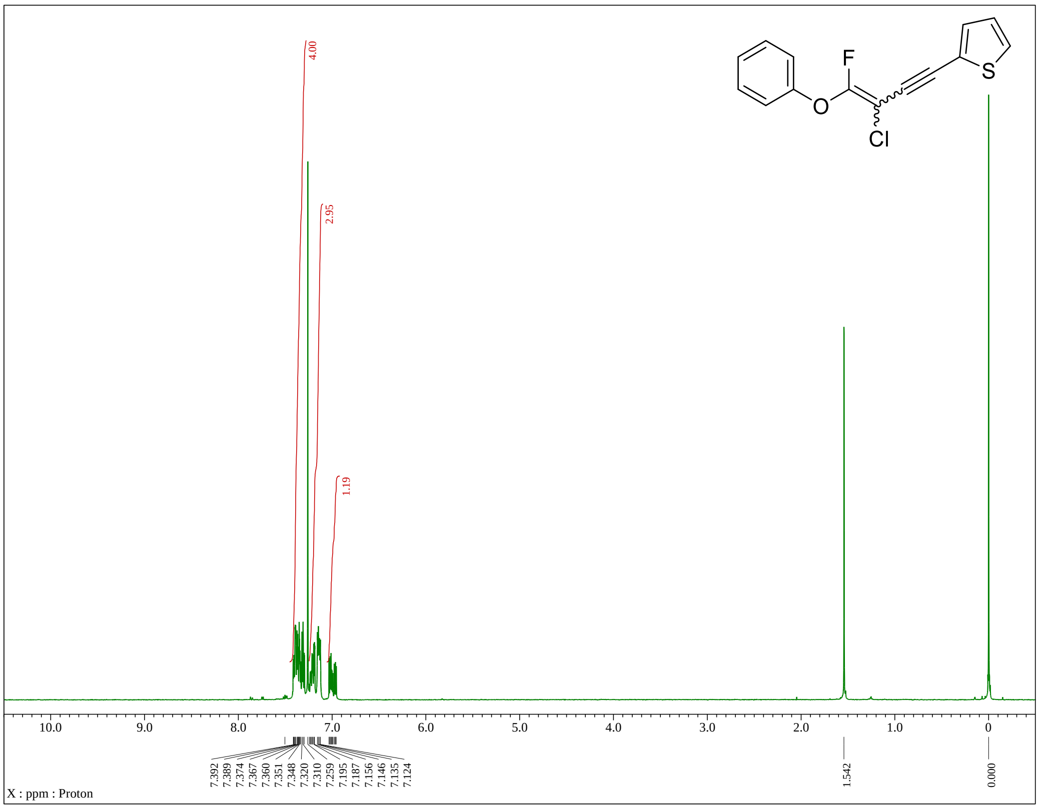

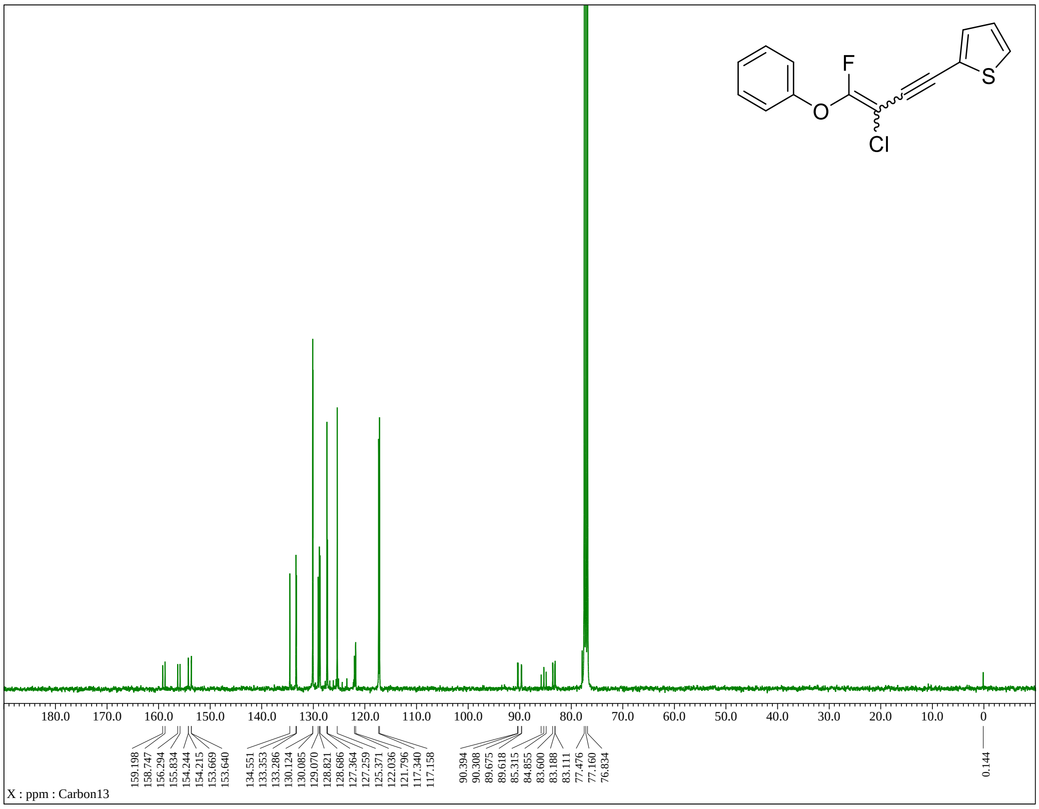

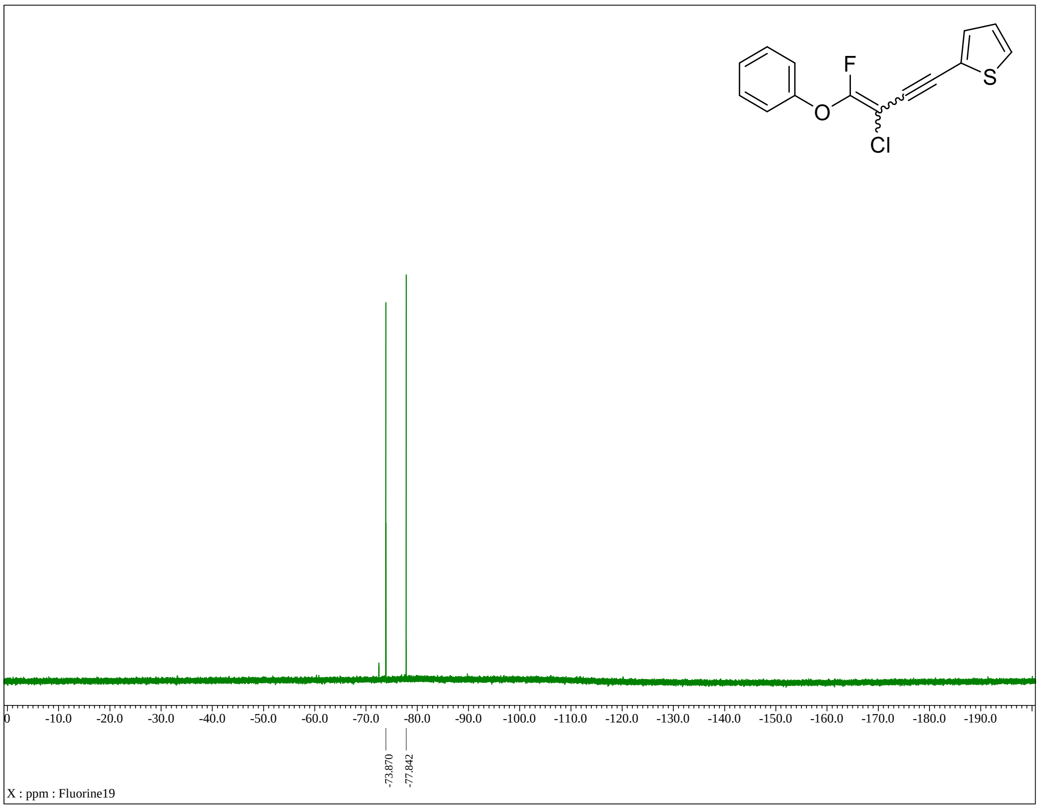

2-Chloro-1-fluoro-1-phenoxy-oct-1-en-3-yne (3p)

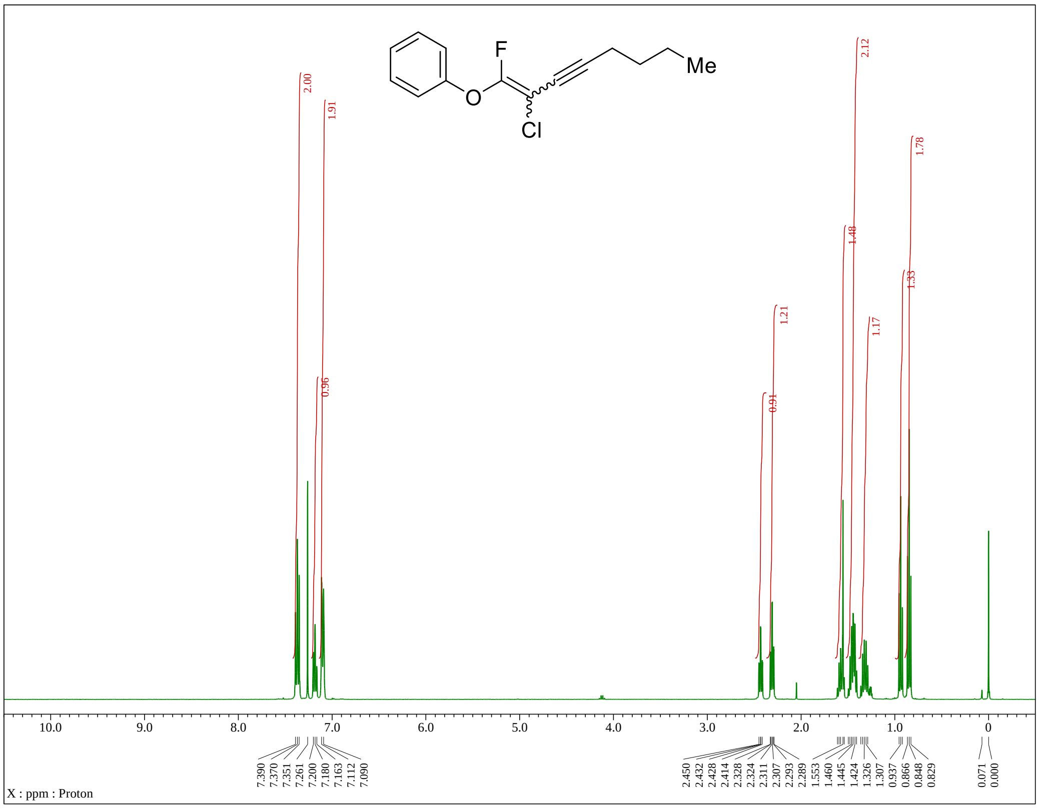

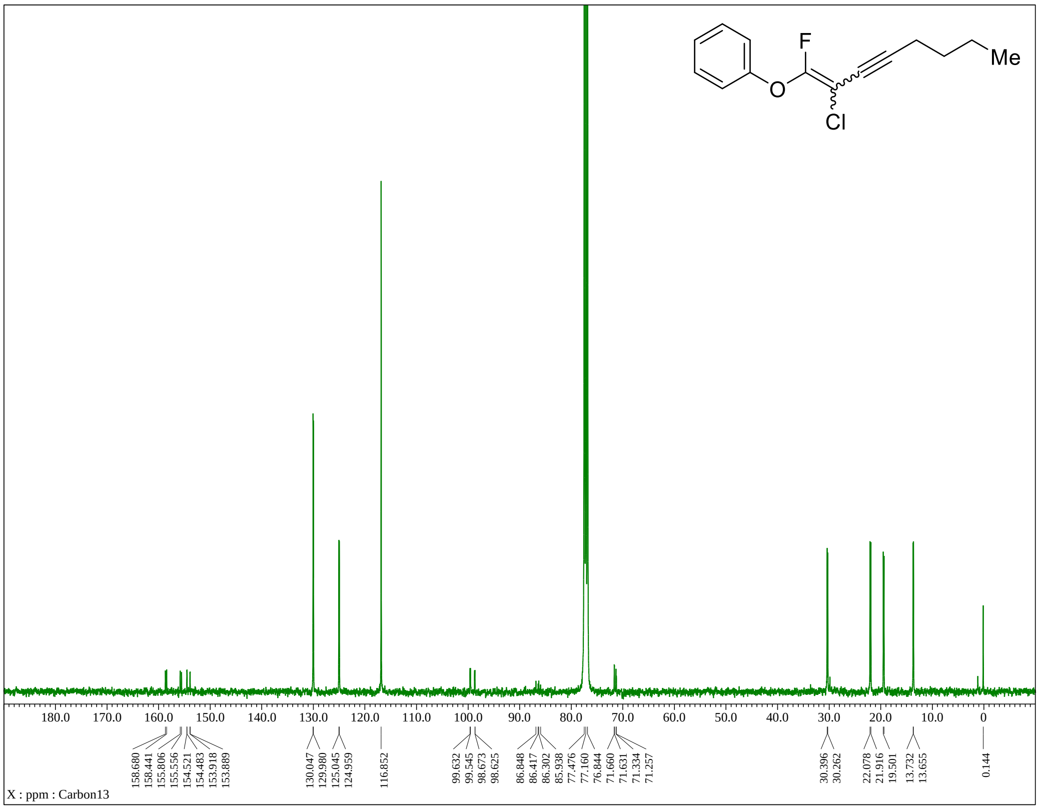
F
O
Cl
Me
180.0 170.0 160.0 150.0 140.0 130.0 120.0 110.0 100.0 90.0 80.0 70.0 60.0 50.0 40.0 30.0 20.0 10.0 0
158.680
158.441
155.806
155.556
154.521
154.483
153.918
153.889
130.047
129.980
125.045
124.959
116.852
99.632
99.545
98.673
98.625
86.848
86.417
86.302
85.938
77.476
77.160
76.844
71.660
71.631
71.334
71.257
30.396
30.262
22.078
21.916
19.501
13.732
13.655
0.144
X : ppm : Carbon13

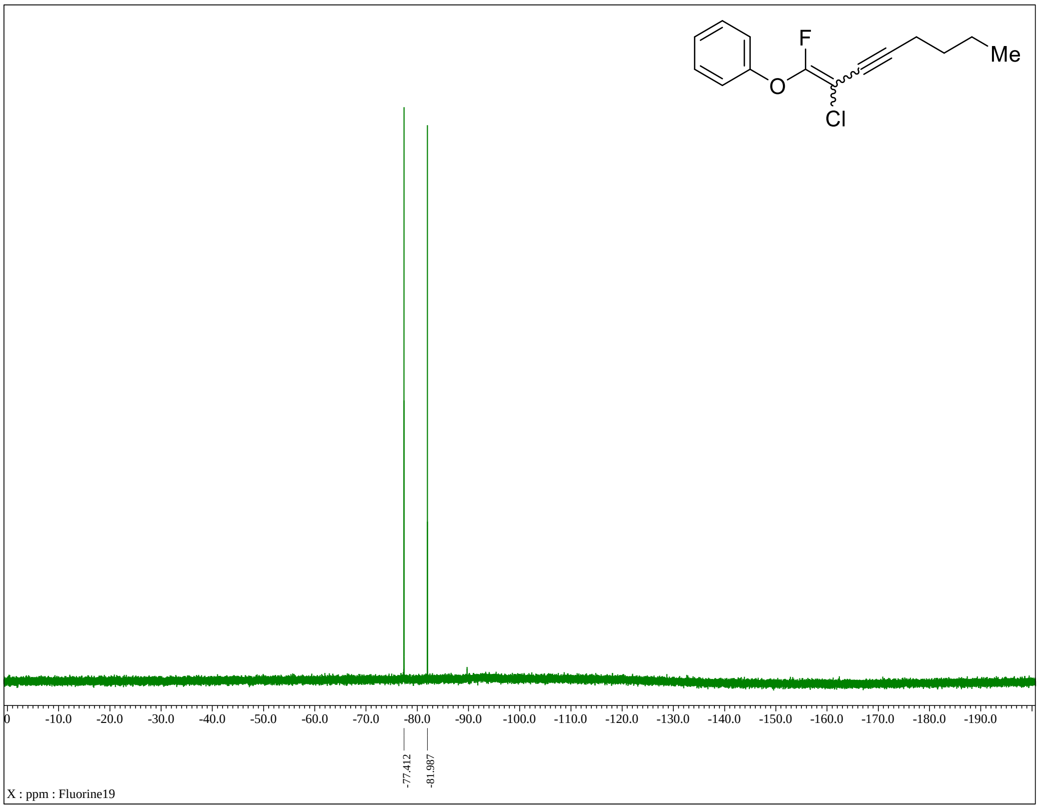
F
O
Cl
Me
0 -10.0 -20.0 -30.0 -40.0 -50.0 -60.0 -70.0 -80.0 -90.0 -100.0 -110.0 -120.0 -130.0 -140.0 -150.0 -160.0 -170.0 -180.0 -190.0
-77.412
-81.987
X : ppm : Fluorine19

2-Chloro-4-cyclopropyl-1-fluoro-1-phenoxy-but-1-en-3-yne (3q)

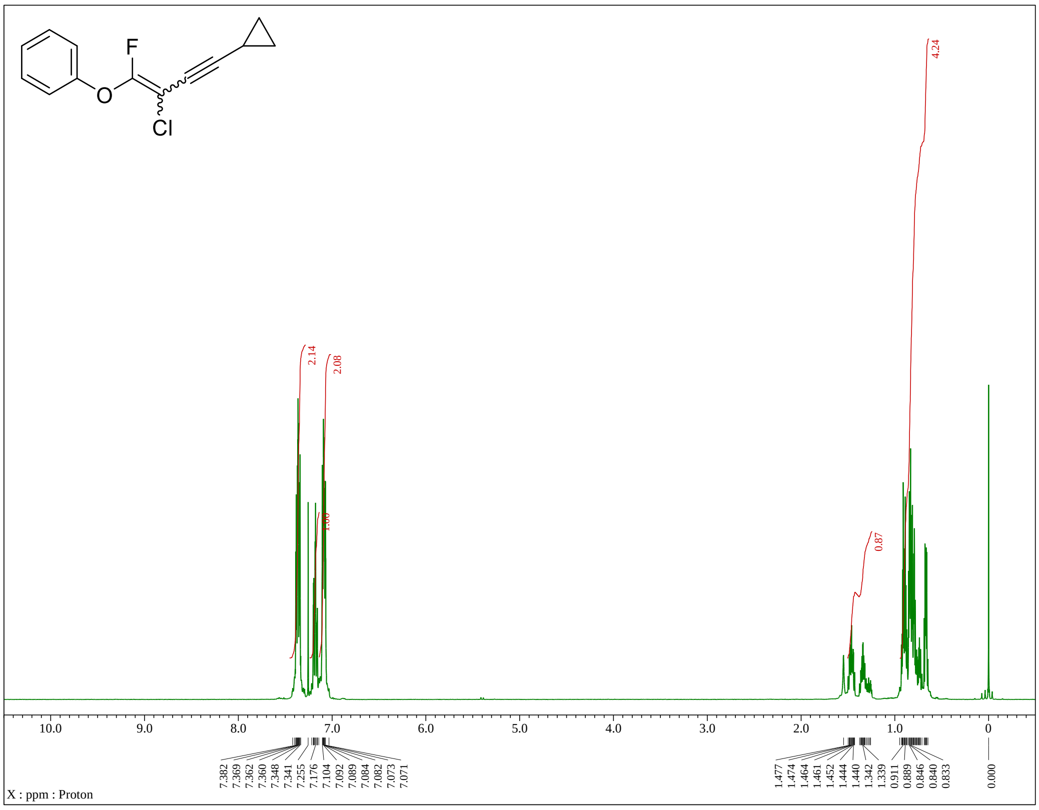

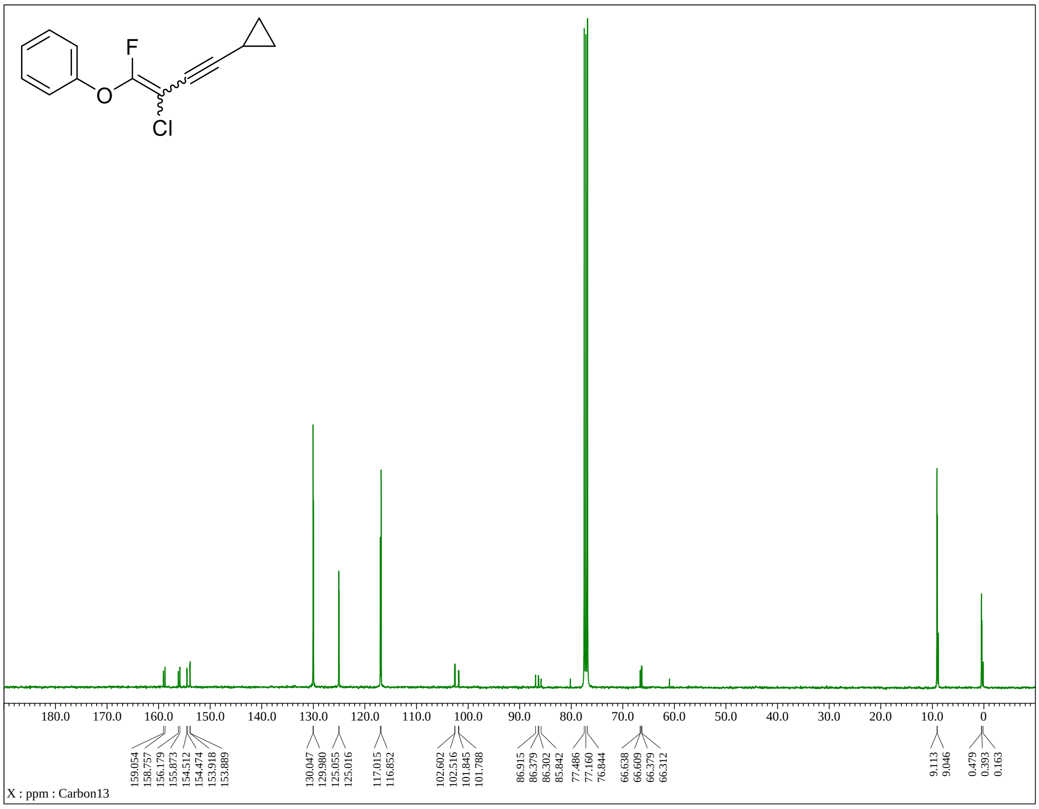

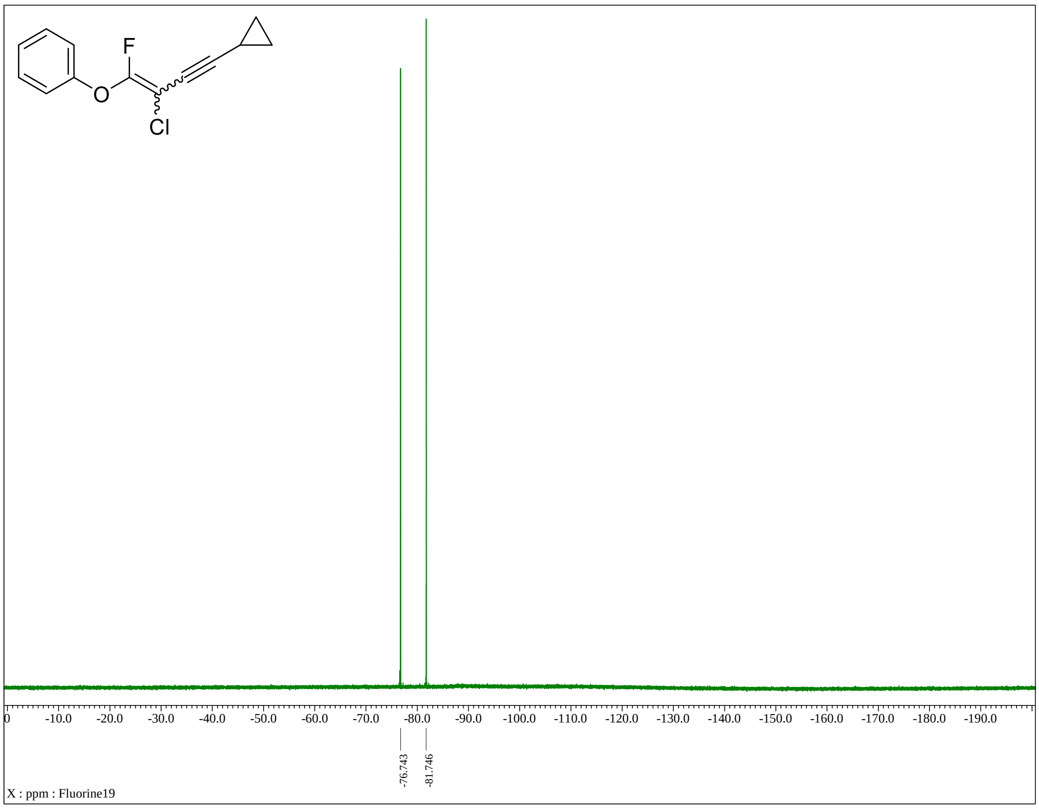

2-Chloro-4-cyclohex-1-yl-1-fluoro-1-phenoxy-but-1-en-3-yne (3r)

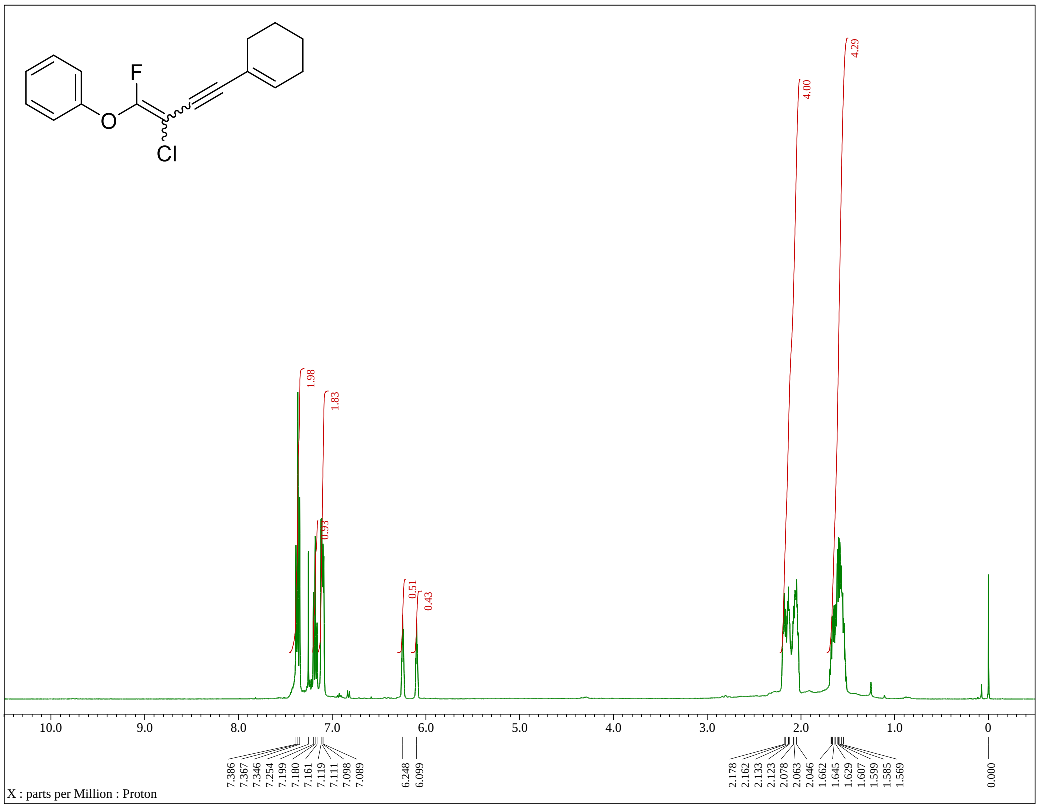

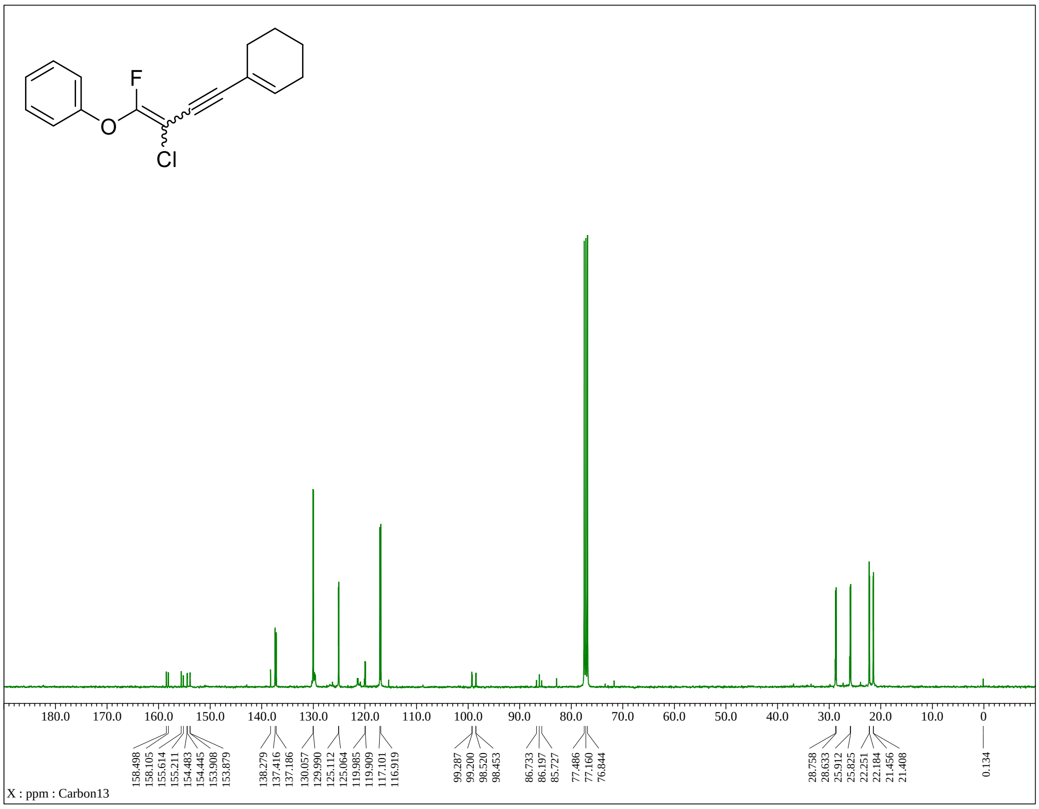
F
O
Cl
180.0 170.0 160.0 150.0 140.0 130.0 120.0 110.0 100.0 90.0 80.0 70.0 60.0 50.0 40.0 30.0 20.0 10.0 0
158.498 158.105 155.614 155.211 154.483 154.445 153.908 153.879
138.279 137.416 137.186 130.057 129.990 125.112 125.064 119.985 119.909 117.101 116.919
99.287 99.200 98.520 98.453
86.733 86.197 85.727
77.486 77.160 76.844
28.758 28.633 25.912 25.825 22.251 22.184 21.456 21.408
0.134
X : ppm : Carbon13

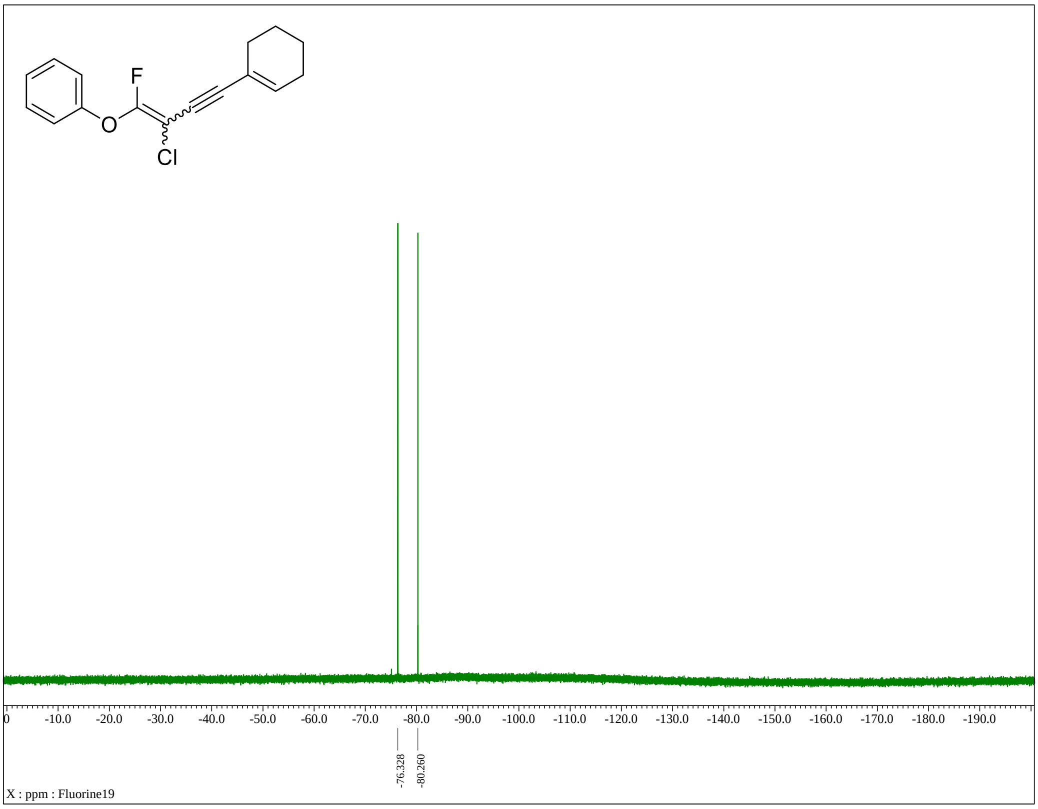
F
O
Cl
0 -10.0 -20.0 -30.0 -40.0 -50.0 -60.0 -70.0 -80.0 -90.0 -100.0 -110.0 -120.0 -130.0 -140.0 -150.0 -160.0 -170.0 -180.0 -190.0
-76.328
-80.260
X : ppm : Fluorine19

5-Chloro-6-fluoro-6-phenoxy-hex-5-en-3-yn-1-ol (3s)

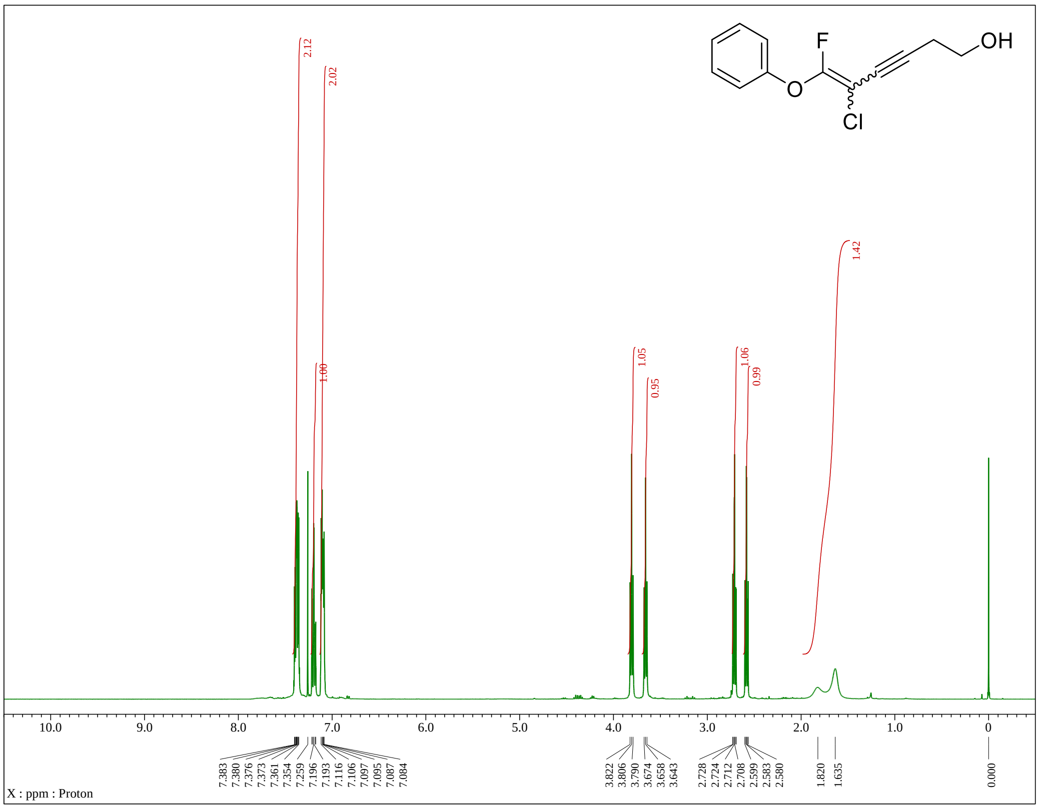

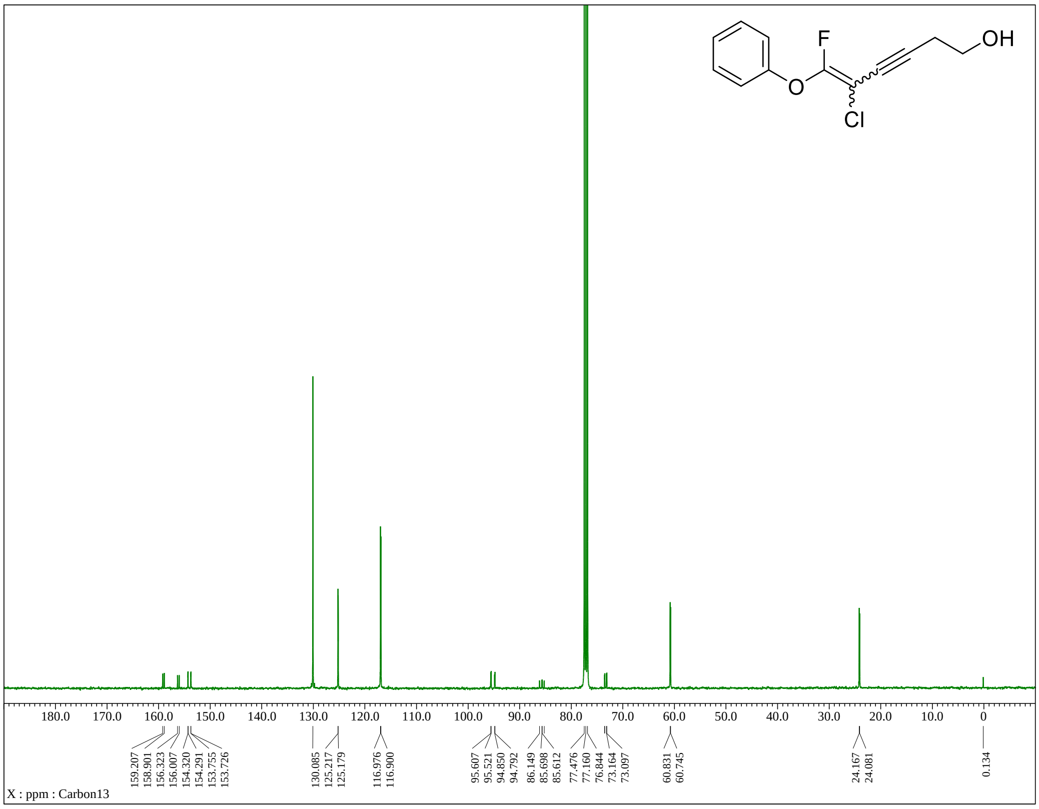

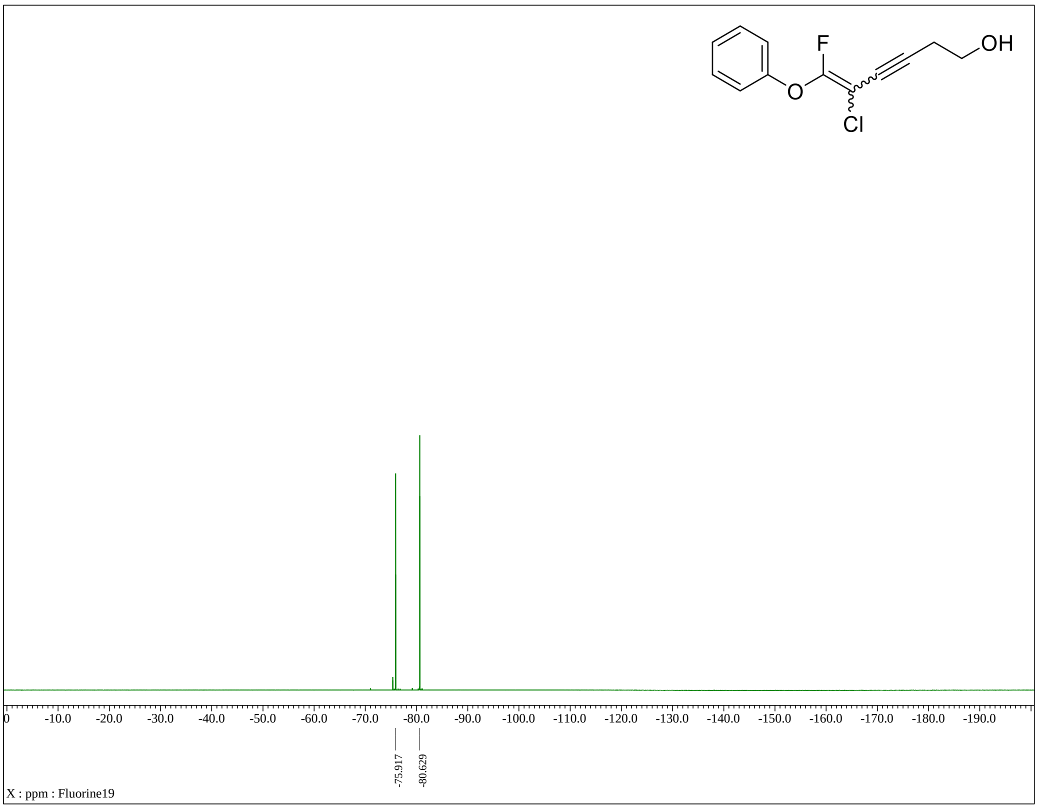

(3-Chloro-4-fluoro-4-(3-methoxyphenoxy)but-3-en-1-yn-1-yl)trimethylsilane (3t)

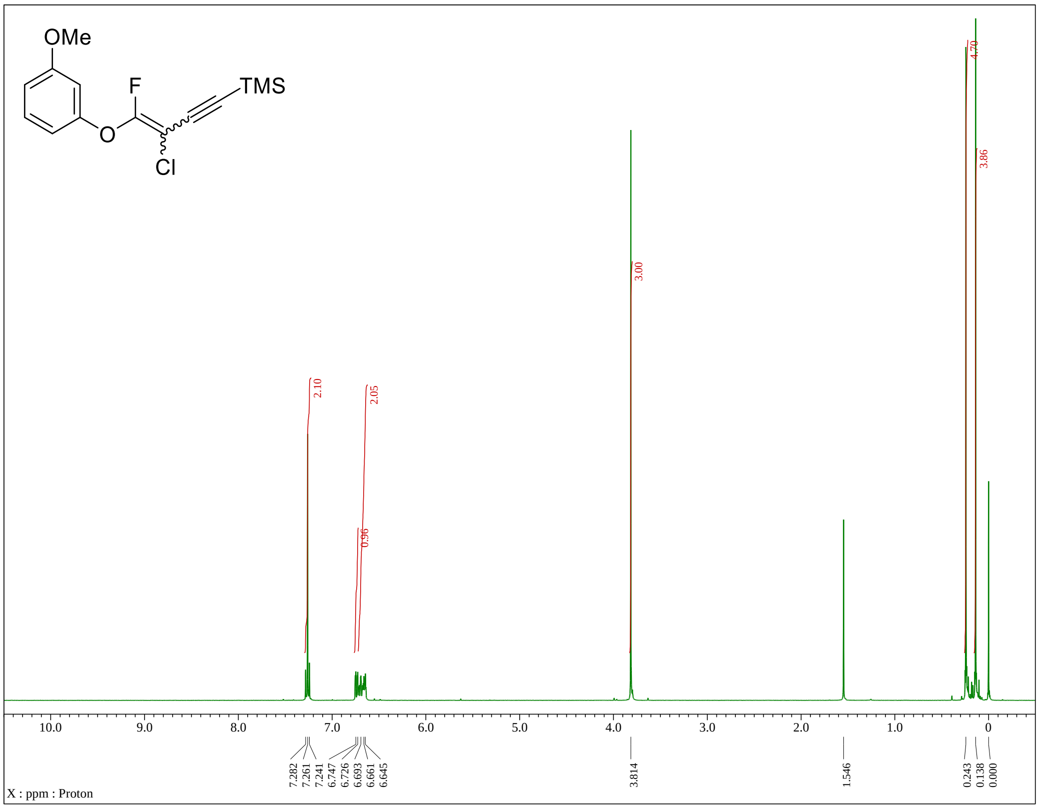

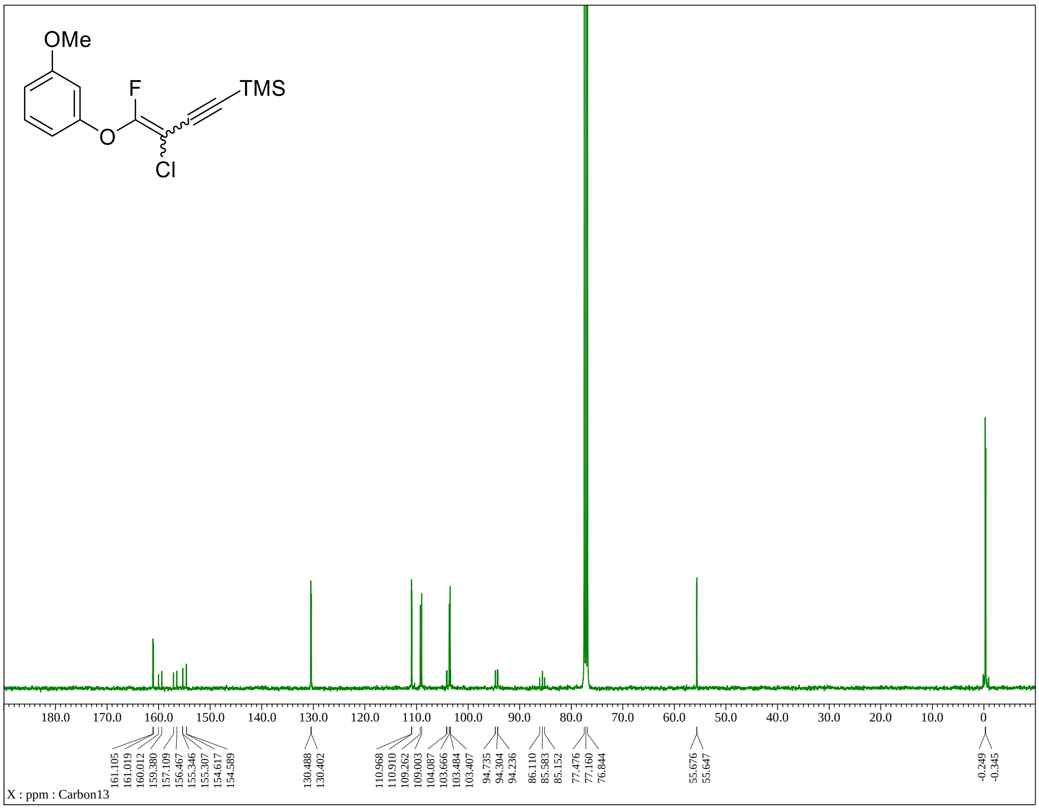
OMe
F
TMS
O
Cl
180.0 170.0 160.0 150.0 140.0 130.0 120.0 110.0 100.0 90.0 80.0 70.0 60.0 50.0 40.0 30.0 20.0 10.0 0
161.105 161.019 160.012 159.380 157.109 156.467 155.346 155.307 154.617 154.589
130.488 130.402
110.968 110.910 109.262 109.003 104.087 103.666 103.484 103.407 94.735 94.304 94.236 86.110 85.583 85.152 77.476 77.160 76.844
55.676 55.647
-0.249 -0.345
X : ppm : Carbon13

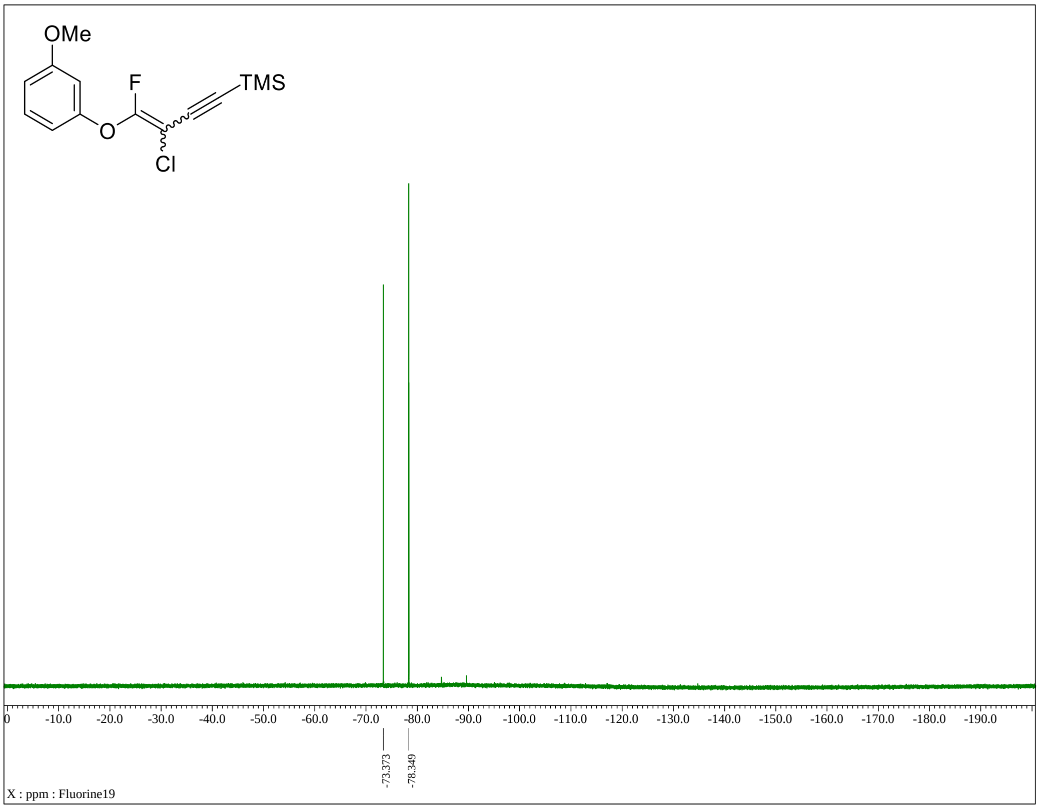
OMe
F
TMS
O
Cl
0 -10.0 -20.0 -30.0 -40.0 -50.0 -60.0 -70.0 -80.0 -90.0 -100.0 -110.0 -120.0 -130.0 -140.0 -150.0 -160.0 -170.0 -180.0 -190.0
-73.373
-78.349
X : ppm : Fluorine19

(3-Chloro-4-fluoro-4-(4-nitrophenoxy)but-3-en-1-yn-1-yl)trimethylsilane (3u)

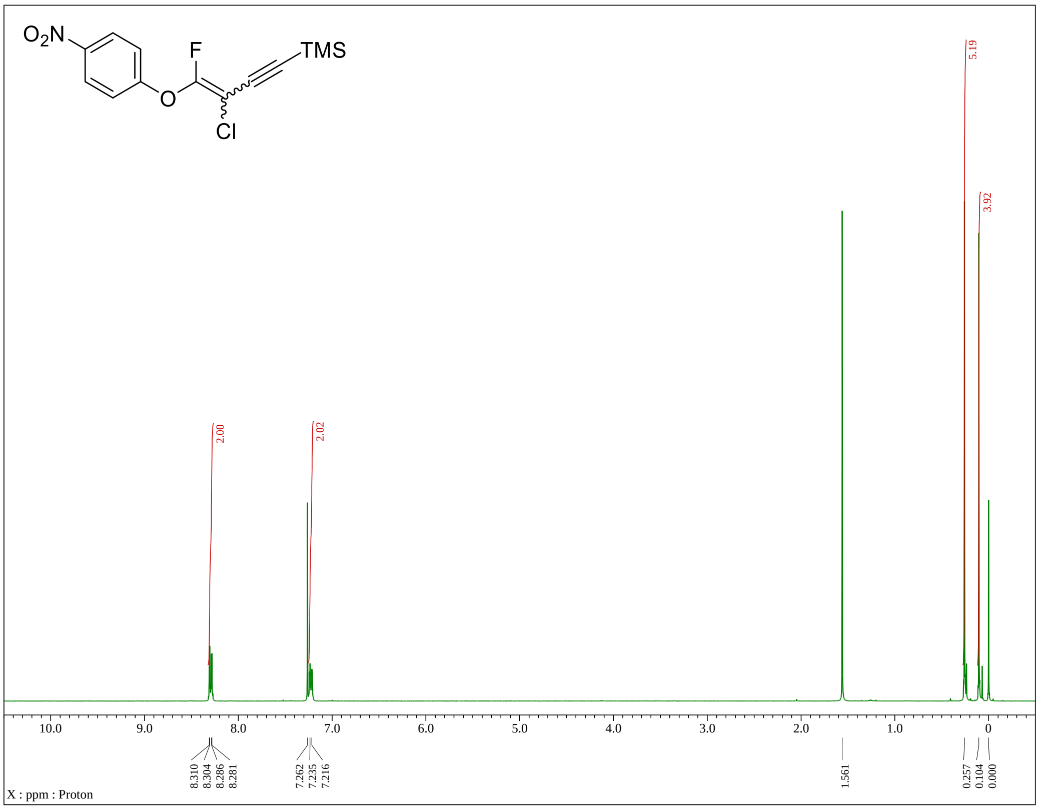

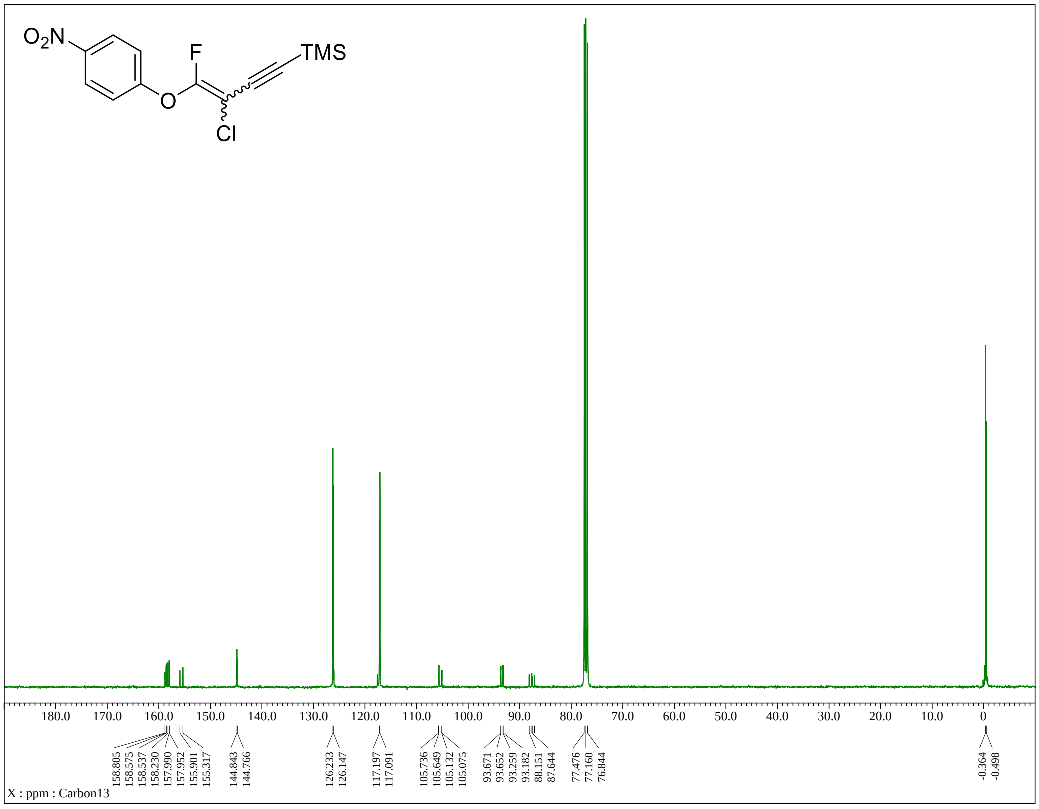

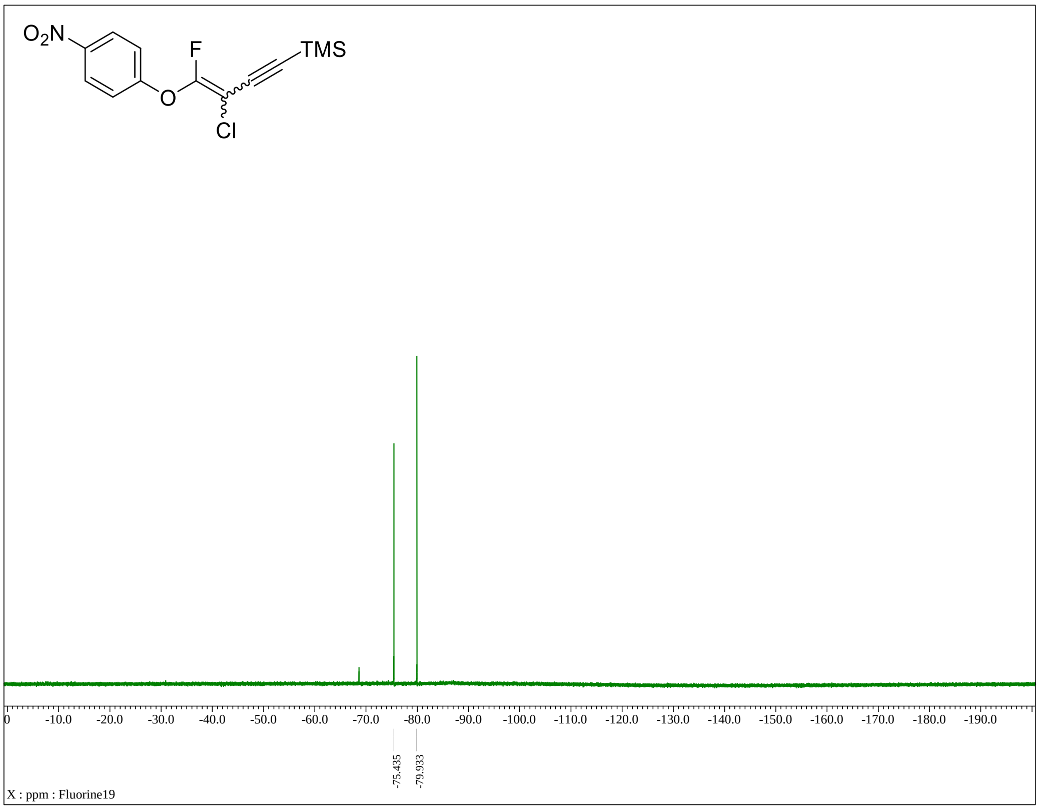

(3-Chloro-4-(4-ethoxycarbonylphenoxy)-4-fluorobut-3-en-1-yn-1-yl)trimethylsilane (3v)

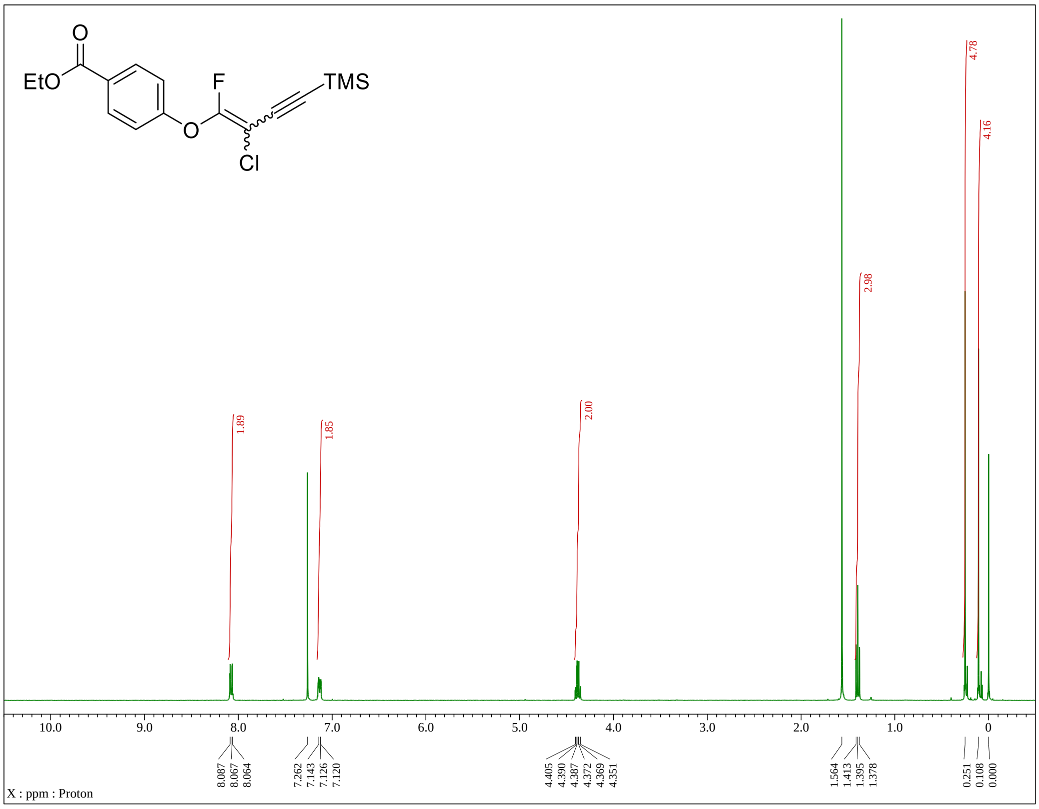

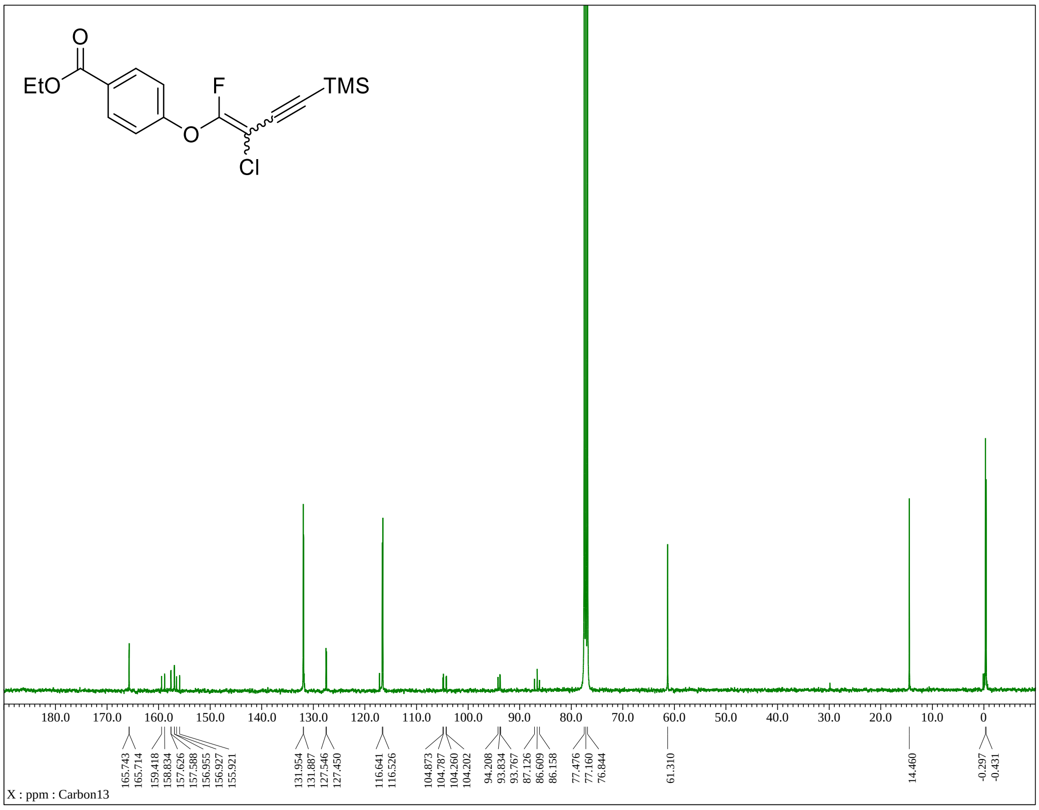
EtO
O
F
TMS
O
Cl
180.0 170.0 160.0 150.0 140.0 130.0 120.0 110.0 100.0 90.0 80.0 70.0 60.0 50.0 40.0 30.0 20.0 10.0 0
165.743
165.714
159.418
158.834
157.626
157.588
156.955
156.927
155.921
131.954
131.887
127.546
127.450
116.641
116.526
104.873
104.787
104.260
104.202
94.208
93.834
93.767
87.126
86.609
86.158
77.476
77.160
76.844
61.310
14.460
-0.297
-0.431
X : ppm : Carbon13

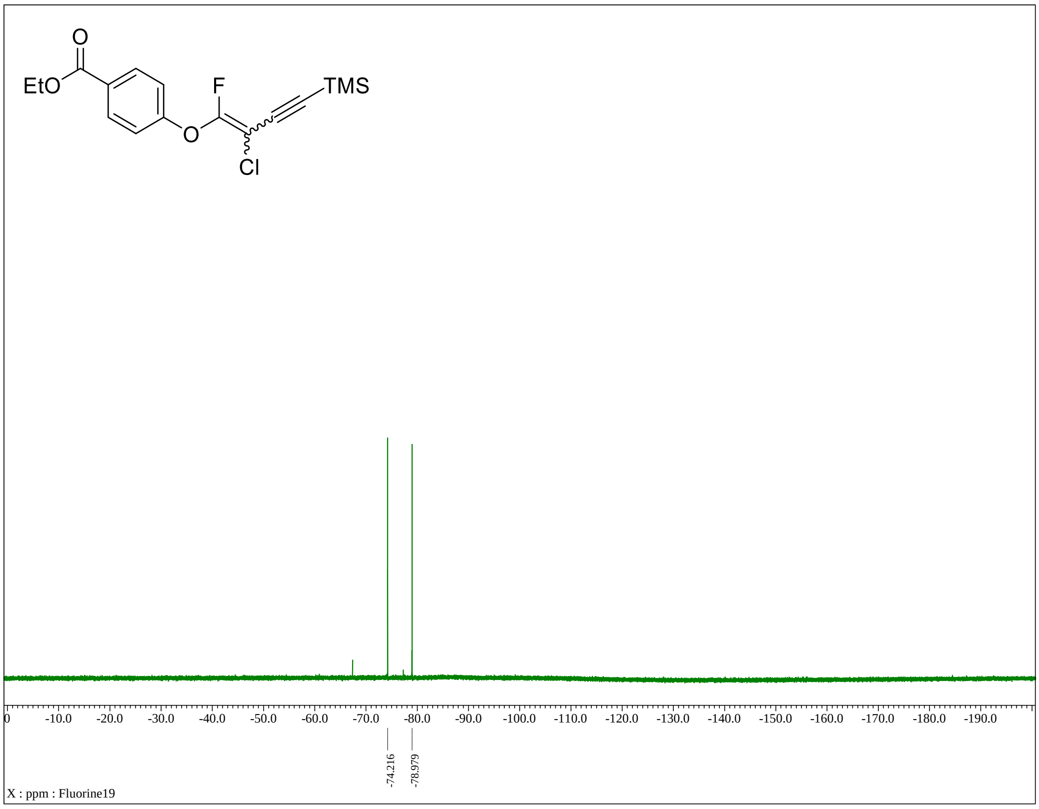
EtO
O
F
TMS
O
Cl
0 -10.0 -20.0 -30.0 -40.0 -50.0 -60.0 -70.0 -80.0 -90.0 -100.0 -110.0 -120.0 -130.0 -140.0 -150.0 -160.0 -170.0 -180.0 -190.0
-74.216
-78.979
X : ppm : Fluorine19

(4-(3-Aminophenoxy)-3-chloro-4-fluorobut-3-en-1-yn-1-yl)trimethylsilane (3w)

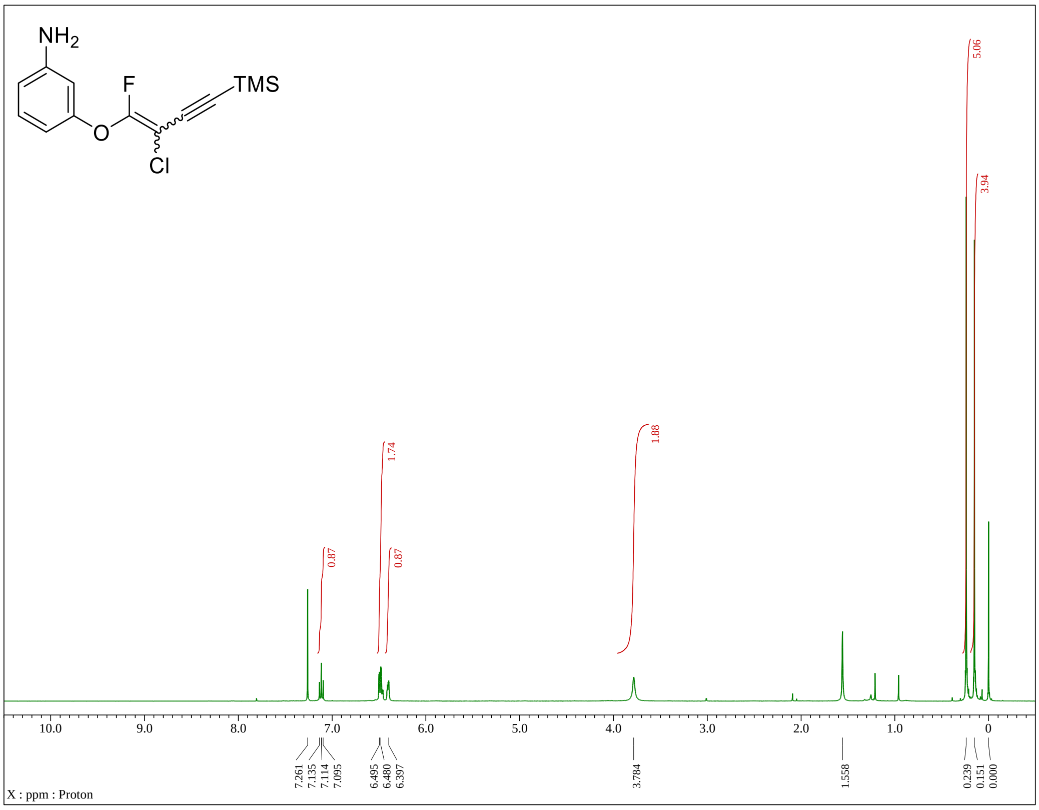

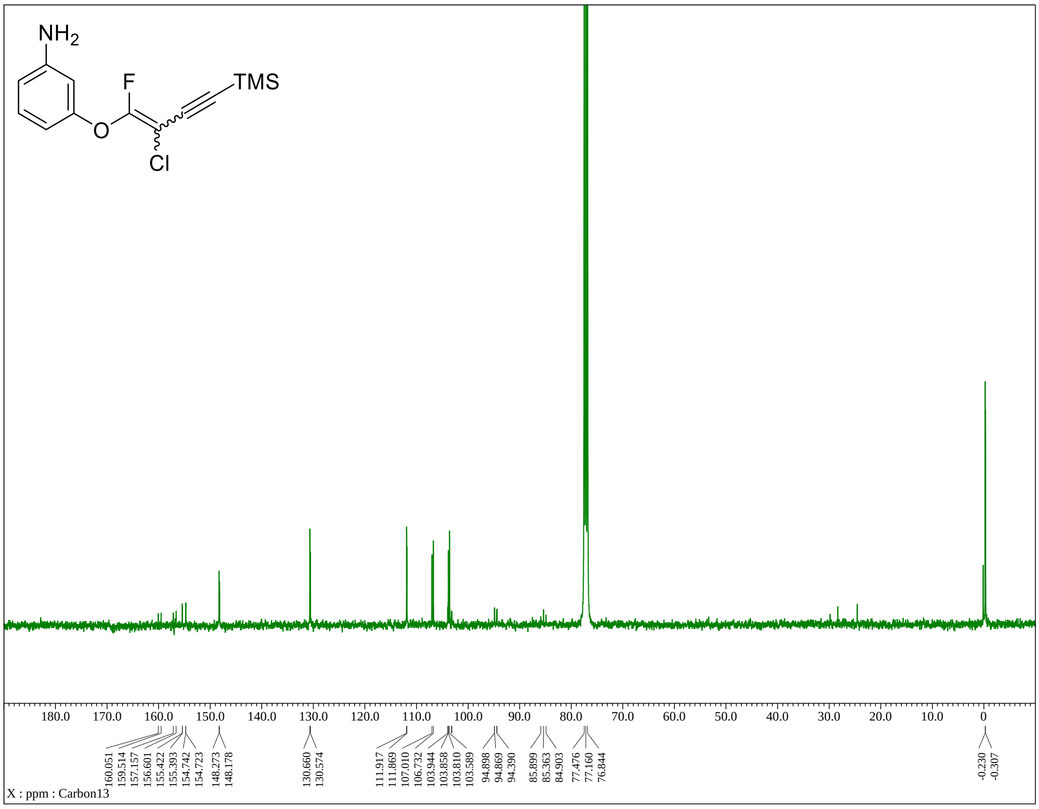

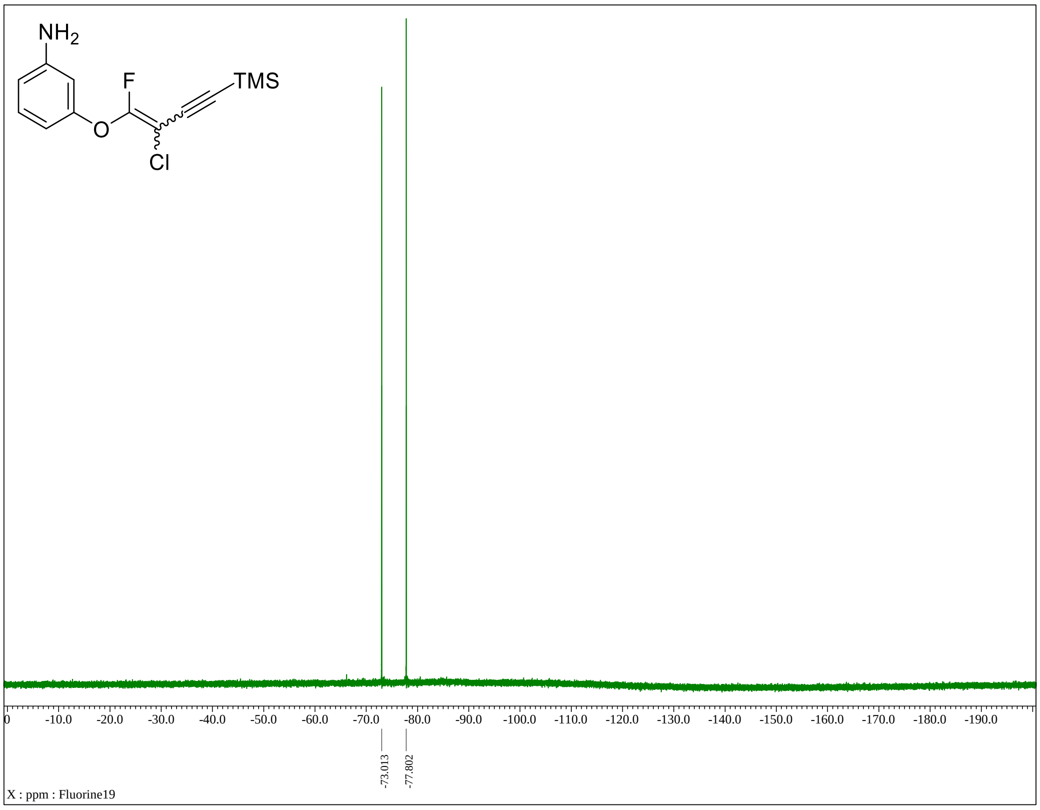

NH2
F
TMS
O
Cl
0
-10.0
-20.0
-30.0
-40.0
-50.0
-60.0
-70.0
-80.0
-90.0
-100.0
-110.0
-120.0
-130.0
-140.0
-150.0
-160.0
-170.0
-180.0
-190.0
-73.013
-77.802
X : ppm : Fluorine19
